# GATA4‐Driven Transcription of HtrA1 Promotes Cellular Senescence in Ménière's Disease and Age‐Related Audio‐Vestibular Dysfunction

**DOI:** 10.1002/advs.202512538

**Published:** 2026-04-14

**Authors:** Na Zhang, Na Li, Yan Wang, Jing Zhang, Jiahui Liu, Lei Chen, Yongdong Song, Yurong Mu, Yuechen Han, Yafeng Lyu, Xiaofei Li, Hanyue Wang, Jing Wang, Yao Lu, Zhaomin Fan, Daogong Zhang, Haibo Wang

**Affiliations:** ^1^ Department of Otolaryngology‐Head and Neck Surgery Shandong Provincial ENT Hospital Shandong University Jinan Shandong China; ^2^ Shandong Second Provincial General Hospital & Shandong Institute of Otorhinolaryngology & Shandong Key Laboratory of Deafness and Vertigo & Shandong Clinical Medical Research Center for Otolaryngological Diseases & Shandong Key Laboratory of Vertigo and Vestibular Medicine Jinan Shandong China; ^3^ Center of Clinical Laboratory Shandong Second Provincial General Hospital Jinan Shandong China

**Keywords:** age‐related audio‐vestibular dysfunction, cellular senescence, GATA4, HDAC6, HtrA1, Ménière's disease

## Abstract

Ménière's disease (MD), a chronic inflammatory disorder with age‐related increased incidence, exhibits poorly understood pathogenesis and limited therapeutic options. Here, we demonstrate that cellular senescence, marked by mitochondrial damage, reactive oxygen species accumulation, and senescence‐associated secretory phenotype (SASP), is prevalent in the vestibular tissue of MD patients and an endolymphatic hydrops mouse model. The transcription factor GATA4 is upregulated in MD and mice, and its genetic deletion in hair cells alleviates LPS‐induced audio‐vestibular dysfunction and cellular senescence in mice and HEI‐OC1 cells. Mechanistically, HDAC6 interacts with GATA4 and restrains its nuclear transport, while RNA‐seq and ChIP‐seq identify HtrA1, a serine protease, as a direct transcriptional target of GATA4. Inhibition of HDAC6 or AAV‐mediated HtrA1 overexpression exacerbates MD‐like symptoms, whereas inhibition of HtrA1 by Galegenimab ameliorates these phenotypes in mice. In aged mice, GATA4 deletion reduces age‐related audio‐vestibular deficits and senescence markers. Collectively, our findings establish GATA4 as a critical regulator of cellular senescence and inflammaging in inner ear pathologies, providing promising therapeutic targets for MD and age‐related audio‐vestibular disorders.

## Graphical abstract

This study identifies the HDAC6/GATA4/HtrA1 axis as a critical driver of cellular senescence in the inner ear. GATA4 nuclear translocation, facilitated by HDAC6 downregulation, transcriptionally activates HtrA1, promoting hair cell senescence, SASP, and audio‐vestibular dysfunction in models of Ménière's disease and age‐related audio‐vestibular dysfunction. Targeting this axis with an HtrA1‐inhibiting antibody ameliorates pathology, revealing a novel therapeutic strategy.

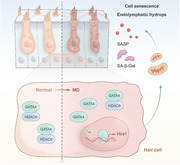

## 1 Introduction

Dizziness and deafness are prevalent health issues that cause significant communication difficulties and increase fall‐related mortality [1, 2]. These symptoms are more common with aging, as 45% of adults older than 65 years experience dizziness [3], and the prevalence of vestibular vertigo is 14% in the general population over 70 years [4]. Among various peripheral vestibular syndromes, Ménière's disease (MD) is chronic and heterogeneous, characterized by recurrent episodes of vertigo accompanied by cochlear symptoms such as fluctuating sensorineural hearing loss, tinnitus, and aural fullness [5]. The initial disease onset predominantly occurs between 40–60 years of age [6]. Its pathophysiology is associated with endolymphatic hydrops [7] and degeneration of vestibular end‐organs (VEOs) [8]. The pathogenesis of MD remains unclear and is thought to involve multifactorial dysfunction of the inner ear, including genetic factors, impaired endolymph production and absorption, and immune reactions [9]. However, the molecular and mechanistic links between aging and MD remain largely unknown.

The histopathological findings revealed that the neuroepithelium of VEOs in patients with MD exhibited varying degrees of degeneration, characterized by basement membrane thickening, cytoplasmic vacuolization, hair cells (HCs) loss, and extracellular matrix expansion, which are hallmarks of cellular senescence [8, 10]. Cellular senescence is characterized by stable growth arrest and altered gene expression patterns, including the upregulation of cell cycle inhibitors like p16^INK4a^ and p21^Cip1^ [11, 12]. Moreover, the secretion of a complex mixture of cytokines, chemokines, growth factors, and proteases, collectively termed the senescence‐associated secretory phenotype (SASP), is a hallmark of cellular senescence [13]. The chronic presence of senescent cells is closely linked to tissue dysfunction and age‐related chronic diseases [14, 15]. HCs are crucial sensory cells in the inner ear, which play a vital role in converting sound and mechanical stimuli into electrical signals that can be interpreted by the brain [16]. The function and integrity of HCs are closely related to the aging process [17, 18]. Notably, senescent HCs exhibit a persistent DNA damage response, progressive mitochondrial dysfunction [19, 20], and elevated pro‐inflammatory cytokines (e.g., IL‐6, TNF‐α, IL‐1β) that align with the SASP [21]. Accumulating evidence indicates elevated expression of inflammatory mediators in MD pathogenesis [22, 23], with particular emphasis on IL‐1β signaling pathways [24]. However, critical gaps persist in understanding HC senescence mechanisms, particularly the molecular crosstalk between senescent HCs and the cochlear microenvironment during MD progression.

Emerging as a key senescence regulator, GATA4 orchestrates the SASP through p53‐independent DNA damage response pathways in various tissues [25]. While GATA4 is known to be involved in osteoarthritis [26], intervertebral disc degeneration [27], and liver fibrosis [28], its role in the audio‐vestibular senescence remains enigmatic. In this study, we demonstrated that GATA4 was upregulated with senescence markers and SASP components in VEOs from MD patients and EH mouse models, and that genetic deletion of GATA4 attenuated senescence in vitro and inner ear dysfunction in vivo. Notably, HDAC6 declines during hair cell senescence, facilitating GATA4 nuclear translocation. We identified HtrA1 as a direct GATA4 target and showed that HtrA1 inhibition suppressed SASP and improved audio‐vestibular function, whereas HtrA1 overexpression exacerbated degeneration. These findings underscore the critical role of the GATA4/HtrA1 axis in driving inner ear senescence and audio‐vestibular degeneration, establishing it as a novel therapeutic target for MD.

## 2 Results

### 2.1 Vestibular Organs in Ménière's Disease Exhibit Senescence‐Associated Molecular Alterations

**FIGURE 1:**
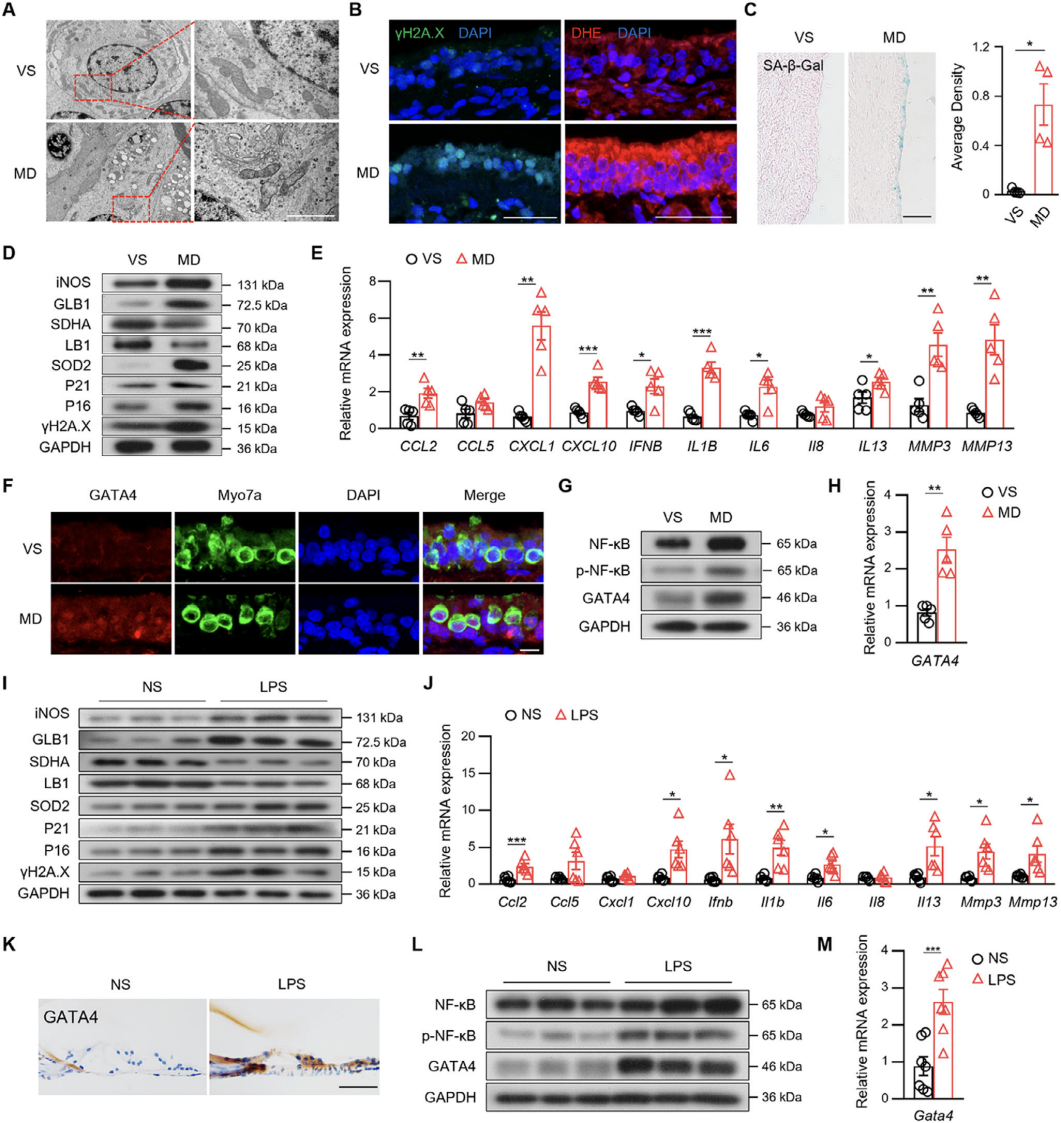
HC senescence and upregulation of GATA4 in patients with MD. (A) The representative electron microscopy images of VEOs of VS and MD (scale bar, 1 µm). (B) Representative confocal microscopy images showing γH2A.X (green), DHE (red), and DAPI (blue) staining in the VEOs of VS and MD patients (scale bar, 10 µm). (C) Representative images (left) and quantification (right) of SA‐β‐gal staining in VEOs of VS (*n* = 4) and MD (*n* = 4) (scale bar, 50 µm). (D) Western blot analysis of senescence‐associated protein expression in VEOs of VS and MD. (E) The mRNA levels of SASP in VEOs of VS (*n* = 5) and MD (*n* = 5). (F) Representative confocal microscopy images showing GATA4 (red), Myo7a (green), and DAPI (blue) staining in the VEOs of VS and MD patients (scale bar, 10 µm). (G) Western blot analysis of GATA4 and NF‐κB expression in VEOs of VS and MD. (H) The mRNA levels of GATA4 in VEOs of VS (*n* = 5) and MD (*n* = 5). (I) Western blot analysis of senescence‐associated protein expression in cochleae of mice treated with LPS or saline. (J) The mRNA levels of SASP in cochleae of mice treated with LPS or saline (*n* = 6). (K) The distribution of GATA4 in the cochlea was determined by immunohistochemistry in mice treated with treated with LPS or saline. (L) Western blot analysis of GATA4 and NF‐κB expression in cochleae of mice treated with LPS or saline. (M) The mRNA levels of GATA4 in cochleae of mice treated with LPS (*n* = 7) or saline (n = 7). Data are shown as mean ± SEM. Data were analyzed by unpaired t test [(C), (E), (H), (J), and (M)]. *p < 0.05, **p < 0.01, ***p < 0.001.

Initially, we investigated the cellular senescence status in the VEOs of MD patients. Transmission electron microscopy identified ultrastructural mitochondrial abnormalities in vestibular HCs of MD patients, including cristae loss, membrane discontinuity, and marked swelling (Figure 1A). Immunofluorescence analysis in vestibular hair cells revealed elevated ROS accumulation (Figure 1B, Figure S1A), concurrent with upregulated iNOS/SOD2 and decreased SDHA—a key component of mitochondrial Complex II essential for redox homeostasis (Figure 1D). Senescence hallmarks were further confirmed through nuclear γH2A.X foci accumulation [29] (Figure 1B, Figure S1B), enhanced SA‐β‐Gal activity in hair cells (Figure 1C and Figure S1C), and coordinated upregulation of senescence effectors (GLB1, P21, P16) (Figure 1D and Figure S1D,E). Notably, MD tissues exhibited reduced Lamin B1 expression (Figure 1D), indicating compromised nuclear integrity [29]. Additionally, the expression of the SASP in the VEOs of MD patients was remarkably higher compared to that in VS patients (*CCL2*, *CXCL1*, *CXCL10*, *IFNB*, *IL1B*, *IL6*, *IL13*, *MMP3*, *MMP13*; Figure 1E).

GATA4/NF‐κB is thought to promote cellular senescence by upregulating the production of the SASP [30]. To investigate the potential role of transcription factor GATA4 in the regulation of cellular senescence in MD, we next examined its expression in both MD patients and EH mouse model. We observed upregulation of GATA4 and activation of NF‐κB in MD VEOs relative to VS patients, with particularly strong nuclear localization in hair cells (Figure 1F–H, Figure S1F). An endolymphatic hydrops (EH) mouse model induced by LPS treatment was examined to assess these findings. Consistent with human data, LPS‐treated mice exhibited increased senescence markers (γH2A.X, P21) and SASP factors in the cochlea (Figure 1I,J, Figure S1G,H), coupled with reduced LB1 and SDHA expression (Figure 1I). Simultaneously, GATA4 expression and NF‐κB activation were elevated in these mice (Figure 1K–M, Figure S1I). In summary, the above results demonstrate the presence of cellular senescence and SASP in the inner ear of both MD patients and EH mice, strongly suggesting that GATA4 may play a critical role in this pathological process.

### 2.2 GATA4 Deletion in Hair Cells Ameliorates LPS‐Induced Audio‐Vestibular Dysfunction and Cellular Senescence

**FIGURE 2:**
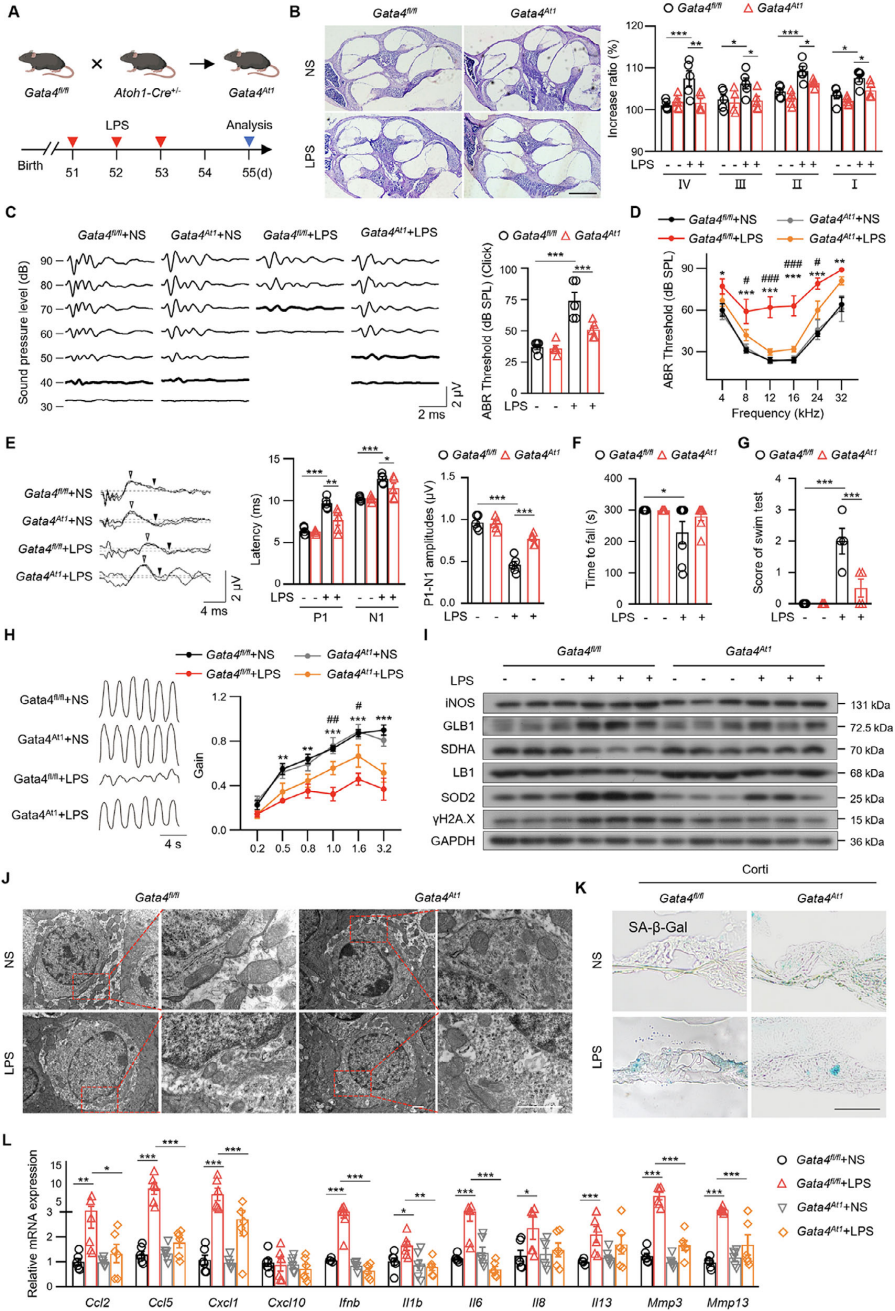
Genetic deletion of GATA4 in hair cells ameliorates LPS‐induced audio‐vestibular symptoms and cellular senescence in mice. (A) Schematic representation of the crossbreeding strategy to generate hair‐cell‐specific Gata4‐knockout mice and the time scales for analysis. The *Atoh1‐Cre* ^+/−^;*Gata4^fl/fl^* mice (referred to as *Gata4^At1^*) or *Atoh1‐Cre* ^+/+^;*Gata4^fl/fl^* mice (referred to as *Gata4^fl/fl^*) were treated with LPS (10 mg/kg, p.a.) for 3 consecutive days at P51, then analyzed at 55 days. (B) Representative images of mid‐modiolar cochlear sections, scale bar = 100 µm. Measurements of IR‐L in cochlear half‐turns I–IV (*n* = 5). (C) Representative serial ABR wave recordings and thresholds in response to click sounds (*n*=5). (D) ABR thresholds in response to pure tone bursts across all frequencies (*n* = 6). * significant difference compared with *Gata4^fl/fl^+*NS and *Gata4^fl/fl^+*LPS; # significant difference compared with *Gata4^fl/fl^+*LPS and *Gata4^At1^+*LPS. (E) Representative click‐evoked VEMP waves, the P1 (white triangle) and N1 (black triangle) peak latencies, and P1‐N1 peak amplitudes of VEMPs at 100 dB nHL (*n* = 5). (F) Quantification of rotarod test (*n* = 7). (G) Swimming posture scores of swim tests (*n* = 4). (H) Representative horizontal VOR waves of all groups. The VOR gains and phases are plotted for all groups at a peak velocity (20°/s) and frequencies range from 0.2 Hz to 3.2 Hz (*n*=6), * significant difference compared with *Gata4^fl/fl^+*NS and *Gata4^fl/fl^+*LPS; # significant difference compared with *Gata4^fl/fl^+*LPS and *Gata4^At1^+*LPS. (I) Western blot analysis of senescence‐associated protein expression in the inner ear. (J) The representative electron microscopy images of vestibular HCs of mice (scale bar, 1 µm). (K) Representative images of SA‐β‐gal staining in the HCs (scale bar, 50 µm). (L) The mRNA levels of SASP in the inner ear (*n* = 6). Data are shown as mean ± SEM. Data were analyzed by one‐way ANOVA with LSD multiple comparisons posttests [(B), (C), (D), (E), (F), (G), (H), and (L)]. * or # *p* < 0.05, ** or ## *p* < 0.01, *** or ### *p* < 0.001.

The lipopolysaccharide (LPS)‐induced MD model was employed to determine how GATA4‐regulated senescence dynamics modulate MD‐associated pathophenotypes [22]. To investigate the potential role of GATA4 in regulating clinically relevant MD pathophysiology, we employed an LPS‐induced MD mouse model and validated this using conditional Gata4 knockout mice with HC‐specific gene deletion. Specifically, we postauricularly (p.a.) injected LPS to age‐ and sex‐matched *Atoh1*‐*Cre* ^+/−^;*Gata4* ^fl/fl^ (*Gata4^At1^*) and littermate *Atoh1*‐*Cre* ^−/−^;*Gata4* ^fl/fl^ (*Gata4^fl/fl^*) mice for 3 consecutive days, followed by comprehensive evaluation of endolymphatic hydrops (EH), auditory‐vestibular dysfunction, and senescence‐related biomarkers (Figure 2A). In these *Gata4^At1^* mice, GATA4 signals were absent in HCs but still observed in spiral ganglion neurons, verifying our detection of GATA4 expression in the cochlea and the HC specificity of the *Gata4^At1^* mouse (Figure S2A,B). After administration of LPS, *Gata4^At1^* mice exhibited significantly reduced EH in cochlear half‐turns I–IV than did *Gata4^fl/fl^* mice (Figure 2B), without affecting body weight (Figure S2C). Assessment of hearing function via auditory brainstem response (ABR) revealed that *Gata4^At1^* mice reversed LPS‐induced threshold increases in response to click and tone burst stimuli across the 8–24 kHz frequency range (Figure 2C,D).

Vestibular deficits were also attenuated in *Gata4^At1^* mice, as evidenced by shorter latencies of vestibular‐evoked myogenic potentials (VEMPs) P1/N1 peaks (Figure 2E), prolonged rotarod latency (Figure 2F), and improved swimming posture scores (Figure 2G). Furthermore, vestibular ocular reflex (VOR) gain deficits at 1.0 and 1.6 Hz were reversed in *Gata4^At1^* mice (Figure 2H). Thus, GATA4 deficiency decreased the severity of hydrops and ameliorated damage to auditory and vestibular function in the LPS‐induced MD mouse model. Examination of senescence‐related markers revealed that the LPS‐induced reduction of iNOS, GLB1, SOD2, and γH2A.X, and the upregulation of SDHA and LB1 in the inner ear of *Gata4^At1^* mice compared to *Gata4^fl/fl^* mice (Figure 2I). Immunofluorescence analysis also revealed decreased P16 in the inner ear of *Gata4^At1^* mice (Figure S2D). Furthermore, quantitative analysis of TEM images showed that the proportion of healthy mitochondria was significantly higher in *Gata4^At1^* mice compared to *Gata4^fl/fl^* mice after LPS challenge (Figure 2J, Figure S2E). SA‐β‐gal staining indicated lower senescence activity in the inner ear of *Gata4^At1^* mice (Figure 2K, Figure S2F). Additionally, mRNA levels of SASP were significantly decreased in the inner ears of *Gata4^At1^* mice (Figure 2L). This suggests that GATA4 enhanced cellular senescence in vivo.

The HEI‐OC1 cell line, which demonstrates morphological and functional homology to hair cells while retaining their characteristic biomarkers, has emerged as a principal model system for investigating molecular mechanisms underlying hair cell degeneration and potential therapeutic interventions [31]. Doxorubicin has been widely used in aging research [32, 33]. In our experiments to delineate aging‐induced HC damage, we induced senescence in HEI‐OC1 cells using varying concentrations of Dox over 24 h and cultured them for 0, 24, 48, or 72 h. Cell viability assessments using the CCK‐8 reagent revealed a notable decline in cell survival at concentrations exceeding 0.5 µM and 48 h (Figure S3A,B). Additionally, the expression of P21 and P16 increased significantly and there was mitochondrial damage at 48 h post‐treatment (0.5 µM Dox) (Figure S3C–E). These results suggested that Dox triggered senescence in HEI‐OC1 cells. GATA4 was downregulated in HEI‐OC1 to validate its role in protecting HCs against Dox‐induced senescence (Figure S3F,G). Post‐transfection with *GATA4* siRNA and subsequent treatment with Dox, as indicated by SA‐β‐gal staining, the percentage of senescence‐positive HCs decreased significantly following GATA4 downregulation (Figure S4A).

Flow cytometry analysis showed that Dox led to an increase in the S phase and the G2 phase (Figure S4B). However, when GATA4 was downregulated at the same time, the proportion of cells in the G1/S phase reverted (Figure S4B). EdU experiments showed that Dox significantly reduced the percentage of EdU‐positive cells, while siGata4 partially reversed this inhibitory effect, increasing the proportion of EdU‐positive cells in Dox‐treated cells (Figure S4C). We next examined the activity status of CDK2, a central kinase driving DNA replication and mitotic entry [34]. Western blot showed that Dox treatment significantly enhanced inhibitory Y15 phosphorylation and reduced activating T160 phosphorylation of CDK2, while GATA4 downregulation had the opposite effect (Figure S4D). Additionally, GATA4 downregulation suppressed cytoplasmic and mitochondrial ROS levels while rescued cell viability in senescence cells (Figure S4E–G). With reduction in the levels of senescence‐related proteins (Figure S4H), GATA4 downregulation halted the expression of several SASP factors during senescence (Figure S4I).

Furthermore, we demonstrated through SA‐β‐gal staining that ectopic expression of GATA4 induced senescence in HEI‐OC1 cells (Figure S3H,I, Figure S5A). GATA4 overexpression triggered diminished cellular viability (Figure S5B), S/G2 phase arrest and inhibited S phase (Figure S5C), impaired proliferative capacity (Figure S5D), functional inactivation of CDK2 (Figure S5E), and elevated cytoplasmic and mitochondrial ROS levels in the HEI‐OC1 cells (Figure S5F,G). Notably, transcriptional activation of GATA4 upregulated SASP components, which correlated with enhanced expression of senescence biomarkers (Figure S5H,I).

Previous studies have shown that autophagy suppresses GATA4 during cellular senescence [25, 26, 27, 35, 36]. We examined autophagy activity in vestibular tissues from MD patients and found that autophagy was markedly downregulated compared with controls, as evidenced by reduced LC3B‐II/LC3B‐I ratio and elevated p62 expression (Figure S5J). To investigate whether autophagy regulates the protein level of GATA4 in hair cells, we performed additional autophagy activation and blockade experiments. Activation of autophagy with rapamycin in Dox‐induced senescent HEI‐OC1 cells increased LC3B‐II and decreased p62 and GATA4 protein levels (Figure S5K). Inhibition of autophagy with 3‐methyladenine reduced LC3B‐II, elevated p62, and further increased GATA4 protein levels in senescent cells (Figure S5L). These results suggest that autophagy may contribute to GATA4 upregulation in hair cell senescence. Collectively, these data indicate that GATA4 deficiency mitigates LPS‐induced EH and audio‐vestibular dysfunction via attenuation of cellular senescence.

### 2.3 HDAC6 Physically Binds GATA4 and Restrains GATA4 Nuclear Translocation

**FIGURE 3:**
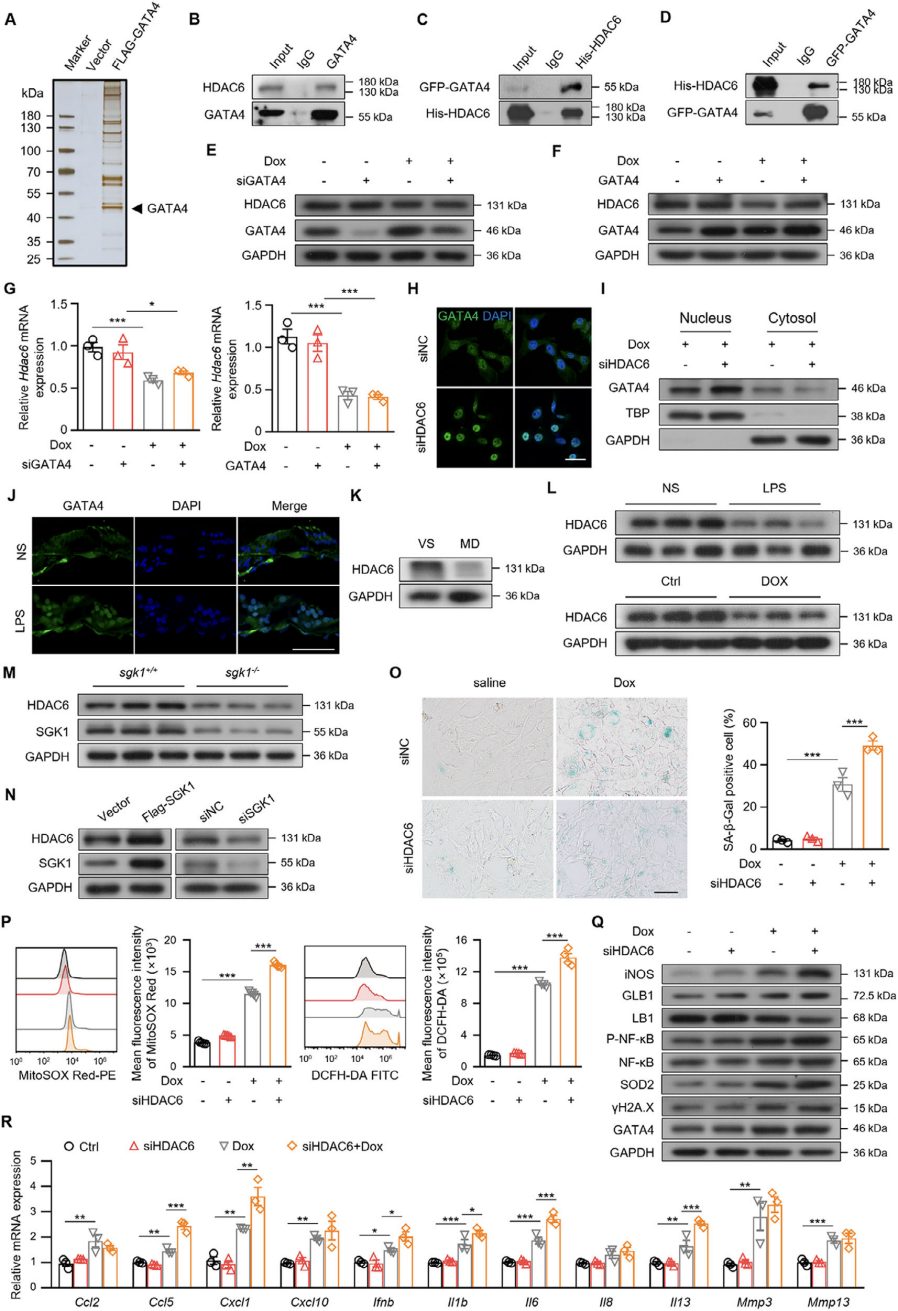
HDAC6 physically interacts with GATA4 and restrains GATA4 nuclear transport. (A) Immunoaffinity purification and mass spectrometry analysis of GATA4 containing protein complexes. Whole‐cell extracts from HEI‐OC1 cells stably expressing FLAG (Vector) or FLAG‐GATA4 were immunopurified using anti‐FLAG affinity columns and eluents with FLAG peptide. Eluates were resolved using SDS‐PAGE and silver‐stained. Protein bands were retrieved and analyzed using mass spectrometry. (B) Association of GATA4 with HDAC6 in HEI‐OC1 cells. IgG served as the negative control. (C,D) Association of GFP‐GATA4 with His‐HDAC6 in HEI‐OC1 cells. IgG served as the negative control. (E,F) Western blot analysis of HDAC6 and GATA4 protein expression in HEI‐OC1 cells. (G) The mRNA levels of HDAC6 in HEI‐OC1 cells (*n* = 3). (H) Representative confocal microscopy images of GATA4 (green) and DAPI (blue) staining in HEI‐OC1 cells transfected with HDAC6 siRNA (scale bar, 10 µm). (I) Western blot analysis detecting changes in GATA4 expression within the nucleus and cytoplasm of HEI‐OC1 cells. (J) Representative confocal microscopy images of GATA4 (green) and DAPI (blue) staining in hair cells of mice (scale bar, 50 µm). (K,L) Western blot analysis of HDAC6 expression in VEOs of VS and MD (K), cochleae of mice treated with LPS or saline, and HEI‐OC1 cells treated with/without Doxorubicin (L). (M) Western blot analysis of HDAC6 protein expression in the inner ear of *Sgk1^−/−^* ^.^mice. (N) Western blot analysis of HDAC6 protein expression in HEI‐OC1 cells following SGK1 overexpression or knockdown. (O) Representative images (left) and quantification (right) of SA‐β‐gal staining in HEI‐OC1 cells stimulated with HDAC6 siRNA‐transfected and Doxorubicin (0.5 µM, 24 h) (scale bar, 50 µm, *n* = 3). (P) Flow cytometry analysis and quantification of ROS levels in HEI‐OC1 cells using DCFH‐DA and Mito‐SOX Red (*n* = 4). (Q) Western blot analysis of senescence‐associated protein expression in HEI‐OC1 cells. (R) The mRNA levels of SASP in HEI‐OC1 cells (*n* = 3). Data are shown as mean ± SEM. Data were analyzed by one‐way ANOVA with LSD multiple comparisons posttests [(G), (O), (P), and (R)]. *p < 0.05, **p < 0.01, ***p < 0.001.

To investigate the molecular basis for the nuclear accumulation of GATA4 observed in MD patient tissues (Figure 1F), we utilized affinity purification‐mass spectrometry in HEI‐OC1 cells to identify interacting proteins. We introduced stably expressed FLAG‐tagged GATA4 (FLAG‐GATA4) into HEI‐OC1 cells. Silver staining revealed more specific protein bands in the FLAG‐GATA4 lane than those in the empty vector control (Figure 3A). The mass spectrometry details are shown in Data file S1. HDAC6 was identified as a protein closely associated with GATA4, which was further validated by co‐immunoprecipitation experiments (Figure 3B–D).

Notably, neither GATA4 depletion nor overexpression influenced HDAC6 protein levels in both normal and senescent HEI‐OC1 cells (Figure 3E–G), and HDAC6 depletion/overexpression did not affect GATA4 levels (Figure S6A–C). Immunofluorescence analysis and subcellular fractionation confirmed HDAC6 depletion increased GATA4 nuclear localization and decreased GATA4 cytoplasmic localization in HEI‐OC1 cells (Figure 3H,I). Consistent with human VEOs (Figure 1F), the hair cells from LPS mice showed a similar trend of elevated nuclear GATA4 relative to controls (Figure 3J). Notably, HDAC6 expression was decreased in VEOs of MD (Figure 3K, Figure S6D), as well as in EH mice and senescent HEI‐OC1 cells (Figure 3L, Figure S6E,F). To explore what might drive HDAC6 transcriptional downregulation, we investigated serum/glucocorticoid‐regulated kinase 1 (SGK1), a known positive regulator of HDAC6 in neurons [37]. Our previous work showed that SGK1 expression is downregulated in the VEOs of patients with MD [22]. HDAC6 expression was decreased in the inner ear of SGK1‐knockout mice (Figure 3M); in HEI‐OC1 cells, overexpression of SGK1 upregulated HDAC6 expression, whereas SGK1 knockdown downregulated HDAC6 levels (Figure 3N). These data suggest that reduced SGK1 activity may contribute to the downregulation of HDAC6 observed in MD and senescence, providing a plausible upstream initiating event in the pathway.

In vitro, we found that HDAC6 knockdown did not affect senescence phenotypes or HtrA1 levels in untreated HEI‐OC1 cells (Figure 3O–R). However, following Dox treatment, the percentage of senescence‐positive cells significantly increased in HDAC6‐siRNA HEI‐OC1 cells (Figure 3O). Flow cytometry analysis further revealed that Dox treatment increased both cytoplasmic and mitochondrial ROS levels, while HDAC6 knockdown reduced ROS levels (Figure 3P). Additionally, senescence‐related proteins and several SASP factors were elevated upon HDAC6 knockdown (Figure 3Q,R), indicating that HDAC6 knockdown exacerbates doxorubicin‐induced senescence in HEI‐OC1 cells, associated with the upregulation of HtrA1 and SASP factors. In vivo, we treated LPS‐challenged mice with the HDAC6 inhibitor ACY775 (Figure S7A), and observed that HDAC6 inhibition exacerbated cochlear EH in cochlear half‐turns II and IV (Figure S7B), worsened hearing loss (Figure S7C,D), and aggravated vestibular dysfunction (Figure S7E–G) in LPS‐challenged mice. Notably, GATA4 nuclear localization and NF‐κB activation were significantly increased in ACY775‐treated mice, while GATA4 levels remained unaffected (Figure S7H,I). These results corroborate our in vitro findings that HDAC6 inhibition worsens disease severity.

Collectively, these findings demonstrate that HDAC6 downregulation in MD promotes GATA4 nuclear translocation, thereby activating its transcriptional regulatory function and driving accelerated cellular senescence.

### 2.4 Identification of HtrA1 as a Key GATA4 Target

**FIGURE 4:**
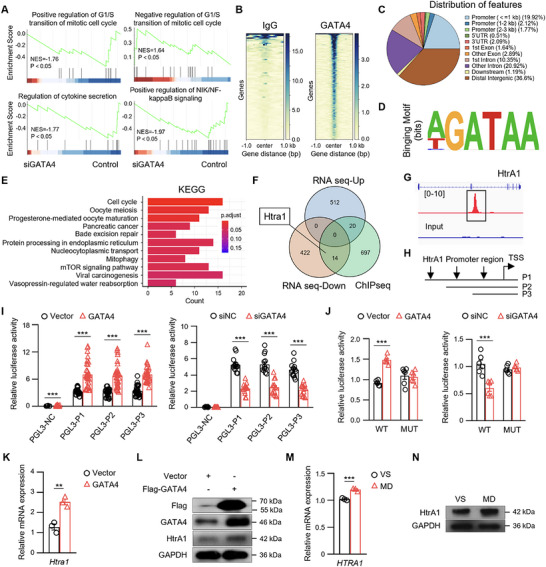
Genome‐wide transcriptional‐target analysis for GATA4. (A) GSEA showed that genes differentially expressed in response to GATA4 knockdown were enriched in gene sets significantly related to cell cycle, cytokine secretion and NF‐κB signaling. (B) ChIP‐seq density heatmaps and profiles of GATA4 binding regions. (C) Pie chart representing the genomic distribution of GATA4 targets based on ChIP‐seq analysis. (D) GATA4‐bound motifs analyzed using the MEME suite. (E) KEGG pathway analysis of functional group target genes regulated by GATA4. (F) Venn diagram of overlapping promoters bound by GATA4 in ChIP‐seq set and DEGs in RNA‐seq set. Numbers represent the number of promoters targeted by the indicated proteins. (G) Overview of the HtrA1 promoter region with ChIP‐seq data for GATA4 in HEI‐OC1 cells. (H) A schematic of the three HtrA1 promoter regions (P1–P3). (I) Dual luciferase reporter assay for the activity of the three HtrA1 promoter regions, pGL3‐HtrA1‐P1, P2, and P3 in HEI‐OC1 cells cotransfected with GATA4 plasmid (*n* = 34) or siGATA4 (*n* = 14). (J) Dual luciferase reporter assay for the activity of the WT or mutat promoter regions in HEI‐OC1 cells cotransfected with GATA4 plasmid or siGATA4 (*n* = 6). (K) The mRNA levels of HtrA1 in HEI‐OC1 cells following vector or GATA4 plasmid transfection (*n* = 3). (L) Western blot analysis of HtrA1 expression in HEI‐OC1 cells following vector or GATA4 plasmid transfection. (M) The mRNA levels of HtrA1 in VEOs of VS and MD (*n* = 3). (N) Western blot analysis of HtrA1 protein expression in VEOs of VS and MD. Data are shown as mean ± SEM. Data were analyzed by unpaired t test [(I), (J), (K), and (M)]. ** *p* < 0.01, *** *p* < 0.001.

To further delineate the molecular pathways that depend on GATA4, we performed high‐throughput RNA sequencing (RNA‐seq) analysis in HEI‐OC1 cells with GATA4 depletion. Compared to the control, siRNA knockdown of GATA4 resulted in significant dysregulation of 951 genes (fold change >1.5, p adj <0.05), including 429 downregulated and 522 upregulated transcripts (Figure S8A–C, Data file S2). Functional annotation using GSEA and KEGG pathway analysis revealed that differentially expressed genes (DEGs) were enriched in pathways critical to cellular senescence. Specifically, genes annotated to positive regulation of G1/S transition, regulation of cytokine secretion, and NIK/NF‐κB signaling were significantly downregulated, while genes annotated to negative regulation of G1/S transition were markedly upregulated (Figure 4A, Figure S8D and Data file S3). These results suggest that GATA4 is involved in modulating senescence‐related processes. Next, we selected 12 representative DEGs and confirmed their altered expression in siGATA4 cells using RT‐qPCR, validating upregulation of *Rarg, Ndrg1, Wbp2, Klhdc8b, Mrnip, Prs6ka1, Sfn* and downregulation of *Htra1, Adra1b, Ifit3, Ifit3b, Ppp6c* (Figure S8E).

To identify direct genomic targets of GATA4, chromatin immunoprecipitation sequencing (ChIP‐seq) was performed using GATA4‐specific antibodies. Analysis of ChIP‐seq signal distribution revealed that GATA4 predominantly binds within promoter regions (±3 kb of transcription start sites), with 741 out of 3,112 total binding peaks (p<0.001) located in promoter areas (Figure 4B, Data file S4). Moreover, within the ≤1 kb region, 620 binding peaks were identified, accounting for 19.92% and in the 1–2 and 2–3 kb regions, we identified 66 and 55 binding peaks, accounting for 2.12% and 1.77%, respectively (Figure 4C). Motif analysis using HOMER's findMotifsGenome.pl tool identified the canonical GATA‐binding motif (AGATAA) as the predominant sequence motif at GATA4‐bound loci (Figure 4D). Furthermore, the KEGG pathway analysis of GATA4‐bound genes highlighted cell cycle regulation as the most significantly enriched biological process (Figure 4E). Quantitative ChIP validation confirmed robust GATA4 occupancy at the promoters of candidate target genes, including *Chek2, Tcf3, Htra1, Wbp2, Wnk1, Birc5, Fzr1, Mapk11, Braf* and *Cdc6* (Figure S8F).

To identify key genes downstream of GATA4, we intersected DEGs from RNA‐seq with genes bound by GATA4 in ChIP‐seq datasets. HtrA1, a member of the serine peptidase family, plays a critical role in regulating programmed cell death [38]. Malik et al. found that HtrA1 mutations lead to conductive hearing loss [39]. More importantly, oxidative stress induces elevated expression of HtrA1, which promotes cellular senescence via the p38 MAPK pathway [40]. Among the 34 overlapping target genes (14 downregulated and 20 upregulated), HtrA1 emerged as a critical candidate due to its established roles in hearing loss and cellular senescence (Figure 4F). Here, we delineate the molecular mechanisms governing GATA4‐dependent regulation of HtrA1 and its functional consequences in cellular aging. To dissect GATA4's role in regulating HtrA1, we first validated GATA4 binding to the HtrA1 proximal promoter using Integrative Genomics Viewer analysis, demonstrating robust enrichment at specific genomic loci (Figure 4G).

Subsequent promoter activity assays utilized three luciferase reporter constructs spanning differing promoter regions (P1–P3) (Figure 4H). The results of luciferase reporter assays showed that ectopically expressed GATA4 enhanced the luciferase activity driven by all fragments, whereas GATA4 knockdown significantly reduced luciferase activity of these fragments (Figure 4I). These results confirmed that the P3 region of the HtrA1 promoter contained a GATA4‐responsive region. We then generated binding site mutant HtrA1‐Luc reporter in P3 region and found that site‐directed mutagenesis of potential GATA4 binding motifs failed to respond to GATA4 (Figure 4J). These findings pinpointed a functional GATA4‐responsive element within the P3 promoter region.

The results of qRT‐PCR and western blot confirmed that GATA4 depletion resulted in HtrA1 suppression in HEI‐OC1 cells (Figure S9A,B) and inner ear tissues (Figure S9C), while enforced GATA4 increased HtrA1 levels (Figure 4K,L), suggesting that GATA4 modulated HtrA1 expression at the transcriptional level. Western blot further showed that levels of the HtrA1 were partially but significantly elevated in VEOs of EH mice and MD patients (Figure 4M,N, Figure S9D,E).

### 2.5 HtrA1 Mediates GATA4‐Induced Audio‐Vestibular Dysfunction and Cellular Senescence

**FIGURE 5:**
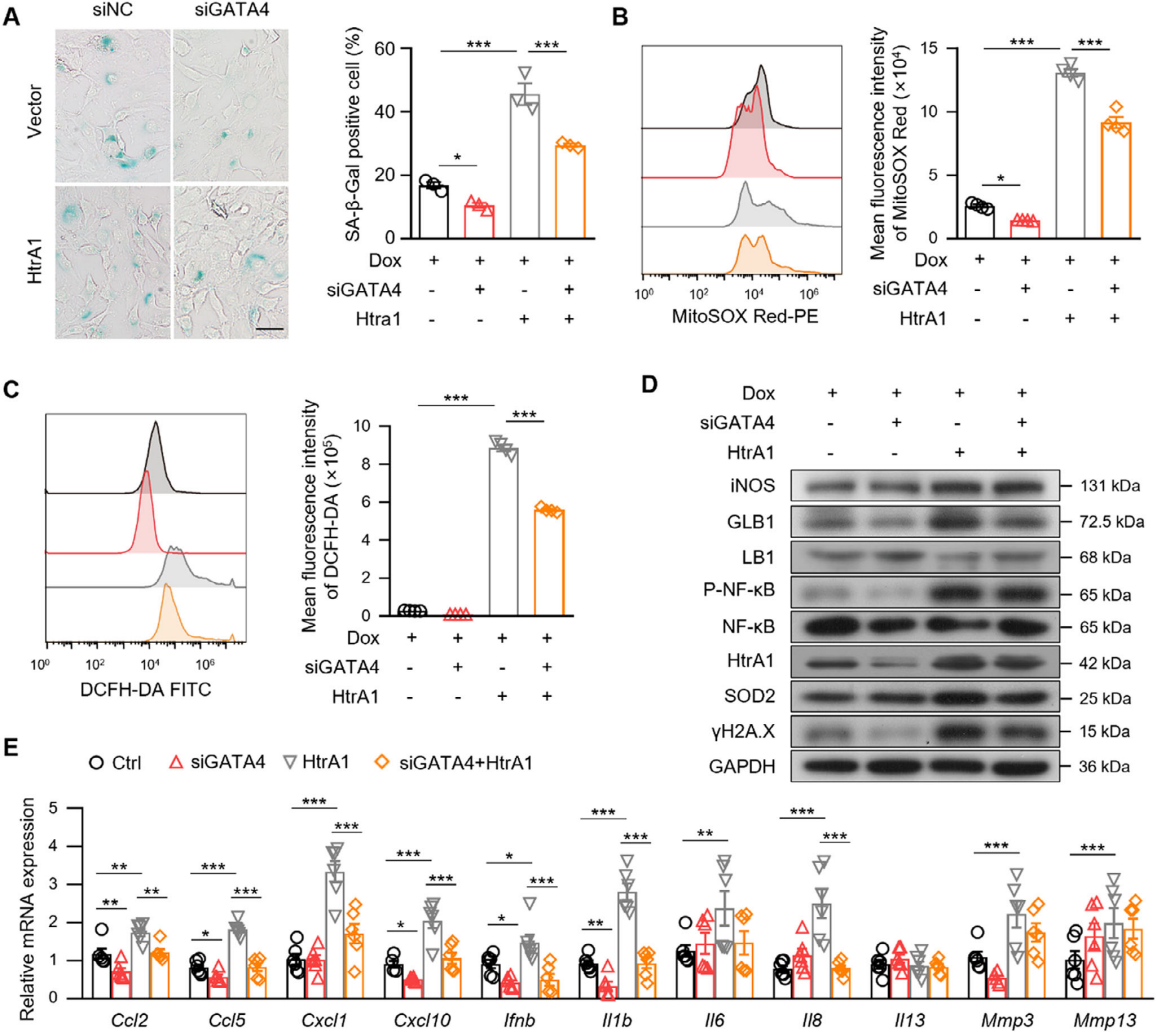
GATA4 promotes cellular senescence by activating HtrA1 expression. (A) Representative images (left) and quantification (right) of SA‐β‐gal staining in HEI‐OC1 cells stimulated with GATA4 siRNA‐transfected, HtrA1‐transfected, and Doxorubicin (0.5 µM, 24 h) (scale bar, 50 µm, *n* = 3). (B) Flow cytometry analysis (left) and quantification (right) of ROS levels in HEI‐OC1 cells using Mito‐SOX Red (*n* = 4). (C) Flow cytometry analysis (left) and quantification (right) of ROS levels in HEI‐OC1 cells using DCFH‐DA (*n* = 4). (D) Western blot analysis of senescence‐associated protein expression in HEI‐OC1 cells. (E) The mRNA levels of SASP in HEI‐OC1 cells (*n* = 6). Data are shown as mean ± SEM. Data were analyzed by one‐way ANOVA with LSD multiple comparisons posttests [(A), (B), (C), and (E)]. *p < 0.05, **p < 0.01, ***p < 0.001.

To investigate the regulatory role of HtrA1 in GATA4‐mediated senescence, we examined the effects of HtrA1 overexpression in GATA4‐depleted cells. SA‐β‐gal staining (Figure 5A) showed that GATA4 depletion reduced the proportion of senescent cells, whereas HtrA1 overexpression significantly increased SA‐β‐gal–positive cells in the siNC group, and this pro‐senescent effect was largely preserved even when HtrA1 was overexpressed in GATA4‐depleted cells. Analysis of ROS using MitoSOX and DCFH‐DA demonstrated that GATA4 depletion decreased mitochondrial and total ROS levels, while HtrA1 overexpression partially restored these signals (Figure 5B,C). In the Western blot analysis, GATA4 depletion reduced basal levels of iNOS, GLB1, p‐NF‐κB, SOD2 and γH2A.X, and increased levels of LB1 (Figure 5D). Upon HtrA1 overexpression, these proteins were restored (Figure 5D), confirming that HtrA1 can rescue the senescence phenotype suppressed by GATA4 depletion. Notably, although HtrA1 overexpression increased GLB1, p‐NF‐κB, SOD2 and γH2A.X in both siNC and siGATA4 cells, their levels remained slightly lower in the siGATA4 group despite comparable HtrA1 overexpression, indicating a partial reduction that mirrors the modest decrease in HtrA1 expression under GATA4 knockdown. In contrast, iNOS expression was fully restored by HtrA1 overexpression and reached comparable levels in both siNC and siGATA4 groups, suggesting that the iNOS related response is largely driven by HtrA1 and less dependent on GATA4. Moreover, qRT‐PCR showed that GATA4 depletion suppressed the transcription of multiple SASP factors (e.g., *Ccl2, Ccl5, Cxcl10, Il6*), and HtrA1 overexpression restored their transcription (Figure 5E). As shown in Figure S10, co‐depletion of HtrA1 significantly suppressed these pro‐senescent effects (e.g., increased SA‐β‐gal activity, elevated ROS levels, and enhanced SASP factor transcription, and senescence‐associated protein expression) induced by GATA4 overexpression (Figure S10A–E). Collectively, these data demonstrate that HtrA1 is required for GATA4‐driven senescence.

**FIGURE 6:**
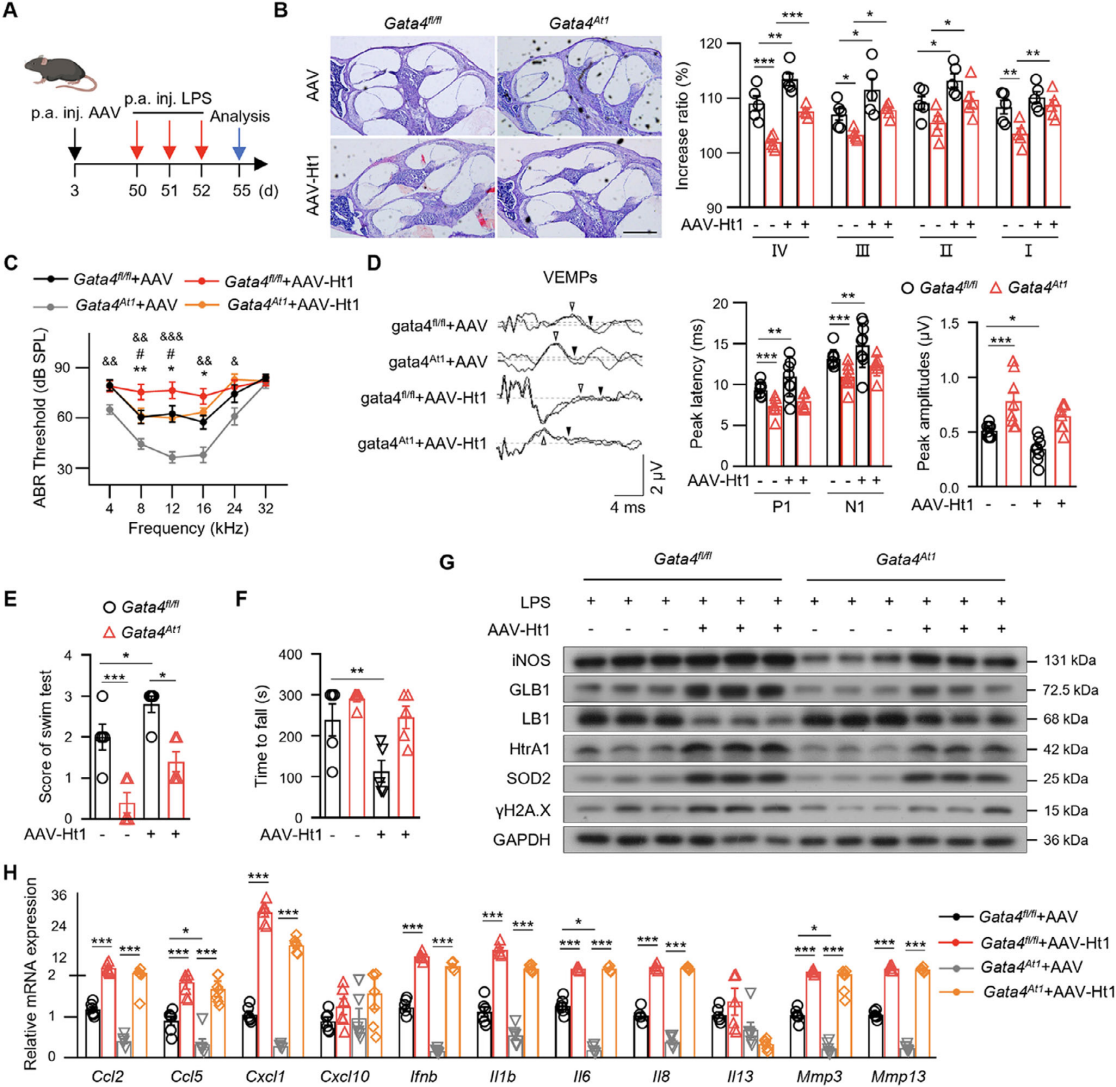
HtrA1 partially contributes to GATA4 function in audio‐vestibular dysfunction and cellular senescence in mice. (A) Schematic representation of the time scales for delivery of AAV vectors via posterior semicircular canal and analysis. The *Atoh1‐Cre* ^+/−^;*Gata4^fl/fl^* mice (referred to as *Gata4^At1^*) or *Atoh1‐Cre* ^+/+^;*Gata4^fl/fl^* mice (referred to as *Gata4^fl/fl^*) were treated with AAV or AAV‐HtrA1 (PSCC) at P3, treated with LPS (10 mg/kg, p.a.) for 3 consecutive days at P51, then analyzed at 55 days. (B) Representative images of mid‐modiolar cochlear sections, scale bar = 100 µm. Measurements of IR‐L in cochlear half‐turns I–IV (*n* = 5). (C) ABR thresholds in response to pure tone bursts across all frequencies (*n* = 10 or 7). * significant difference compared with *Gata4^fl/fl^+*AAV and *Gata4^At1^+*AAV; # significant difference compared with *Gata4^At1^+*AAV and *Gata4^At1^+*AAV‐Ht1; & significant difference compared with *Gata4^fl/fl^+*AAV and *Gata4^fl/fl^+*AAV‐Ht1. (D) Representative click‐evoked VEMP waves, the P1 (white triangle) and N1 (black triangle) peak latencies (*n* = 9), and P1‐N1 peak amplitudes of VEMPs at 100 dB nHL (*n* = 8). (E) Swimming posture scores of swim tests (*n* = 5). (F) Quantification of rotarod test (*n* = 5). (G) Western blot analysis of senescence‐associated protein expression in the inner ear. (H) The mRNA levels of SASP in the inner ear (*n* = 6). Data are shown as mean ± SEM. Data were analyzed by one‐way ANOVA with LSD multiple comparisons posttests [(B), (C), (D), (E), (F), and (H)]. * or # or & *p* < 0.05, ** or ## or && *p* < 0.01, *** or ### or &&& *p* < 0.001.

To verify whether the function of GATA4 is mediated through targeting HtrA1, we conducted in vivo overexpression studies using AAV2‐mediated HtrA1 (AAV‐Ht1) delivery into the mouse cochlea via the posterior semicircular canal at postnatal day 3 (Figure 6A). AAV2‐HtrA1 successfully targeted HCs and elevated HtrA1 transcriptional levels (Figure S11A,B). Following treatment with AAV2‐HtrA1, in both *Gata4^At1^* and *Gata4^fl/fl^* mice, HtrA1 overexpression exacerbated cochlear EH in cochlear half‐turns I‐IV (Figure 6B), worsened hearing loss (Figure 6C, Figure S11C), and aggravated vestibular dysfunction (Figure 6D–F), particularly in *Gata4^fl/fl^* mice. Furthermore, the expression of senescence markers and SASPs were significantly upregulated in AAV2‐HtrA1‐treated mice compared to AAV‐control groups (Figure 6G,H). These data indicate that overexpression of HtrA1 in mice deteriorates the disease severity in the LPS‐induced EH model, consistent with our observations in HEI‐OC1 cells.

To evaluate therapeutic potential, we selected Galegenimab (FHTR2163, RO7171009), a monoclonal antibody fragment targeting HtrA1's protease domain, which forms a trimeric complex to inhibit its activity [41, 42]. In LPS‐induced EH models, Galegenimab treatment had no effect on EH severity (Figure S12A,B), but restored ABR thresholds across 4–32 kHz (Figure S12C,D), shortened VEMP P1 latencies (Figure S12E), increased swim scores (Figure S12F), improved rotarod performance (Figure S12G), and enhanced VOR gain (Figure S12H). Galegenimab‐treated mice also regained weight and exhibited decreased senescence/SASP markers (Figure S12I–K).

HtrA1 levels were increased in senescent HEI‐OC1 cells following HDAC6 depletion or ACY775‐treated EH mice (Figure S13A–D). To verify whether HtrA1 acts as a downstream effector of HDAC6, we transfected HEI‐OC1 cells with HtrA1 siRNA after HDAC6 knockdown in Doxorubicin‐treated HEI‐OC1 cells. HtrA1 depletion reversed the senescence phenotype induced by HDAC6 depletion, significantly reducing SA‐β‐gal activity, senescence‐related proteins, SASP factor expression, and ROS levels (Figure S13E–I), directly demonstrating that HtrA1 depletion rescues HDAC6 depletion‐induced cellular senescence.

Collectively, these results suggest that inhibiting HtrA1 activity may provide therapeutic benefits for LPS‐associated inner ear inflammation and structural damage.

### 2.6 Impact of GATA4 Deletion on Age‐Related Audio‐Vestibular Dysfunction and Cellular Senescence

**FIGURE 7:**
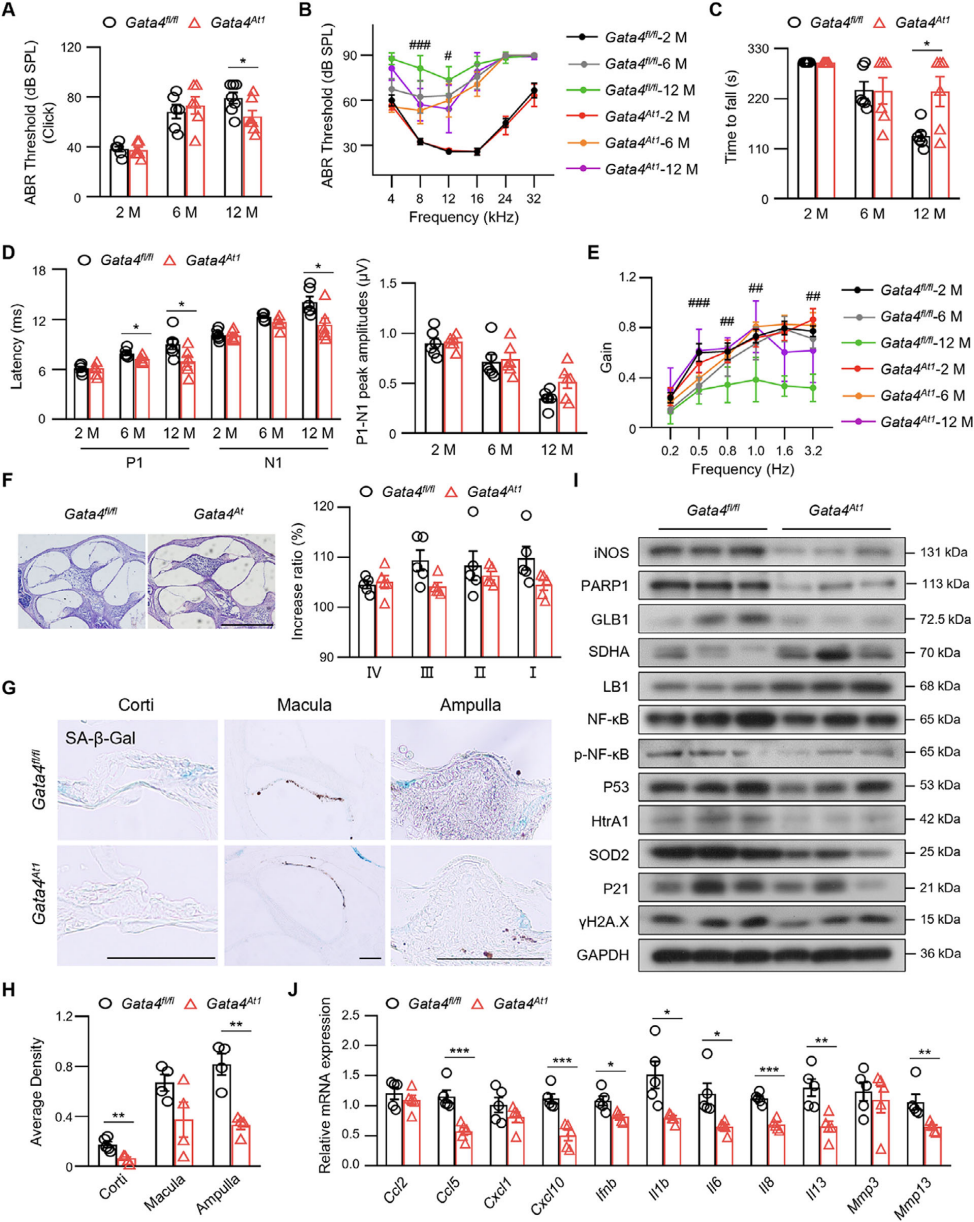
Genetic deletion of GATA4 in hair cells ameliorates audio‐vestibular symptoms and cellular senescence in aged mice. (A) ABR thresholds in response to click sounds (*n* = 6 or 7). (B) ABR thresholds in response to pure tone bursts across all frequencies (*n* = 6 or 7), # significant difference compared with *Gata4^fl/fl^*‐6m and *Gata4^At1^*‐6m. (C) Quantification of rotarod test (*n* = 6). (D) The P1 latencies, N1 peak latencies, and P1‐N1 peak amplitudes of VEMPs at 100 dB nHL (*n* = 6). (E) The VOR gains at a peak velocity (20°/s) and frequencies range from 0.2 to 3.2 Hz (n = 5 or 6), # significant difference compared with *Gata4^fl/fl^*‐6m and *Gata4^At1^‐6m*. (F) Representative images of mid‐modiolar cochlear sections, scale bar = 200 µm. Measurements of IR‐L in cochlear half‐turns I–IV (*n* = 5). (G) Representative images of SA‐β‐gal staining in the corti, macula, and ampulla (scale bar, 100 µm). (H) Quantification of SA‐β‐gal staining (*n*=4). (I) Western blot analysis of senescence‐associated protein expression in the inner ear. (J) The mRNA levels of SASP in the inner ear (*n* = 6). Data are shown as mean ± SEM. Data were analyzed by unpaired t test [(A), (B), (C), (D), (E), (F), (H), and (J)]. * *p* < 0.05, ** *p* < 0.01, *** *p* < 0.001.

The results of the above studies prompted us to explore whether a similar situation would occur in the aged mice. To address this, we defined three distinct time points for phenotypic detection of Gata4 KO mice, namely young (2 months old), middle‐aged (6 months old), and aged (12 months old). We have included both *Gata4^At1^* mice and their littermate controls at each time point, ensuring direct comparisons between age‐matched groups. The results revealed that aged *Gata4^At1^* mice exhibited significantly reduced thresholds in response to click and tone burst stimuli across the 8‐12 kHz frequency range (Figure 7A,B), prolonged rotarod latency (Figure 7C), shorter P1 and N1 latencies (Figure 7D), elevated gain of VOR (Figure 7E), compared to aged *Gata4^fl/fl^* controls no significant differences observed in 2‐month‐old and 6‐month‐old mice. Genetic deletion of GATA4 in hair cells did not affect the EH in the aged mice (Figure 7F). Notably, SA‐β‐gal staining revealed decreased SA‐β‐gal activity in the inner ear of aged *Gata4^At1^* mice (Figure 7G,H). Consistent with these observations, *Gata4^At1^* mice showed significantly lower expression of senescence markers, HtrA1 and SASP components relative to *Gata4^fl/fl^* littermates (Figure 7I,J). Collectively, these findings suggest that GATA4 deletion mitigates age‐associated audio‐vestibular deficits and cellular senescence in murine models of aging.

## 3 Discussion

Currently, due to the poorly defined pathophysiology, treatment for MD is designed to relieve symptoms [43], and there is an urgent need for more in‐depth research to elucidate its pathophysiological mechanisms and develop more effective therapeutic strategies. Here, we discovered the upregulation of GATA4 in the vestibular tissues of MD patients and in the EH mouse model. We have shown that GATA4 enhances the changes associated with cellular senescence in hair cells in vitro and in vivo. Genetic deletion of GATA4 in hair cells ameliorates LPS‐induced and age‐related audio‐vestibular symptoms and cellular senescence. HDAC6 interacts with GATA4 and restrains its nuclear transport. Through genome‐wide analysis, we identified HtrA1 as a key GATA4 target and found that GATA4 promotes cellular senescence by activating HtrA1 expression. Additionally, the efficacy of HtrA1 inhibition in EH mice highlighted the critical role of GATA4/HtrA1 in modulating cellular senescence, inner ear pathologies and representing promising therapeutic target for MD and age‐related inner ear disorders.

Recent studies have shown that the occurrence and development of MD are closely related to the immune response [23, 44]. A common feature of chronic age‐related diseases is low‐grade chronic systemic inflammation [45]. SASP, a hallmark of inflammaging, influences the microenvironment and cell‐cell communication [46]. Emerging evidence demonstrated that increased SASP (e.g., IL‐6, IL‐1β) is an important mechanism in MD, leading to a chronically inflamed microenvironment [44, 47], which is consist with our findings. The specific mechanisms of action of SASP in different stages of MD, and how to treat MD by regulating SASP remain unclear.

The GATA family consists of several proteins (GATA1‐6) with different variations of DNA‐binding domains composed of zinc finger structures [48]. Particularly, GATA4 is the member that is associated with aging [49], contributing to age‐related diseases such as atherosclerosis [50] and heart failure [51]. During embryonic development of the inner ear, cells need to precisely progress through the cell cycle to ensure proper proliferation, differentiation, and morphogenesis of hair cells and other inner ear cell types [52]. GATA3 is essential for stabilizing inner HC fate [53]. GATA2 and GATA3 redundantly function to maintain spiral ganglion cells and hearing [54]. In our study, we found that GATA4 is involved in regulating the G1/S transition of the mitotic cell cycle, which could have implications for inner ear development. Future studies could explore whether abnormal GATA4 expression during embryonic inner ear development affects proper progression through G2 (and the G1/S transition) and how this might contribute to congenital inner ear defects.

The study indicated that DNA damage contributed to GATA4 accumulation [25, 55]. We found that there was increased DNA damage in VEOs of MD. Notably, doxorubicin, a DNA damage‐inducing agent, facilitated the upregulation of GATA4 in HEI‐OC1 cells, which implied that DNA damage may be a reason for GATA4 upregulation in MD. Autophagy is a crucial cellular process, and mounting evidence suggests that aging is linked to a gradual deterioration in autophagic activity [56, 57]. GATA4 is degraded by p62‐mediated selective autophagy, but this regulation is suppressed during senescence, thereby stabilizing GATA4 and contributing to the accumulation of senescence‐associated markers [26, 58]. Autophagy levels decrease in the cochlear HCs of aged deaf mice; enhanced autophagy reduces HCs loss and improves hearing [59, 60]. Our study further showed reduced autophagy in the VEOs of MD patients, alongside GATA4 accumulation. Consistent with our in vitro data, pharmacological autophagy activation decreases GATA4 protein levels in senescent HEI‐OC1 cells, whereas inhibition increases them. In MD, impaired autophagy may thus serve as a complementary or upstream pathway that stabilizes GATA4 protein, operating alongside the HDAC6 mediated regulation of its nuclear translocation.

Senescence is typically associated with G0/G1 arrest, but stressors like severe DNA damage can induce a senescence‐like state with S/G2 accumulation in cells failing to complete these phases [61, 62]. Dox, a well‐characterized DNA‐damaging agent, induces senescence [63]; its initial 24–48 h effects involve DNA damage checkpoint activation to stall replication forks or induce S/G2 arrest for DNA repair, preceding stable G0/G1 arrest [64, 65]. Consistent with 0.5 µM Dox driving S‐phase progression and G2/M arrest [66], our flow cytometry showed elevated S/G2 populations at 24 h post‐Dox. GATA4 knockdown attenuated Dox‐induced S/G2 accumulation, while overexpression promoted S/G2 arrest—consistent with transcriptomic data showing GATA4 depletion triggers G1 arrest and inhibits S‐phase entry. These results support that GATA4 promotes senescent‐associated cell cycle arrest, matching Dox's early DNA damage response. Notably, flow cytometry revealed conflicting PI staining (S/G2 accumulation) and reduced EdU incorporation in Dox‐treated/GATA4‐overexpressing cells; CDK2 inactivation is the key mechanism, as Dox and GATA4 overexpression impair CDK2 function, blocking DNA replication and explaining reduced EdU incorporation in S/G2‐phase cells.

The enrichment of GATA4 binding peaks in the promoter regions of genes involved in cell cycle regulation and SASP suggests that GATA4 plays a central role in orchestrating the senescence‐associated transcriptional program. Previous studies demonstrate the critical role of HtrA1 in promoting cellular senescence and tissue degeneration [67, 68]. We found that GATA4 directly activates HtrA1 expression through binding to the AGATAA motif. Similar motifs have been reported where GATA4 regulates genes, which induce tumor progression and SASP [69, 70]. Given that GATA4 is known to activate NF‐κB to initiate the SASP [25], and HtrA1 has been reported to act upstream of the NF‐κB pathway [71, 72], we posit a positive feedback loop: GATA4‐driven transcriptional upregulation of HtrA1 may enhance or sustain NF‐κB activation, which in turn could further amplify the inflammatory and senescent phenotype. This synergistic interaction would collectively potentiate the SASP and inflammatory response, thereby accelerating disease progression. Elucidating the precise molecular interplay within this regulatory circuit represents a promising direction for future research.

Previous studies have emphasized that the nucleocytoplasmic shuttling of GATA4 is a crucial regulatory mechanism for its transcriptional activities. For instance, during obesity and hypertension, the nuclear translocation of GATA4 is dynamically regulated by ROS and impacts vascular smooth muscle cells remodeling [73]. In cardiomyocyte hypertrophy, abnormal nuclear translocation of GATA4 has been linked to cell size increase [74]. In the present study, we demonstrate that HDAC6 physically interacts with GATA4 without affecting expression levels, suggesting that HDAC6 may act as a molecular chaperone or anchor, sequestering GATA4 in the cytoplasm and preventing its nuclear translocation rather than regulating the synthesis or degradation of GATA4. When HDAC6 expression is downregulated, this inhibitory effect is alleviated, leading to significant nuclear accumulation of GATA4 and subsequent activation of HtrA1. This mechanism aligns with the observed enrichment of GATA4 in the nucleus of tissues from MD patients, highlighting the critical role of HDAC6 in modulating GATA4 transcriptional activity. HDAC6, a distinctive member of the class IIb histone deacetylase (HDAC) family, possesses two functional deacetylase domains and is primarily localized in the cytosol due to the presence of two nuclear export domains and a human ortholog‐specific sequence of an eight‐consecutive‐Ser‐Glu tetradecapeptide repeat domain [75, 76]. HDAC6 has been demonstrated to have a role in major cellular pathways, including microtubule transport, chaperone‐mediated stress response, oxidative stress, autophagy and lipophagy, inflammasome response, aggresome formation, and neurite outgrowth [77]. The HDAC6‐mediated regulation of GATA4 nuclear translocation may represent a key molecular mechanism underlying accelerated cellular senescence during MD development, offering novel theoretical insights for future exploration of disease pathogenesis.

While our study provides novel insights into GATA4's role in inflammaging‐mediated Meniere's disease, several limitations should be acknowledged. First, our analysis predominantly concentrated on hair cells, neglecting other key inner ear cell types, such as supporting cells, spiral ganglion neurons, and macrophages, which maintain inner ear function [78]. Inner ear pathologies often involve complex interactions between different cell types. For instance, abnormal stria morphology and function can lead to hair cell death [79]. Future studies should explore GATA4's role in these additional cell types. Additionally, reliance on animal models and cell lines may not fully capture human inner ear complexity, necessitating validation in human tissues and clinical settings. Longitudinal studies in animal models to track GATA4 expression changes over time could clarify its relationship with age‐related lesions. Lastly, potential compensatory mechanisms and interactions with other transcription factors in GATA4‐deficient conditions remain unexplored, warranting further investigation into functional redundancy and network dynamics.

In summary, our study identifies GATA4 as a critical regulator of hair cell senescence and inner ear inflammation, linking its upregulation to the progression of Meniere's disease and age‐related hearing and balance impairments. We propose that under environmental stressors such as stress, antigen exposure, or other stimuli, the reduction in HDAC6 expression in hair cells allows highly expressed GATA4 to translocate into the nucleus. There, GATA4 activates the transcription of HtrA1, promoting hair cell aging and triggering the upregulation of SASP genes, which exacerbate inner ear inflammation and endolymphatic hydrops, ultimately contributing to disease development. Future efforts should prioritize translational validation, including biomarker‐driven clinical trials and gene‐editing strategies to modulate GATA4 activity in inner ear diseases.

## 4 Experimental Section/Methods

### 4.1 Study Design

The objective of the study is to define whether there is cellular senescence in the vestibular tissue of MD patients, whether GATA4 is upregulated in the vestibular tissue of MD patients, EH mouse models, aged animal models and senescent cells, and to determine the effect and specific mechanism of GATA4 in regulating cellular senescence and inflammaging in inner ear pathologies, providing promising therapeutic targets for MD and age‐related audio‐vestibular disorders. The study objectives were achieved through the following methods: (i) clarifying the cellular senescence in the vestibular tissues of MD patients and the cochlea of elderly mice, as well as the expression of GATA4 in the vestibular tissues of MD patients, the cochlea of EH mouse models, the cochlea of elderly mice, and the senescent cells induced by doxorubicin; (ii) exploring the role of HDAC6 in the nuclear localization of GATA4; (iii) analyzing the role and mechanism of GATA4 transcriptional regulation of HtrA1 through RNA‐seq and ChIP‐seq analysis; (iv) evaluating the therapeutic value of the GATA4/HtrA1 axis in EH mice and aged mice. Human vestibular tissues (utricles, saccules, and ampullae) and clinical data were obtained from Shandong Provincial ENT Hospital under protocols approved by the institutional Ethics Committee (IRB XYK‐20220817). Written informed consent was obtained prior to the surgical procedure and the collection of VEOs and clinical information for research. For in vitro coculture experiments, at least three biological replicates for each group were performed. In all in vivo experiments, no animals were excluded from the analysis. All control and treated mice in each experiment were sex and age matched. For all treatments, mice were randomized with cages. All pathology analyses were performed blindly by a pathologist. The ethical approval for animal experiments was obtained from Shandong Provincial ENT Hospital under protocols approved by the institutional Ethics Committee (IRB XYK‐20220818). Detailed information regarding the number of replicates, the statistical test used, and the corresponding *p* values are provided in the figure legends.

### 4.2 Human Studies

The study was carried out in accordance with the principles of the Declaration of Helsinki (2013 revision) for investigations involving humans. Tissues were acquired from patients with MD (n = 8) undergoing labyrinthectomy and from patients with VS (n = 8) undergoing translabyrinthine surgery (as a control group), in close collaboration with doctors to minimize tissue disruption. The demographic and clinical characteristics of the MD patients are shown in Table S1.

### 4.3 Animal Studies and Treatments

All protocols were approved by the Animal Care Committee of Shandong University and conformed with the Guidelines for the Care and Use of Laboratory Animals of the National Institutes of Health. All the mice were housed in a temperature‐controlled (20–22°C) room, with a 12/12 h light/dark cycle and had free access to food and water.

Wild type C57BL/6 mice were purchased from the Animal Center of Shandong University (Jinan, China). Atoh1‐Cre mice (Stock No. 011104) on a C57BL/6J background were obtained from the Jackson Laboratory. *Gata4^fl/fl^* (MJ1010‐272) mice on a C57BL/6J background were purchased from Cyagen (China). The *Atoh1‐Cre^+/−^;Gata4^fl/fl^* (*Gata4^At1^*) mice were generated by crossing *Atoh1‐Cre^+/−^* mice with *Gata4^fl/fl^* mice. *Sgk1* knocked out (*sgk1^−/−^*) mice were purchased from Cyagen Biosciences.

C57BL/6 mice with LPS‐induced EH were used as models for MD. To establish the EH mouse model, mice were challenged with LPS (2 mg/kg, Sigma‐Aldrich, St. Louis, MO, USA) dissolved in saline through postauricular (p.a.) injection once a day for 3 days. The control groups received an equivalent amount of 0.9% physiological saline. To evaluate the effect of the HtrA1 inhibition, LPS‐challenged mice were treated with HtrA1 inhibitor Galegenimab (6 mg/kg, i.p., MCE, NJ, USA) via intraperitoneal injection (i.p.) for 7 consecutive days.

### 4.4 Cell Lines, Cell Culture, and Treatments

The contamination‐free HEI‐OC1 cells (RRID:CVCL_D899, Table S2) were cultured in high‐glucose Dulbecco's modified Eagle medium (DMEM, Gibco, Grand Island, NE, USA) with 10% fetal bovine serum (FBS, Gibco) at 33°C in a humidified incubator containing 10% CO_2_. In the experiments, HEI‐OC1 cells were stimulated with Doxorubicin (MCE) for 24 h. Analyses were then performed at 48 h after the Doxorubicin stimulation. For autophagy activation and blockade, HEI‐OC1 cells were incubated with rapamycin (50 nM) or 3‐methyladenine (1 mM) for 48 h.

All experiments were performed with mycoplasma‐free cells. siRNAs and plasmids were transfected using Lipofectamine RNAiMAX Reagent (Thermo Fisher Scientific, Cleveland, OH, USA) or PEIpro (Polyplus, Strasbourg, France) per the manufacturer's instructions. GATA4 siRNA was constructed by GenePharma (Shanghai, China), GATA4‐GFP and HtrA1‐GFP plasmids were constructed by Genechem (Shanghai, China). The firefly luciferase reporter plasmid, pGL3‐HtrA1‐luciferase, and Renilla plasmids were synthesized by Guangzhou Ribobio. All siRNA/shRNA sequences are listed in Table S3.

### 4.5 AAV Construction and Injection in Mice

The HtrA1 overexpression plasmid was designed by Genechem (Shanghai, China). AAV vector AAV2/Anc80L65 is reported to efficiently infect inner ear cells [80]. The AAV‐HtrA1 (AAV‐Ht1) tagged with the fluorescent protein mNeonGreen was constructed by OBIO Technology (Shanghai, China). Viral injections through the posterior semicircular canal (PSCC) were performed as outlined in prior studies [81]. Briefly, following anesthesia by cooling on ice, an incision was made behind the left neck skin, the muscles were separated, and the PSCC was exposed in the neonatal mice. The AAVs were injected into the cochlea via the PSCC, and the volume was controlled at 1.5 µL by the UMP3 UltraMicro Pump (World Precision Instrument). After the AAV injection, the wound was sealed using a Dermafuse tissue adhesive (Millpledge Ltd., UK).

### 4.6 CCK‐8 Assay

HEI‐OC1 cells (passage 12–15) were seeded at 5 ×10^3^ cells/well in 96‐well plates and allowed to adhere for 24 h. HEI‐OC1 cells were treated with 0.1, 0.5, and 1 µM doxorubicin hydrochloride (MCE) for 24 h. Cell viability was quantified using Cell Counting Kit‐8 (CCK‐8, Protein Biotechnology, Portland, OR, USA) according to the manufacturer's protocol. Briefly, 10 µL CCK‐8 reagent was aseptically added to each well, followed by 60 min incubation protected from light. The absorbance of the medium was measured at 450 nm using a microtiter plate reader (BIO‐RAD, CA, USA), with blank control subtraction performed prior to analysis.

### 4.7 The Intracellular ROS Determination

The generation of ROS was measured using a MitoSOX Red mitochondrial superoxide indicator (MitoSOX‐Red, Life Technologies, Carlsbad, CA, USA) and 2’,7’‐Dichlorofluorescin diacetate (DCFH‐DA, Solarbio, Beijing, China). Cells were harvested and washed with serum‐free medium and incubated with DCFH‐DA (5 µM) for 10 min and DCFH‐DA (10 µM) for 30 min, and then analyzed with a flow cytometer (BD Biosciences, Franklin Lakes, NJ, USA).

### 4.8 Transmission Electron Microscopy (TEM)

The human vestibular tissue was harvested, immediately fixed using a 3% glutaraldehyde fixative solution and 1% osmic acid, dehydrated, infiltrated, and embedded in Epon 812. Subsequently, ultrathin radial sections were stained with lead citrate and uranyl acetate before being examined under a transmission electron microscope (JEOL, Tokyo, Japan).

### 4.9 Cell Cycle Analysis

Cell cycle analysis was performed using a Cell Cycle and Apoptosis Analysis Kit (BioScience). Briefly, cells were trypsinized and fixed overnight in 70% ethanol at 20°C. Next, the cells were stained with propidium iodide and subjected to cell cycle analysis using a cell analyzer (BD LSRFortessa, BD Biosciences). Data were collected and analyzed using ModFit LT software (Verity Software House, ME, USA).

### 4.10 Proliferation Assay

Proliferation of HEI‐OC1 cells was measured using the Click‐iTTM EdU Cell Proliferation Kit for Imaging (Invitrogen, Carlsbad, CA, USA), which was performed according to the manufacturer's instructions. For 5‐Ethynyl‐2′‐deoxyuridine (EdU) detection, HEI‐OC1cells infected with indicated lentivirus were seeded in a 12‐well plate (1 × 105 cells/well) containing slides and incubated for 24 h. In the last 2 h, EdU solution was added to the culture medium, and EdU incorporation was detected using a Leica SP8 inverted confocal microscope.

### 4.11 RNA Preparation and Sequencing

After transfection with siGata4, the total RNA of HEI‐OC1 cells was extracted and subjected to RNA‐seq. Briefly, approximately 5 µg of cDNA was generated from total RNA by using Smart‐seq2. The resulting cDNA was measured with a Qubit 3.0 Fluorometer (Thermo Fisher Scientific), fragmented by using sonication (Bioruptor Sonication System; Diagenode, Inc., Denville, NJ, USA), profiled by using an Agilent 2100 High Sensitivity DNA Assay Kit (Agilent Technologies, Santa Clara, CA, USA), and subjected to Illumina library preparation by using the SPRIWorks HT kit (Beckman Coulter, Inc., Brea, CA, USA). The quality, quantity, and size distribution of the Illumina library were determined by using an Agilent 2100 Bioanalyzer (Agilent Technologies). The library was subjected to sequencing according to standard procedures (HiSeq 2500; Illumina, San Diego, CA, USA). Paired‐end 10^6^‐nucleotide (nt) reads were generated and evaluated for data quality by using FASTQC software (Babraham Institute, Cambridge, UK).

### 4.12 Bioinformatics Analysis

Differential expression tests were performed by using the DESeq R package (R Foundation for Statistical Computing, Vienna, Austria) according to manufacturer instructions. The main outcome measure of the study was differentially expressed genes (DEGs). The protein functions of the identified DEGs were analyzed by using gene ontology (GO) (InterProScan, version 5.14‐53.0) terms in the cellular component, molecular function, and biological process categories. The Kyoto Encyclopedia of Genes and Genomes (KEGG) (KAAS, version 2.0; KEGG Mapper, version 2.5) database was used to annotate protein pathways. For the functional enrichment of GO and pathway analysis, a two‐tailed Fisher's exact test was used, and a corrected *p*‐value <0.05 was considered statistically significant. Enrichment‐based clustering was visualized by using the R package, pheatmap (version 2.03).

### 4.13 Quantitative Chromatin Immunoprecipitation (qChIP), and ChIP Sequencing (ChIP‐seq) Assays

ChIP and qChIP assays were performed using HEI‐OC1 cells. Briefly, 1 × 107 cells were crosslinked with 1 % formaldehyde, sonicated, precleared, and incubated with 2 µg primary antibody specific to normal mouse IgG (control) or GATA4 (Table S4). The complex was washed with low‐salt and high‐salt buffers, and the DNA was extracted for qChIP and ChIP‐seq assays. For ChIP‐seq, 10 ng DNA was resolved on an Agilent Technologies 2100 Bioanalyzer, and 50–250 bp fractions were extracted, which were then subjected to end‐repair and 3ʹ‐adenylation. Adapter‐ligated libraries were amplified, purified, and selected using an Agencourt AMPure XP‐Medium kit, and the final library was composed of single‐stranded cDNA. In‐depth whole‐genome DNA sequencing was outsourced to Xiuyue Biol (Jinan, China). Sequencing data acquired from the Illumina analysis pipeline were compared with the unmasked human reference genome hg38 (UCSC GRCh38) using ELAND (Illumina, San Diego, CA, USA). The peaks were identified using Model‐based Analysis of ChIP‐Seq after filtering through the input. ChIP seeker was used to analyze the genomic distribution of GATA4 binding sites.

### 4.14 Luciferase Reporter Assay

The activity of the HtrA1 promoter was determined using a dual‐luciferase reporter assay system, according to the manufacturer's instructions (Promega, Madison, WI, USA). The three promoter regions (P1‐P3) of the HtrA1 gene were cloned into the pGL3‐basic vector by Guangzhou Ribobio. P1: − 1750–100, P2: −1300–100, P3: −650–100. The HtrA1 transcription factor binding sites were mutated based on a consensus nucleotide sequence in the HtrA1 P3 promoter. The resulting constructs were pGL3‐ HtrA1‐P3‐mut.

### 4.15 Immunofluorescence Staining

Human vestibular tissue and mouse inner ears were fixed overnight at 4°C in 4% paraformaldehyde solution and decalcified with 0.5 M EDTA for 24 h. Samples were gradient dehydrated in sucrose, embedded in OCT, and sectioned at 5 µm thickness. For antigen retrieval, slides were incubated in antigen retrieval solution (Beyotime, Shanghai, China) for 30 min at 99°C. After cooling down, slides were permeabilized in 1% Triton X‐100 and blocked for 1 h using 5% donkey serum and 1% BSA diluted in PBS. The samples were then incubated with different primary antibodies at 4°C overnight. After triple rinses with PBS, sections were treated with secondary antibodies (1:1000, Thermo Fisher Scientific) and DAPI (1:1000, Sigma‐Aldrich) diluted in PBS with 1% BSA for 2 h. All imaging was performed with a Leica SP8 inverted confocal microscope.

### 4.16 SA‐β‐Gal Staining and Quantitative Evaluation

SA‐β‐gal staining was performed using a Senescence‐β Galactosidase Staining Kit (Beyotime) according to the manufacturer's protocol. After transfection, the cells were washed once with PBS, fixed, and stained with the reagents in the kit. The percentage of positively stained cells was assessed using a Leica SP8 inverted confocal microscope.

All quantification in the study was performed in a blinded fashion. Images were analyzed using ImageJ v1.51. The eight‐bit blue channel image was corrected for background illumination using the eight‐bit blue channel of a bright‐field image. A threshold was then applied to the image to measure the total expression. The Average density of the fields was used to indicate the Average Density.

### 4.17 Dihydroethidium Fluorescence

Intracellular superoxide levels were quantified using Dihydroethidium (DHE), an oxidative fluorescent dye (Distinct mitochondrial retrograde signals control the G1‐S cell cycle checkpoint). Fresh unfixed VEOs were embedded in OCT compound and rapidly frozen in liquid nitrogen‐cooled isopentane, and transverse sections (10 µm) were obtained using a cryostat. Sections were then incubated in a light‐protected chamber at room temperature for 30 min with 10 µmol/L DHE (Molecular Probes). Images were obtained with a Leica SP8 inverted confocal microscope.

### 4.18 RNA Extraction and qRT‐PCR

The total RNA of human vestibular tissue, mice inner ears, and HEI‐OC1 cells was extracted using TRIzol reagent following the manufacturer's protocols (Invitrogen). For reverse transcription, 1 mg of RNA was processed using high capacity DNA reverse transcription kit (Thermo Fisher Scientific). The relative expression levels of mRNA were performed using Power SYBR green PCR master mix (TaKaRa, Osaka, Japan) and quantified on an Eppendorf PCR machine (Hamburg, Germany), and normalized against housekeeping genes β‐Actin. The primers sequences were provided in Table S2.

### 4.19 Immunopurification and Mass Spectrometry

To generate a stable cell line expressing FLAG‐GATA4, HEI‐OC1 cells were transfected with FLAG‐tagged GATA4 for 48 h. Anti‐FLAG immunoaffinity columns were prepared using an anti‐FLAG M2 affinity gel (Sigma‐Aldrich) following the manufacturer's protocol. Subsequently, a 0.2 mg/mL FLAG peptide (Sigma‐Aldrich) was used to elute the FLAG protein complex from the columns. Eluted fractions were collected, separated by SDS‐polyacrylamide gel electrophoresis, silver‐stained, and analyzed by liquid chromatography‐tandem mass spectrometry for protein identification and data analysis.

### 4.20 Immunoprecipitation (IP) and Western Blotting

For IP assays, cells were washed twice with cold PBS, and extracts prepared by incubating cells in lysis buffer (containing 50 mM Tris–HCl, pH 7.4, 150 mM NaCl, 1 mM EDTA, 0.5% NP‐40, 0.25% sodium deoxycholate, and a protease inhibitor cocktail) at 4°C for 30 min, followed by centrifuging at 12 000×*g* for 10 min. Protein concentrations were detected using the BCA Protein Assay Kit (Beyotime). Next, 500 µg of cellular extract were incubated with appropriate primary antibodies or normal rabbit/mouse IgG at 4°C overnight with continuous rotation. Then, glutathione‐sepharose beads were added, and the mixture was incubated at 4°C for 2 h. After washing the beads four times with cell lysis buffer, the captured immune complexes were obtained.

The total protein was extracted with cold RIPA lysis buffer (Beyotime) supplemented with protease inhibitor cocktail (Sigma‐Aldrich). For cytoplasmic and nuclear fractionation assays, HEI‐OC1 cells were harvested and separated using a Nuclear and Cytoplasmic Protein Extraction Kit (Beyotime) following the manufacturer's instructions. Equal amounts of protein were denatured at 99°C for 10 min. The captured immune complexes and protein were separated using 10% SDS‐PAGE gel electrophoresis. The proteins were transferred to polyvinylidene difluoride membranes (Millipore, MA, USA), blocked in 5% skim milk, incubated with primary antibodies followed by immunoblotting with HRP‐conjugated secondary antibodies (1:10 000). The protein signals were detected using an ECL kit (Epizyme). All blocking, incubation, and washing procedures were conducted using TBST solution (Solarbio).

### 4.21 Measurement of SASP in Cochlear Tissue

The protein levels of SASP factors (CCL2, CCL5, CXCL1, IL‐1β, and IL‐6) in mouse cochlear homogenates were measured using a multiplex immunoassay. Cochlear tissues were homogenized in cold PBS, and the supernatants were collected after centrifugation. Cytokine/chemokine concentrations were determined using the ABplex Mouse 5‐Plex Custom Panel according to the manufacturer's instructions. Data were acquired on an ABplex‐100 detection system, and analyte concentrations were calculated from standard curves. Final values were normalized to the total protein concentration of each sample.

### 4.22 Auditory Brainstem Response (ABR)

ABR was used to assess the auditory function of mice, utilizing tone pip at different frequencies (4, 8, 12, 16, 24, and 32 kHz), with 1,024 stimulus repetitions per record in a sound isolation booth using a TDT RZ6 auditory physiology workstation (Tucker‐Davis Technologies, FL, USA). Mice were anesthetized through intraperitoneal injections of xylazine (10 mg/kg) and ketamine (100 mg/kg). Needle electrodes were placed subcutaneously at the vertex (recording electrode), the infra‐auricular mastoid region of ipsilateral ear (reference electrode), and the back (ground electrode). The sound levels commenced at 90‐dB sound pressure level (SPL) and decreased in 5‐dB increments to the acoustic thresholds for each frequency. The ABR threshold was identified as the minimal SPL resulting in a reliable ABR recording with distinguishable waves upon visual inspection. The process was repeated around the threshold to ensure the waveform consistency.

### 4.23 Vestibular Evoked Myogenic Potential (VEMP)

Click‐evoked VEMP recordings were initiated with simultaneous recording of electromyography (EMG) potentials post‐anesthesia. Mice were positioned prone, and the neck was hyperextended and stabilized by wire suspension behind the front teeth. Needle electrodes were inserted into the neck extensors (recording electrode), cervico‐occipital region at the midline (reference electrode), and the back (ground electrode). Each mouse underwent VEMP testing using a stimulus intensity of 100 dB nHL, with repeatability verified by consecutive runs (>3 times). The latencies of the positive and negative peaks were subsequently measured.

### 4.24 Vestibular Ocular Reflexes (VOR)

Mice were immobilized using a noninvasive setup, and an infrared camera was positioned at a 45° angle relative to the mouse's anteroposterior axis, with illumination provided by two near‐infrared LED lights. Eye tracking was conducted using a template‐matching method at 60 frames/s, focusing on the pupil region. Ellipse fitting determined the pupil center, facilitating extraction of horizontal eye movements. Rotational stimuli were delivered at 20°/s across various frequencies. VOR gain was calculated as the ratio between the response amplitude and stimulus amplitude after Fourier transformation of the eye‐position data using MATLAB 2016b.

### 4.25 Rotarod Test

Mice were placed on a motorized rotating rod (Anhui Zhenghua Biologic Apparatus Facilities) rotating at a maximum speed of 30 rpm, which gradually accelerated to this speed over 5 min. Each animal underwent training sessions twice daily for 3 days, followed by two test trials. The average time before falling off the rod was recorded for analysis.

### 4.26 Swim Test

General vestibular function could be scored using swimming tests [82]. Mice were placed in a standard pool with a water level height of approximately 15 cm and a water temperature of around 25°C. Mice were scored for swimming posture, with mice swimming in the water (score 0), swimming irregularly (score 1), floating stationarily (score 2), or rolling underwater (score 3).

### 4.27 Quantitative Assessment of Changes in Endolymphatic Space in Cochlea

Frozen sections of mouse cochleae were stained with hematoxylin (Solarbio) for 3 min and then with eosin (Beyotime) for 2 min. The sections were then dehydrated using gradient ethanol and sealed with neutral gum. To quantitatively evaluate the changes in the endolymphatic space, the length of the extended Reissner's membrane (L) and its ideal length (L*) were measured in each cochlea using ImageJ. The increased ratios of the length change in Reissner's membrane (IR‐L (%) = L/L* × 100) were calculated to assess the EH levels for each turn. The values were determined using ImageJ.

### 4.28 Quantitative Evaluation of Mitochondria

The number of mitochondria per hair cell was quantified in three subjects per group, who were imaged at 15 000× magnification in a blinded fashion. The mitochondrial count was determined via the point‐counting method with ImageJ v1.51. Healthy mitochondria were defined by oval or spherical morphology, well‐demarcated cristae, intact membrane integrity, and dense matrix.

### 4.29 Statistical Analysis

No statistical methods were used to predetermine sample sizes. No data were excluded. All the experiments were replicated at least twice. Animals were randomly distributed in groups where different treatments were used (using a simple randomization method). The investigators were blinded to the exact sample information during sample collection and analysis. Statistical analysis was performed using SPSS 13.0 software (SPSS Inc., USA). For experiments with a small sample size that failed to pass the normal distribution test, a nonparametric t‐test or one‐way ANOVA was used. All results are shown as the mean ± standard error of the mean (SEM). unless stated otherwise. Data distribution was assumed to be normal, but this was not formally tested. Comparisons between two groups were made by an unpaired two‐tailed Student's t‐test. Multiple comparisons of one‐variable data were carried out by one‐way analysis of variance (ANOVA) followed by LSD multiple comparisons posttests. For consistency in comparisons, significance in all figures is denoted as follows: * or # or & *p* < 0.05, ** or ## or && *p* < 0.01, *** or ### or &&& *p* < 0.001. For all representative findings, triplicate or multiple independent experiments were performed, and similar results were obtained.

## Author Contributions

Conceptualization: N.Z., N.L., D.Z., and H.W. Methodology: N.Z., Y.W., J.Z., J.L., and Y.S. Sources: Y.H., Y.L., X.L., J.W., Y.L., and Z.F. Investigation: N.Z., N.L., Y.W., J.Z., and H.W. Visualization: N.Z., Y.W., and J.Z. Funding acquisition: N.Z., N.L., L.C, Y.M., D.Z., and H.W. Project administration: N.L., D.Z., and H.W. Supervision: D.Z. and H.W. Writing – original draft: N.Z. and N.L.; Writing – review & editing: N.Z., N.L., D.Z., and H.W.

## Funding

This work was supported by the National Natural Science Foundation of China (No. 82401358, No. 82371154, No. 82271172, No. 82171150, No. 82471175, No. 82401364), China Postdoctoral Science Foundation (No. 2024M761821), Natural Science Foundation of Shandong Province (No. ZR2024QH421, No. ZR2024MH027, ZR2024QH182, No. ZR2024QH422), Taishan Scholars Program of Shandong Province (No tsqn202408385), Shandong Province medical health science and technology project (No. 202307010258, No.202307011110, No.202307010241, No.202307011364), Key R&D Program of Shandong Province, China (No.2023CXPT038).

## Conflicts of Interest

The authors declare no conflicts of interest.

## Supporting information




**Supporting File 1**: advs75263‐sup‐0001‐SuppMat.docx.

**Supporting File 2**: advs75263‐sup‐0002‐FigureS1.docx.

**Supporting File 3**: advs75263‐sup‐0003‐Data1.xlsx.

**Supporting File 4**: advs75263‐sup‐0004‐Data2.xlsx.

**Supporting File 5**: advs75263‐sup‐0005‐Data3.xlsx.

**Supporting File 6**: advs75263‐sup‐0006‐Data4.xls.

**Supporting File 7**: advs75263‐sup‐0007‐Data5.zip.

## Data Availability

All data are available in the main text or the supplementary materials.
