# Supplementary material for: GATA4‐Driven Transcription of HtrA1 Promotes Cellular Senescence in Ménière's Disease and Age‐Related Audio‐Vestibular Dysfunction
Source: Adv Sci (Weinh). 2026 Apr 14;13(39):e12538. doi: 10.1002/advs.202512538 (PMC13334991; doi:10.1002/advs.202512538)
Supplement: Supplementary file 1 — Supporting File 1: advs75263‐sup‐0001‐SuppMat.docx. [file ADVS-13-e12538-s002.docx]

Supporting Information

GATA4-Driven Transcription of HtrA1 Promotes Cellular Senescence in Ménière's Disease and Age-Related Audio-Vestibular Dysfunction

Na Zhang, Na Li*, Yan Wang, Jing Zhang, Jiahui Liu, Lei Chen, Yongdong Song, Yurong Mu, Yuechen Han, Yafeng Lyu, Xiaofei Li, Hanyue Wang, Jing Wang, Yao Lu, Zhaomin Fan, Daogong Zhang*, Haibo Wang*

**This PDF file includes:**

Fig S1 to S13

Tables S1 to S4

**Other Supplementary Materials for this manuscript include the following:**

Data files S1 to S4

Supplementary Figures


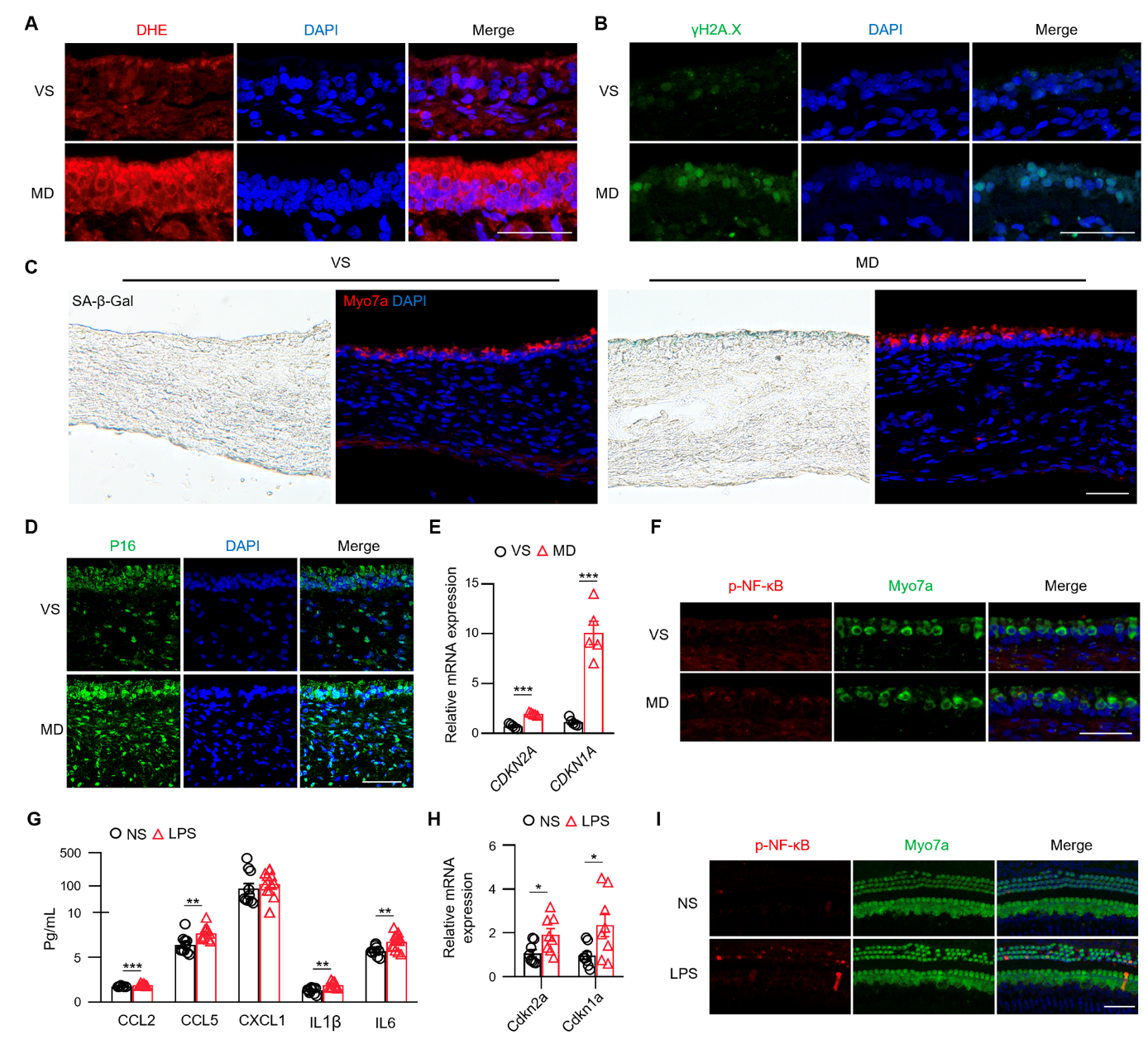


**Figure S1.** **HC senescence in patients with MD and EH mice.** (**A**) Representative confocal microscopy images of single color for Figure 1B showing DHE (red) and DAPI (blue) staining in the VEOs of VS and MD patients (scale bar, 10 μm). (**B**) Representative confocal microscopy images of single color for Figure 1B showing γH2A.X (green) and DAPI (blue) staining in the VEOs of VS and MD patients (scale bar, 10 μm). (**C**) Left: Representative images of SA-β-gal staining; right: confocal microscopy images showing Myo7a (red) and DAPI (blue) staining in VEOs of VS and MD patients (scale bar, 10 μm). (**D**) Representative confocal microscopy images showing P16 (green) and DAPI (blue) staining in the macula of VS and MD patients (scale bar, 50 μm). (**E**) The mRNA levels of P16 and P21 in VEOs of VS (n = 5) and MD (n = 5). (**F**) Representative confocal microscopy images showing p-NF-κB (red), Myo7a (green), and DAPI (blue) staining in VEOs of VS and MD patients (scale bar, 50 μm). (**G**) Multiple cytokines and chemokines were detected with the ABplex Mouse 5-Plex Custom Panel in the cochlea of mice treated with LPS (n = 12) or saline (n = 10). (**H**) The mRNA levels of P16 and P21 in cochlea of mice treated with LPS (n = 8) or saline (n = 8). (**I**) Representative confocal microscopy images showing p-NF-κB (red), Myo7a (green), and DAPI (blue) staining in the HCs of mice treated with LPS or saline (scale bar, 50 μm). Data are shown as mean ± SEM. Data were analyzed by unpaired t test [(E), (G), and (H)]. *p < 0.05, **p < 0.01, ***p < 0.001.


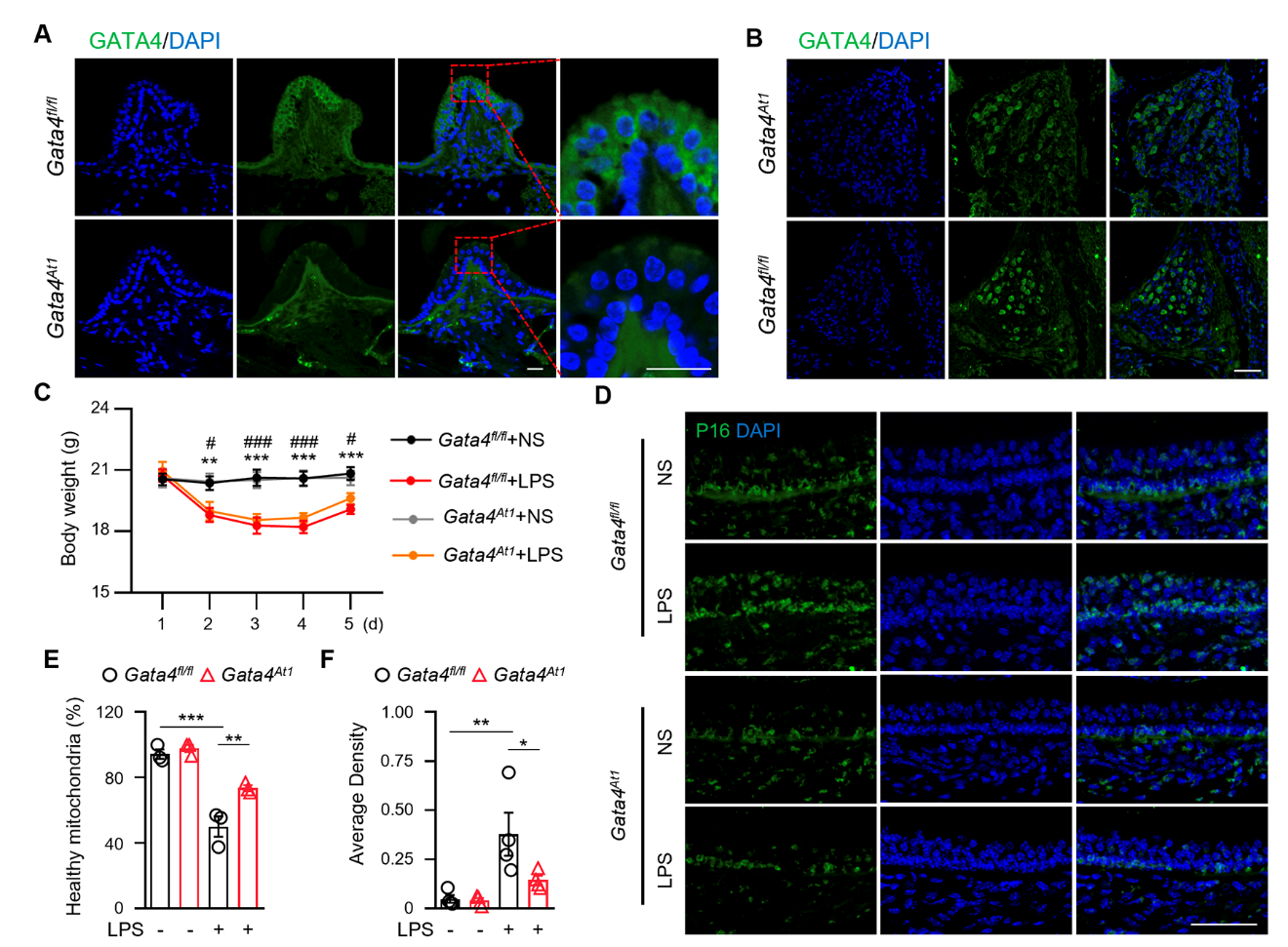


**Figure S2.** **GATA4 staining patterns and HC senescence of mice.** (**A**) Representative confocal microscopy images showing GATA4 (green) and DAPI (blue) staining in the VEOs of *Gata4^fl/fl^* and *Gata4^At1^* mice (scale bar, 20 μm). (**B**) Representative confocal microscopy images showing GATA4 (green) and DAPI (blue) staining in the spiral ganglion neurons of *Gata4^fl/fl^* and *Gata4^At1^* mice (scale bar, 50 μm). (**C**) Changes of body weight in all groups (n = 8). * significant difference compared with *Gata4^fl/fl^+*NS and *Gata4^fl/fl^+*LPS, # significant difference compared with *Gata4^At1^*+NS and *Gata4^At1^*+LPS. (**D**) Representative confocal microscopy images showing P16 (green), and DAPI (blue) staining in the macula of mice treated with LPS or saline (scale bar, 50 μm). (**E**) Quantification of healthy mitochondria in HCs of mice (n = 3). (**F**) Quantification of SA-β-gal staining in HCs of mice (n = 4). Data are shown as mean ± SEM. Data were analyzed by one-way ANOVA with LSD multiple comparisons posttests [(C), (E), and (F)]. * or # p < 0.05, **p < 0.01, *** or ### p < 0.001.


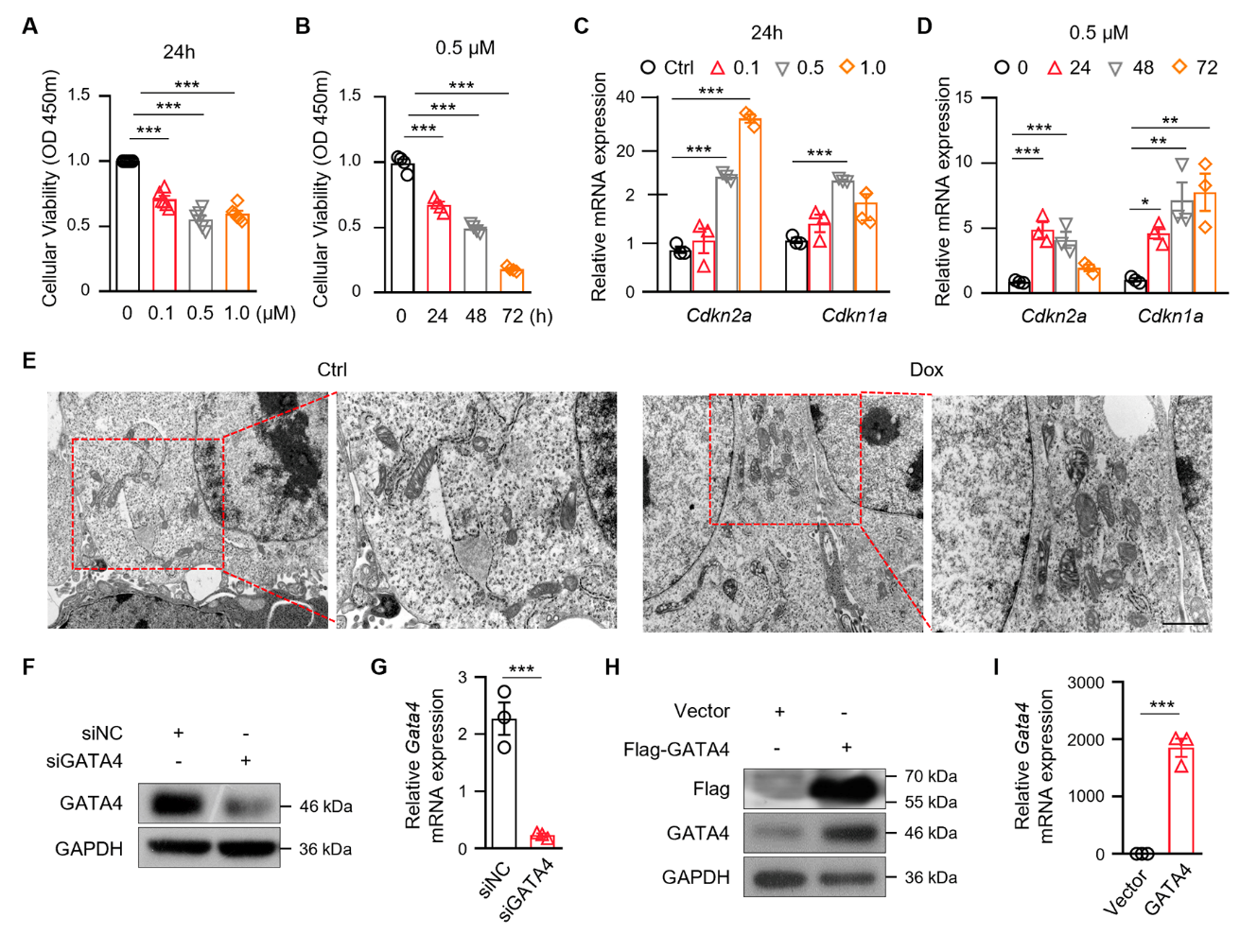


**Figure S3.** **Doxorubicin mediates cellular senescence in HEI-OC1 cells.** (**A**) The cellular viability of HEI-OC1 cells stimulated with Doxorubicin for 24 h (n = 6). (**B**) The cellular viability of HEI-OC1 cells at 0 h, 24 h, 48 h, and 72 h following stimulation with Doxorubicin for 24 h (n = 6). (**C, D**) The mRNA levels of P16 and P21 in HEI-OC1 cells (n = 3). (**E**) The representative electron microscopy images of HEI-OC1 cells stimulated with control or 0.5 μM Doxorubicin for 24 h (scale bar, 1 μm). (**F**) Western blot analysis of GATA4 expression in HEI-OC1 cells following GATA4 siRNA-transfected. (**G**) The mRNA levels of GATA4 in HEI-OC1 cells following GATA4 siRNA-transfected (n = 3). (**H**) Western blot analysis of GATA4 expression in HEI-OC1 cells following vector or GATA4 plasmid transfection. (**I**) The mRNA levels of GATA4 in HEI-OC1 cells following vector or GATA4 plasmid transfection (n = 3). Data are shown as mean ± SEM. Data were analyzed by one-way ANOVA with LSD multiple comparisons posttests [(A), (B), (C), (D), (G), and (I)]. ***p < 0.001.


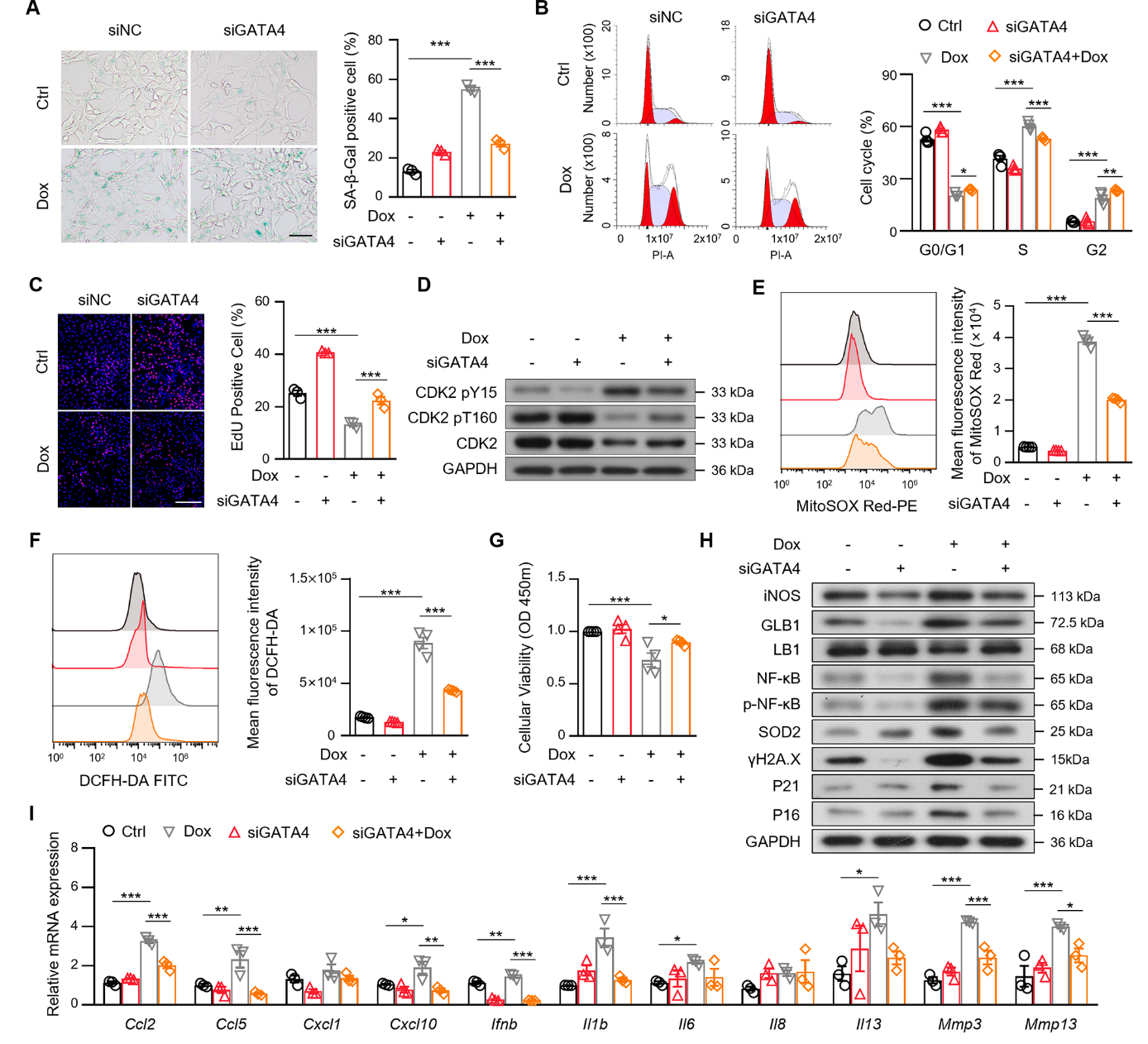


**Figure S4.** **GATA4 downregulation rescues Doxorubicin-induced cellular senescence in HEI-OC1 cells.** (**A**) Representative images (left) and quantification (right) of SA-β-gal staining in HEI-OC1 cells stimulated with GATA4 siRNA-transfected and Doxorubicin (0.5 μM, 24 h) (scale bar, 50 μm, n = 3). (**B**) Cell cycle analysis of HEI-OC1 cells infected with siGata4 or control siRNA with/without Doxorubicin assessed by flow cytometry (left) with percentage of cells in each phase (right) (n = 4). (**C**) Representative confocal microscopy images showing EdU (red) and DAPI (blue) staining for HEI-OC1 cells, which were incubated with EdU for 2 h following GATA4 siRNA-transfected and Doxorubicin (0.5 μM, 24 h) (n = 3). Scale bar, 200 μm. (**D**) Western blot analysis of CDK2 protein expression in HEI-OC1 cells. (**E**) Flow cytometry analysis (left) and quantification (right) of ROS levels in HEI-OC1 cells using Mito-SOX Red (n = 4). (**F**) Flow cytometry analysis (left) and quantification (right) of ROS levels in HEI-OC1 cells using DCFH-DA (n = 4). (**G**) The cellular viability of HEI-OC1 cells (n = 4). (**H**) Western blot analysis of senescence-associated protein expression in HEI-OC1 cells. (**I**) The mRNA levels of SASP in HEI-OC1 cells (n = 3). Data are shown as mean ± SEM. Data were analyzed by one-way ANOVA with LSD multiple comparisons posttests [(A), (B), (C), (D), (E), (F), (G),and (I)]. *p < 0.05, **p < 0.01, ***p < 0.001.


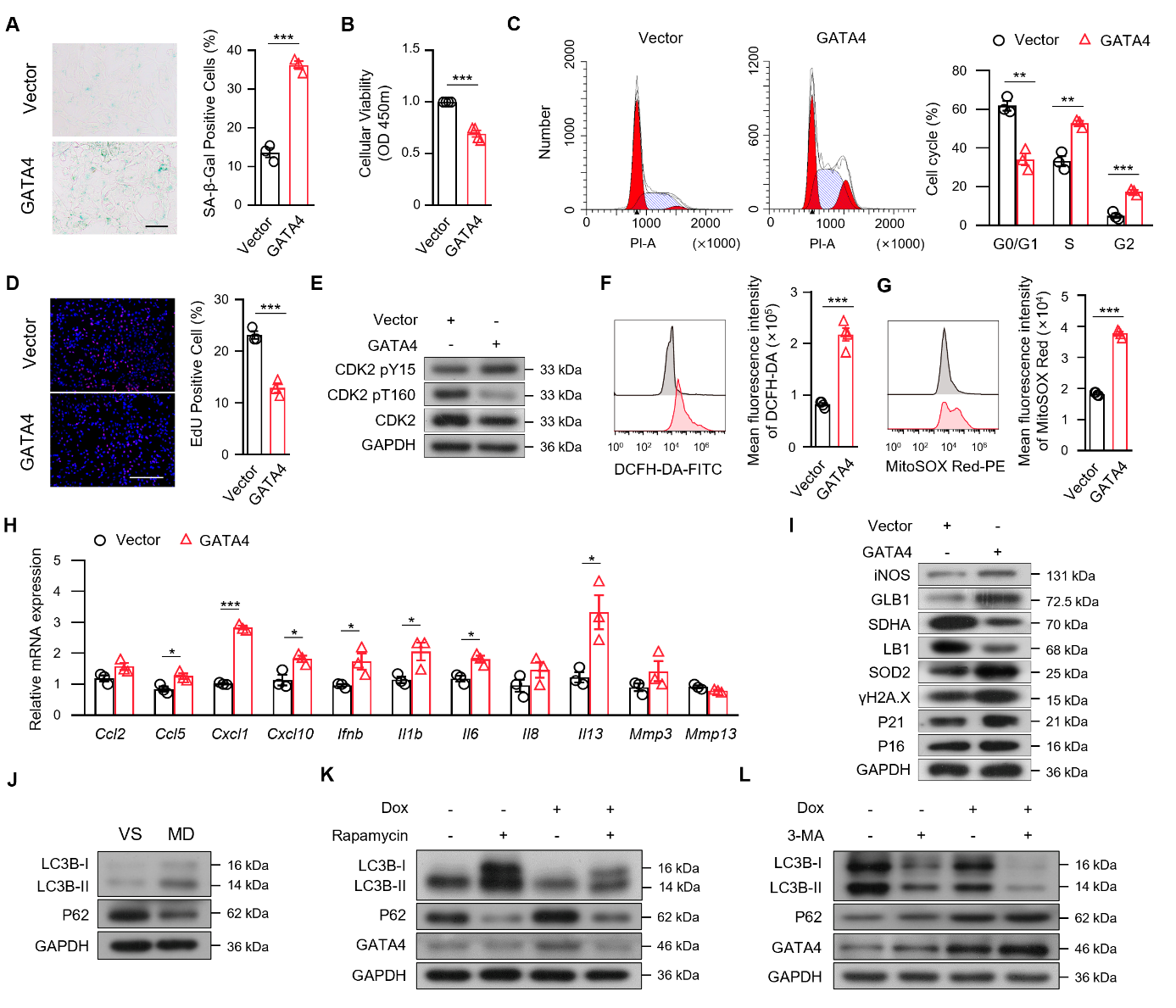


**Figure S5.** **GATA4 enhances the changes associated with cellular senescence in HEI-OC1 cells.** (**A**) Representative images (left) and quantification (right) of SA-β-gal staining in HEI-OC1 cells following vector or GATA4 plasmid transfection (scale bar, 50 μm, n = 3). (**B**) The cellular viability of HEI-OC1 cells following vector or GATA4 plasmid transfection (n = 4). (**C**) Cell cycle analysis of HEI-OC1 cells following vector (n = 3) or GATA4 plasmid transfection (n = 4) assessed by flow cytometry (left) with percentage of cells in each phase (right). (**D**) Representative confocal microscopy images showing EdU (red) and DAPI (blue) staining for HEI-OC1 cells, which were incubated with EdU for 2 h following vector or GATA4 plasmid transfection (scale bar, 100 μm, n = 3). (**E**) Western blot analysis of CDK2 expression in HEI-OC1 cells following vector or GATA4 plasmid transfection. (**F**) Flow cytometry analysis (left) and quantification (right) of ROS levels in HEI-OC1 cells using DCFH-DA (n = 4). (**G**) Flow cytometry analysis (left) and quantification (right) of ROS levels in HEI-OC1 cells using Mito-SOX Red (n = 4). (**H**) The mRNA levels of SASP in HEI-OC1 cells following vector or GATA4 plasmid transfection (n = 3). (**I**) Western blot analysis of senescence-associated protein expression in HEI-OC1 cells following vector or GATA4 plasmid transfection. (**J**) Western blot analysis of the protein levels for LC3B and P62 in VEOs of VS and MD. (**K**) Western blot analysis of the protein levels for LC3B, P62 and GATA4 in normal and Doxorubicin-induced senescent HEI-OC1 cells treated with or without rapamycin (50 nM) for 48 hours. (**L**) Western blot analysis of the protein levels for LC3B, P62 and GATA4 in normal and Doxorubicin-induced senescent HEI-OC1 cells treated with or without 3-MA (1 mM) for 48 hours. Data are shown as mean ± SEM. Data were analyzed by unpaired t test [(A), (B), (D), (F), (G), and (H)]. *p < 0.05, ***p < 0.001.


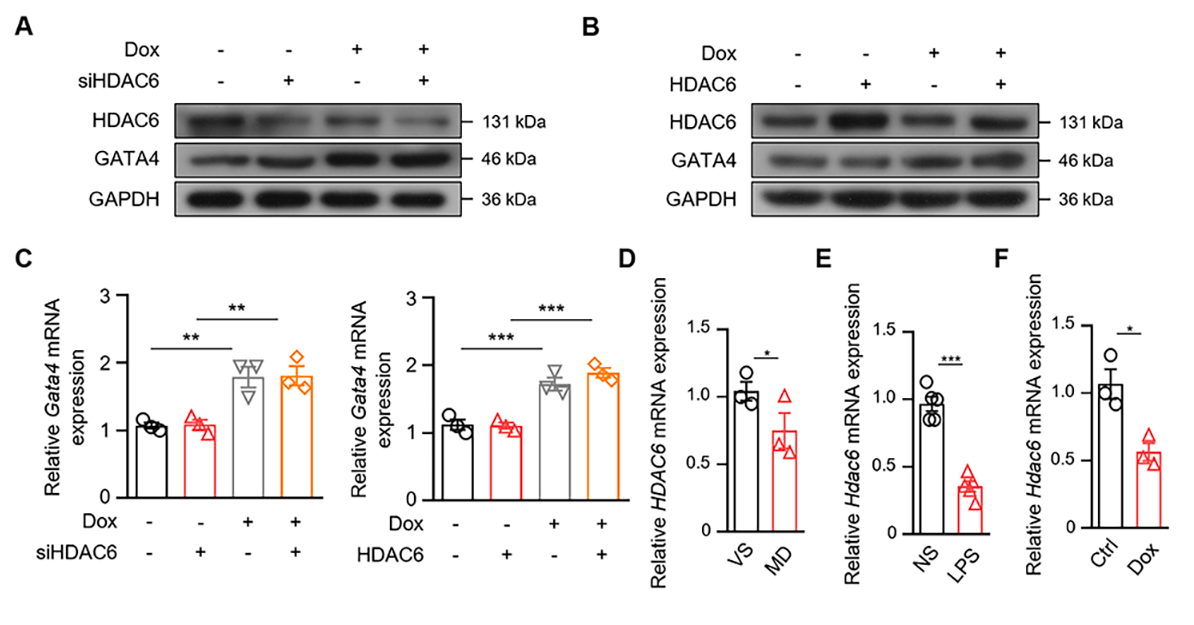


**Figure S6. The expression of HDAC6 and GATA4.** (**A,B**) Western blot analysis of HDAC6 and GATA4 protein expression in HEI-OC1 cells. (**C**) The mRNA levels of GATA4 in HEI-OC1 cells (n = 3). (**D,E,F**) The mRNA levels of HDAC6 the VEOs of VS and MD patients (n = 3) (**D**), cochleae of mice treated with LPS or saline (n = 5) (**E**), HEI-OC1 cells stimulated with Doxorubicin or saline (n = 3) (**F**). Data are shown as mean ± SEM. Data were analyzed by one-way ANOVA with LSD multiple comparisons posttests [(B) and (C)] and unpaired t test [(D), (E), and (F)]. *p < 0.05, **p < 0.01, ***p < 0.001.


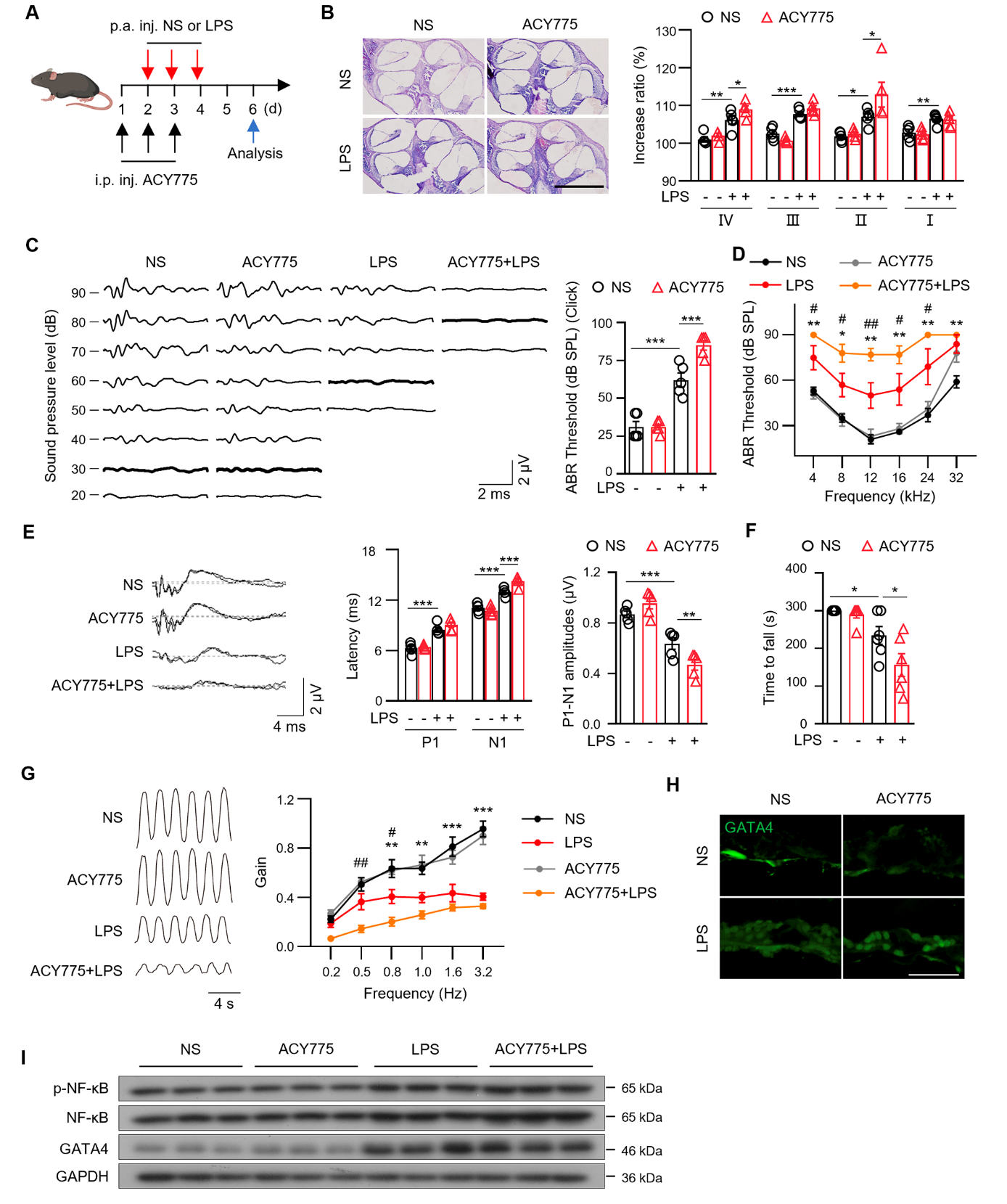


**Figure S7. Pharmacological inhibition of HDAC6 aggravates the severity of LPS-induced EH and audio-vestibular symptoms in vivo.** (**A**) Schematic representation of the time scales for analysis. Mice aged 6-8 weeks were treated with the HDAC6 inhibitor ACY775 (10 mg/kg, i.p.) for 3 consecutive days and LPS (10 mg/kg, p.a.) for 3 consecutive days from the second day. All mice were then subjected to analysis on day 6. (**B**) Representative images (left) and quantification (right) of mid-modiolar cochlear sections, scale bar = 200 μm. Measurements of IR-L in cochlear half-turns I–Ⅳ (n = 5). (**C**) Representative serial ABR wave recordings and thresholds in response to click sounds (n = 5). (**D**) ABR thresholds in response to pure tone bursts across all frequencies (n = 5). * significant difference compared with NS and LPS; # significant difference compared with LPS and ACY775+LPS. (**E**) Representative click-evoked VEMP waves, the P1 and N1 peak latencies, and P1-N1 peak amplitudes of VEMPs at 100 dB nHL (n = 5). (**F**) Quantification of rotarod test (n = 6). (**G**) Representative horizontal VOR waves of all groups. The VOR gains are plotted for all groups at a peak velocity (20º/s) and frequencies range from 0.2 Hz to 3.2 Hz (n = 5), * significant difference compared with NS and LPS; # significant difference compared with LPS and ACY775+LPS. (**H**) Representative confocal microscopy images showing GATA4 (green) staining in the HCs (scale bar, 50 μm). (**I**) Western blot analysis of NF-κB and GATA4 protein expression in the inner ear. Data are shown as mean ± SEM. Data were analyzed by one-way ANOVA with LSD multiple comparisons posttests [(B), (C), (D), (E), (F), and (G)]. * or # p < 0.05, ** or ## p < 0.01, *** p < 0.001.


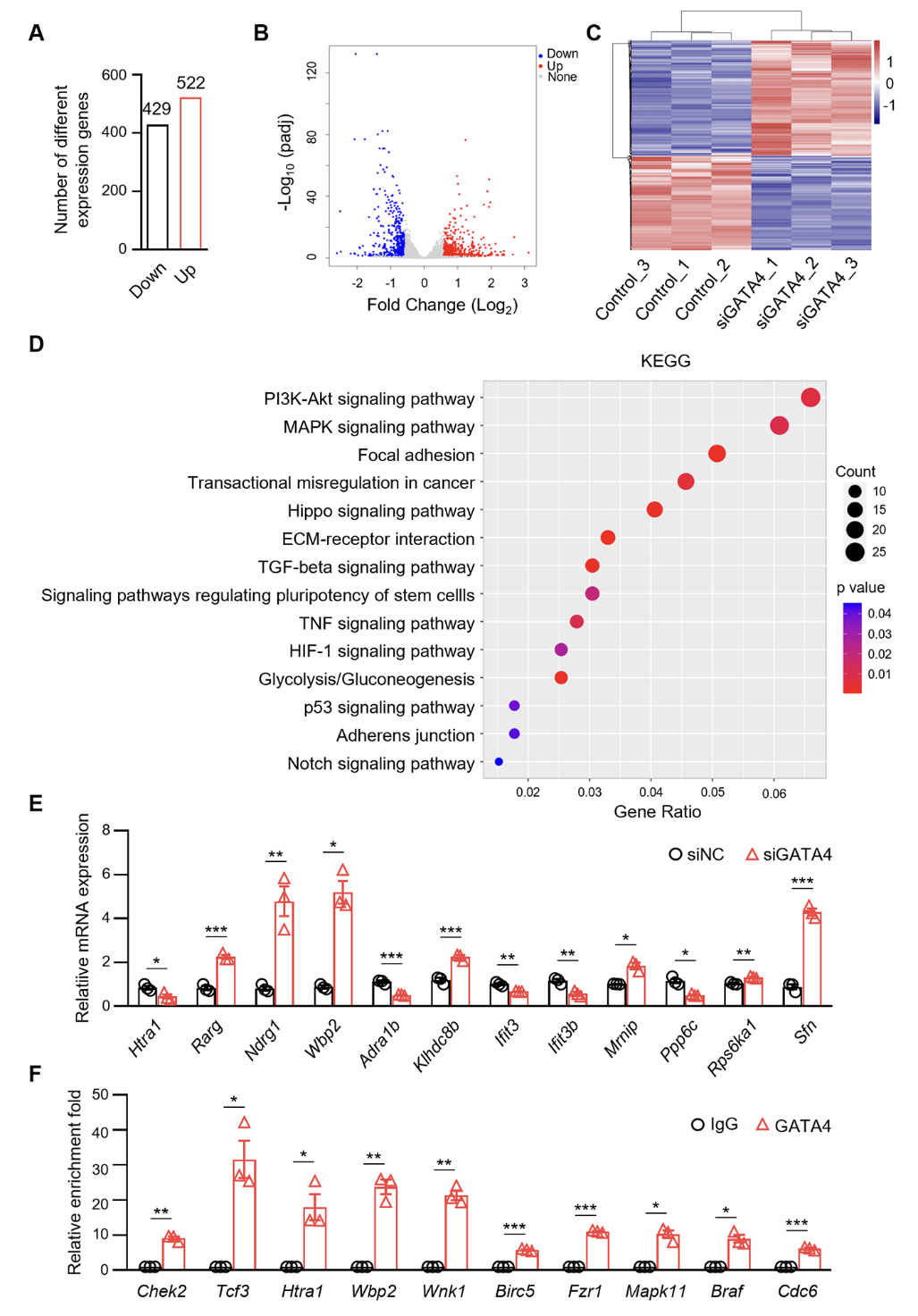


**Figure S8.** **Identification and functional analysis of differentially expressed genes in GATA4-depleted HEI-OC1 cells.** (**A**) Histogram representing the distribution of differentially expressed genes (DEGs) identified in control and siGata4 cells (fold-change > 1.5, padj < 0.05). (**B**) Volcano plot showing gene expression differences among all genes identified in control and siGata4 cells (red, upregulated genes; blue, downregulated genes; grey, unchanged genes). (**C**) Heatmap of DEGs in control and siGata4 cells. (**D**) KEGG pathway analysis of DEGs. (**E**) The mRNA levels of selected DEGs in HEI-OC1 cells following GATA4 siRNA-transfected (n = 3). (**F**) Verification of ChIP-seq results through qChIP analysis of genes using antibodies against GATA4 in HEI-OC1 cells (n = 3). Results are expressed as fold-change relative to IgG, and GAPDH was used as a negative control. Data are shown as mean ± SEM. Data were analyzed by unpaired t test [(E) and (F)]. *p < 0.05, **p < 0.01, ***p < 0.001.


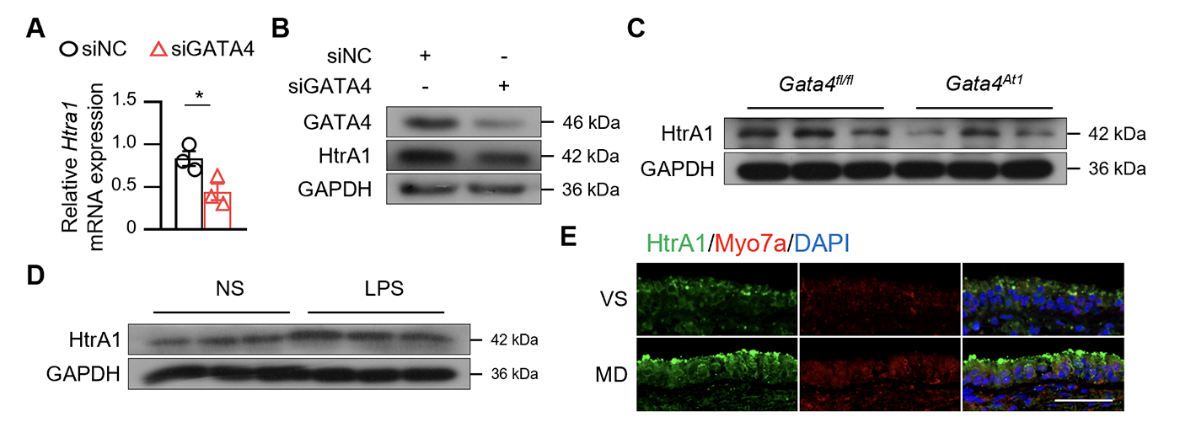


**Figure S9.** **GATA4 activates HtrA1 transcriptional activity.** (**A**) The mRNA levels of HtrA1 in HEI-OC1 cells following GATA4 siRNA-transfected (n = 3). (**B**) Western blot analysis of HtrA1 expression in HEI-OC1 cells following GATA4 siRNA-transfected. (**C**) Western blot analysis of HtrA1 protein expression in cochleae of *Gata4^fl/fl^* and *Gata4^At1^* mice. (**D**) Western blot analysis of HtrA1 protein expression in cochleae of mice treated with LPS or saline. (**E**) Representative confocal microscopy images showing HtrA1 (green), GATA4 (red) and DAPI (blue) staining in the VEOs of VS and MD patients (scale bar, 50 μm). Data are shown as mean ± SEM. Data were analyzed by unpaired t test [(A)]. *p < 0.05.


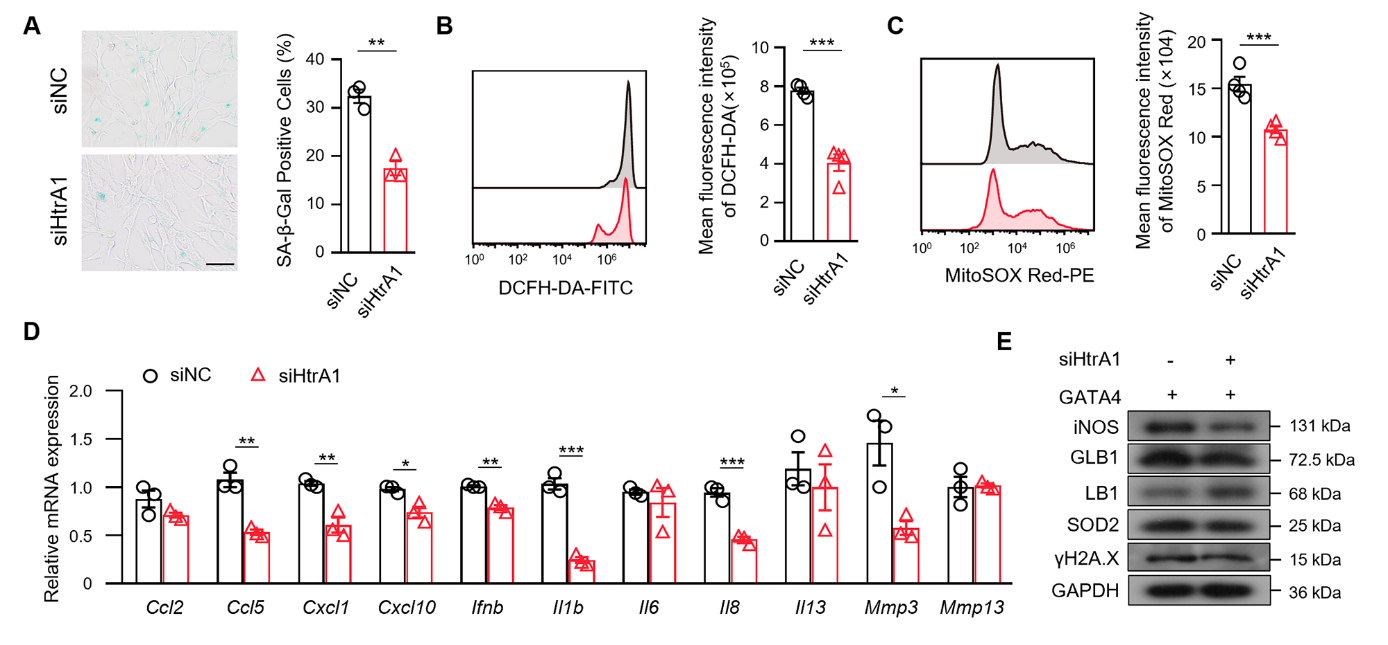


**Figure S10. HtrA1 downregulation rescues GATA4 overexpression-induced cellular senescence in HEI-OC1 cells.** (**A**) Representative images (left) and quantification (right) of SA-β-gal staining in HEI-OC1 cells following GATA4 plasmid transfection and HtrA1 siRNA transfection (scale bar, 50 μm) (n = 3). (**B**) Flow cytometry analysis (left) and quantification (right) of ROS levels in HEI-OC1 cells using DCFH-DA (n = 4). (**C**) Flow cytometry analysis (left) and quantification (right) of ROS levels in HEI-OC1 cells using Mito-SOX Red (n = 4). (**D**) The mRNA levels of SASP in HEI-OC1 cells following GATA4 plasmid transfection and HtrA1 siRNA transfection (n = 3). (**E**) Western blot analysis of senescence-associated protein expression in HEI-OC1 cells following GATA4 plasmid transfection and HtrA1 siRNA transfection. Data are shown as mean ± SEM. Data were analyzed by unpaired t test [(A), (B), (C), and (D)]. *p < 0.05, **p < 0.01, ***p < 0.001.


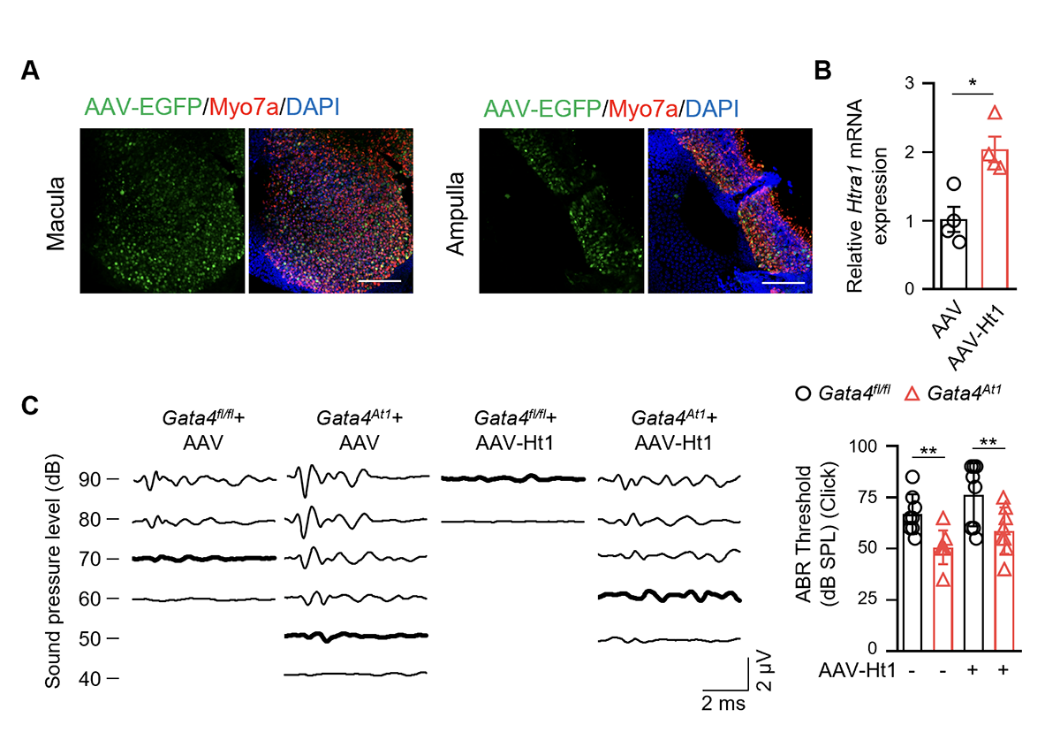


**Figure S11.** **Effects of HtrA1 transduction on inner ear hair cells, HtrA1 expression and hearing levels in mice.** (**A**) Representative images of the control virus infecting inner ear HCs in P3 mice (scale bar, 100 μm). (**B**) The transcriptional expression levels of HtrA1 in the VEOs of the HtrA1-transduced cochlea (n = 4). (**C**) Representative serial ABR wave recordings and thresholds in response to click sounds (n = 8). Data are shown as mean ± SEM. Data were analyzed by unpaired t test [(B)] and one-way ANOVA with LSD multiple comparisons posttests [(C)]. *p < 0.05, **p < 0.01.


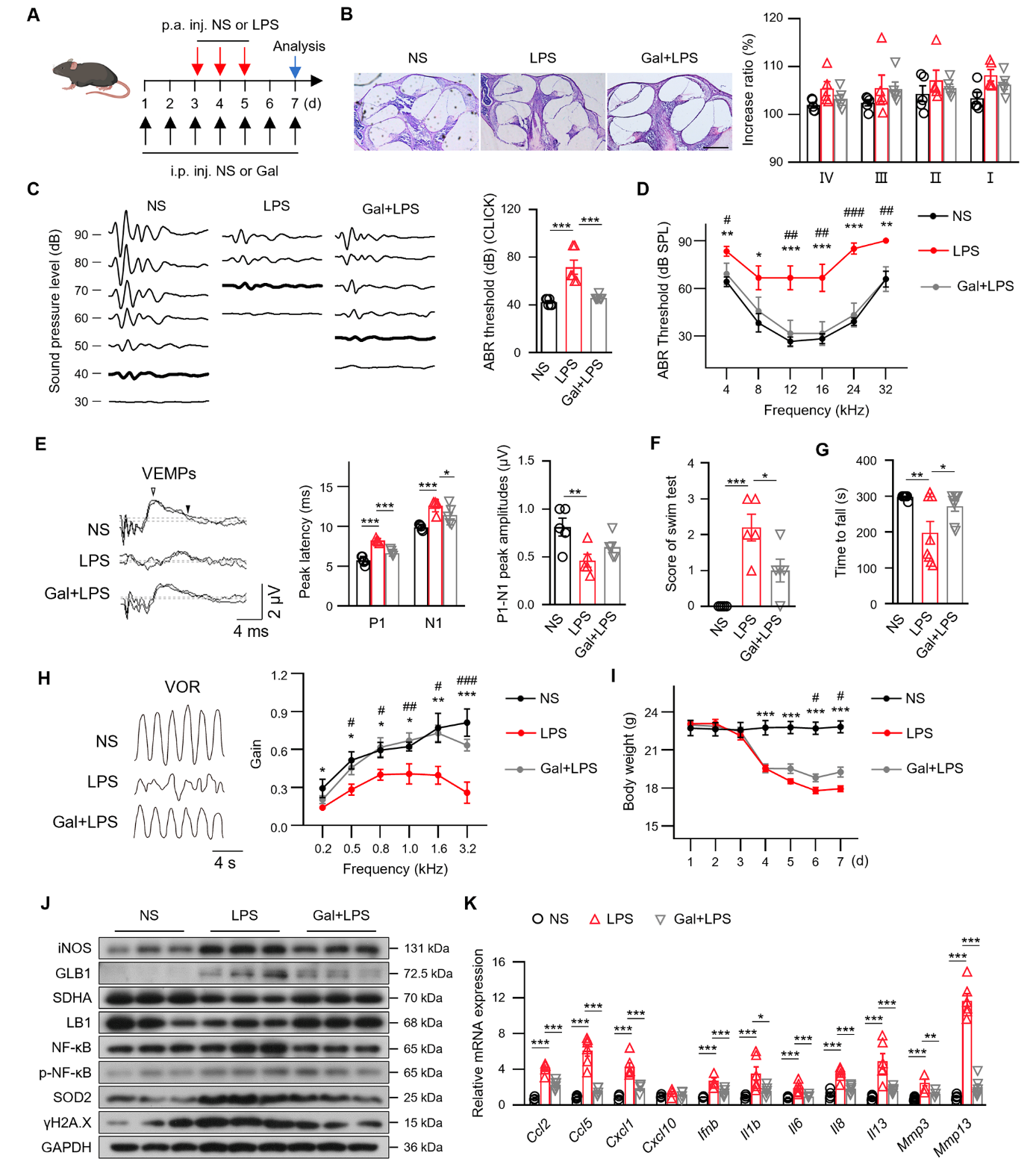


**Figure S12.** **Pharmacological inhibition of HtrA1 ameliorates the severity of LPS-induced EH and audio-vestibular symptoms in vivo.** (**A**) Schematic representation of the time scales for analysis. The 6-8 week-mice were treated with HtrA1 inhibitor Galegenimab (6 mg/kg, i.p.) for 7 consecutive days, LPS (10 mg/kg, p.a.) for 3 consecutive days from third days for 3 consecutive days, then analyzed at 7 days. (**B**) Representative images of mid-modiolar cochlear sections, scale bar = 100 μm. Measurements of IR-L in cochlear half-turns I–Ⅳ (n = 5). (**C**) Representative serial ABR wave recordings and thresholds in response to click sounds (n=6). (**D**) ABR thresholds in response to pure tone bursts across all frequencies (n = 6). * significant difference compared with NS and LPS; # significant difference compared with LPS and Gal*+*LPS. (**E**) Representative click-evoked VEMP waves, the P1 (white triangle) and N1 (black triangle) peak latencies, and P1-N1 peak amplitudes of VEMPs at 100 dB nHL (n = 5). (**F**) Swimming posture scores of swim tests (n = 5). (**G**) Quantification of rotarod test (n = 8). (**H**) Representative horizontal VOR waves of all groups. The VOR gains and phases are plotted for all groups at a peak velocity (20º/s) and frequencies range from 0.2 Hz to 3.2 Hz (n = 6), * significant difference compared with NS and LPS; # significant difference compared with LPS and Gal*+*LPS. (**I**) Changes of body weight in all groups (n = 4 or 5), * significant difference compared with NS and LPS; # significant difference compared with LPS and Gal*+*LPS. (**J**) Western blot analysis of senescence-associated protein expression in the inner ear. (**K**) The mRNA levels of SASP in the inner ear (n = 6). Data are shown as mean ± SEM. Data were analyzed by one-way ANOVA with LSD multiple comparisons posttests [(B), (C), (D), (E), (F), (G), (H), (I), and (K)]. * or # p < 0.05, ** or ## p < 0.01, *** or ### p < 0.001.


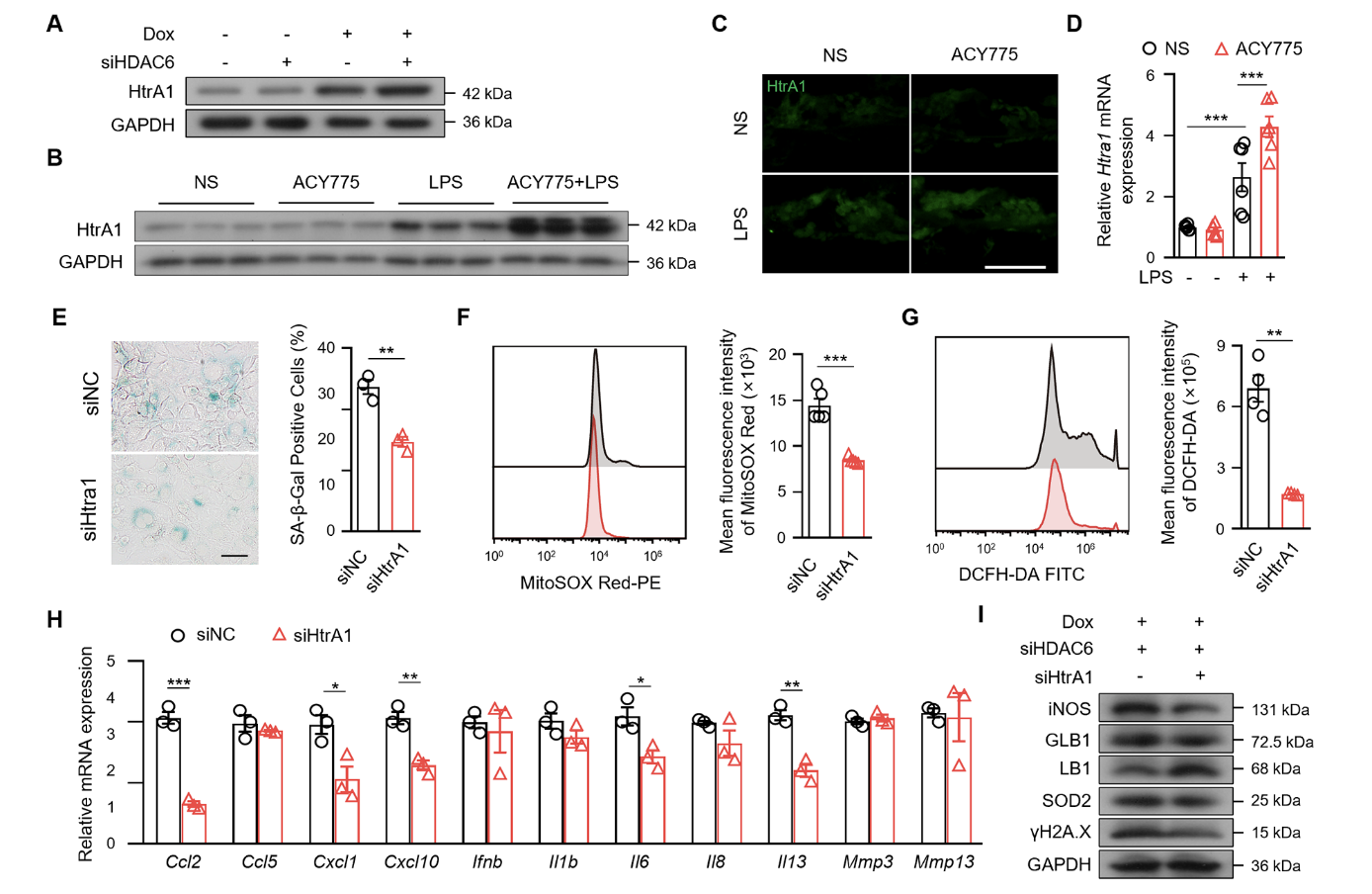


**Figure S13. HtrA1 downregulation rescues HDAC6 depletion-induced cellular senescence in HEI-OC1 cells.** (**A**) Western blot analysis of HtrA1 protein expression in HEI-OC1 cell stimulated with HDAC6 siRNA-transfected and Doxorubicin (0.5 μM, 24 h). (**B**) Western blot analysis of HtrA1 protein expression in the inner ear. (**C**) Representative confocal microscopy images showing HtrA1 (green) staining in the HCs (scale bar, 50 μm). (**D**) The mRNA levels of HtrA1 in the inner ear (n = 6). (**E**) Representative images (left) and quantification (right) of SA-β-gal staining in HEI-OC1 cells following HDAC6 siRNA and HtrA1 siRNA transfection (scale bar, 50 μm). (**F**) Flow cytometry analysis (left) and quantification (right) of ROS levels in HEI-OC1 cells using DCFH-DA (n = 4). (**G**) Flow cytometry analysis (left) and quantification (right) of ROS levels in HEI-OC1 cells using Mito-SOX Red (n = 4). (**H**) The mRNA levels of SASP in HEI-OC1 cells following HDAC6 siRNA and HtrA1 siRNA transfection (n = 3). (**I**) Western blot analysis of senescence-associated protein expression in HEI-OC1 cells following HDAC6 siRNA and HtrA1 siRNA transfection. Data are shown as mean ± SEM. Data were analyzed by unpaired t test [(D), (E), (F), (G), and (H)]. * p < 0.05, ** p < 0.01, *** p < 0.001.

| **Supplementary Tables**  **Table S1 The general features and clinical characteristics of the patients** | | | | | | | | |
| --- | --- | --- | --- | --- | --- | --- | --- | --- |
| **Patient ID** | **MD-1** | **MD-2** | **MD-3** | **MD-4** | **MD-5** | **MD-6** | **MD-7** | **MD-8** |
| Sample available | VO | VO | VO | VO | VO | VO | VO | VO |
| Use in study | PCR | PCR | PCR, TEM | PCR, WB | PCR, WB | WB | IF | IF |
| Age | 61 | 74 | 62 | 68 | 50 | 43 | 49 | 64 |
| Gender | Female | Female | Female | Female | Male | Female | Male | Female |
| Age of onset | 58 | 70 | 57 | 57 | 48 | 35 | 48 | 63 |
| Side | Right | Left | Left | Left | Right | Right | Left | Right |
| PTA (dB) | 66.25 | 67.5 | 85 | 81.25 | 72.5 | 90 | 93.75 | 77.5 |
| Tumarkin crisis | None | None | None | None | None | None | None | None |
| High blood pressure | None | Yes | Yes | None | None | None | None | None |
| Diabetes | None | None | None | None | None | None | None | None |
| Autoimmune disease | None | None | Yes | None | None | None | None | None |
| Migraine | None | None | None | None | None | None | None | None |
| cVEMP | Normal | Abnormal | Normal | Abnormal | Normal | Abnormal | Abnormal | Abnormal |
| oVEMP | Abnormal | Abnormal | Abnormal | Normal | Abnormal | Abnormal | Abnormal | Normal |
| UW (%) | 45 | 34 | 28.6 | 97 | 36.6 | 96 | 84.1 | 44 |
| Grading of endolymphatic hydrops | 4 | 3 | 4 | 4 | 2 | 4 | 4 | 4 |
| Aminoglycoside treatment | None | None | None | None | None | None | None | None |
| Steroid treatment | Yes | Yes | Yes | Yes | Yes | Yes | Yes | Yes |
| **Patient ID** | **VS-1** | **VS-2** | **VS-3** | **VS-4** | **VS-5** | **VS-6** | **VS-7** | **VS-8** |
| Sample available | VO | VO | VO | VO | VO | VO | VO | VO |
| Use in study | PCR | PCR | PCR, TEM | PCR, WB | PCR, WB | WB | IF | IF |
| Age | 64 | 52 | 43 | 54 | 57 | 70 | 55 | 65 |
| Gender | Female | Female | Female | Male | Female | Female | Female | Female |
| Age of onset | 50 | 52 | 41 | 49 | 52 | 53 | 53 | 55 |
| Side | Right | Right | Right | Left | Right | Left | Right | Right |
| PTA (dB) | 71.25 | 36.25 | 75 | 78.75 | 93.75 | 110 | 53.75 | 60 |
| Tumarkin crisis | None | None | None | None | None | None | None | None |
| High blood pressure | None | None | None | None | None | Yes | None | None |
| Diabetes | None | None | None | None | None | None | None | None |
| Autoimmune disease | None | None | None | Yes | None | None | None | None |
| Migraine | None | None | None | None | None | None | None | None |
| cVEMP | Normal | Abnormal | Normal | Normal | Abnormal | Normal | Abnormal | Abnormal |
| oVEMP | Normal | Normal | Normal | Abnormal | Abnormal | Normal | Abnormal | Abnormal |
| UW (%) | 8.7 | 76.8 | 74 | 6.93 | - | 36.7 | 72.5 | 72.5 |
| Grading of endolymphatic hydrops | - | - | - | - | - | - | - | - |
| Aminoglycoside treatment | None | None | None | None | None | None | None | None |
| Steroid treatment | None | None | None | None | None | None | None | None |
| PTA, pure tone average of 0.5, 1, 2 and 4 kHz; UW, unilateral weakness; cVEMP, vestibular evoked myogenic potential; oVEMP, ocular vestibular evoked myogenic potential. Grading of endolymphatic hydrops using magnetic resonance imaging according to the four-stage grading system. | | | | | | | | |

**Table S2: Lists of chemicals and other resources.**

| REAGENT or RESOURCE | SOURCE | IDENTIFIER |
| --- | --- | --- |
| Biological samples | | |
| Human vestibular end organs | Shandong ENT hospital | XYK20190814 |
| Chemicals, peptides, and recombinant proteins | | |
| Accutase | Thermo Fisher Scientific | Cat# A1110501 |
| ACY-775 | Selleck.cn | Cat# S0864 |
| Ampicillin (100mg/ml solution) | Solarbio | Cat# A1170 |
| Antigen retrieval solution | Beyotime | Cat# P0088 |
| Cell lysis buffer for Western and IP | Beyotime | Cat# P0013 |
| Chemiluminescent HRP substrate | Millipore | Cat# WBKLS0500 |
| Cole’s Hematoxylin Solution(For Conventional Stain) | Solarbio | Cat# G1140 |
| DAPI | Sigma | Cat# 28718-90-3 |
| DCFH-DA | Solarbio | Cat# D6470 |
| Dermafuse tissue adhesive | Millpledge Ltd. | Cat# DF00113 |
| Dihydroethidium | Thermo Fisher Scientific | Cat# D1168 |
| DMSO | Abcam | Cat# ab120497 |
| DNase I | Solarbio | Cat# D8070 |
| Doxorubicin hydrochloride | MCE | Cat# HY-15142 |
| EDTA Antigen Retrieval Solution | Beyotime | Cat# P0085 |
| Eosin | Beyotime | Cat# C0109 |
| Ethanol | Hushi | Cat# 64-17-5 |
| FBS | Gibco | Cat# 10099-141C |
| Film | Cardstream | Cat# XBT-1 |
| FLAG peptide | Sigma | Cat# F3290 |
| Galegenimab | MCE | Cat# HY-P99629 |
| Glycine | Beyotime | Cat# ST085 |
| Lipofectamine® RNAiMAX Reagent | Thermo Fisher Scientific | Cat# 13778-150 |
| LPS | Sigma | Cat# L2880 |
| Anti-FLAG M2 affinity gel | Sigma | Cat# A2220 |
| Methanol | Hushi | Cat# 67-56-1 |
| MitoSOX Red | Life Technologies | Cat# M36008 |
| Normal Donkey Serum | Solarbio | Cat# SL050 |
| OCT | Leica | Cat# 3801481 |
| Opti-MEM | Thermo Fisher Scientific | Cat# 31985-070 |
| PBS | Solarbio | Cat# P1020 |
| PEIpro | Polyplus | Cat# 115-0015 |
| Penicillin-Streptomycin Solution (10000 U/mL) | Gibco | Cat# 15140122 |
| PFA | Solarbio | Cat# P1110 |
| Phosphatase Inhibitor Cocktail I | MCE | Cat# HY-K0021 |
| Phosphatase Inhibitor Cocktail II | MCE | Cat# HY-K0022 |
| PMSF | Solarbio | Cat# P0100 |
| Primary Antibody Dilution Buffer | Beyotime | Cat# P0023A |
| ProLong Gold Antifade Mountant with DAPI | Thermo Fisher Scientific | Cat# P36935 |
| Protease inhibitor cocktail | Sigma | Cat# P8340 |
| Protein A Agarose Beads | CST | Cat# 9863 |
| Protein ladder | Thermo Fisher Scientific | Cat# 26620 |
| RIPA lysis buffer | Beyotime | Cat# P0013B |
| SDS-PAGE Sample Loading Buffer (6X) | Beyotime | Cat# P0015F |
| Skim milk | BioFroxx | Cat# 1172GR500 |
| Sucrose | Solarbio | Cat# S8271 |
| TBST | Solarbio | Cat# T1081 |
| TritonX-100 | Sigma | Cat# T8787 |
| TRIzol | Invitrogen | Cat# 15596018CN |
| Trypsin-EDTA (0.05%), phenol red | Gibco | Cat# 25300062 |
| Xylene | Hushi | Cat# 1330-20-7 |
| Critical commercial assays | | |
| Agilent 2100 High Sensitivity DNA Assay Kit | Agilent Technologies | Cat# 5067-4626 |
| BCA protein Assary kit | Beyotime | Cat# P0012 |
| Cell Counting Kit-8 | Beyotime | Cat# C0037 |
| DAB Detection Kit | ZSGB-BIO | Cat# SP9000 |
| Cell Cycle and Apoptosis Kit | BioScience | Cat# C6031S |
| Click-iT® EdU Imaging Kits | Invitrogen | Cat# C10337 |
| DNA Damage Assay Kit by γ-H2AX Immunofluorescence | Beyotime | Cat# C2035S |
| Dual-Luciferase® Reporter Assay System | Promega | Cat# E1910 |
| KAPA HiFi HotStart Ready Mix | Roche | Cat# KK2602 |
| Nuclear and Cytoplasmic Protein Extraction Kit | Beyotime | Cat# P0028 |
| Omni-ECL™ Ultra Sensitive Chemiluminescent Detection Kit (Femtocell Grade) | Epizyme | Cat# SQ201 |
| QubitTM dsDNA Assay Kit | Thermo Fisher Scientific | Cat# Q32854 |
| RevertAid Fist Strand cDNA Synthesis Kit | Thermo Fisher Scientific | Cat# K1622 |
| SDS-PAGE Gel Quick Preparation Kit | Beyotime | Cat# P0012AC |
| Senescence β-Galactosidase Staining Kit | Beyotime | Cat# C0602 |
| SimpleChIP® Plus Enzymatic Chromatin IP Kit (Magnetic Beads) | CST | Cat# 9005S |
| Single Cell 3′ Library Gel Bead Kit V3 | 10x Genomics | Cat# PN1000075 |
| SPRIWorks HT kit | Beckman Coulter | Cat# B06938 |
| SYBY Green Premix EX Taq | TakaRa | Cat# RR420A |
| Experimental models: Cell lines | | |
| HEI-OC1 cells | Neng et al., 2013 | N/A; RRID:CVCL_D899 |
| Experimental models: Organisms/strains | | |
| Mouse: C57BL/6 | Shandong university | C57BL/6J |
| Mouse:C57BL/6-Gata4^fl/fl^ | Cyagen Biosciences | C57BL/6J |
| Mouse: C57BL/6-Atoh1-Cre | Jackson Laboratory | C57BL/6J |
| Mouse: C57BL/6-Sgk1^tm1cyagen^ | Cyagen Biosciences | C57BL/6 |
| Software and algorithms | | |
| Agilent 2100 Bioanalyzer | Agilent Technologies | <https://www.agilent.com/zh-cn/product/automated-electrophoresis/bioanalyzer-systems/bioanalyzer-instrument/2100-bioanalyzer-instrument-228250> |
| Bioruptor Sonication System | Diagenode | <https://www.diagenode.com/en/p/bioruptor-plus-sonication-device> |
| BX53 System Microscope | OLYMPUS | <https://lifescience.evidentscientific.com.cn/zh/microscopes/upright/bx53f2/> |
| DESeq R package | R package | <https://bioconductor.org/packages/release/bioc/html/DESeq.html> |
| FASTQC software | Babraham Institute | <https://www.bioinformatics.babraham.ac.uk/projects/fastqc/> |
| Flow cytometry | BD Biosciences | <https://www.bdbiosciences.com/zh-cn/products/instruments/flow-cytometers/research-cell-analyzers/bd-accuri-c6-plus> |
| FlowJo | FlowJo, LLC | https://www.flowjo.com/ |
| FS800 Pathology Frozen Slicer | RWD | <https://www.rwdls.com/product-solutions/life-sciences/pathology/cryostats/data_64.html> |
| GO | InterProScan | <http://geneontology.org/> |
| GraphPad Prism | GraphPad Software | https://www.graphpad.com/ |
| ImageJ | NIH | https://imagej.nih.gov/ |
| KEGG |  | <https://www.kegg.jp/> |
| Leica LAS AF | Leica Microsystem | https://leica-las-af-lite.software.informer.com/4.0/ |
| Microtiter plate reader | BIO-RAD | <https://www.bio-rad.com/zh-cn/product/microplate-manager-software?ID=3532bc90-496d-42b8-baf2-55b5a598b501> |
| ModFit LT software | Verity Software House | <https://www.vsh.com/products/mflt/> |
| Neuro-Audio | Neurosoft | https://neurosoft.com/en/catalog/audio/neuro-audio |
| OFT | Shanghai Xinruan Information Technology Co., Ltd. | <https://www.shxinruan.com/sjjsxw/10.html> |
| PCR machine | Eppendorf | <https://www.eppendorf.com/cn-zh/Products/PCR/Thermocyclers/Mastercycler-nexus-X2-p-PF-82586> |
| PowerPac™ Basic Power Supply | BIO-RAD | <https://www.bio-rad.com/zh-cn/product/powerpac-basic-power-supply?ID=bea5dea1-cef0-43ad-8af5-b2c0287f6e07> |
| Rotating rod ZH-600B | Anhui Zhenghua Biologic Apparatus Facilities | <https://6062307.com/product/showproduct.php?id=111> |
| RZ6 auditory physiology workstation | Tucker-Davis Technologies | https://www.tdt.com/component/biosigrz-abr-dpoae-software/ |
| SPSS | SPSS Inc | N/A |
| STELLARIS Confocal Microscope | Leica | <https://cn.leica-microsystems.com.cn/product/life-science-microscope/confocal/STELLARIS> |
| Transmission Electron Microscope | JEOL | <https://www.jeol.com/products/scientific/tem/> |
| VEMP | INTELLIGENT HEARING | <https://ihsys.info/site/en/diagnostics/smartep/smartvemp/> |
| VOR | GIANT TEX | <http://www.gianttek.cn/about_9.html> |
| Other | | |
| Polyvinylidene difluoride membranes | Millipore | Cat# ISEQ00010 |

| **Table S3: RT-PCR Primers and siRNA** | | |
| --- | --- | --- |
| **Oligonucleotides** | **SOURCE** | **IDENTIFIER** |
| Human Ccl2 Forward primer 5'-AGTCTCTGCCGCCCTTCT-3' | This manuscript | N/A |
| Human Ccl2 Reverse primer 5'-GTGACTGGGGCATTGATTG-3' | This manuscript | N/A |
| Human Ccl5 Forward primer 5'-TGCCCACATCAAGGAGTATTT-3' | This manuscript | N/A |
| Human Ccl5 Reverse primer 5'-CTTTCGGGTGACAAAGACG-3' | This manuscript | N/A |
| Human Cxcl1 Forward primer 5'-CATCGAAAAGATGCTGAACAGT-3' | This manuscript | N/A |
| Human Cxcl1 Reverse primer 5'-ATAAGGGCAGGGCCTCCT-3' | This manuscript | N/A |
| Human Cxcl10 Forward primer 5'-CCAGAATCGAAGGCCATCAA-3' | This manuscript | N/A |
| Human Cxcl10 Reverse primer 5'-CATTTCCTTGCTAACTGCTTTCAG-3' | This manuscript | N/A |
| Human Gapdh Forward primer 5'-GAGTCAACGGATTTGGTCGT-3' | This manuscript | N/A |
| Human Gapdh Reverse primer 5'-TTGATTTTGGAGGGATCTCG-3' | This manuscript | N/A |
| Human Gata4 Forward primer 5'-GTGTCCCAGACGTTCTCAGTC-3' | This manuscript | N/A |
| Human Gata4 Reverse primer 5'-GGGAGACGCATAGCCTTGT-3' | This manuscript | N/A |
| Human Htra1 Forward primer 5'-TCCCAACAGTTTGCGCCATAA-3' | This manuscript | N/A |
| Human Htra1 Reverse primer 5'-CCGGCACCTCTCGTTTAGAAA-3' | This manuscript | N/A |
| Human Ifnb Forward primer 5'-AAACTCATGAGCAGTCTGCA-3' | This manuscript | N/A |
| Human Ifnb Reverse primer 5'-AGGAGATCTTCAGTTTCGGAGG-3' | This manuscript | N/A |
| Human Il13 Forward primer 5'-CAGTGCCATCGAGAAGACCCAGAG-3' | This manuscript | N/A |
| Human Il13 Reverse primer 5'-TCCCTAACCCTCCTTCCCGCCTA-3' | This manuscript | N/A |
| Human Il1b Forward primer 5'-ATGATGGCTTATTACAGTGGCAA-3' | This manuscript | N/A |
| Human Il1b Reverse primer 5'-GTCGGAGATTCGTAGCTGGA-3' | This manuscript | N/A |
| Human Il6 Forward primer 5'-ACTCACCTCTTCAGAACGAATTG-3' | This manuscript | N/A |
| Human Il6 Reverse primer 5'-CCATCTTTGGAAGGTTCAGGTTG-3' | This manuscript | N/A |
| Human Il8 Forward primer 5'-TTTCAGGAATTGAATGGGTTTGC-3' | This manuscript | N/A |
| Human Il8 Reverse primer 5'-TGTGAGGTAAGATGGTGGCTAAT-3' | This manuscript | N/A |
| Human Mmp13 Forward primer 5'-GGTGACTGGCAAACTTGACGATA-3' | This manuscript | N/A |
| Human Mmp13 Reverse primer 5'-GGACCATTTAAGAGTTCGAGGGA-3' | This manuscript | N/A |
| Human Mmp3 Forward primer 5'-ACAAGGAGGCAGGCAAGACAGCA-3' | This manuscript | N/A |
| Human Mmp3 Reverse primer 5'-GCCACGCACAGCAACAGTAGGAT-3' | This manuscript | N/A |
| Human β-Actin Forward primer 5'-GTTGCTATCCAGGCTGTG-3' | This manuscript | N/A |
| Human β-Actin Reverse primer 5'-TGATCTTGATCTTCATTGTG-3' | This manuscript | N/A |
| Mouse Adra1b Forward primer 5'-ACATTGGGGTGCGATACTCTC-3' | This manuscript | N/A |
| Mouse Adra1b Reverse primer 5'-TTGGGCGCAGGTTCTTTCC-3' | This manuscript | N/A |
| Mouse Birc5-1275 Forward primer 5'-TATCAGGGCACTCGTTT-3' | This manuscript | N/A |
| Mouse Birc5-1275 Reverse primer 5'-CCCGTTTTCAGTGTCTC-3' | This manuscript | N/A |
| Mouse Birc5-1984 Forward primer 5'-TCCACTCCCAGAAGGC-3' | This manuscript | N/A |
| Mouse Birc5-1984 Reverse primer 5'-CCAAGACGACTCAAACG-3' | This manuscript | N/A |
| Mouse Birc5-459 Forward primer 5'-GGCTACTTCCTGTTGATT-3' | This manuscript | N/A |
| Mouse Birc5-459 Reverse primer 5'-CTCCTGGTTACTGTTGTTG-3' | This manuscript | N/A |
| Mouse Braf-1487 Forward primer 5'-ATACCCTGAAGACCAGC-3' | This manuscript | N/A |
| Mouse Braf-1487 Reverse primer 5'-CATAGAACTGTCTTGGAGC-3' | This manuscript | N/A |
| Mouse Braf-1926 Forward primer 5'-CACGACCGTGACATGC-3' | This manuscript | N/A |
| Mouse Braf-1926 Reverse primer 5'-CCACCTCTGGAACCC-3' | This manuscript | N/A |
| Mouse Braf-261 Forward primer 5'-GATGCTGATAAGGATGTTG-3' | This manuscript | N/A |
| Mouse Braf-261 Reverse primer 5'-GGCTGTCCTGGAACTT-3' | This manuscript | N/A |
| Mouse Ccl2 Forward primer 5'-CATCCACGTGTTGGCTCA-3' | This manuscript | N/A |
| Mouse Ccl2 Reverse primer 5'-GATCATCTTGCTGGTGAATGAGT-3' | This manuscript | N/A |
| Mouse Ccl5 Forward primer 5'-ATATGGCTCGGACACCACTC-3' | This manuscript | N/A |
| Mouse Ccl5 Reverse primer 5'-ACTGCAAGATTGGAGCACTTG-3' | This manuscript | N/A |
| Mouse Cdc6-1630 Forward primer 5'-CTAGCCCACCAGAATAAT-3' | This manuscript | N/A |
| Mouse Cdc6-1630 Reverse primer 5'-TCTACTTTGTCCTCCACC-3' | This manuscript | N/A |
| Mouse Cdc6-1902 Forward primer 5'-GACTTCGTGGTGGTCG-3' | This manuscript | N/A |
| Mouse Cdc6-1902 Reverse primer 5'-TCTACTTCAGCCTTCTCG-3' | This manuscript | N/A |
| Mouse Cdc6-879 Forward primer 5'-GCGATTGTGCTGTTGT-3' | This manuscript | N/A |
| Mouse Cdc6-879 Reverse primer 5'-GTGCTGAGATTAAAGGTGT-3' | This manuscript | N/A |
| Mouse Cdkn1a Forward primer 5'-TGAATACCGTGGGTGTCAAAGCA-3' | This manuscript | N/A |
| Mouse Cdkn1a Reverse primer 5'-AGACAGGGAGGGAGCCACAATAC-3' | This manuscript | N/A |
| Mouse Cdkn2a Forward primer 5'-GCTTCCTGGACACGCTGGTGGTGCT-3' | This manuscript | N/A |
| Mouse Cdkn2a Reverse primer 5'-AAGGCGGGCTGAGGCCGGATTTAG-3' | This manuscript | N/A |
| Mouse Chek2 Forward primer 5'-TGACAGTGCTTCCTGTTCACA-3' | This manuscript | N/A |
| Mouse Chek2 Reverse primer 5'-GAGCTGGACGAACCCTGATA-3' | This manuscript | N/A |
| Mouse Chek2-1665 Forward primer 5'-TGCCCAAAAGTCTCCT-3' | This manuscript | N/A |
| Mouse Chek2-1665 Reverse primer 5'-TCGGGTCTTGCTACCT-3' | This manuscript | N/A |
| Mouse Chek2-1938 Forward primer 5'-CTTTCTTACTGGTCCTGC-3' | This manuscript | N/A |
| Mouse Chek2-1938 Reverse primer 5'-TTCCCACTTATGACTCACC-3' | This manuscript | N/A |
| Mouse Chek2-700 Forward primer 5'-AGCCCAGAGTCTCATCA-3' | This manuscript | N/A |
| Mouse Chek2-700 Reverse primer 5'-ATAGGGAACATCTCATCG-3' | This manuscript | N/A |
| Mouse Cxcl1 Forward primer 5'-TCGGCTGTGGTTCAGTTGT-3' | This manuscript | N/A |
| Mouse Cxcl1 Reverse primer 5'-GACTCCAGCCACACTCCAAC-3' | This manuscript | N/A |
| Mouse Cxcl10 Forward primer 5'-CCAAGTGCTGCCGTCATTTTC-3' | This manuscript | N/A |
| Mouse Cxcl10 Reverse primer 5'-GGCTCGCAGGGATGATTTCAA-3' | This manuscript | N/A |
| Mouse Fzr1-1302 Forward primer 5'-GAGTAACCAGCAGCGTC-3' | This manuscript | N/A |
| Mouse Fzr1-1302 Reverse primer 5'-CTCCTGTTTGTGGAAGAA-3' | This manuscript | N/A |
| Mouse Fzr1-138 Forward primer 5'-GACTAAAGGGGCATCC-3' | This manuscript | N/A |
| Mouse Fzr1-138 Reverse primer 5'-CCATAGCCAGAGCAAG-3' | This manuscript | N/A |
| Mouse Fzr1-1983 Forward primer 5'-GGATTCCCGTGAGCA-3' | This manuscript | N/A |
| Mouse Fzr1-1983 Reverse primer 5'-TCACCCGTGTCCTCA-3' | This manuscript | N/A |
| Mouse Gapdh Forward primer 5'-TGGCCTTCCGTGTTCCTAC-3' | This manuscript | N/A |
| Mouse Gapdh Reverse primer 5'-GAGTTGCTGTTGAAGTCGCA-3' | This manuscript | N/A |
| Mouse Gata4 Forward primer 5'-CACCCCAATCTCGATATGTTTGA-3' | This manuscript | N/A |
| Mouse Gata4 Reverse primer 5'-GCACAGGTAGTGTCCCGTC-3' | This manuscript | N/A |
| Mouse Hdac6 Forward primer 5'-TCCACCGGCCAAGATTCTTC-3' | This manuscript | N/A |
| Mouse Hdac6 Reverse primer 5'-CAGCACACTTCTTTCCACCAC-3' | This manuscript | N/A |
| Mouse Htra1 Forward primer 5'-TAGCGACGCCAAGACCTACA-3' | This manuscript | N/A |
| Mouse Htra1 Reverse primer 5'-TGACGCAAACTGTTGGGATCT-3' | This manuscript | N/A |
| Mouse Ifit3 Forward primer 5'-GAGGAGAAGGCAACAAT-3' | This manuscript | N/A |
| Mouse Ifit3 Reverse primer 5'-AGTAGATCCAGGCGTAGT-3' | This manuscript | N/A |
| Mouse Ifit3b Forward primer 5'-GAGGAGAAGGCAACAAT-3' | This manuscript | N/A |
| Mouse Ifit3b Reverse primer 5'-AGTAGATCCAGGCGTAGT-3' | This manuscript | N/A |
| Mouse Ifnb Forward primer 5'-CAGCTCCAAGAAAGGACGAAC-3' | This manuscript | N/A |
| Mouse Ifnb Reverse primer 5'-GGCAGTGTAACTCTTCTGCAT-3' | This manuscript | N/A |
| Mouse Il13 Forward primer 5'-GATTCCCTGACCAACATCTCCAA-3' | This manuscript | N/A |
| Mouse Il13 Reverse primer 5'-ATCTCCCTTCCTCCTCAACCCTC-3' | This manuscript | N/A |
| Mouse Il1b Forward primer 5'-GAAATGCCACCTTTTGACAGTG-3' | This manuscript | N/A |
| Mouse Il1b Reverse primer 5'-TGGATGCTCTCATCAGGACAG-3' | This manuscript | N/A |
| Mouse Il6 Forward primer 5'-GGAGCCCACCAAGAACGATAGTCAA-3' | This manuscript | N/A |
| Mouse Il6 Reverse primer 5'-GTCACCAGCATCAGTCCCAAGAA-3' | This manuscript | N/A |
| Mouse Il8 Forward primer 5'-GGCTTTGCGTTGATTCTGGGAACT-3' | This manuscript | N/A |
| Mouse Il8 Reverse primer 5'-AGCGGTGTCCTGATTATCGTCCT-3' | This manuscript | N/A |
| Mouse Klhdc8b Forward primer 5'-CGACTTGCCGGGTATATGGTA-3' | This manuscript | N/A |
| Mouse Klhdc8b Reverse primer 5'-CCAGAGTCTCAGCAGTATCCAG-3' | This manuscript | N/A |
| Mouse Mapk11-1306 Forward primer 5'-TGTGGGGACCATACTTG-3' | This manuscript | N/A |
| Mouse Mapk11-1306 Reverse primer 5'-TTGCCCTAACCTCGA-3' | This manuscript | N/A |
| Mouse Mapk11-1879 Forward primer 5'-AACCTCCACCCACCC-3' | This manuscript | N/A |
| Mouse Mapk11-1879 Reverse primer 5'-GCACCGCTGAGCAAA-3' | This manuscript | N/A |
| Mouse Mapk11-580 Forward primer 5'-GCCTTGAACTTGCTGTG-3' | This manuscript | N/A |
| Mouse Mapk11-580 Reverse primer 5'-TTATGGGTTGAGTGTCG-3' | This manuscript | N/A |
| Mouse Mmp13 Forward primer 5'-TCACCTGATTCTTGCGTGCTATG-3' | This manuscript | N/A |
| Mouse Mmp13 Reverse primer 5'-CTTTATCTGTGCTCATCTGTGGC-3' | This manuscript | N/A |
| Mouse Mmp3 Forward primer 5'-TTTGATGCAGTCAGCACCCTCCG-3' | This manuscript | N/A |
| Mouse Mmp3 Reverse primer 5'-TCGTGCCCTCGTATAGCCCAGAA-3' | This manuscript | N/A |
| Mouse Mrnip Forward primer 5'-ATTTGTTCGGGCTTACG-3' | This manuscript | N/A |
| Mouse Mrnip Reverse primer 5'-CCATTTACAGCTTCCTCC-3' | This manuscript | N/A |
| Mouse Ndrg1 Forward primer 5'-ATGTCCCGAGAGCTACATGAC-3' | This manuscript | N/A |
| Mouse Ndrg1 Reverse primer 5'-CCTGCTCCTGAACATCGAACT-3' | This manuscript | N/A |
| Mouse P19 Forward primer 5'-CTGGAAGAAGTCTGCGTCGG-3' | This manuscript | N/A |
| Mouse P19 Reverse primer 5'-GTCTTGCCAAAGCGGTTCAG-3' | This manuscript | N/A |
| Mouse P53 Forward primer 5'-GTCACAGCACATGACGGAGG-3' | This manuscript | N/A |
| Mouse P53 Reverse primer 5'-TCTTCCAGATGCTCGGGATAC-3' | This manuscript | N/A |
| Mouse Ppp6c Forward primer 5'-CCGCTGGATCTGGACAAGTAT-3' | This manuscript | N/A |
| Mouse Ppp6c Reverse primer 5'-ACACTGGCTGAACATTCGACT-3' | This manuscript | N/A |
| Mouse Rarg Forward primer 5'-CTGAGAGATTCAAGGCAAGAGG-3' | This manuscript | N/A |
| Mouse Rarg Reverse primer 5'-GAACGCGCTATCTTACCCCAG-3' | This manuscript | N/A |
| Mouse Rps6ka1 Forward primer 5'-CCATCACACACCACGTCAAG-3' | This manuscript | N/A |
| Mouse Rps6ka1 Reverse primer 5'-TTGCGTACCAGGAAGACTTTG-3' | This manuscript | N/A |
| Mouse Sfn Forward primer 5'-GTGTGTGCGACACCGTACT-3' | This manuscript | N/A |
| Mouse Sfn Reverse primer 5'-CTCGGCTAGGTAGCGGTAG-3' | This manuscript | N/A |
| Mouse Tcf3-1828 Forward primer 5'-TGTTGTGAGGTGGTGTATG-3' | This manuscript | N/A |
| Mouse Tcf3-1828 Reverse primer 5'-ACTGTCGCCAAAAGC-3' | This manuscript | N/A |
| Mouse Tcf3-1926 Forward primer 5'-TTTGGCGACAGTGGG-3' | This manuscript | N/A |
| Mouse Tcf3-1926 Reverse primer 5'-GCTGCCTCATCTTCCTG-3' | This manuscript | N/A |
| Mouse Tcf3-541 Forward primer 5'-ACCTAGAGGTGCGATTC-3' | This manuscript | N/A |
| Mouse Tcf3-541 Reverse primer 5'-AGGCAGCAAAGAGGAT-3' | This manuscript | N/A |
| Mouse Wbp2 Forward primer 5'-ATGGCGCTCAACAAGAATCAC-3' | This manuscript | N/A |
| Mouse Wbp2 Reverse primer 5'-AGTAAGGTAGACGGTGCCTTT-3' | This manuscript | N/A |
| Mouse Wbp2-1854 Forward primer 5'-TTGTAGTTGGCTGCTTG-3' | This manuscript | N/A |
| Mouse Wbp2-1854 Reverse primer 5'-GGCGTCGTTCGTATT-3' | This manuscript | N/A |
| Mouse Wbp2-1937 Forward primer 5'-ATCAGGTGGTTGGTCAG-3' | This manuscript | N/A |
| Mouse Wbp2-1937 Reverse primer 5'-AGACTCGCAACGCTCT-3' | This manuscript | N/A |
| Mouse Wbp2-889 Forward primer 5'-CCCAAAGCGAACAGA-3' | This manuscript | N/A |
| Mouse Wbp2-889 Reverse primer 5'-GGAACATTGCCACAGA-3' | This manuscript | N/A |
| Mouse Wnk1-1781 Forward primer 5'-CACCAGGCTAGAGCAA-3' | This manuscript | N/A |
| Mouse Wnk1-1781 Reverse primer 5'-AGGCACGATGGGATT-3' | This manuscript | N/A |
| Mouse Wnk1-1977 Forward primer 5'-AGGCTTCAGACATCGG-3' | This manuscript | N/A |
| Mouse Wnk1-1977 Reverse primer 5'-AGCAGTCACCCCACTAG-3' | This manuscript | N/A |
| Mouse Wnk1-507 Forward primer 5'-GGTAGATTTCGGCAAGA-3' | This manuscript | N/A |
| Mouse Wnk1-507 Reverse primer 5'-AAGCGGCTAAACGGA-3' | This manuscript | N/A |
| Mouse β-Actin Forward primer 5'-GTCCCTCACCCTCCCAAAAG-3' | This manuscript | N/A |
| Mouse β-Actin Reverse primer 5'-GCTGCCTCAACACCTCAACCC-3' | This manuscript | N/A |
| siGata4 Sense 5'-GUCCCAGACAUUCAGUACUTT-3' | This manuscript | N/A |
| siGata4 Antisense 5'-AGUACUGAAUGUCUGGGACTT-3' | This manuscript | N/A |
| siHDAC6 Sense 5'-GGCCAGGCUAAAUCAAAGATT-3' | This manuscript | N/A |
| siHDAC6 Antisense 5'-UCUUUGAUUUAGCCUGGCCTT-3' | This manuscript | N/A |
| siHtrA1 Sense 5'-UCGACAGGCCAAAGGGAAATT-3' | This manuscript | N/A |
| siHtrA1 Antisense 5'-UUUCCCUUUGGCCUGUCGATT-3 | This manuscript | N/A |
| siSGK1 Sense 5'-CUCCAGUUGAAACCAAAUATT-3' | This manuscript | N/A |
| siSGK1 Antisense 5'-UAUUUGGUUUCAACUGGAGTT -3 | This manuscript | N/A |

**Table S4: Lists of antibodies.**

| REAGENT or RESOURCE | SOURCE | IDENTIFIER | DILUTION |
| --- | --- | --- | --- |
| Alexa Fluor 488 donkey anti-goat IgG（H+L） | Thermo Fisher Scientific | Cat# A11055; RRID:AB_2534102 | 1:1000 |
| Alexa Fluor 488 donkey anti-mouse IgG（H+L） | Thermo Fisher Scientific | Cat# A21202; RRID:AB_141607 | 1:1000 |
| Alexa Fluor 488 donkey anti-rabbit IgG（H+L） | Thermo Fisher Scientific | Cat# A21206; RRID:AB_2535792 | 1:1000 |
| Alexa Fluor 488 donkey anti-rat IgG（H+L） | Thermo Fisher Scientific | Cat# A21208; RRID:AB_2535794 | 1:1000 |
| Alexa Fluor 546 donkey anti-goat IgG（H+L） | Thermo Fisher Scientific | Cat# A11056; RRID:AB_2534103 | 1:1000 |
| Alexa Fluor 546 donkey anti-mouse IgG（H+L） | Thermo Fisher Scientific | Cat# A10036; RRID:AB_2534012 | 1:1000 |
| Alexa Fluor 546 donkey anti-rabbit IgG（H+L） | Thermo Fisher Scientific | Cat# A10040; RRID:AB_2534016 | 1:1000 |
| Alexa Fluor 647 donkey anti-goat IgG（H+L） | Thermo Fisher Scientific | Cat# A21447; RRID:AB_2535864 | 1:1000 |
| Alexa Fluor 647 donkey anti-mouse IgG（H+L） | Thermo Fisher Scientific | Cat# A21235; RRID:AB_2535804 | 1:1000 |
| Alexa Fluor 647 donkey anti-rabbit IgG（H+L） | Thermo Fisher Scientific | Cat# A31573; RRID:AB_2536183 | 1:1000 |
| CDK2 | Proteintech | Cat# 10122-1-AP; RRID:AB_2078556 | 1:1000 |
| CDK2 pT160 | CST | Cat# 2561S; | 1:1000 |
| CDK2 pY15 | Thermo Fisher Scientific | Cat# MA5-41262; RRID:AB_2899015 | 1:1000 |
| CDKN2A/p16 | Santa | Cat# sc-1661 | 1:100 |
| FLAG | CST | Cat# 14793S | 1:1000 |
| GAPDH | Abcam | Cat# ab8245; RRID:AB_2107448 | 1:10000 |
| GATA4 | Santa | Cat# sc-25310; RRID:AB_627667 | 1:200 |
| GLB1 | GeneTex | Cat# GTX134513; RRID:AB_2887300 | 1:500 |
| HDAC6 | LSBio | Cat# LS-C746922-100 | 1:1000 |
| HDAC6 | Novus Biologicals | Cat# NB100-56343; RRID:AB_838335 | 1:1000 |
| HDAC6 (D2E5) | CST | Cat# 7558T | 1:1000 |
| His | Raybiotech | Cat# RB-10-0002-100 | 1:1000 |
| Histone H2A.X | Santa | Cat# sc-517336; RRID:AB_3675923 | 1:100 |
| HRP-labeled Goat Anti-Mouse IgG(H+L) | ZSGB-BIO | Cat# ZB-2305; RRID:AB_2747415 | 1:10000 |
| HRP-labeled Goat Anti-Rabbit IgG(H+L) | ZSGB-BIO | Cat# ZB-2301; RRID:AB_2747412 | 1:10000 |
| HtrA1 | Proteintech | Cat# 55011-1-AP; RRID:AB_10859830 | 1:1000 |
| HtrA1 | Origen | Cat# TA324517 | 1:1000 |
| iNOS | Abcam | Cat# ab178945; RRID:AB_2861417 | 1:1000 |
| Lamin B1 | Abcam | Cat# ab16048; RRID:AB_443298 | 1:2000 |
| LC3B | Sigma-aldrich | Cat# L7543; RRID:AB_796155 | 1:3000 |
| MYO7A | DSHB | Cat# MYO7A 138-1; RRID:AB_2282417 | 1:100 |
| NF-kB p65 (phospho S536) | Abcam | Cat# ab76302; RRID:AB_1524028 | 1:500 |
| NF-κB p65 | CST | Cat# 8242 | 1:1000 |
| Peroxidase AffiniPure Goat Anti-Mouse IgG (H+L) | Jackson | Cat# 115-035-003; RRID:AB_10015289 | 1:10000 |
| Peroxidase AffiniPure Goat Anti-Rabbit IgG (H+L) | Jackson | Cat# 111-035-003; RRID:AB_2313567 | 1:10000 |
| SDHA | Origene | Cat# TA350393 | 1:3000 |
| SGK1 | ABGENT | Cat# AP3924A-ev | 1:1000 |
| SOD2 | Genetex | Cat# GTX116093; RRID:AB_10624558 | 1:2000 |
| SQSTM1/p62 | Abcam | Cat# ab109012; RID:AB_2810880 | 1:2000 |
| TBP | Proteintech | Cat# 22006-1-AP; RRID:AB_10951514 | 1:500 |
| Waf1/Cip1/CDKN1A p21 | Santa | Cat# sc-6246; RRID:AB_628073 | 1:100 |
| β-actin | CST | Cat# 3700S; RRID:AB_2242334 | 1:10000 |

**Data files S1 to S4**

**Data file S1. The identified Protein in MS**

**Data file S2. The differentially expressed genes in RNA-seq**

**Data file S3. Genes related to cell cycle**

**Data file S4. The binding peaks in ChIP-seq**
