# Supplementary figures and images for: GATA4‐Driven Transcription of HtrA1 Promotes Cellular Senescence in Ménière's Disease and Age‐Related Audio‐Vestibular Dysfunction (part 1 of 2)
Source: Adv Sci (Weinh). 2026 Apr 14;13(39):e12538. doi: 10.1002/advs.202512538 (PMC13334991; doi:10.1002/advs.202512538)

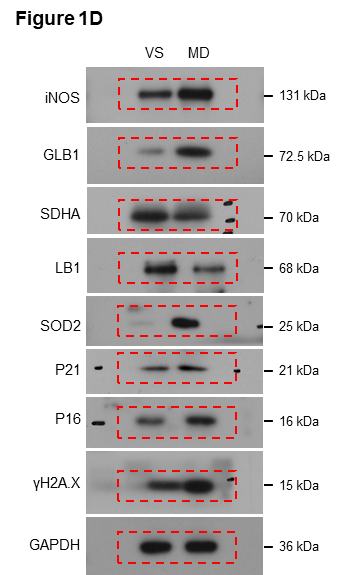

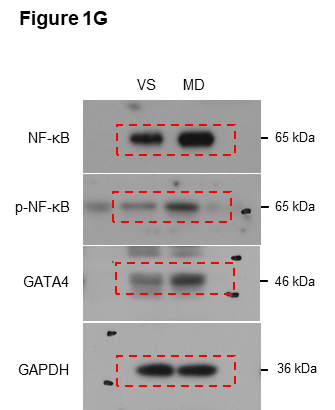


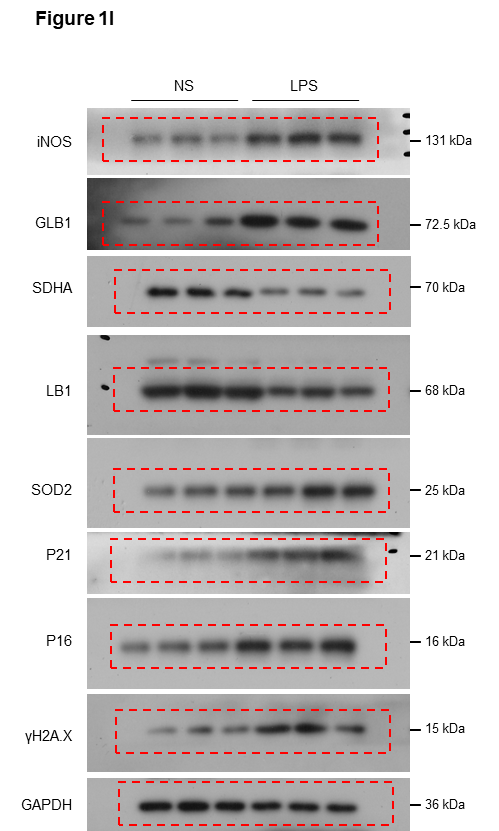

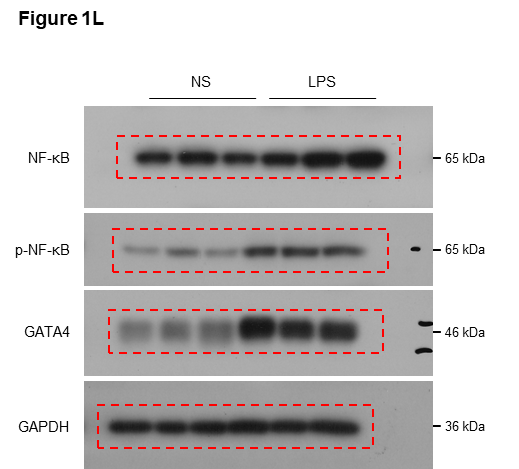

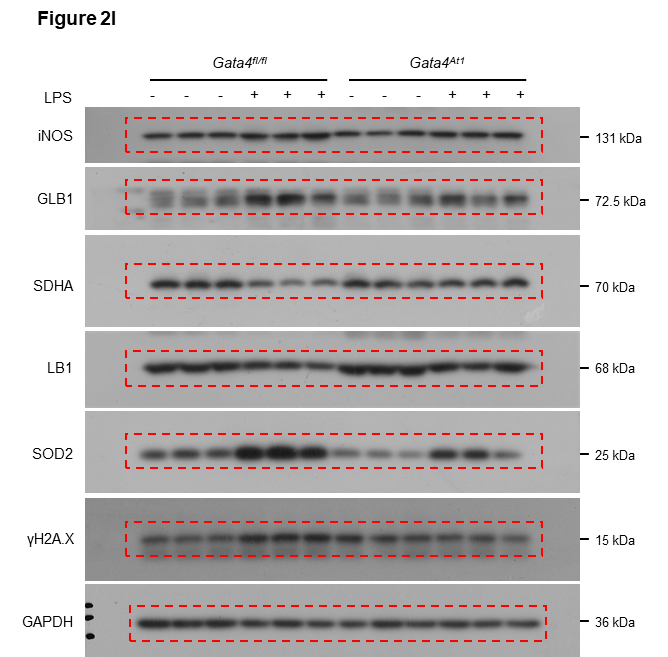


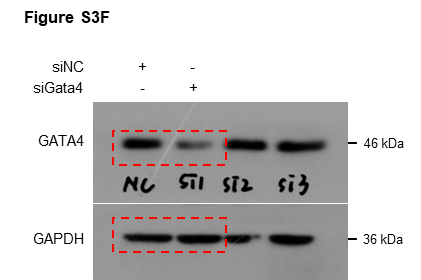


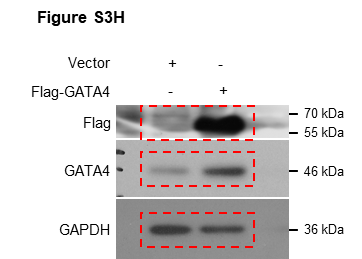


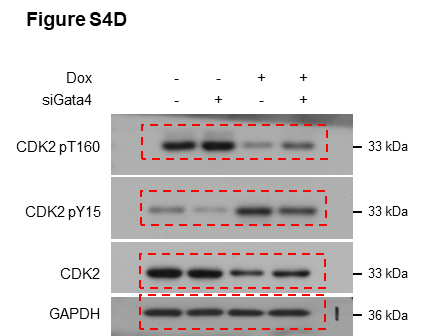

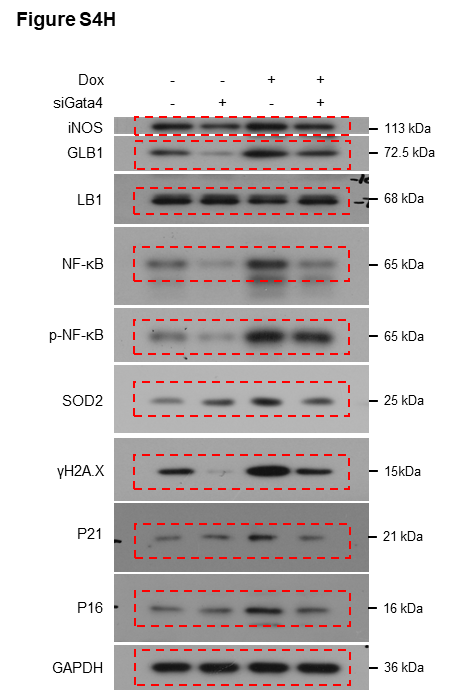


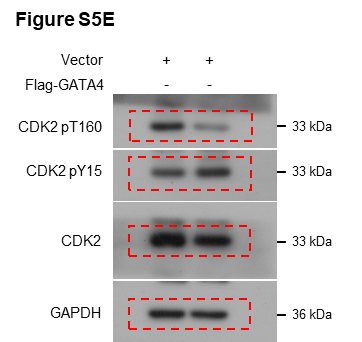

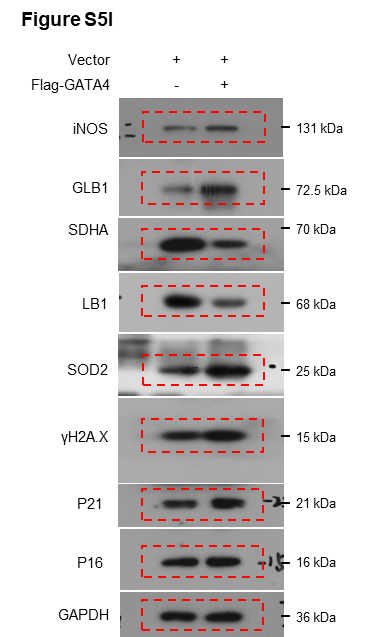


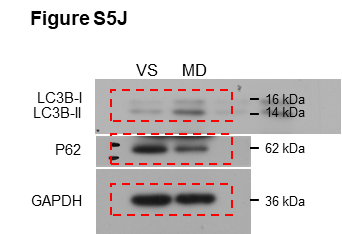

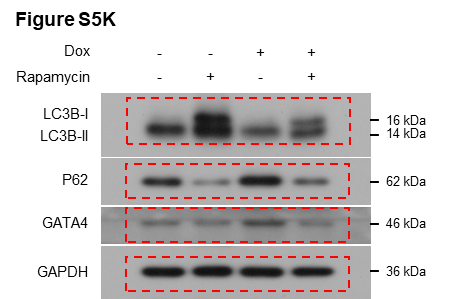


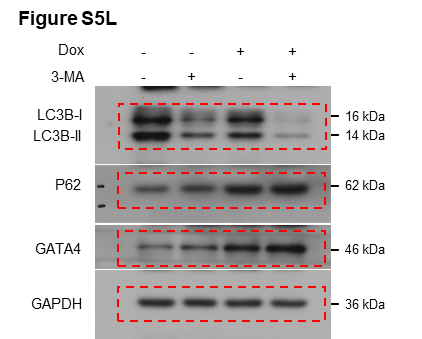


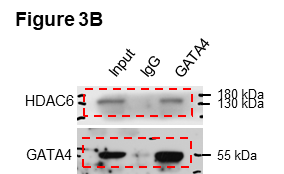

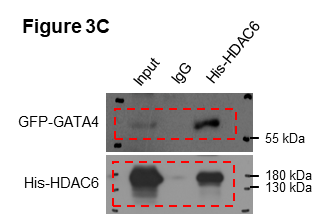

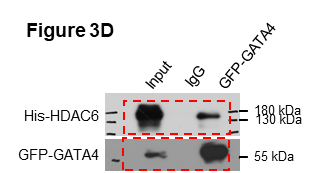


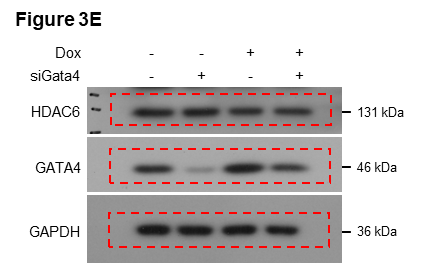

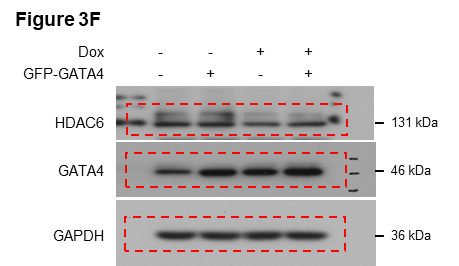


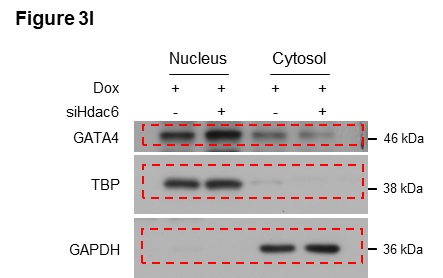

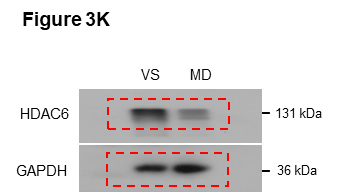


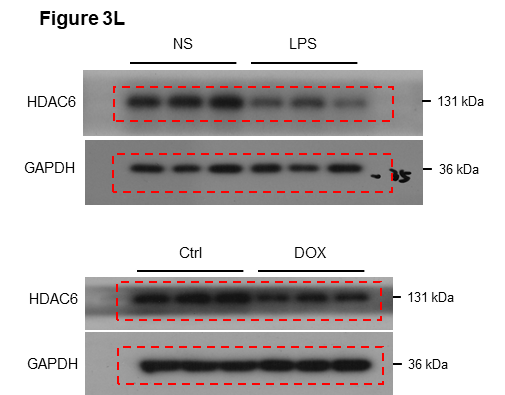

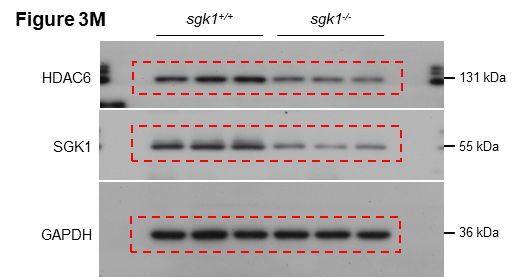


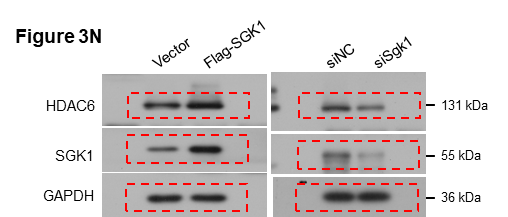

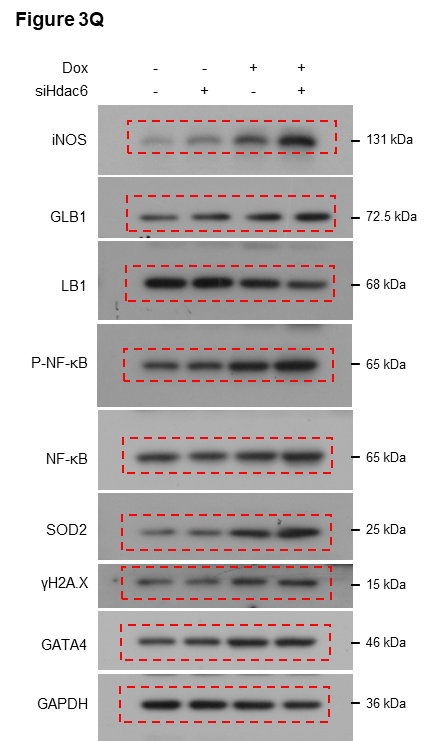


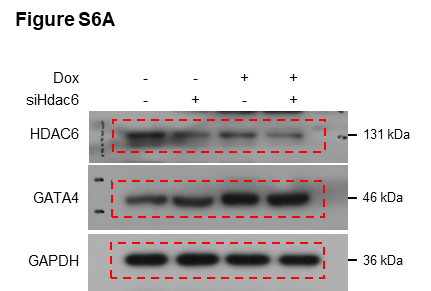


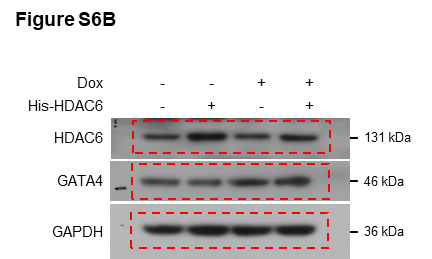


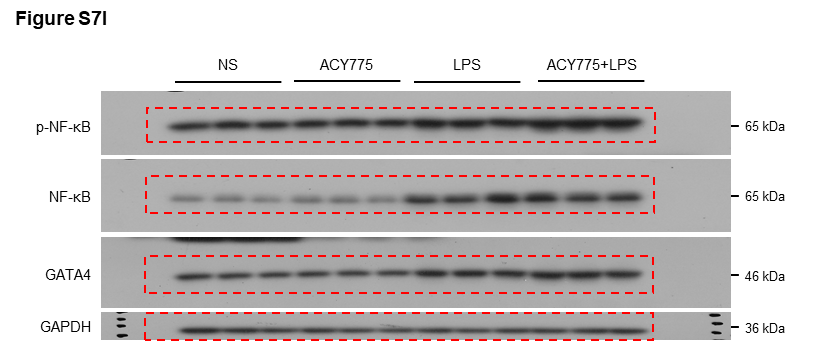


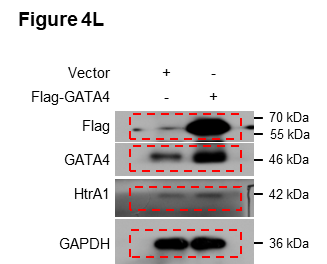


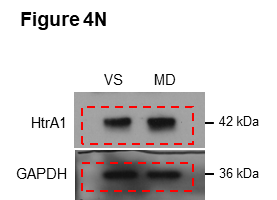


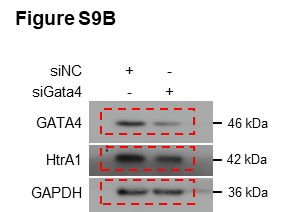


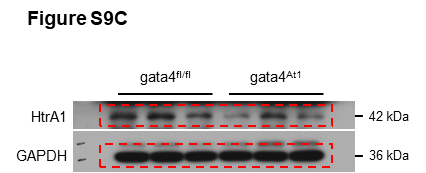


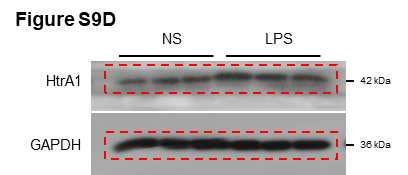


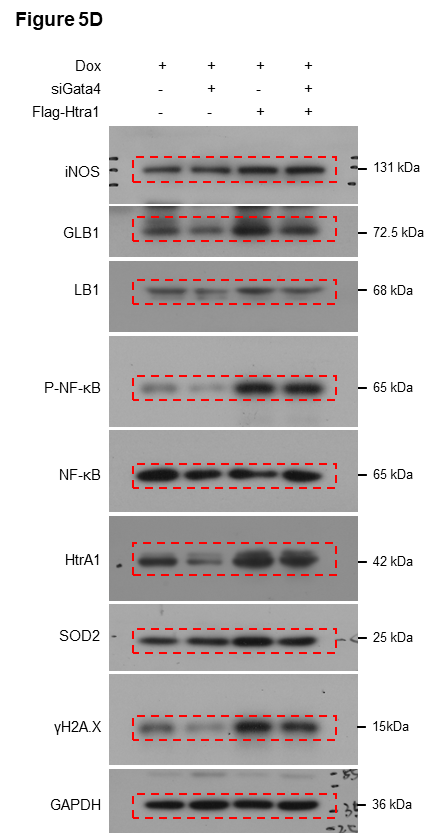


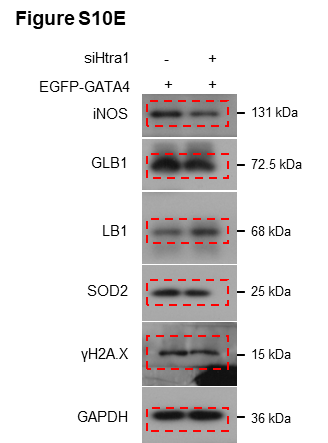


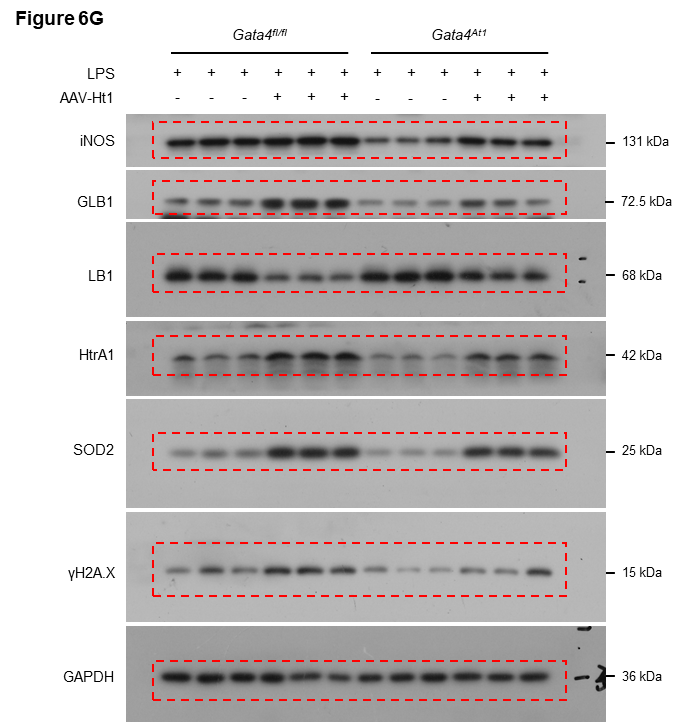


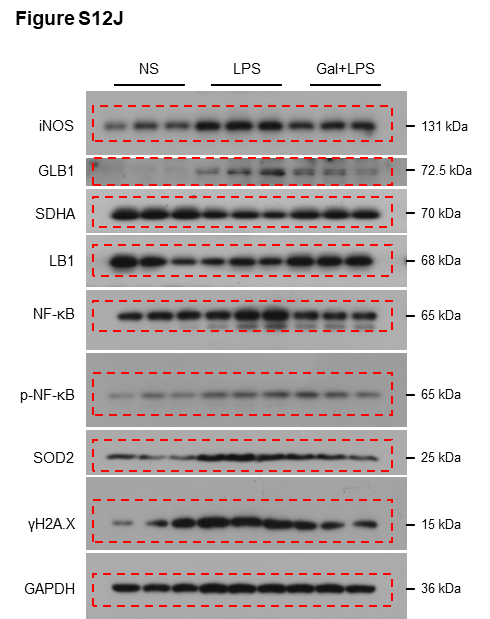


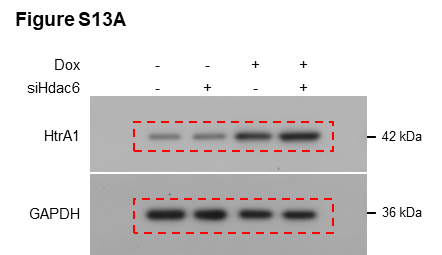


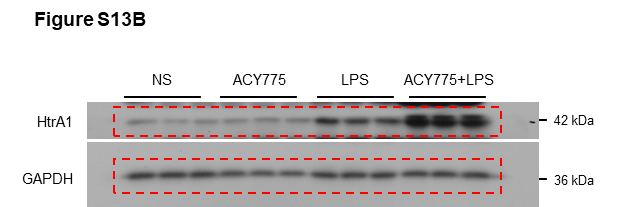


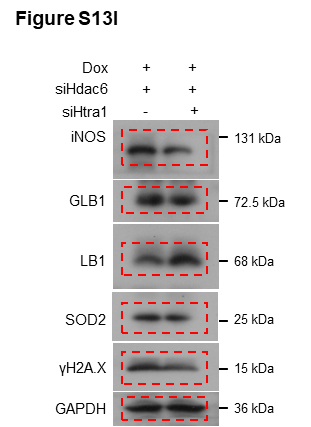


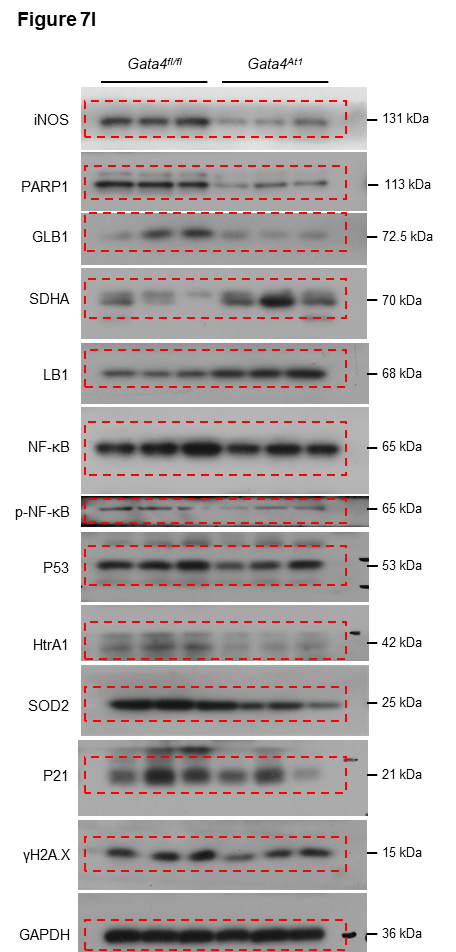

Supplement: Supplementary file 2 — Supporting File 2: advs75263‐sup‐0002‐FigureS1.docx. [file ADVS-13-e12538-s006.docx]

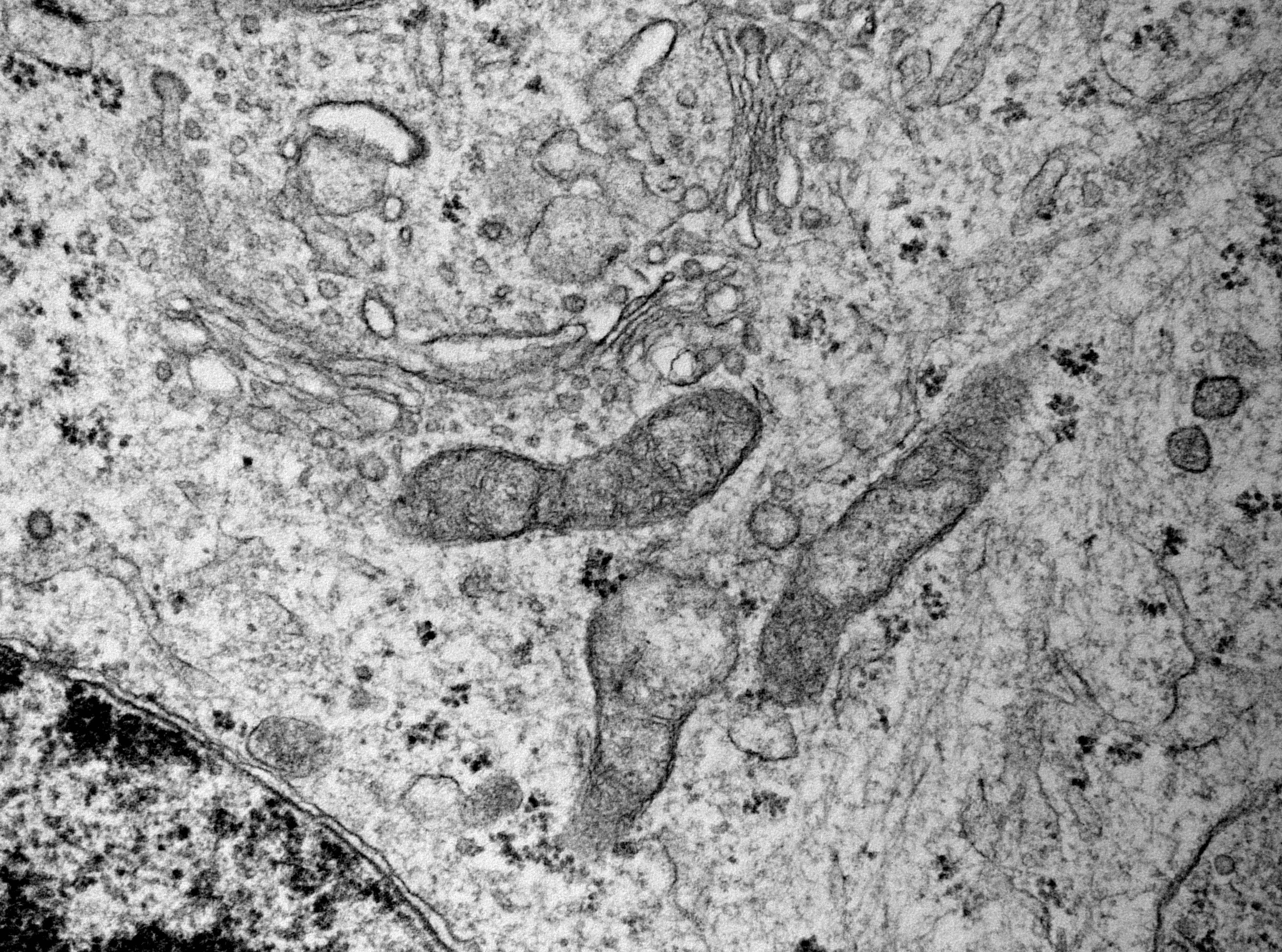

Supplement: Supplementary file 7 — Supporting File 7: advs75263‐sup‐0007‐Data5.zip. [file ADVS-13-e12538-s004.zip › Raw data of microscope images/Figure 1A-MD 2.jpg]

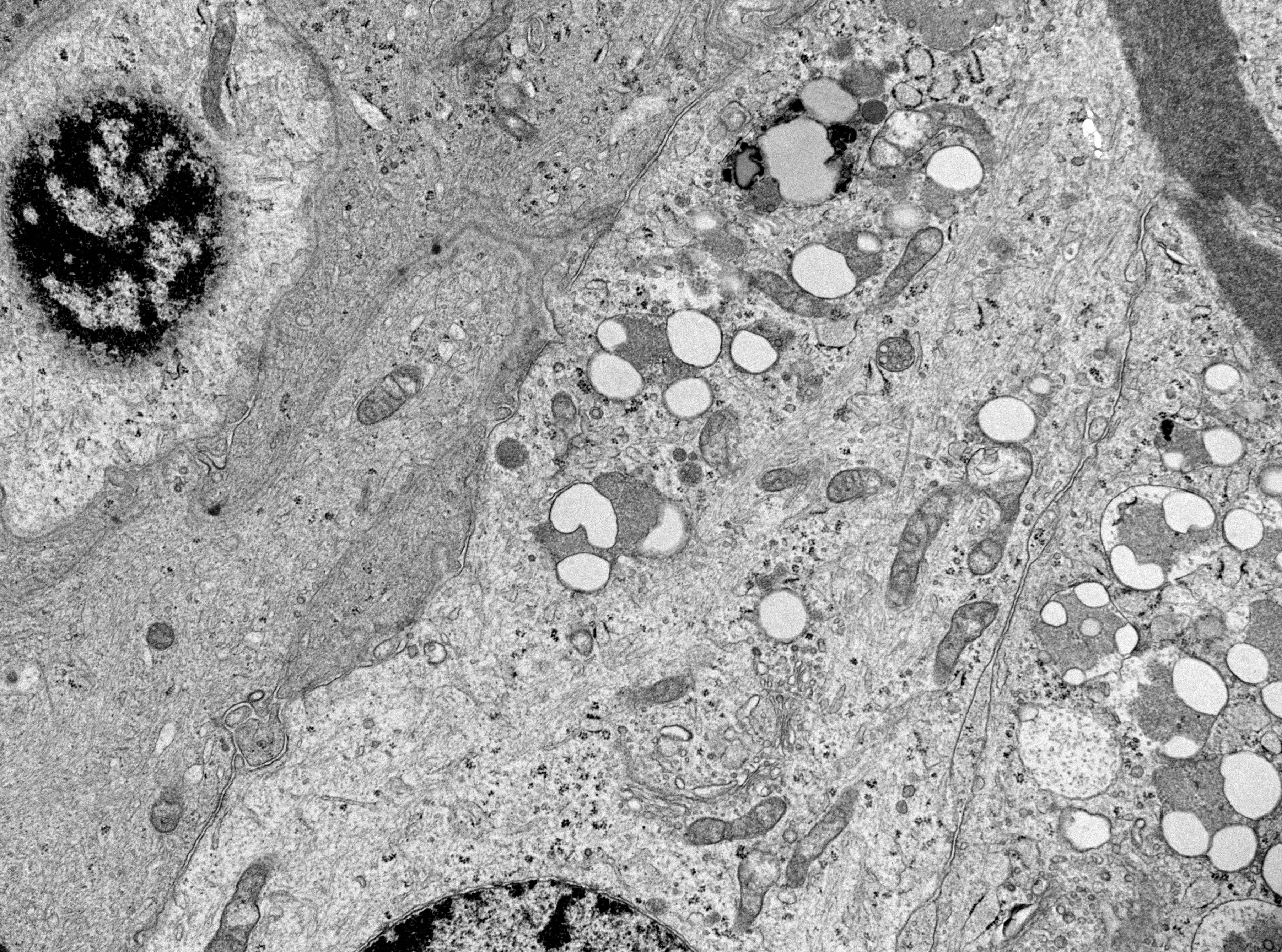

Supplement: Supplementary file 7 — Supporting File 7: advs75263‐sup‐0007‐Data5.zip. [file ADVS-13-e12538-s004.zip › Raw data of microscope images/Figure 1A-MD.jpg]

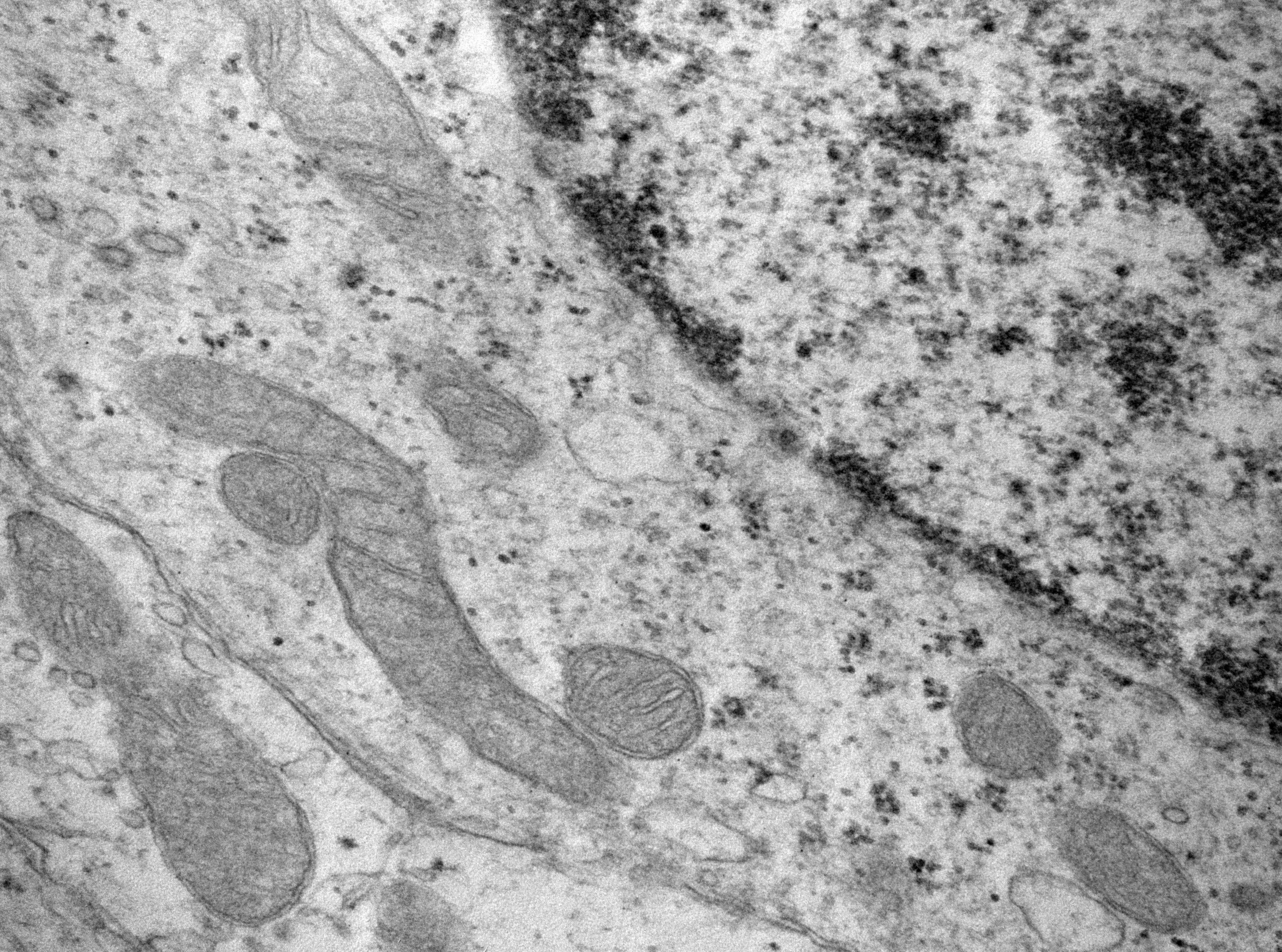

Supplement: Supplementary file 7 — Supporting File 7: advs75263‐sup‐0007‐Data5.zip. [file ADVS-13-e12538-s004.zip › Raw data of microscope images/Figure 1A-VS 2.jpg]

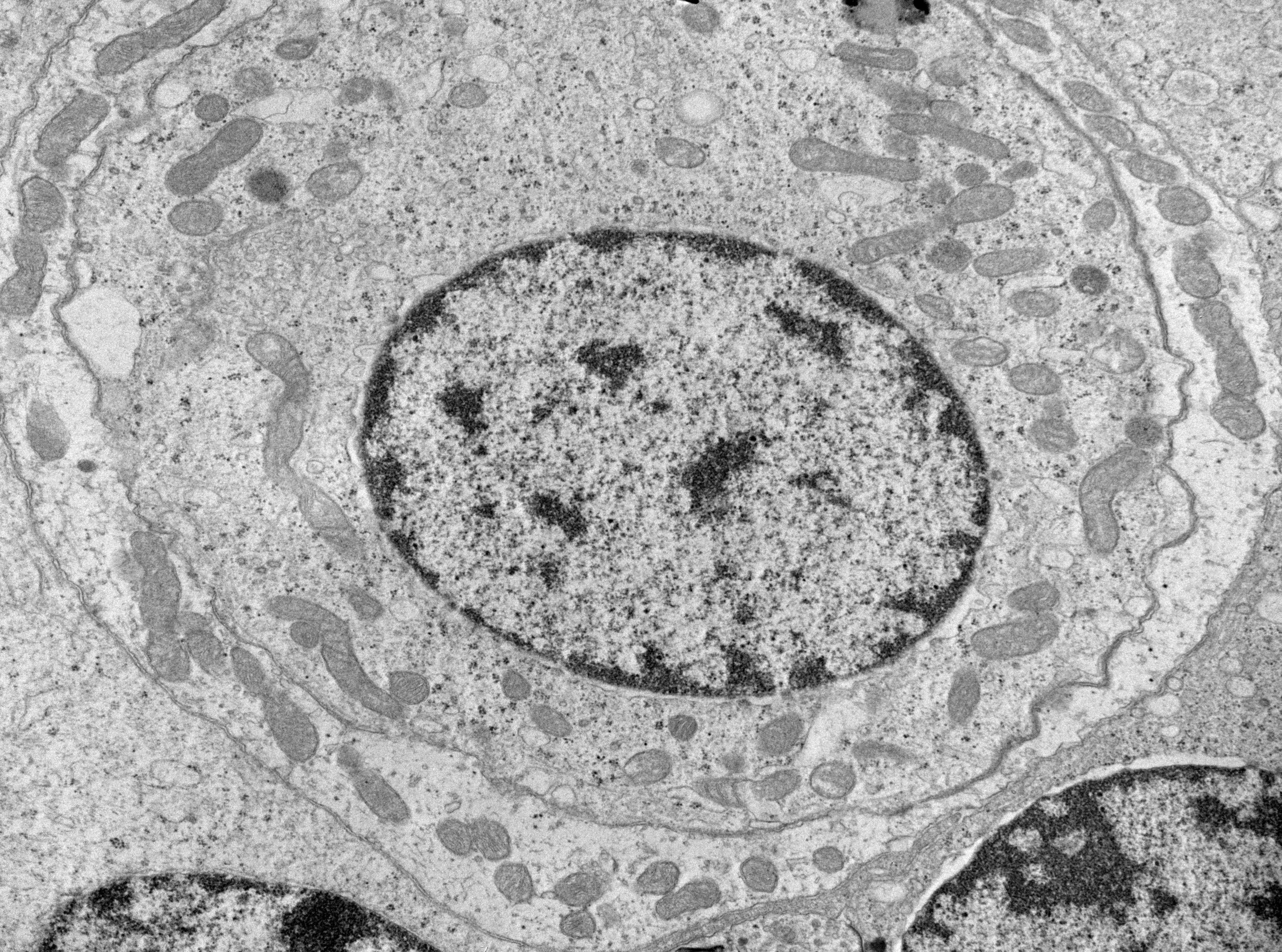

Supplement: Supplementary file 7 — Supporting File 7: advs75263‐sup‐0007‐Data5.zip. [file ADVS-13-e12538-s004.zip › Raw data of microscope images/Figure 1A-VS.jpg]

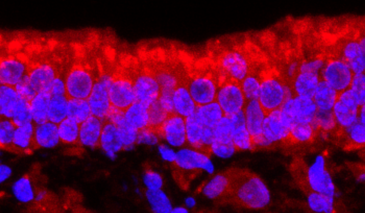

Supplement: Supplementary file 7 — Supporting File 7: advs75263‐sup‐0007‐Data5.zip. [file ADVS-13-e12538-s004.zip › Raw data of microscope images/Figure 1B-MD DHE.tif]

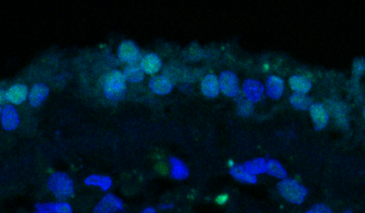

Supplement: Supplementary file 7 — Supporting File 7: advs75263‐sup‐0007‐Data5.zip. [file ADVS-13-e12538-s004.zip › Raw data of microscope images/Figure 1B-MD γH2AX.tif]

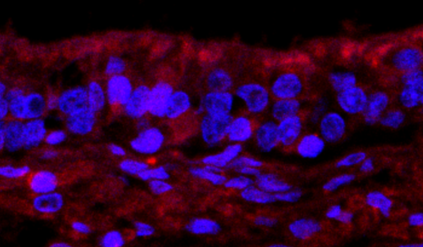

Supplement: Supplementary file 7 — Supporting File 7: advs75263‐sup‐0007‐Data5.zip. [file ADVS-13-e12538-s004.zip › Raw data of microscope images/Figure 1B-VS DHE.tif]

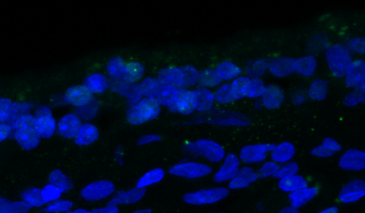

Supplement: Supplementary file 7 — Supporting File 7: advs75263‐sup‐0007‐Data5.zip. [file ADVS-13-e12538-s004.zip › Raw data of microscope images/Figure 1B-VS γH2AX.tif]

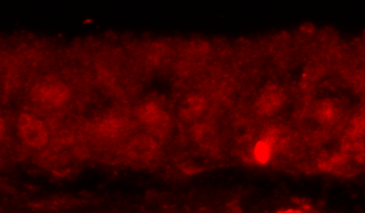

Supplement: Supplementary file 7 — Supporting File 7: advs75263‐sup‐0007‐Data5.zip. [file ADVS-13-e12538-s004.zip › Raw data of microscope images/Figure 1F-MD (1) GATA4.tif]

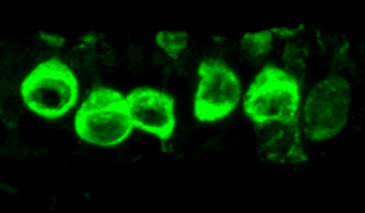

Supplement: Supplementary file 7 — Supporting File 7: advs75263‐sup‐0007‐Data5.zip. [file ADVS-13-e12538-s004.zip › Raw data of microscope images/Figure 1F-MD (2) Myo7a.tif]

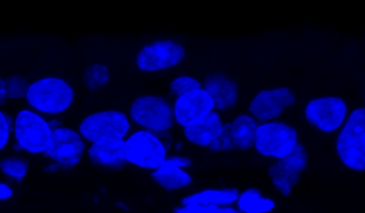

Supplement: Supplementary file 7 — Supporting File 7: advs75263‐sup‐0007‐Data5.zip. [file ADVS-13-e12538-s004.zip › Raw data of microscope images/Figure 1F-MD (3) DAPI.tif]

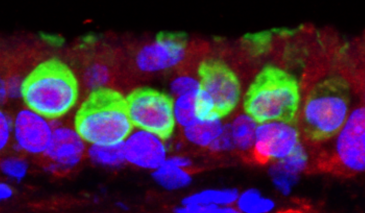

Supplement: Supplementary file 7 — Supporting File 7: advs75263‐sup‐0007‐Data5.zip. [file ADVS-13-e12538-s004.zip › Raw data of microscope images/Figure 1F-MD (4) Merge.tif]

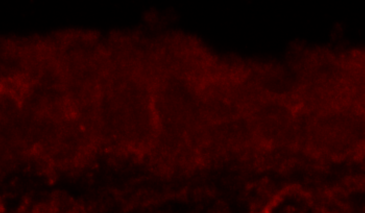

Supplement: Supplementary file 7 — Supporting File 7: advs75263‐sup‐0007‐Data5.zip. [file ADVS-13-e12538-s004.zip › Raw data of microscope images/Figure 1F-VS (1) GATA4.tif]

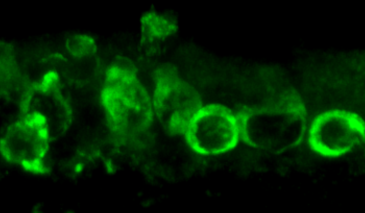

Supplement: Supplementary file 7 — Supporting File 7: advs75263‐sup‐0007‐Data5.zip. [file ADVS-13-e12538-s004.zip › Raw data of microscope images/Figure 1F-VS (2) Myo7a.tif]

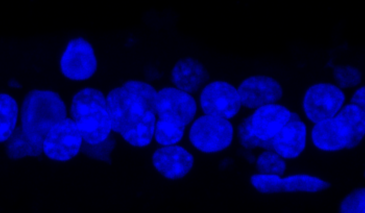

Supplement: Supplementary file 7 — Supporting File 7: advs75263‐sup‐0007‐Data5.zip. [file ADVS-13-e12538-s004.zip › Raw data of microscope images/Figure 1F-VS (3) DAPI.tif]

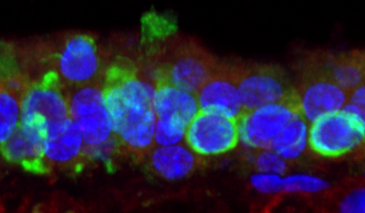

Supplement: Supplementary file 7 — Supporting File 7: advs75263‐sup‐0007‐Data5.zip. [file ADVS-13-e12538-s004.zip › Raw data of microscope images/Figure 1F-VS (4) Merge.tif]

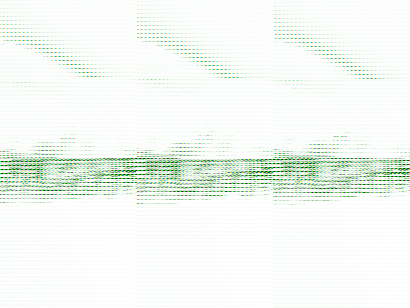

Supplement: Supplementary file 7 — Supporting File 7: advs75263‐sup‐0007‐Data5.zip. [file ADVS-13-e12538-s004.zip › Raw data of microscope images/Figure 1K-LPSS.tif]

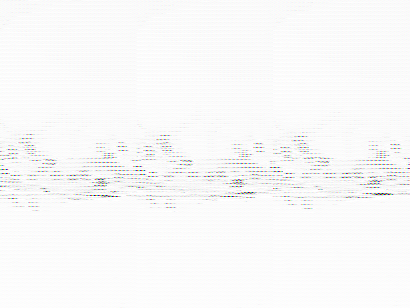

Supplement: Supplementary file 7 — Supporting File 7: advs75263‐sup‐0007‐Data5.zip. [file ADVS-13-e12538-s004.zip › Raw data of microscope images/Figure 1K-NS.tif]

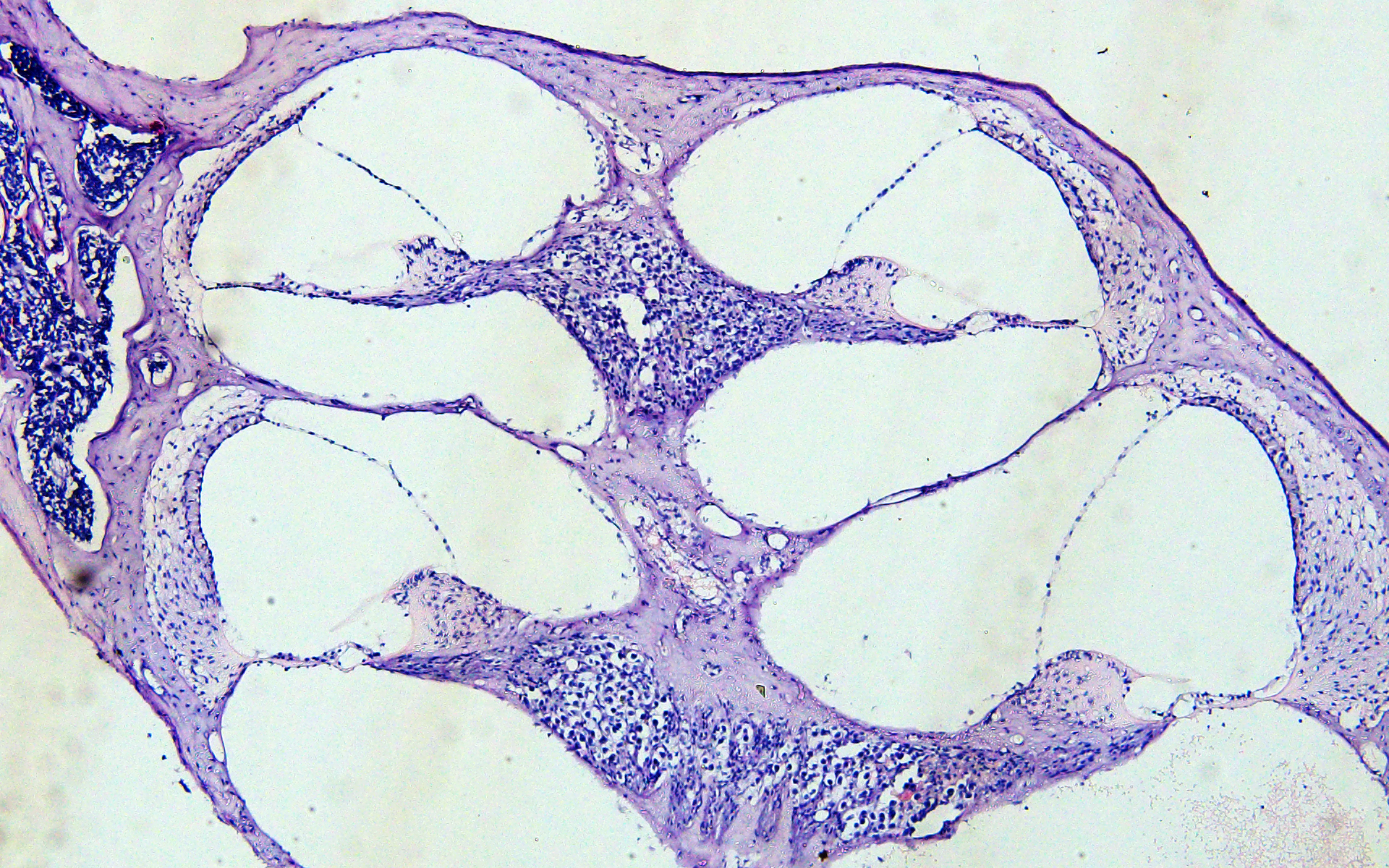

Supplement: Supplementary file 7 — Supporting File 7: advs75263‐sup‐0007‐Data5.zip. [file ADVS-13-e12538-s004.zip › Raw data of microscope images/Figure 2B-KO LPS.tif]

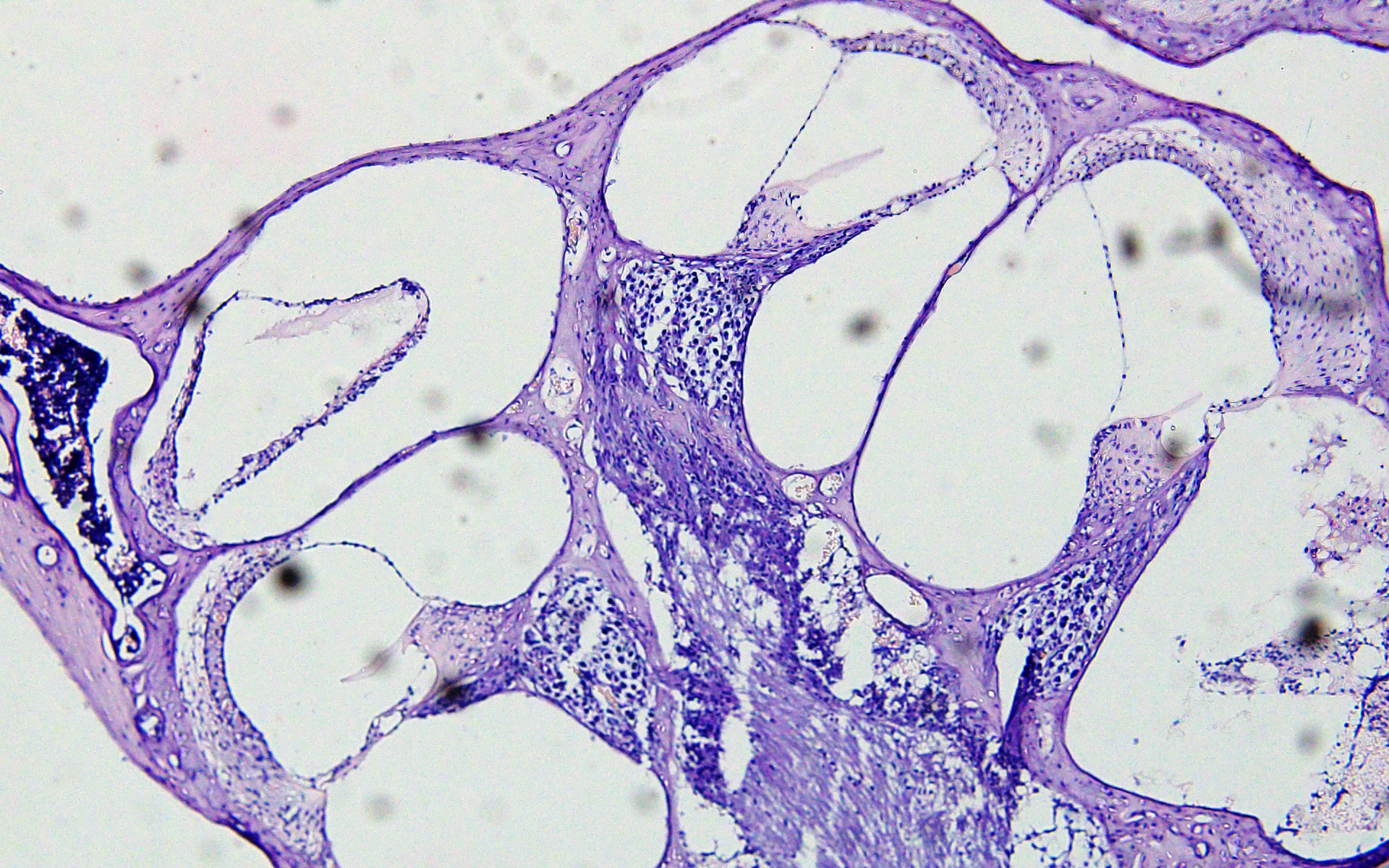

Supplement: Supplementary file 7 — Supporting File 7: advs75263‐sup‐0007‐Data5.zip. [file ADVS-13-e12538-s004.zip › Raw data of microscope images/Figure 2B-KO NS.tif]

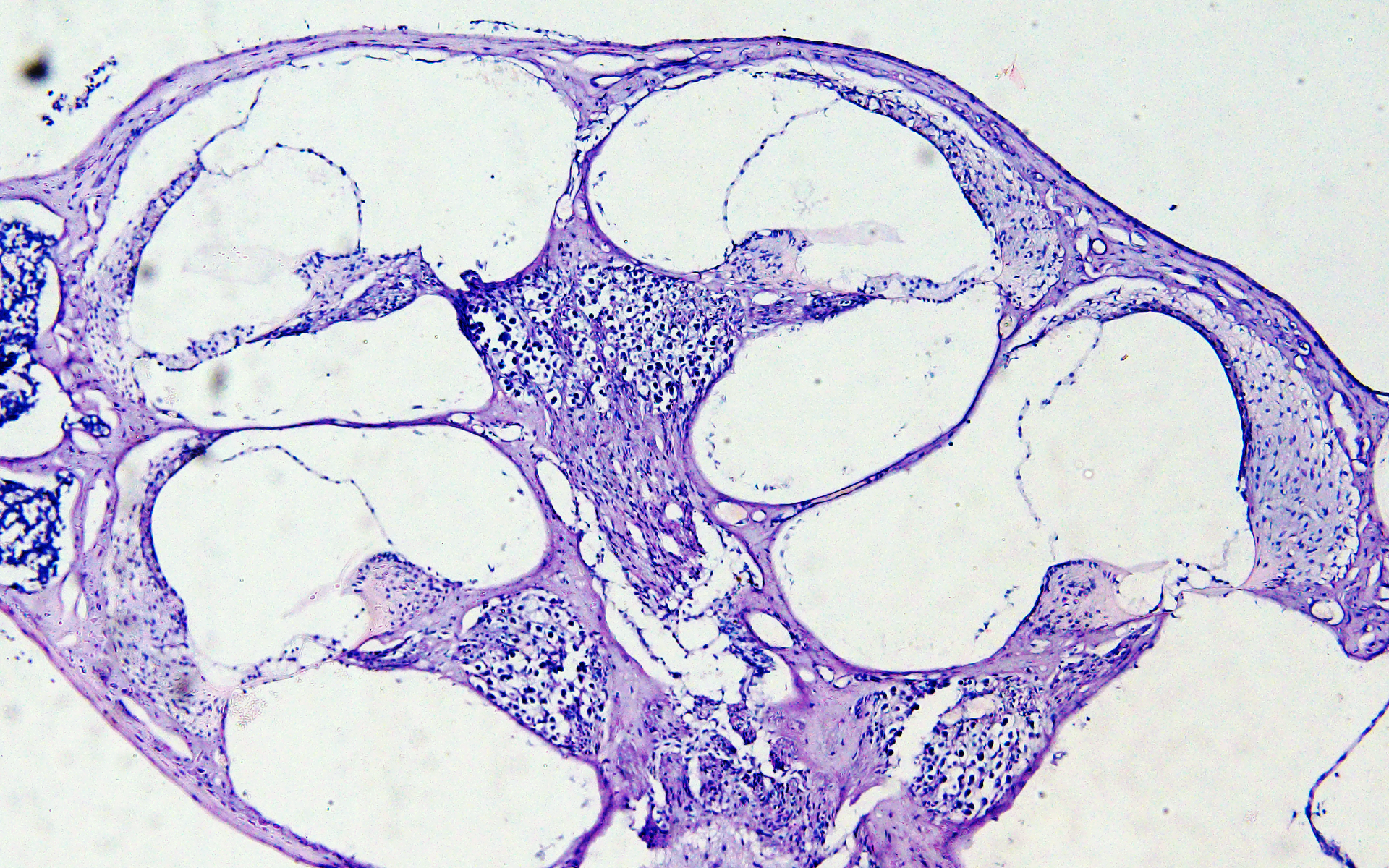

Supplement: Supplementary file 7 — Supporting File 7: advs75263‐sup‐0007‐Data5.zip. [file ADVS-13-e12538-s004.zip › Raw data of microscope images/Figure 2B-wt LPS.tif]

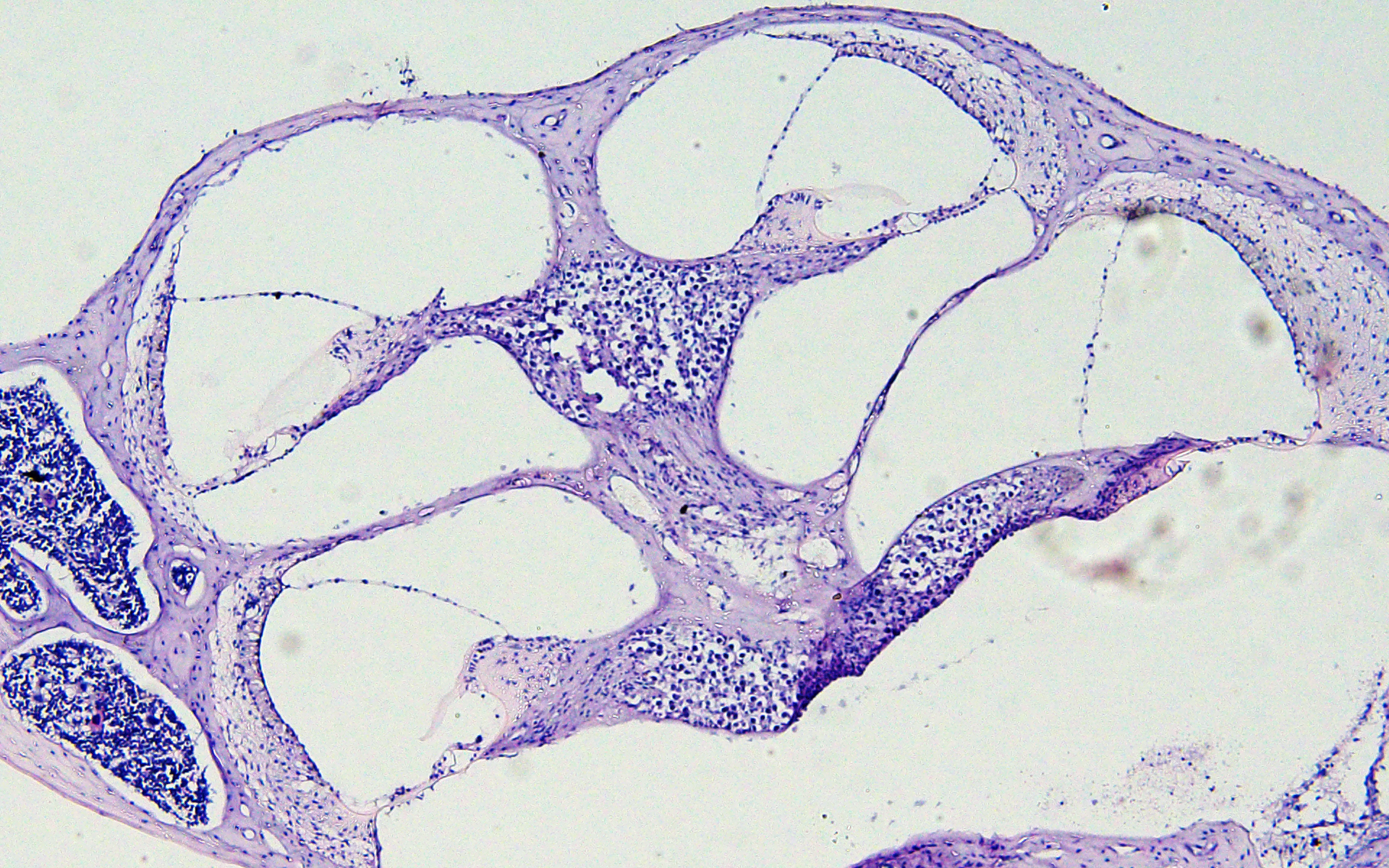

Supplement: Supplementary file 7 — Supporting File 7: advs75263‐sup‐0007‐Data5.zip. [file ADVS-13-e12538-s004.zip › Raw data of microscope images/Figure 2B-wt NS.tif]

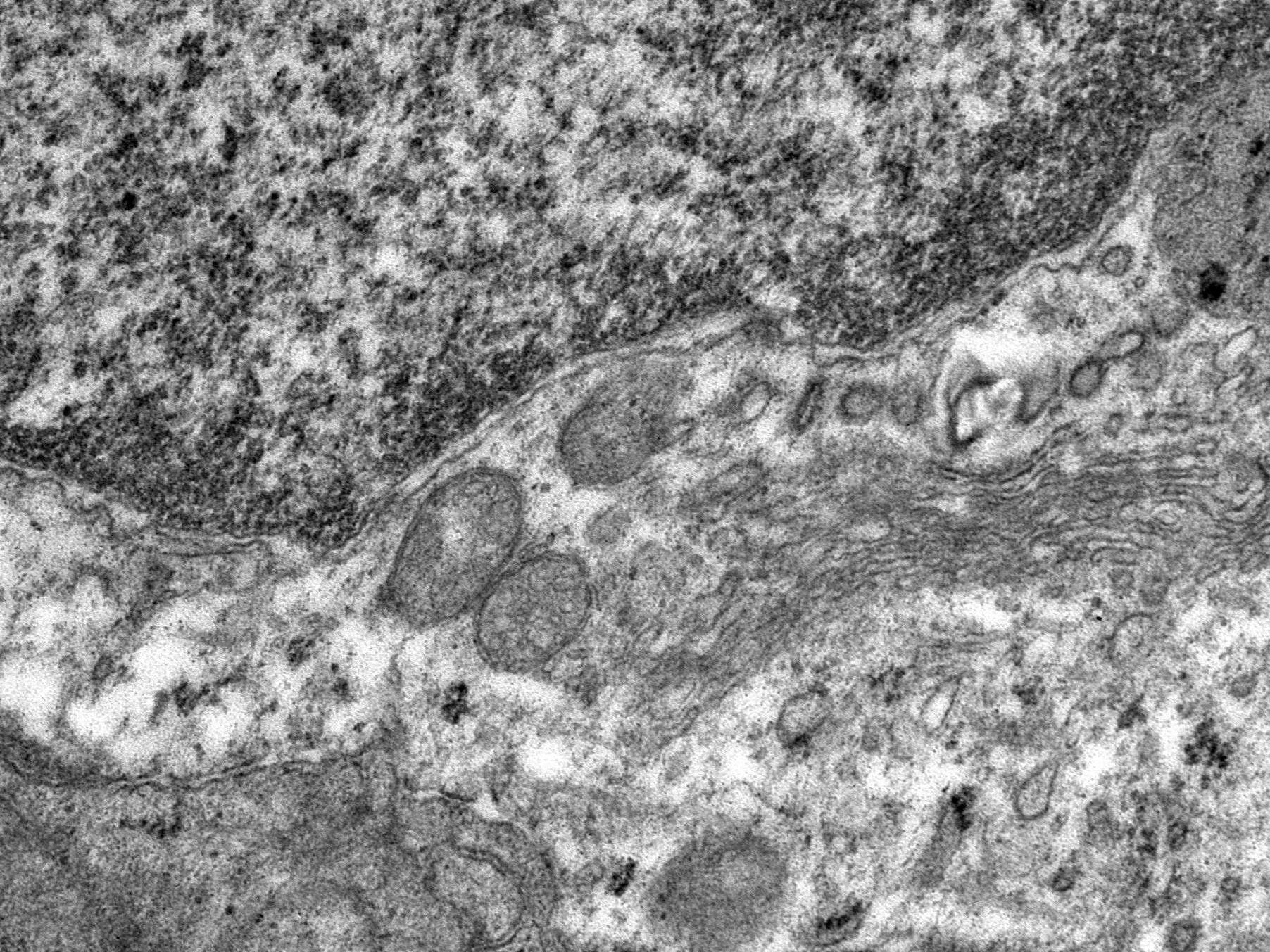

Supplement: Supplementary file 7 — Supporting File 7: advs75263‐sup‐0007‐Data5.zip. [file ADVS-13-e12538-s004.zip › Raw data of microscope images/Figure 2J-at1 LPS 2.jpg]

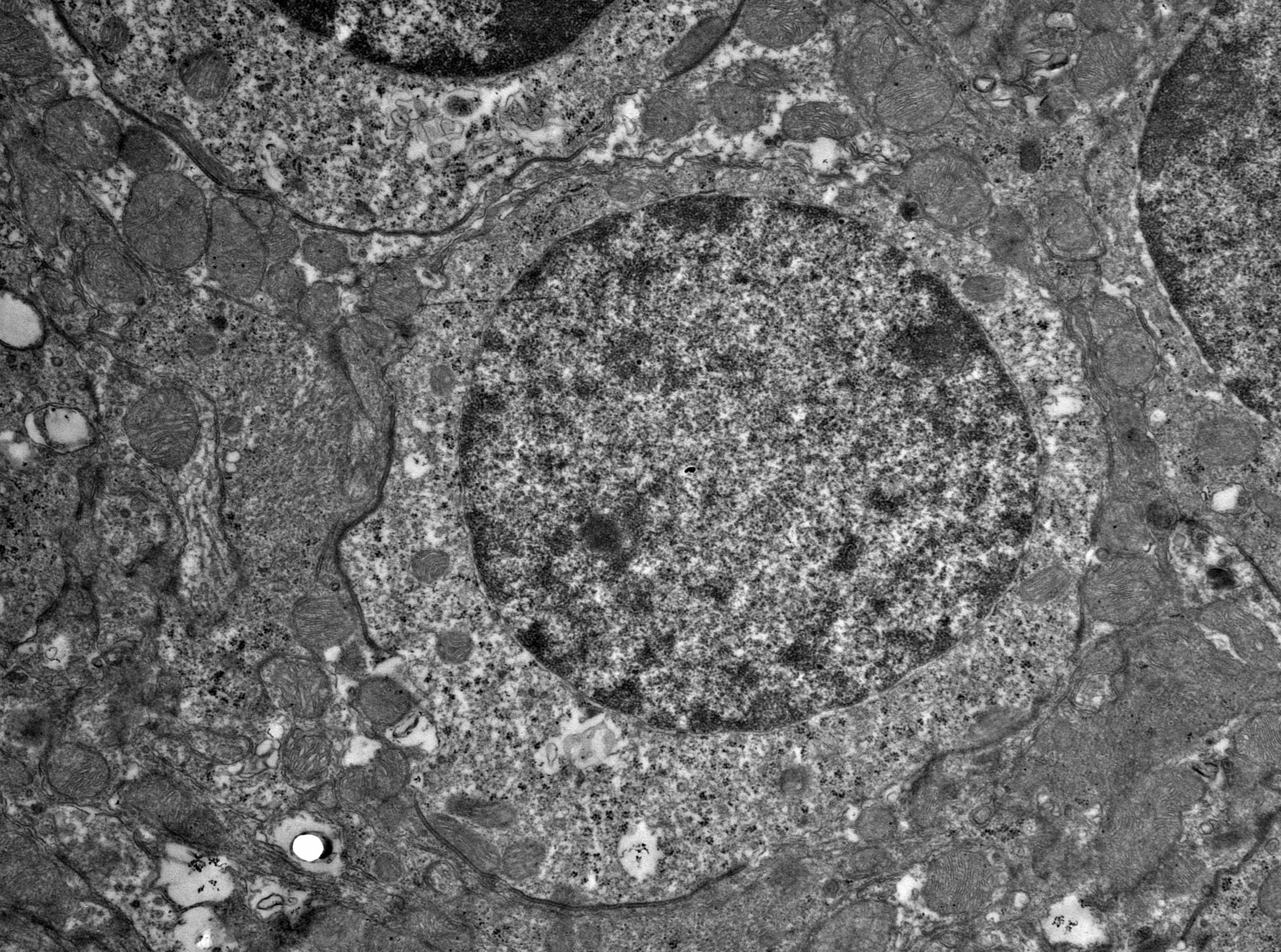

Supplement: Supplementary file 7 — Supporting File 7: advs75263‐sup‐0007‐Data5.zip. [file ADVS-13-e12538-s004.zip › Raw data of microscope images/Figure 2J-at1 LPS.jpg]

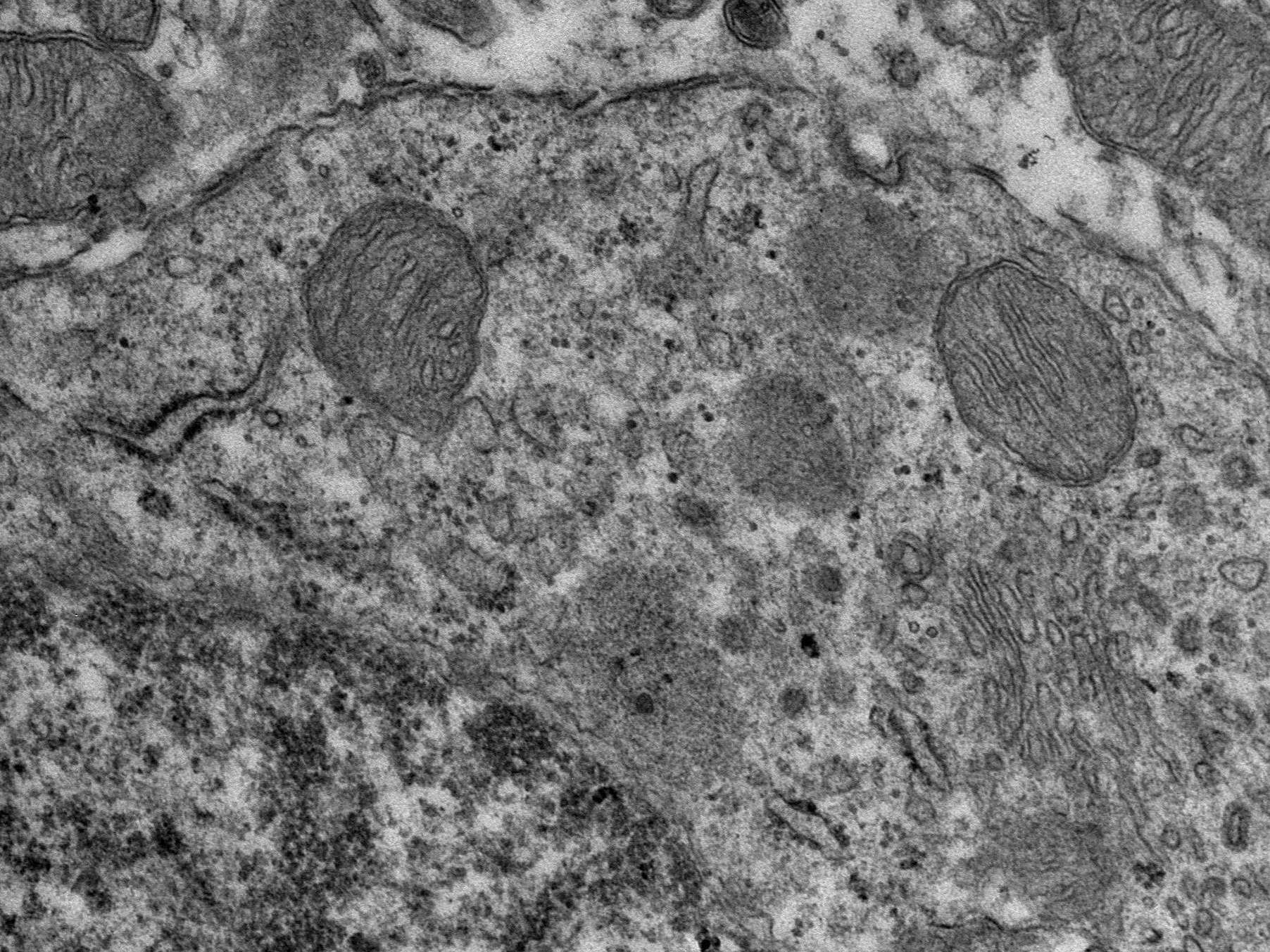

Supplement: Supplementary file 7 — Supporting File 7: advs75263‐sup‐0007‐Data5.zip. [file ADVS-13-e12538-s004.zip › Raw data of microscope images/Figure 2J-at1 NS 2.jpg]

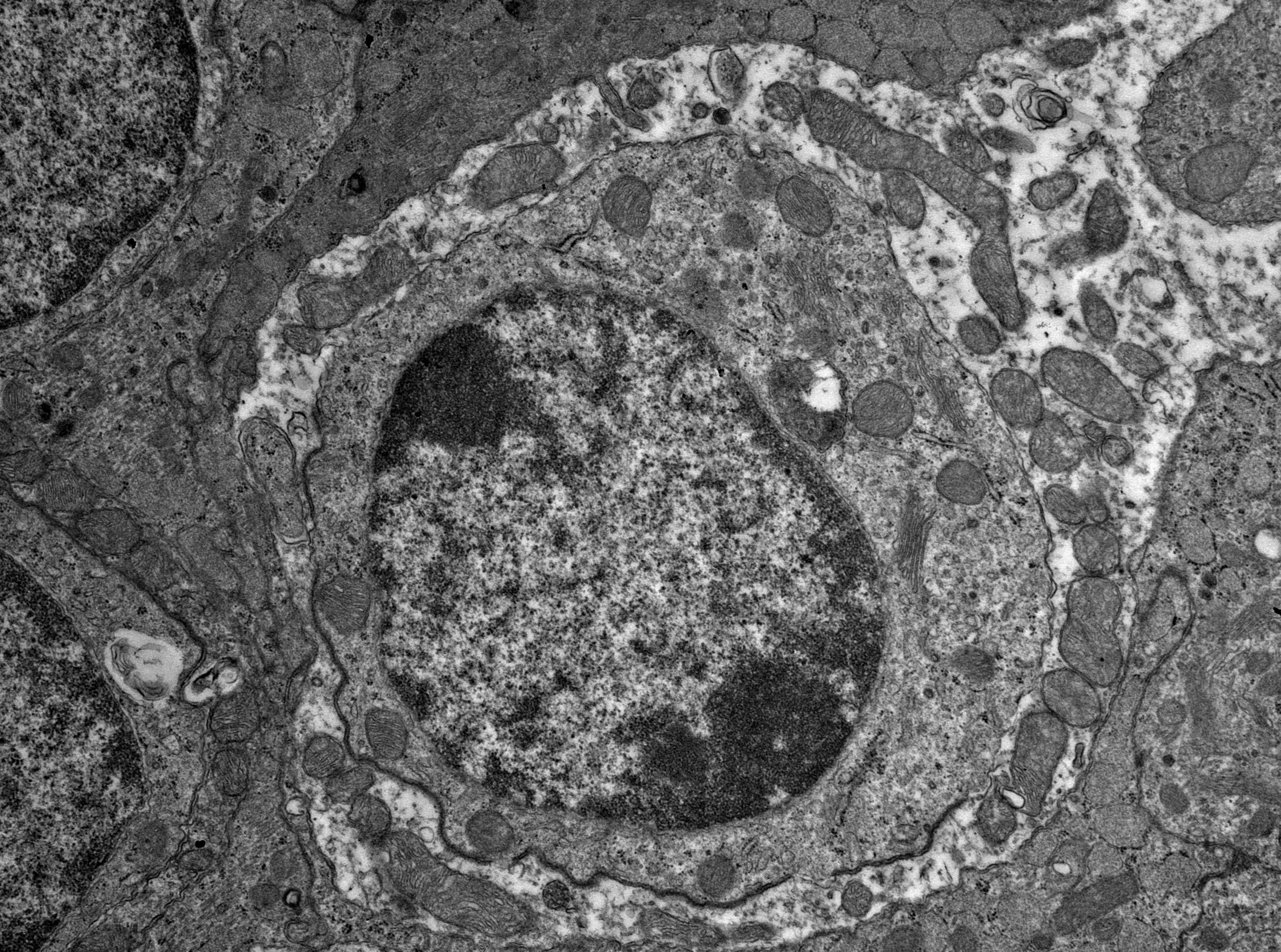

Supplement: Supplementary file 7 — Supporting File 7: advs75263‐sup‐0007‐Data5.zip. [file ADVS-13-e12538-s004.zip › Raw data of microscope images/Figure 2J-at1 NS.jpg]

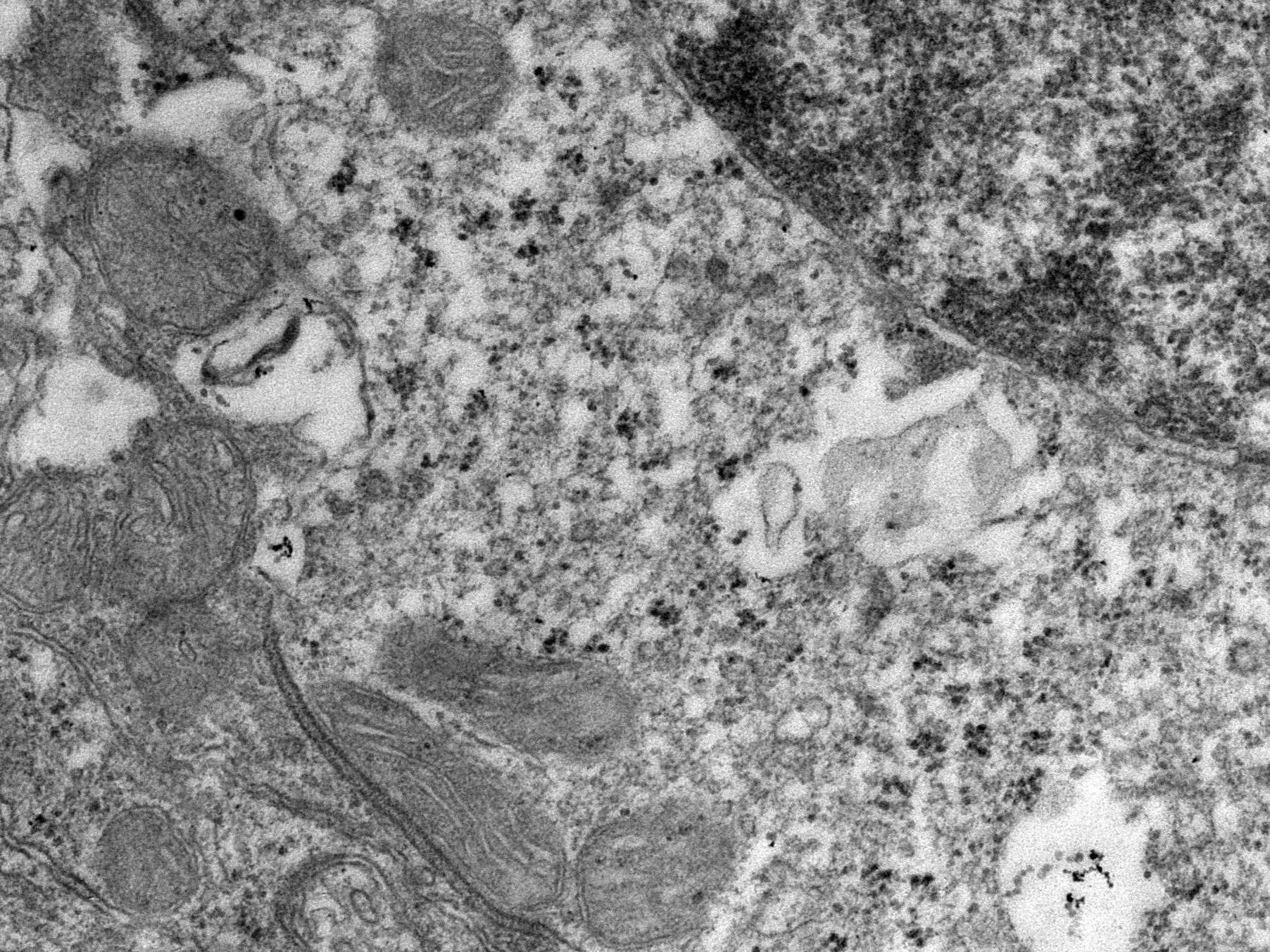

Supplement: Supplementary file 7 — Supporting File 7: advs75263‐sup‐0007‐Data5.zip. [file ADVS-13-e12538-s004.zip › Raw data of microscope images/Figure 2J-flfl LPS 2.jpg]

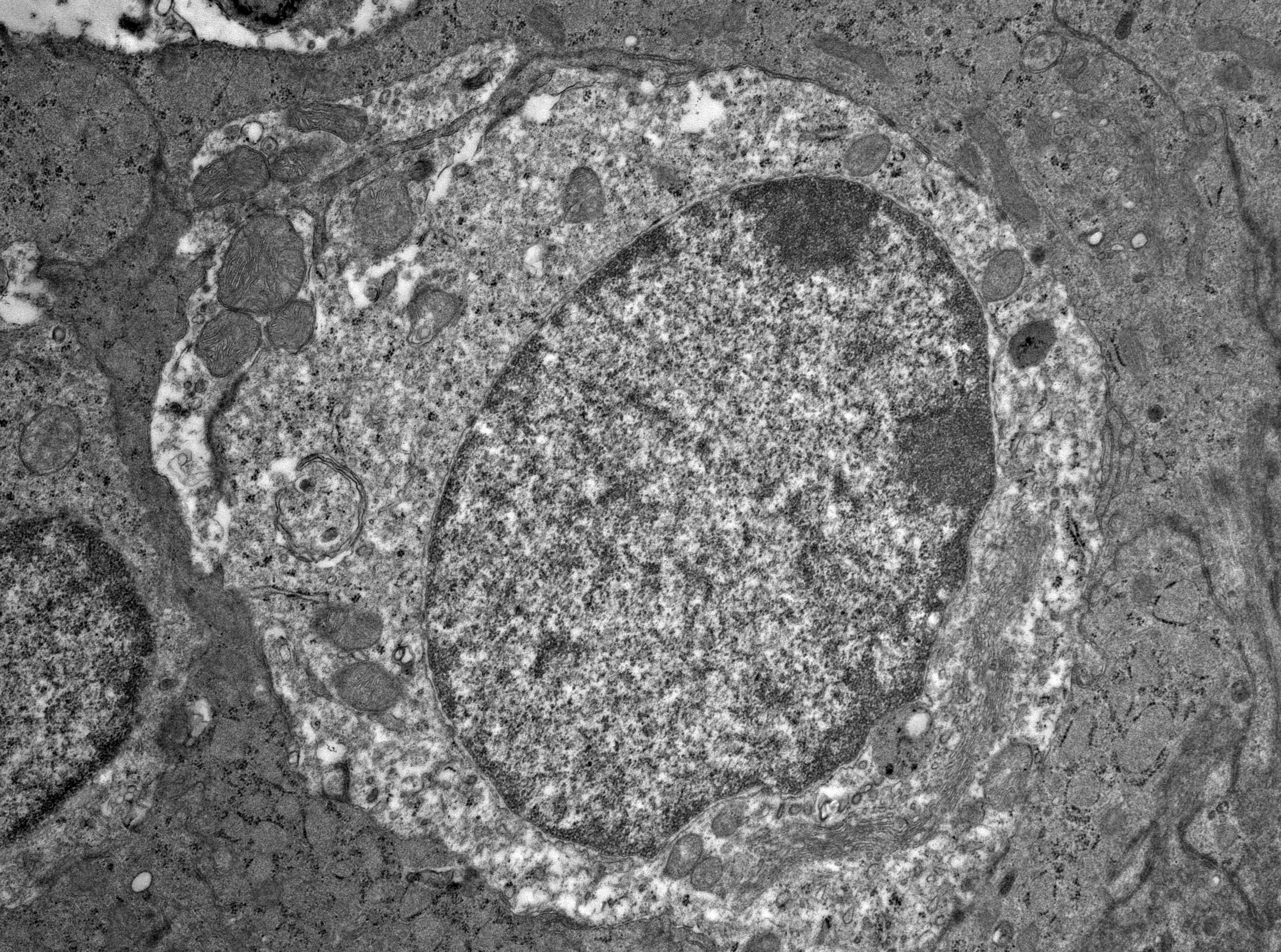

Supplement: Supplementary file 7 — Supporting File 7: advs75263‐sup‐0007‐Data5.zip. [file ADVS-13-e12538-s004.zip › Raw data of microscope images/Figure 2J-flfl LPS.jpg]

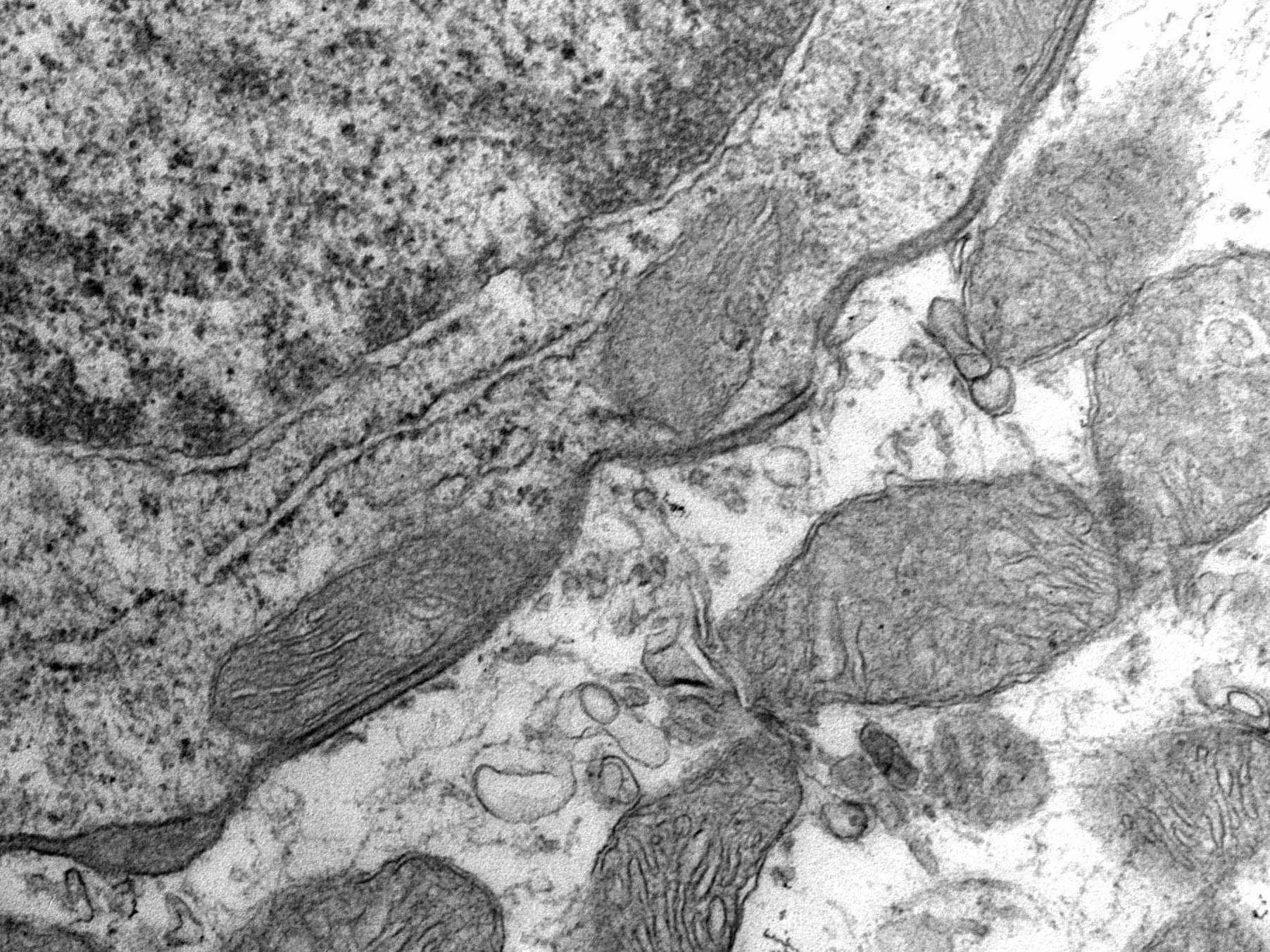

Supplement: Supplementary file 7 — Supporting File 7: advs75263‐sup‐0007‐Data5.zip. [file ADVS-13-e12538-s004.zip › Raw data of microscope images/Figure 2J-flfl NS 2.jpg]

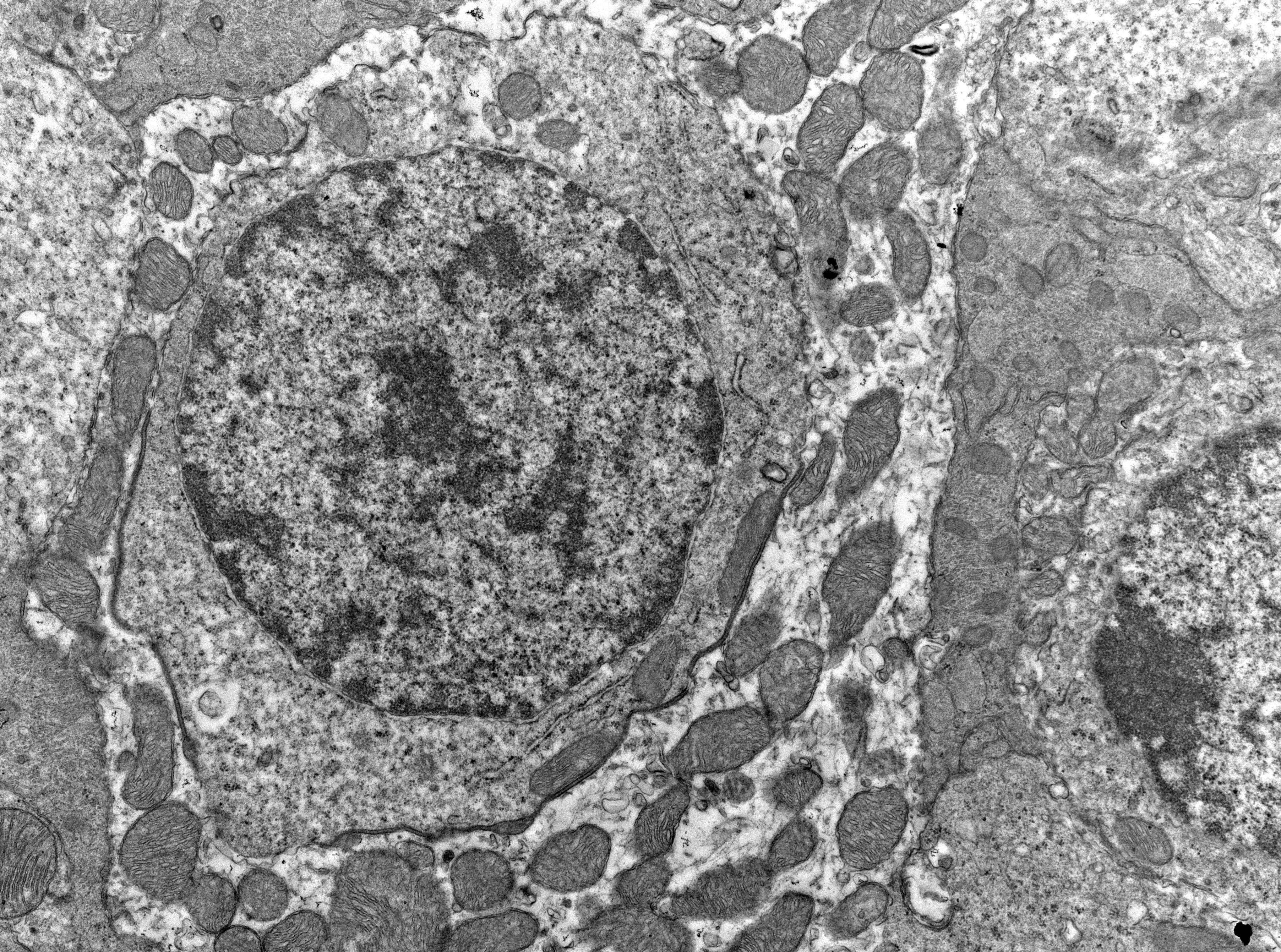

Supplement: Supplementary file 7 — Supporting File 7: advs75263‐sup‐0007‐Data5.zip. [file ADVS-13-e12538-s004.zip › Raw data of microscope images/Figure 2J-flfl NS.jpg]

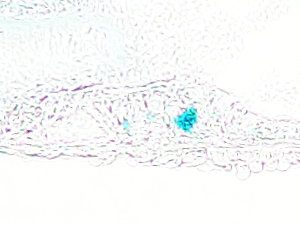

Supplement: Supplementary file 7 — Supporting File 7: advs75263‐sup‐0007‐Data5.zip. [file ADVS-13-e12538-s004.zip › Raw data of microscope images/Figure 2K-KO LPS.tif]

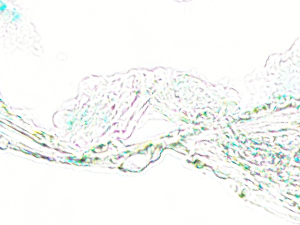

Supplement: Supplementary file 7 — Supporting File 7: advs75263‐sup‐0007‐Data5.zip. [file ADVS-13-e12538-s004.zip › Raw data of microscope images/Figure 2K-KO.tif]

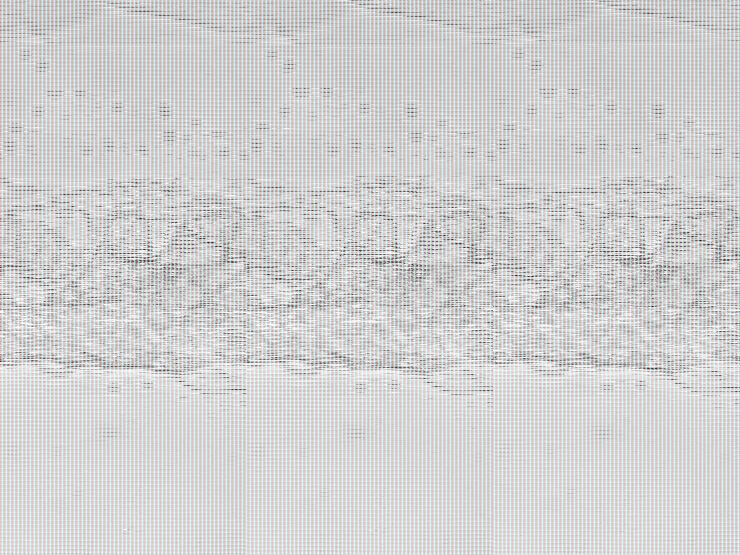

Supplement: Supplementary file 7 — Supporting File 7: advs75263‐sup‐0007‐Data5.zip. [file ADVS-13-e12538-s004.zip › Raw data of microscope images/Figure 2K-wt LPS.tif]

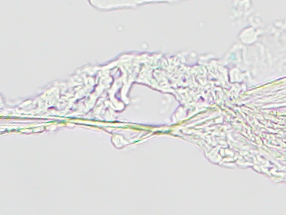

Supplement: Supplementary file 7 — Supporting File 7: advs75263‐sup‐0007‐Data5.zip. [file ADVS-13-e12538-s004.zip › Raw data of microscope images/Figure 2K-wt.tif]

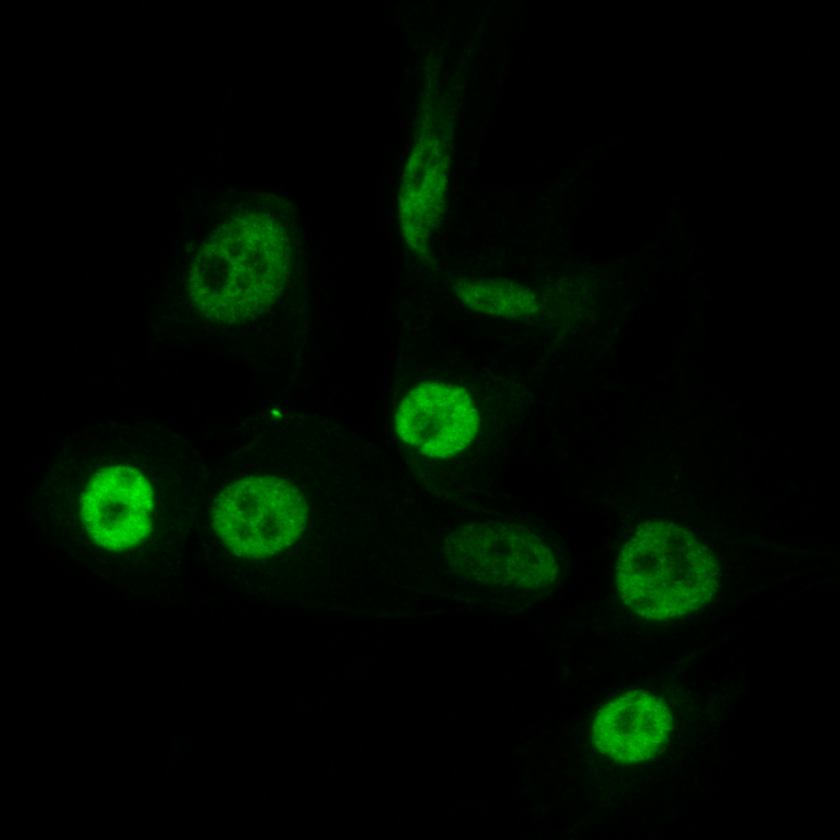

Supplement: Supplementary file 7 — Supporting File 7: advs75263‐sup‐0007‐Data5.zip. [file ADVS-13-e12538-s004.zip › Raw data of microscope images/Figure 3H-siHDAC6 GATA4.tif]

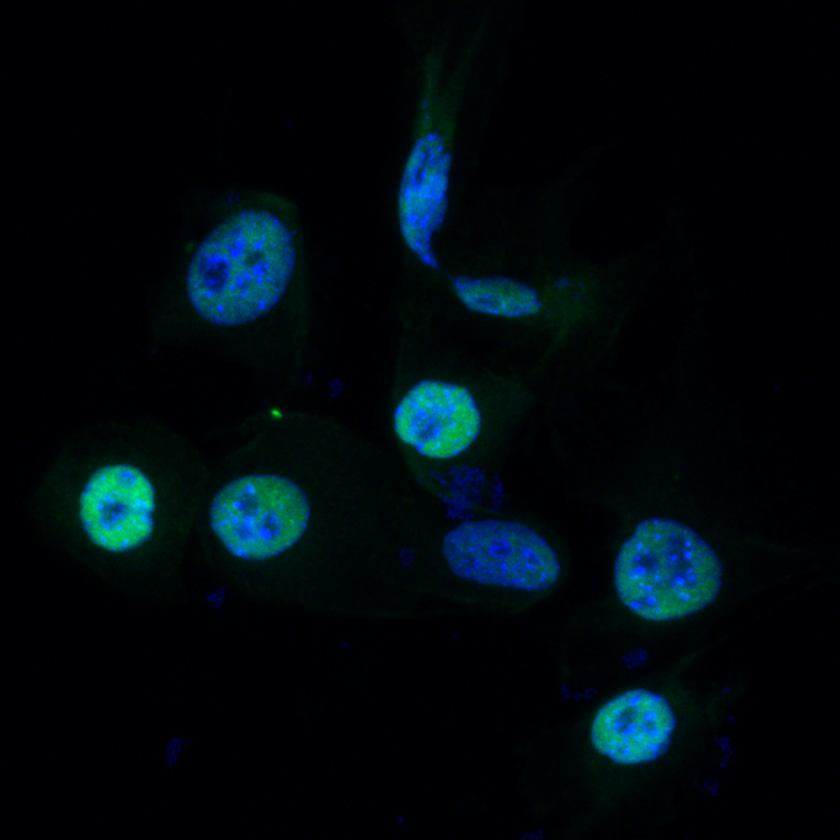

Supplement: Supplementary file 7 — Supporting File 7: advs75263‐sup‐0007‐Data5.zip. [file ADVS-13-e12538-s004.zip › Raw data of microscope images/Figure 3H-siHDAC6 Merge.tif]

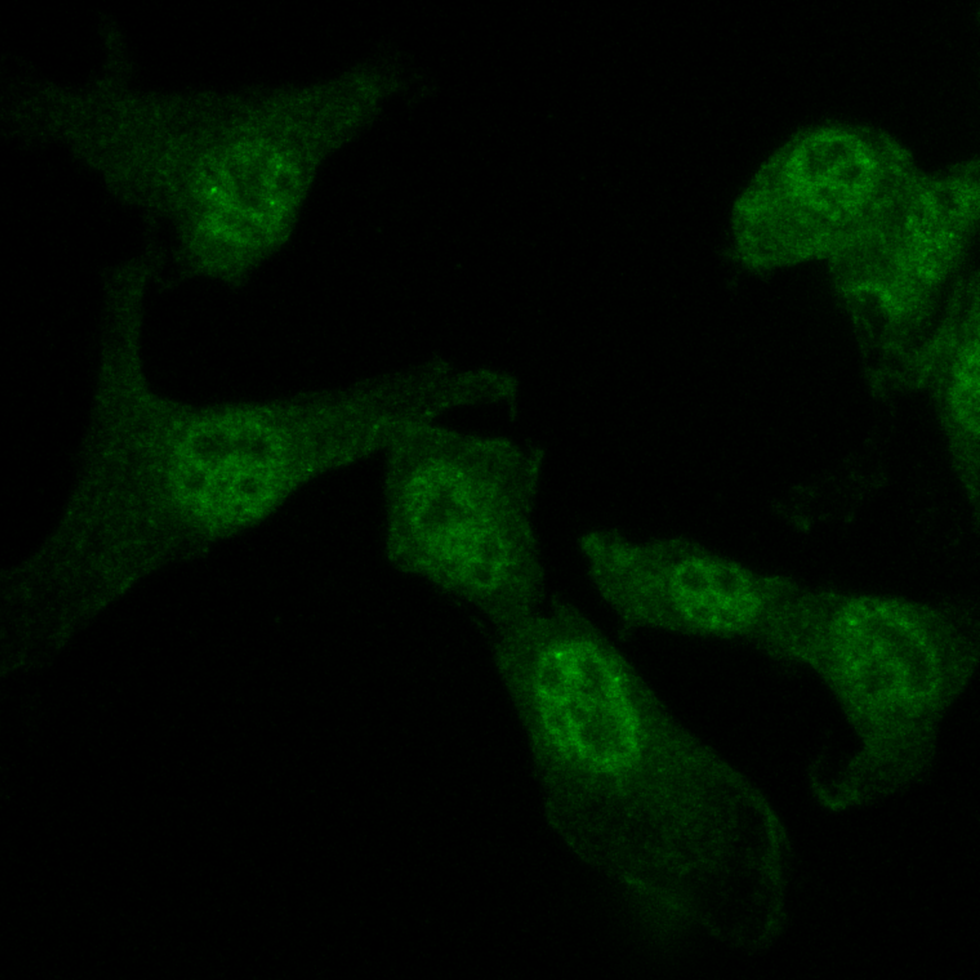

Supplement: Supplementary file 7 — Supporting File 7: advs75263‐sup‐0007‐Data5.zip. [file ADVS-13-e12538-s004.zip › Raw data of microscope images/Figure 3H-siNC GATA4.tif]

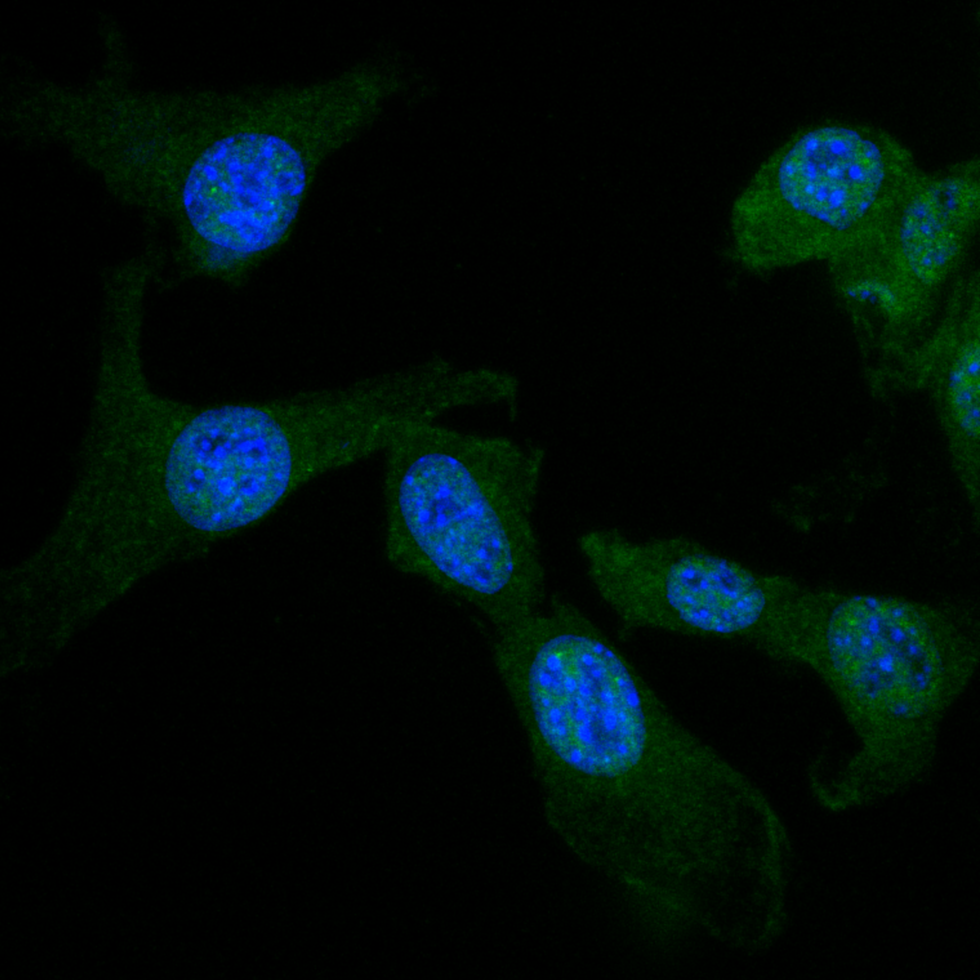

Supplement: Supplementary file 7 — Supporting File 7: advs75263‐sup‐0007‐Data5.zip. [file ADVS-13-e12538-s004.zip › Raw data of microscope images/Figure 3H-siNC Merge.tif]

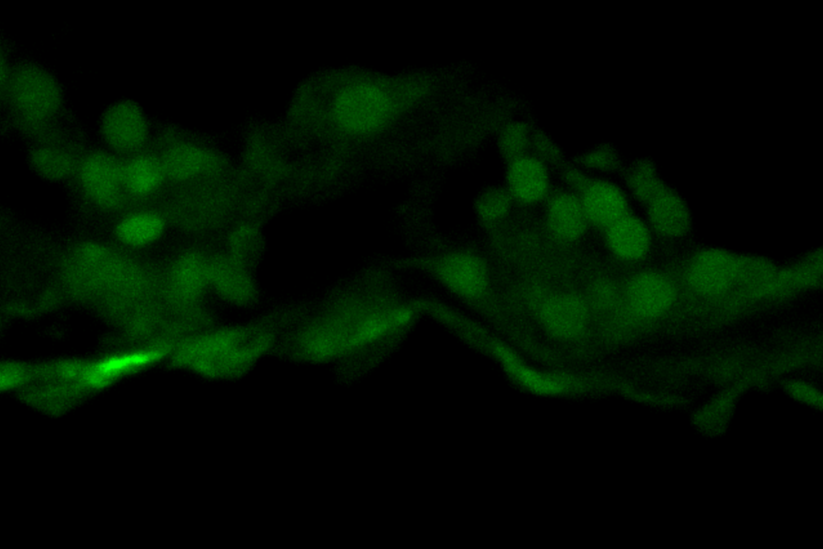

Supplement: Supplementary file 7 — Supporting File 7: advs75263‐sup‐0007‐Data5.zip. [file ADVS-13-e12538-s004.zip › Raw data of microscope images/Figure 3J-LPS (1) GATA4.tif]

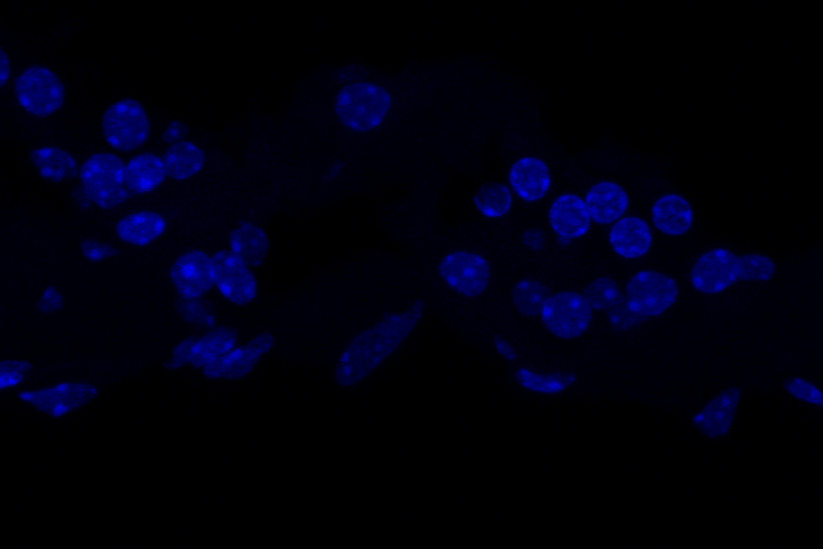

Supplement: Supplementary file 7 — Supporting File 7: advs75263‐sup‐0007‐Data5.zip. [file ADVS-13-e12538-s004.zip › Raw data of microscope images/Figure 3J-LPS (2) DAPI.tif]

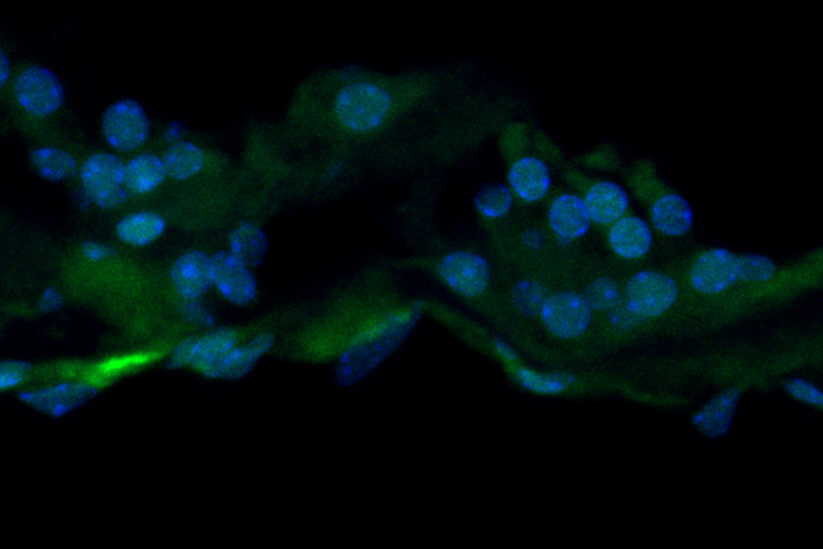

Supplement: Supplementary file 7 — Supporting File 7: advs75263‐sup‐0007‐Data5.zip. [file ADVS-13-e12538-s004.zip › Raw data of microscope images/Figure 3J-LPS (3) Merge.tif]

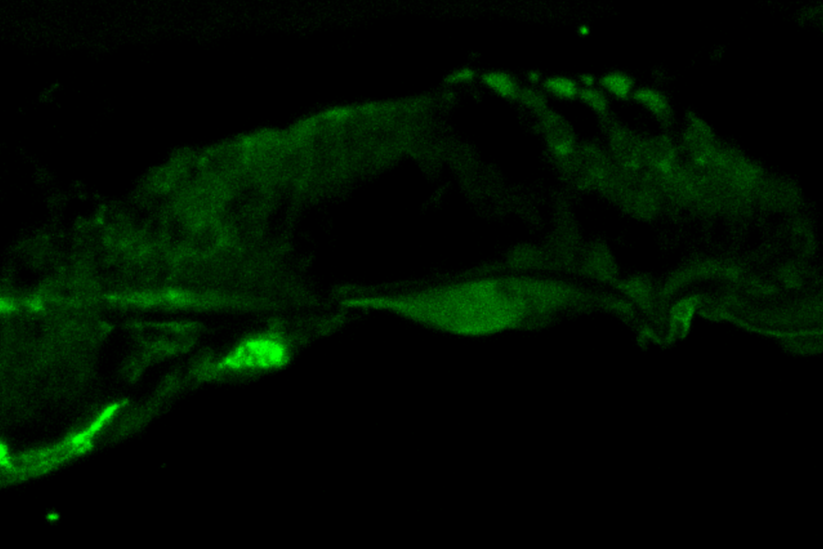

Supplement: Supplementary file 7 — Supporting File 7: advs75263‐sup‐0007‐Data5.zip. [file ADVS-13-e12538-s004.zip › Raw data of microscope images/Figure 3J-NS (1) GATA4.tif]

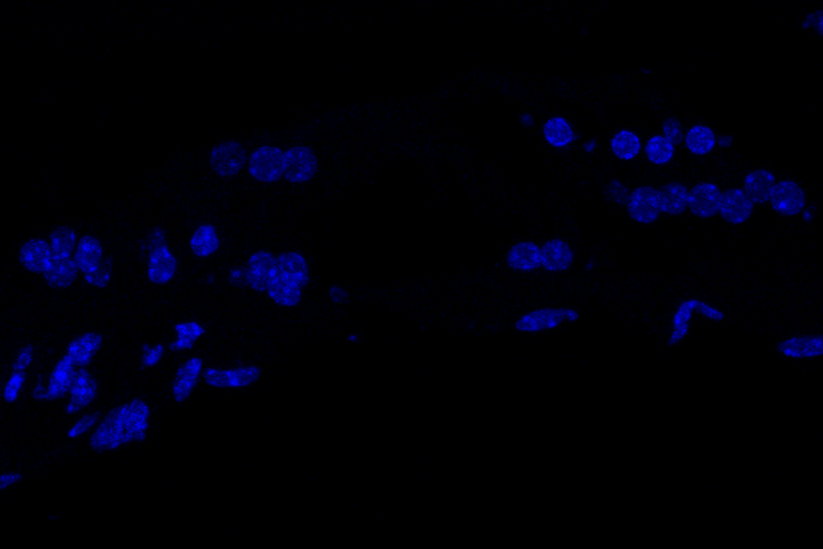

Supplement: Supplementary file 7 — Supporting File 7: advs75263‐sup‐0007‐Data5.zip. [file ADVS-13-e12538-s004.zip › Raw data of microscope images/Figure 3J-NS (2) DAPI.tif]

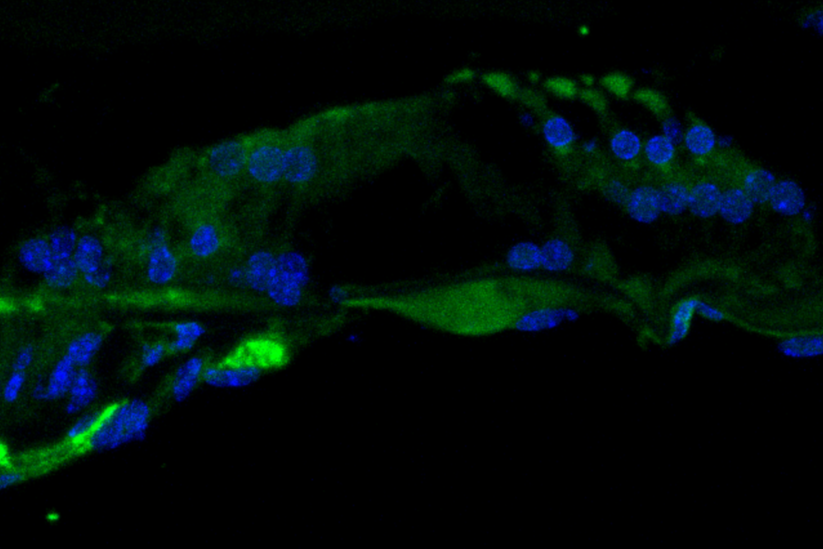

Supplement: Supplementary file 7 — Supporting File 7: advs75263‐sup‐0007‐Data5.zip. [file ADVS-13-e12538-s004.zip › Raw data of microscope images/Figure 3J-NS (3) Merge.tif]

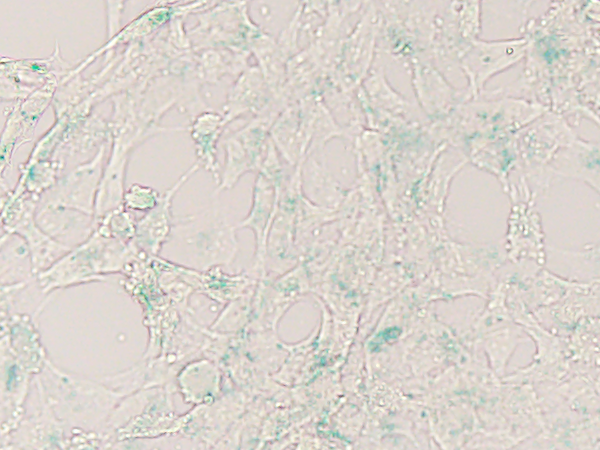

Supplement: Supplementary file 7 — Supporting File 7: advs75263‐sup‐0007‐Data5.zip. [file ADVS-13-e12538-s004.zip › Raw data of microscope images/Figure 3O-Dox+siHDAC6.tif]

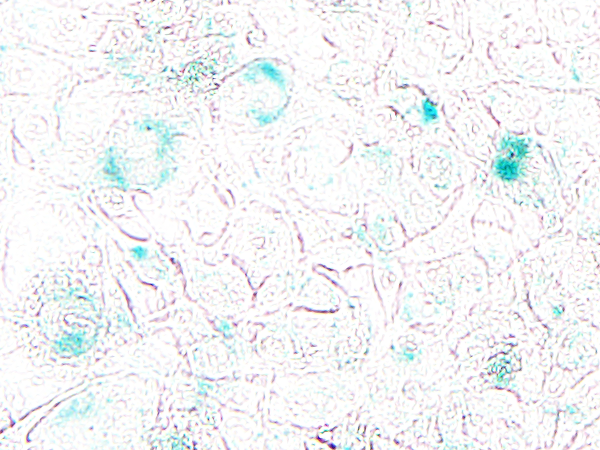

Supplement: Supplementary file 7 — Supporting File 7: advs75263‐sup‐0007‐Data5.zip. [file ADVS-13-e12538-s004.zip › Raw data of microscope images/Figure 3O-Dox.tif]

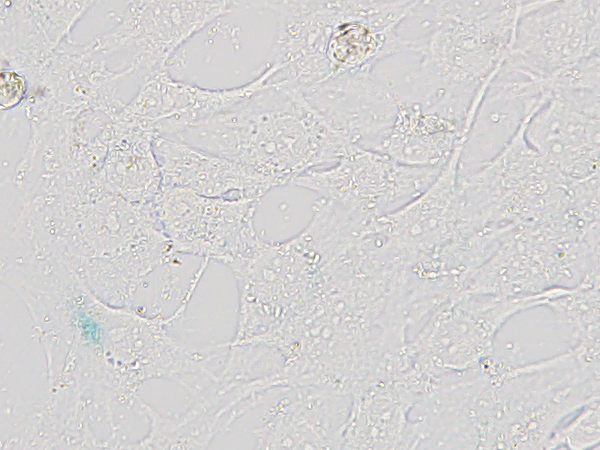

Supplement: Supplementary file 7 — Supporting File 7: advs75263‐sup‐0007‐Data5.zip. [file ADVS-13-e12538-s004.zip › Raw data of microscope images/Figure 3O-saline.tif]

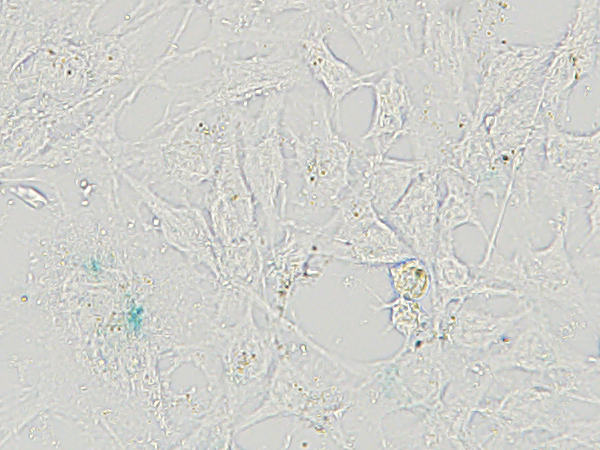

Supplement: Supplementary file 7 — Supporting File 7: advs75263‐sup‐0007‐Data5.zip. [file ADVS-13-e12538-s004.zip › Raw data of microscope images/Figure 3O-siHDAC6.tif]

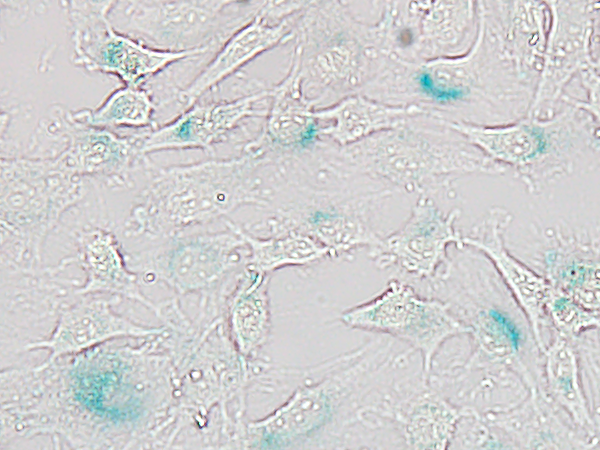

Supplement: Supplementary file 7 — Supporting File 7: advs75263‐sup‐0007‐Data5.zip. [file ADVS-13-e12538-s004.zip › Raw data of microscope images/Figure 5A-HtrA1.tif]

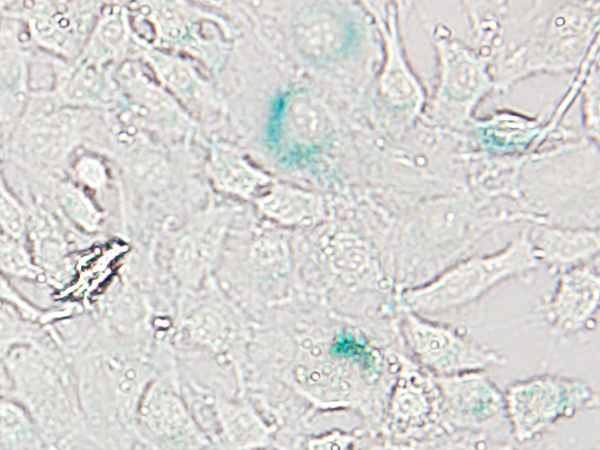

Supplement: Supplementary file 7 — Supporting File 7: advs75263‐sup‐0007‐Data5.zip. [file ADVS-13-e12538-s004.zip › Raw data of microscope images/Figure 5A-siGATA4+HtrA1.tif]

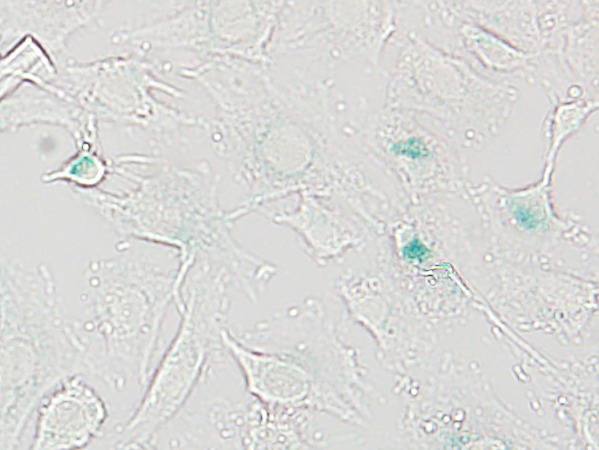

Supplement: Supplementary file 7 — Supporting File 7: advs75263‐sup‐0007‐Data5.zip. [file ADVS-13-e12538-s004.zip › Raw data of microscope images/Figure 5A-siGATA4.tif]

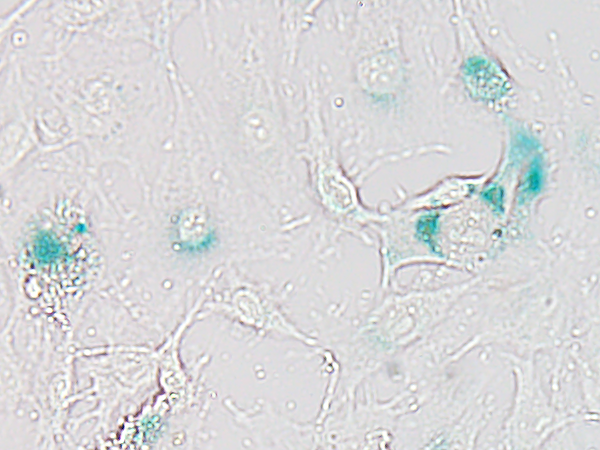

Supplement: Supplementary file 7 — Supporting File 7: advs75263‐sup‐0007‐Data5.zip. [file ADVS-13-e12538-s004.zip › Raw data of microscope images/Figure 5A-siNC.tif]

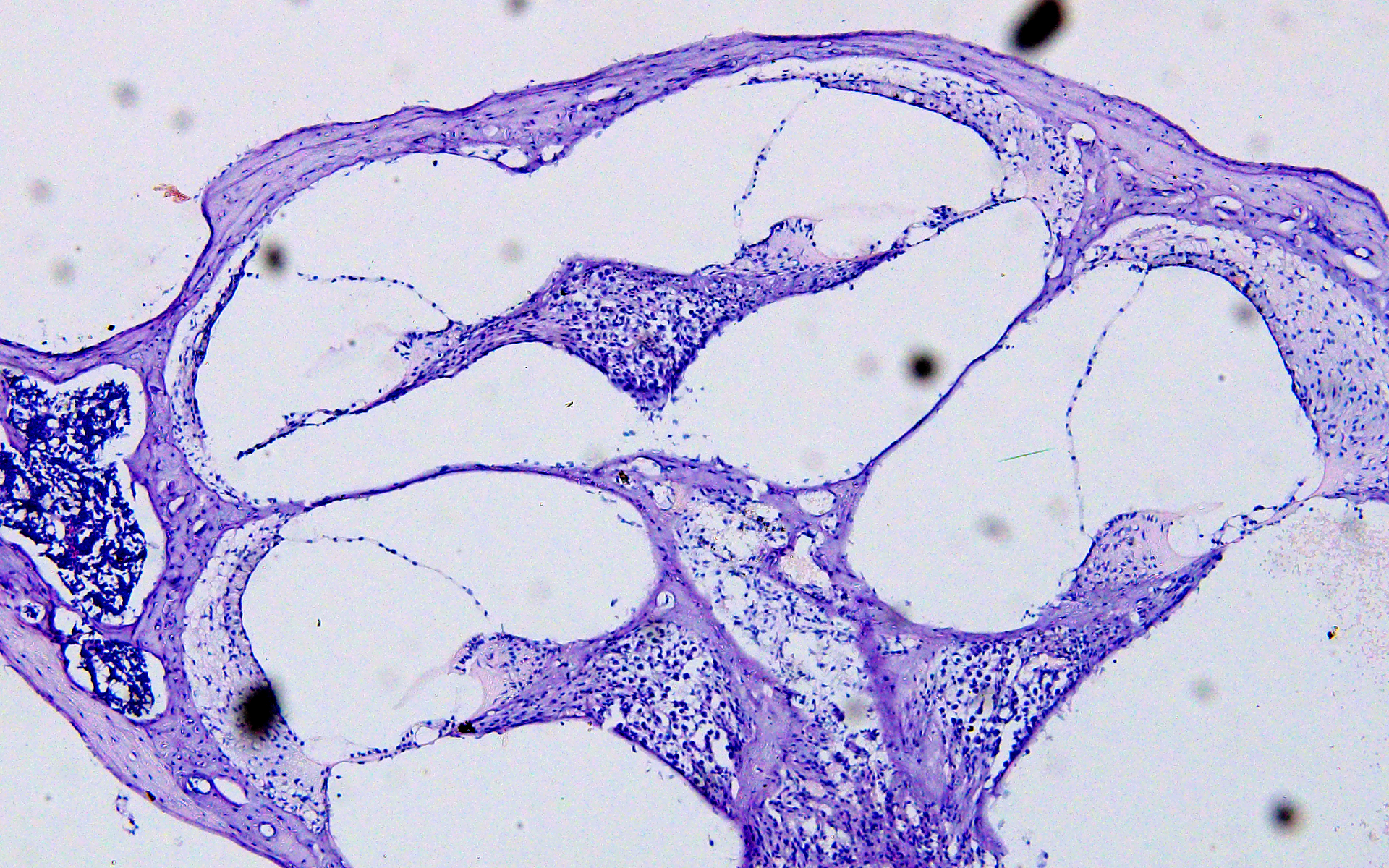

Supplement: Supplementary file 7 — Supporting File 7: advs75263‐sup‐0007‐Data5.zip. [file ADVS-13-e12538-s004.zip › Raw data of microscope images/Figure 6B-KO Ht1.tif]

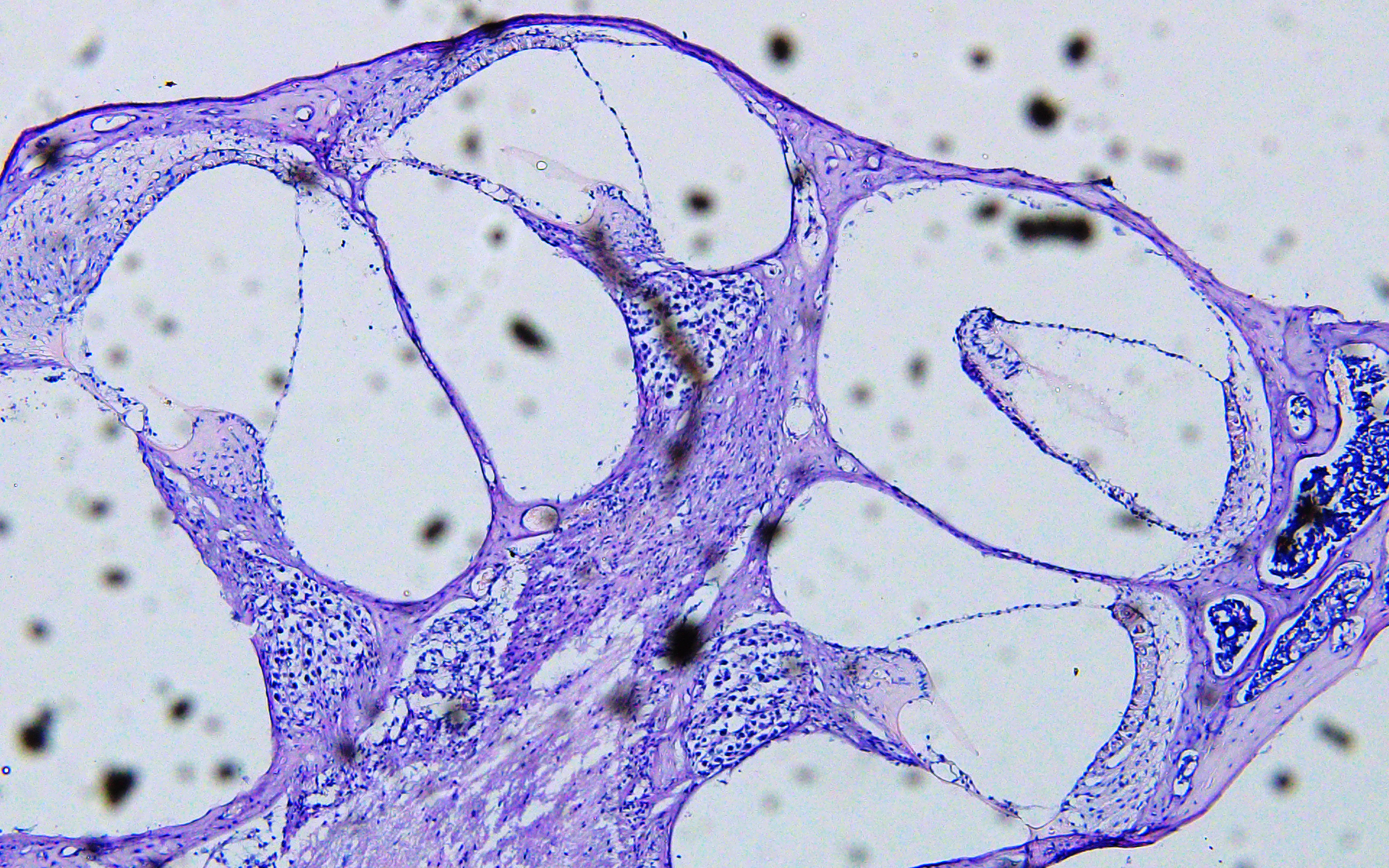

Supplement: Supplementary file 7 — Supporting File 7: advs75263‐sup‐0007‐Data5.zip. [file ADVS-13-e12538-s004.zip › Raw data of microscope images/Figure 6B-KO.tif]

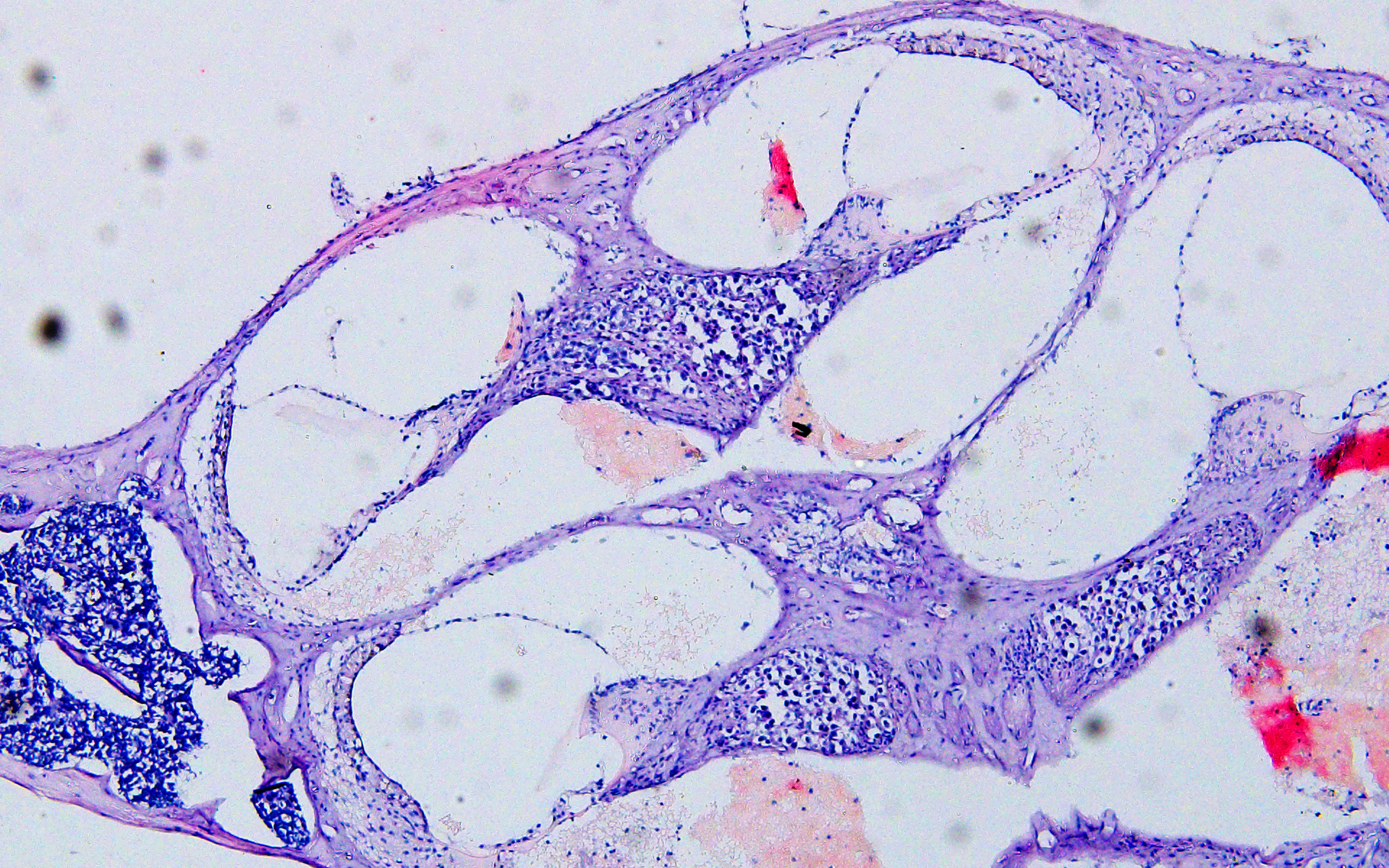

Supplement: Supplementary file 7 — Supporting File 7: advs75263‐sup‐0007‐Data5.zip. [file ADVS-13-e12538-s004.zip › Raw data of microscope images/Figure 6B-wt Ht1.tif]

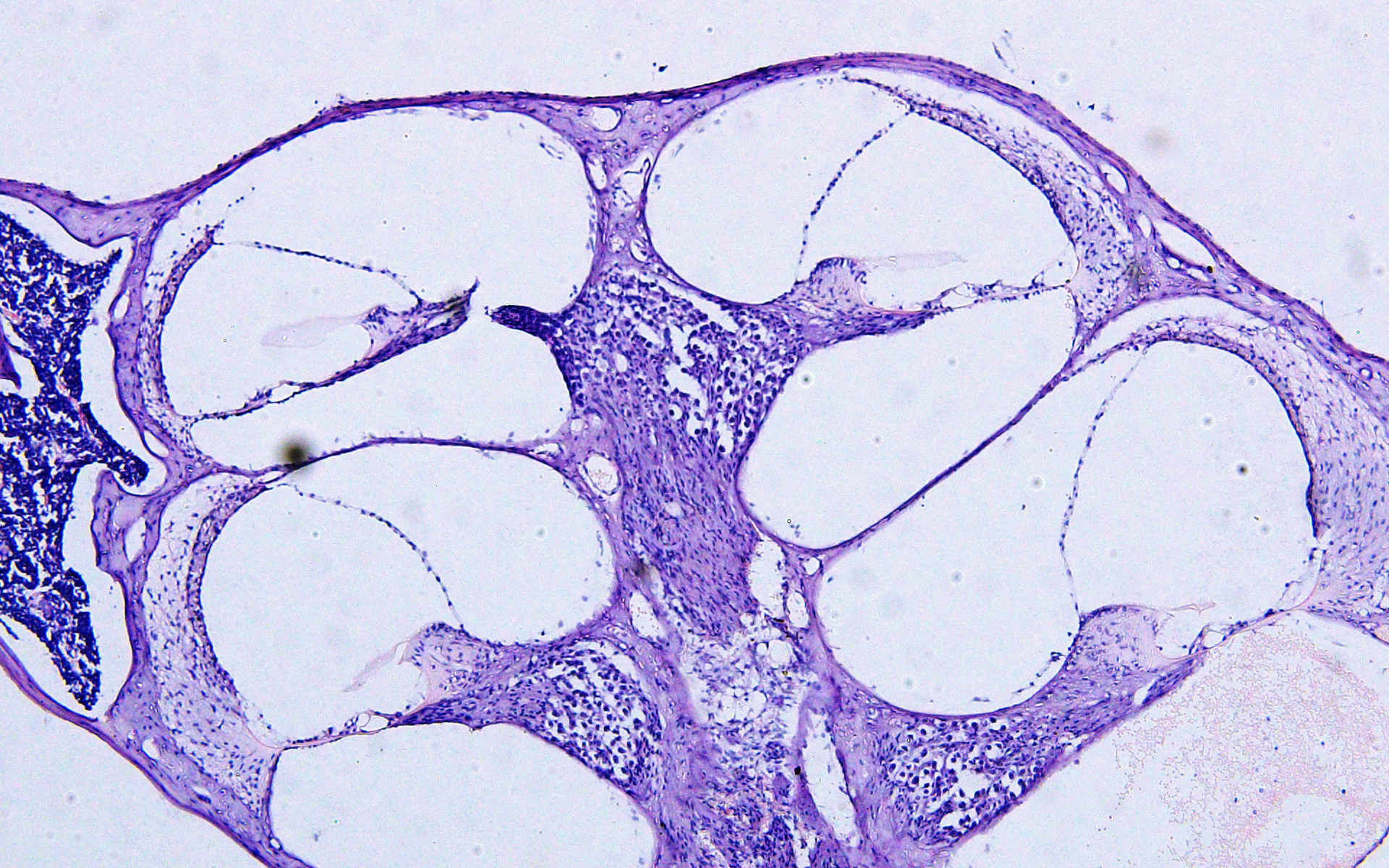

Supplement: Supplementary file 7 — Supporting File 7: advs75263‐sup‐0007‐Data5.zip. [file ADVS-13-e12538-s004.zip › Raw data of microscope images/Figure 6B-wt.tif]

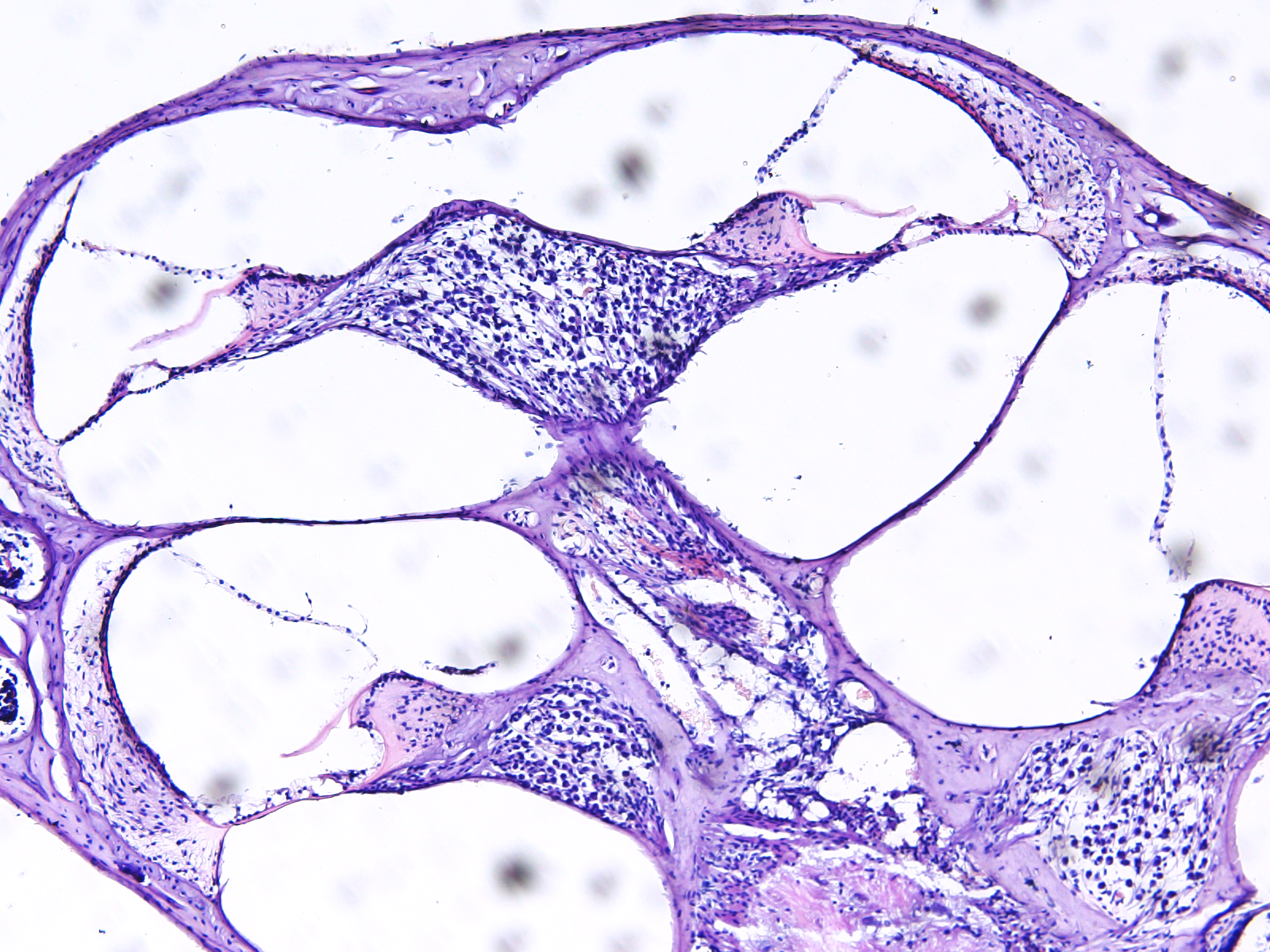

Supplement: Supplementary file 7 — Supporting File 7: advs75263‐sup‐0007‐Data5.zip. [file ADVS-13-e12538-s004.zip › Raw data of microscope images/Figure 7F-KO.tif]

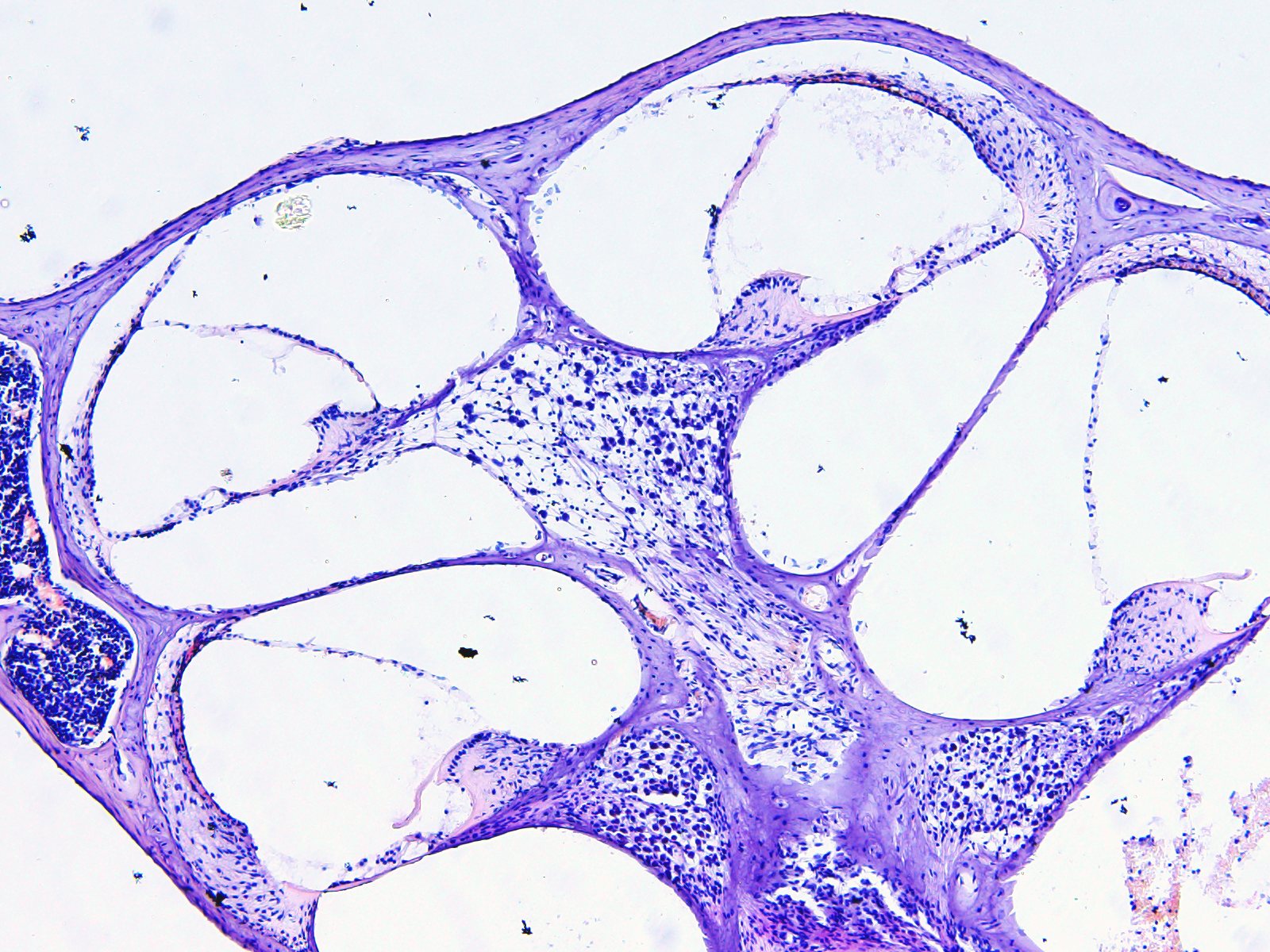

Supplement: Supplementary file 7 — Supporting File 7: advs75263‐sup‐0007‐Data5.zip. [file ADVS-13-e12538-s004.zip › Raw data of microscope images/Figure 7F-wt.tif]

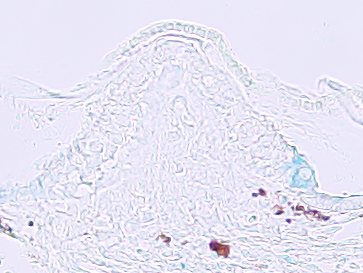

Supplement: Supplementary file 7 — Supporting File 7: advs75263‐sup‐0007‐Data5.zip. [file ADVS-13-e12538-s004.zip › Raw data of microscope images/Figure 7G-KO Ampulla.tif]

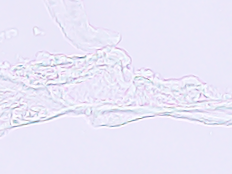

Supplement: Supplementary file 7 — Supporting File 7: advs75263‐sup‐0007‐Data5.zip. [file ADVS-13-e12538-s004.zip › Raw data of microscope images/Figure 7G-KO Corti.tif]

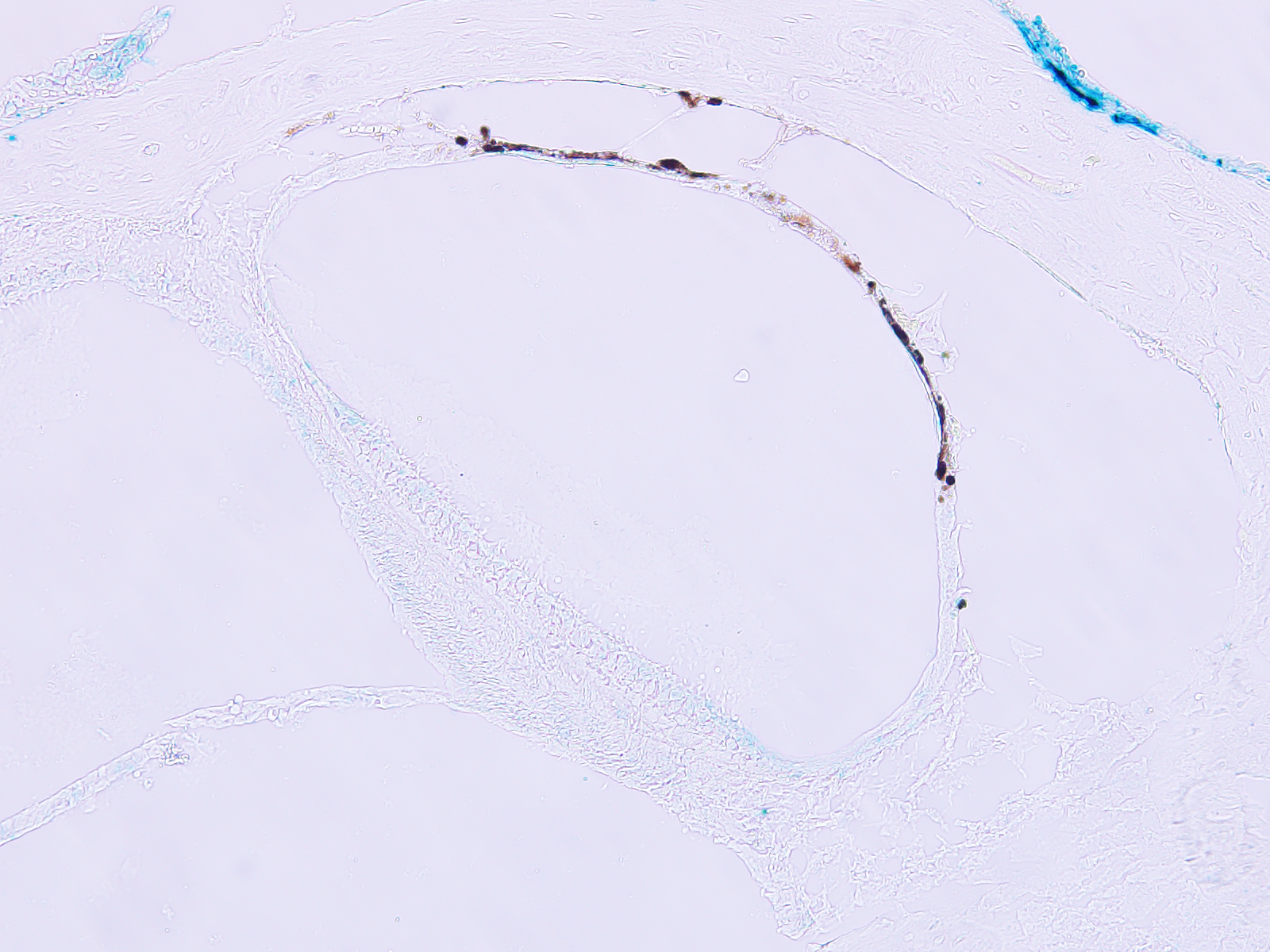

Supplement: Supplementary file 7 — Supporting File 7: advs75263‐sup‐0007‐Data5.zip. [file ADVS-13-e12538-s004.zip › Raw data of microscope images/Figure 7G-KO Macula.tif]

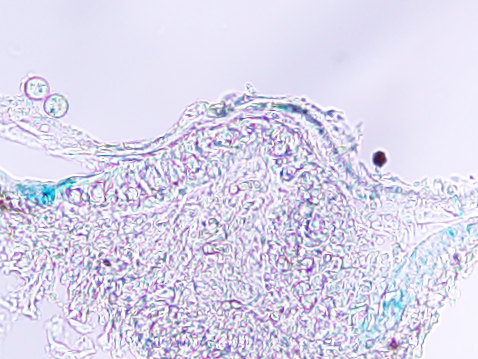

Supplement: Supplementary file 7 — Supporting File 7: advs75263‐sup‐0007‐Data5.zip. [file ADVS-13-e12538-s004.zip › Raw data of microscope images/Figure 7G-wt Ampulla.tif]

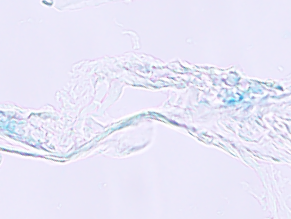

Supplement: Supplementary file 7 — Supporting File 7: advs75263‐sup‐0007‐Data5.zip. [file ADVS-13-e12538-s004.zip › Raw data of microscope images/Figure 7G-wt Corti.tif]

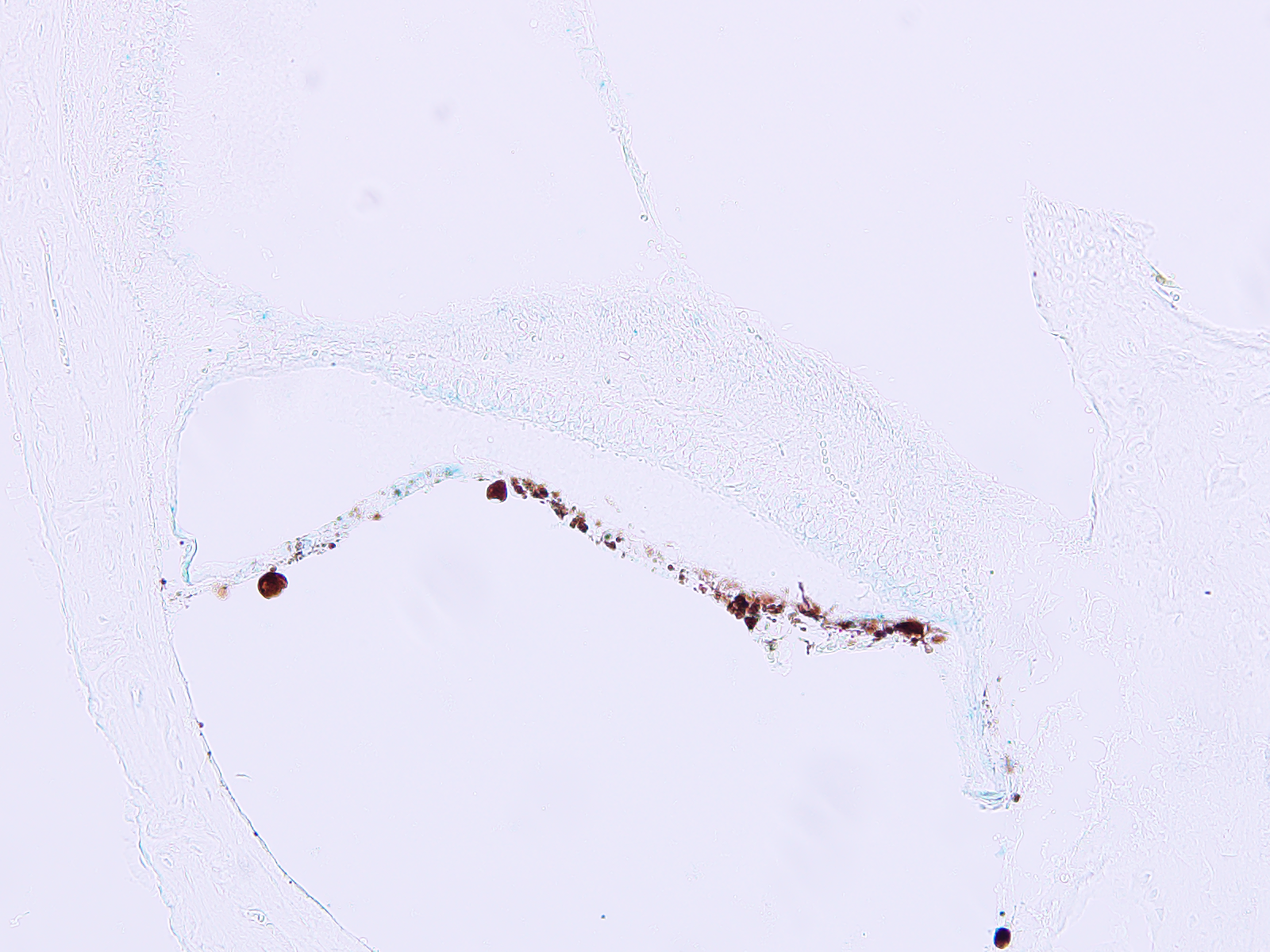

Supplement: Supplementary file 7 — Supporting File 7: advs75263‐sup‐0007‐Data5.zip. [file ADVS-13-e12538-s004.zip › Raw data of microscope images/Figure 7G-wt Macula.tif]

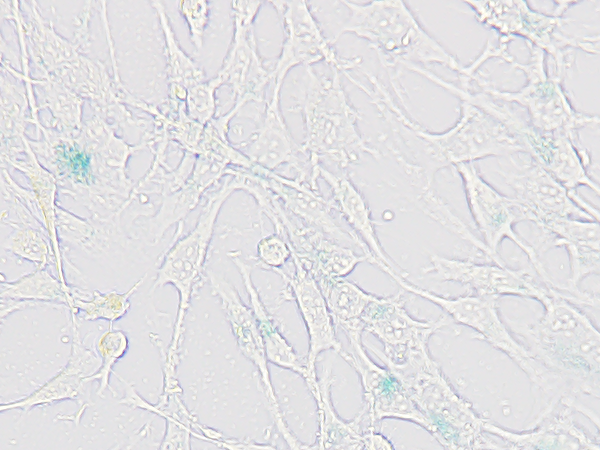

Supplement: Supplementary file 7 — Supporting File 7: advs75263‐sup‐0007‐Data5.zip. [file ADVS-13-e12538-s004.zip › Raw data of microscope images/Figure S10A-siHtrA1.tif]

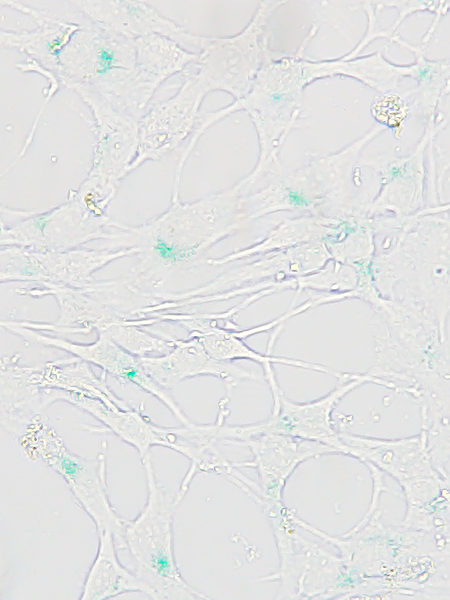

Supplement: Supplementary file 7 — Supporting File 7: advs75263‐sup‐0007‐Data5.zip. [file ADVS-13-e12538-s004.zip › Raw data of microscope images/Figure S10A-siNC.tif]

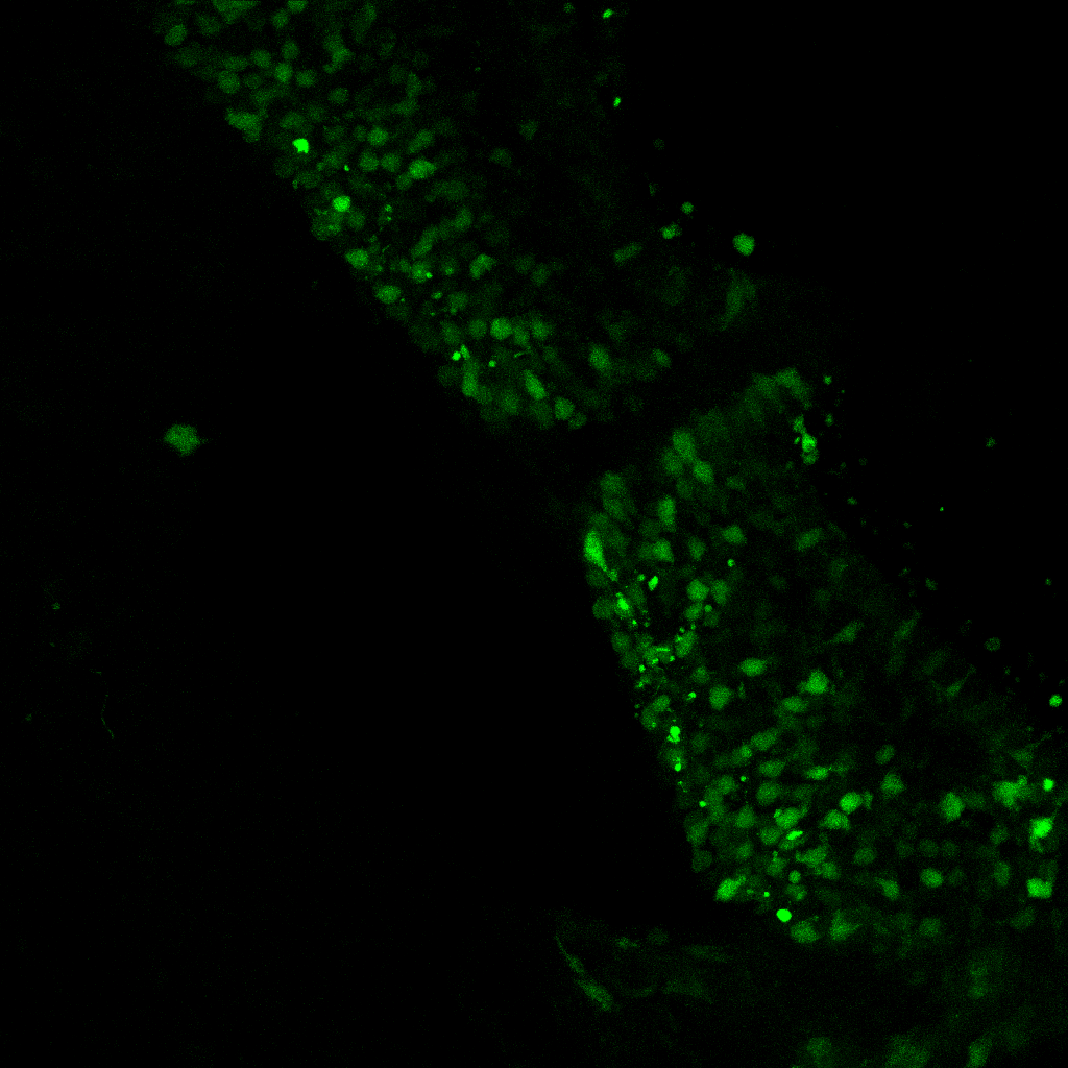

Supplement: Supplementary file 7 — Supporting File 7: advs75263‐sup‐0007‐Data5.zip. [file ADVS-13-e12538-s004.zip › Raw data of microscope images/Figure S11-Ampulla (1) EGFP.tif]

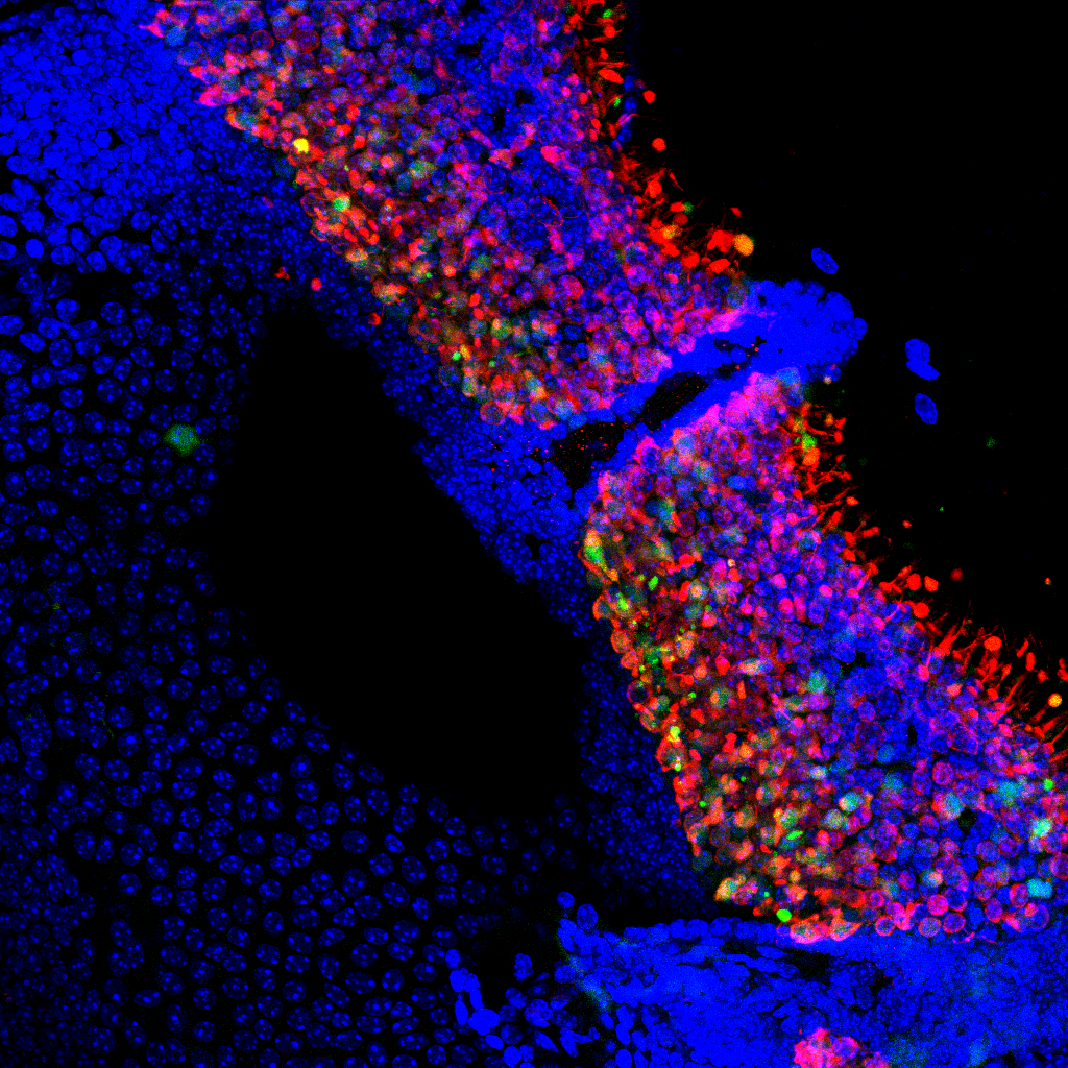

Supplement: Supplementary file 7 — Supporting File 7: advs75263‐sup‐0007‐Data5.zip. [file ADVS-13-e12538-s004.zip › Raw data of microscope images/Figure S11-Ampulla (2) Merge.tif]

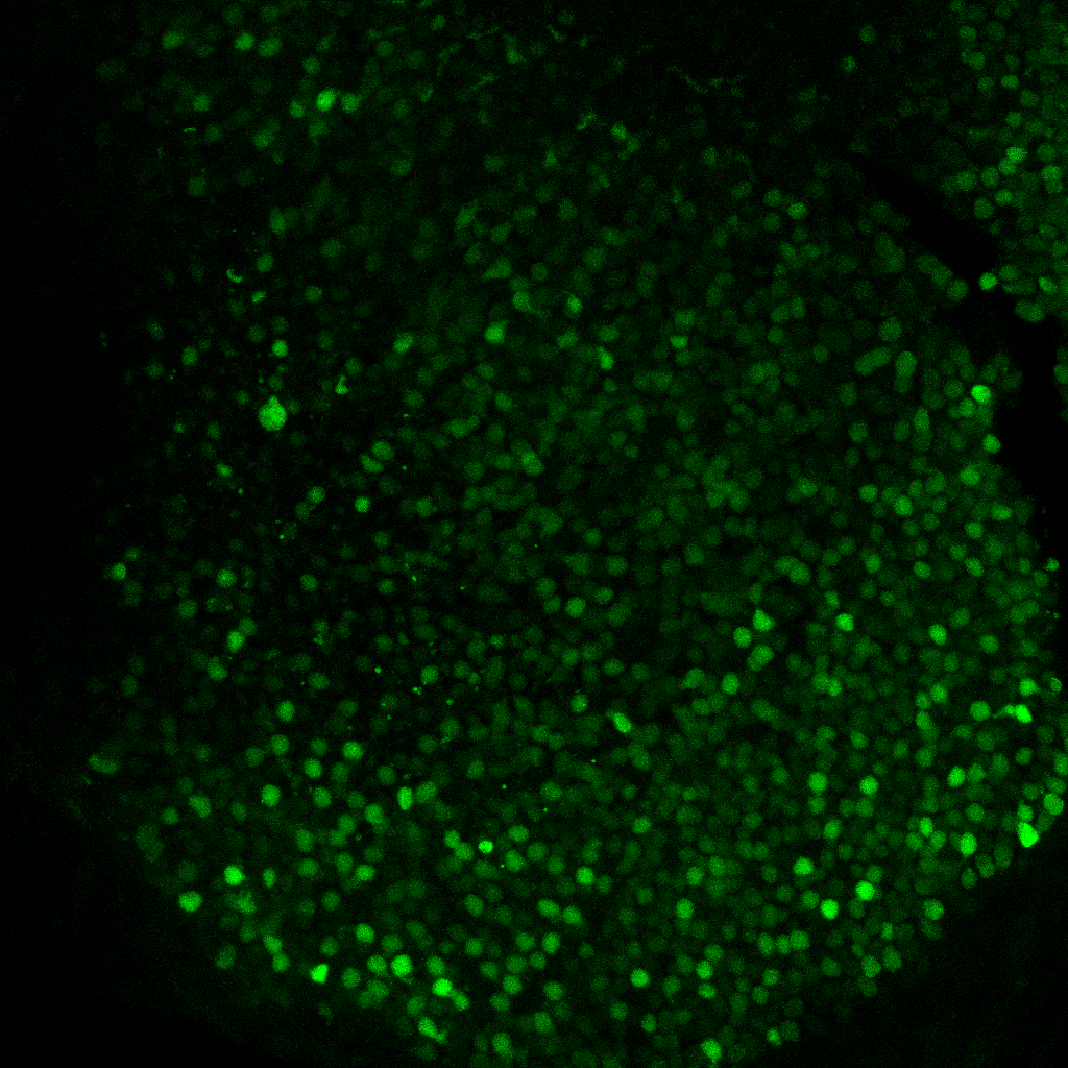

Supplement: Supplementary file 7 — Supporting File 7: advs75263‐sup‐0007‐Data5.zip. [file ADVS-13-e12538-s004.zip › Raw data of microscope images/Figure S11-Macula (1) EGFP.tif]

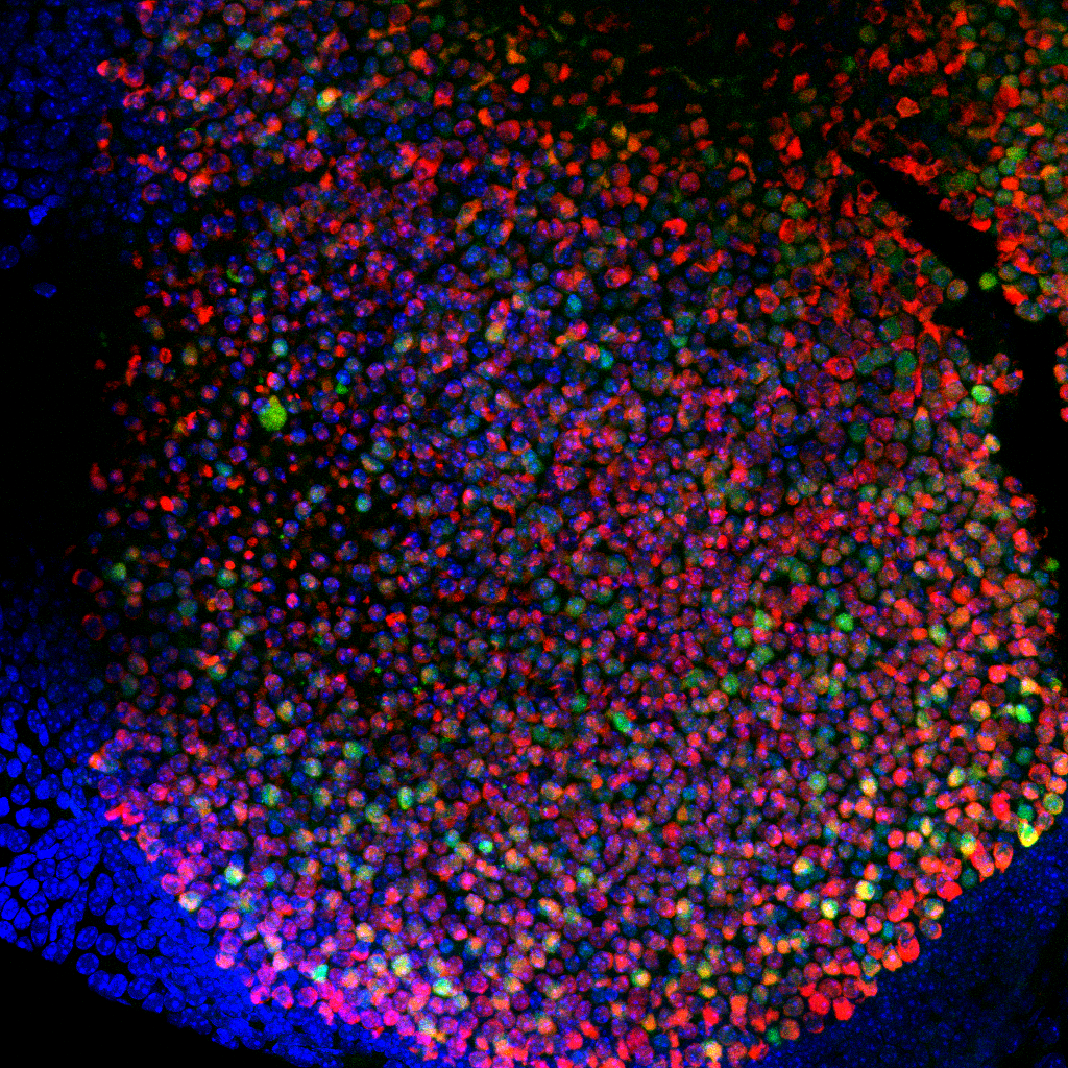

Supplement: Supplementary file 7 — Supporting File 7: advs75263‐sup‐0007‐Data5.zip. [file ADVS-13-e12538-s004.zip › Raw data of microscope images/Figure S11-Macula (2) Merge.tif]

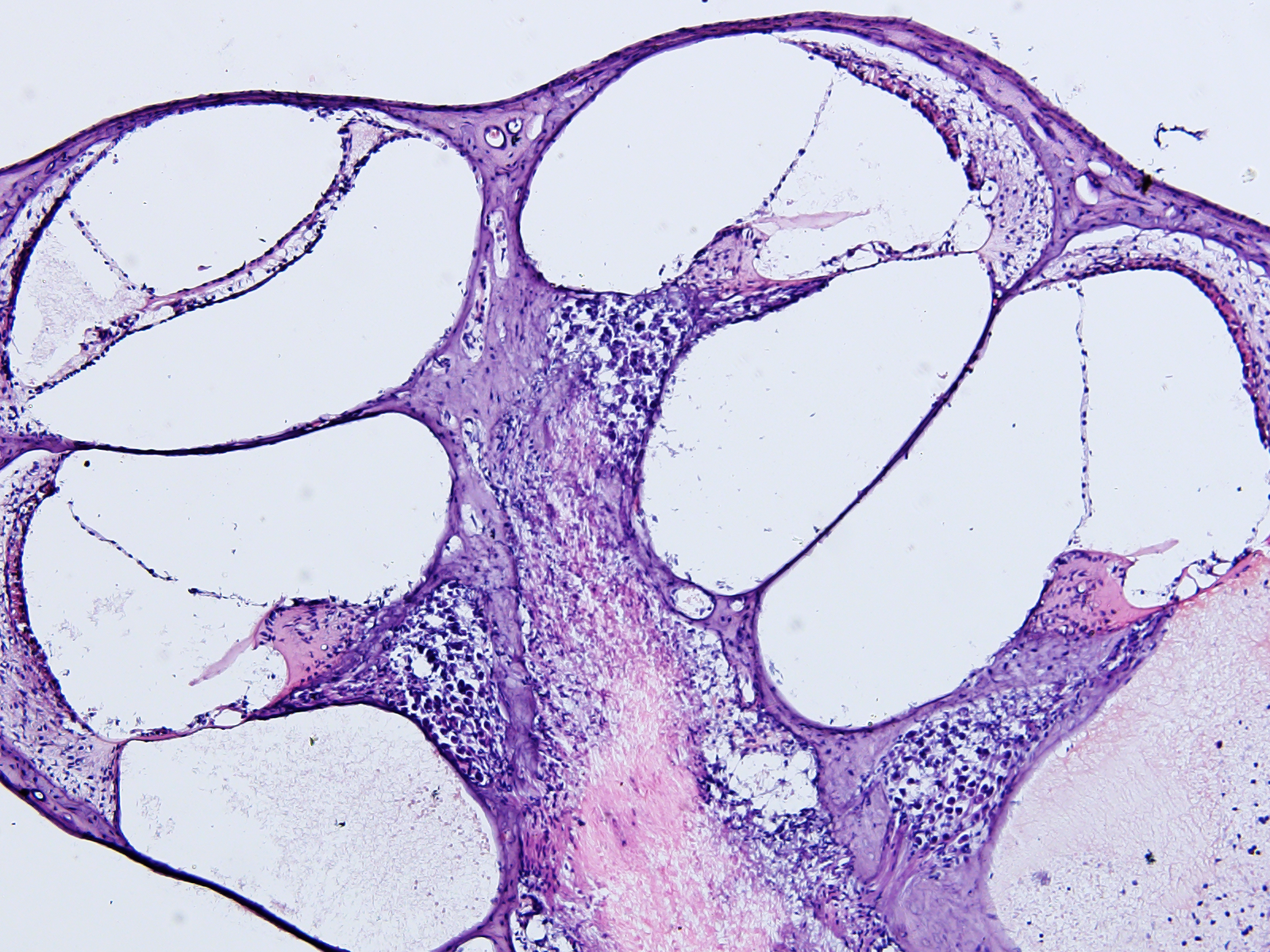

Supplement: Supplementary file 7 — Supporting File 7: advs75263‐sup‐0007‐Data5.zip. [file ADVS-13-e12538-s004.zip › Raw data of microscope images/Figure S12B-LPS+Gal.tif]

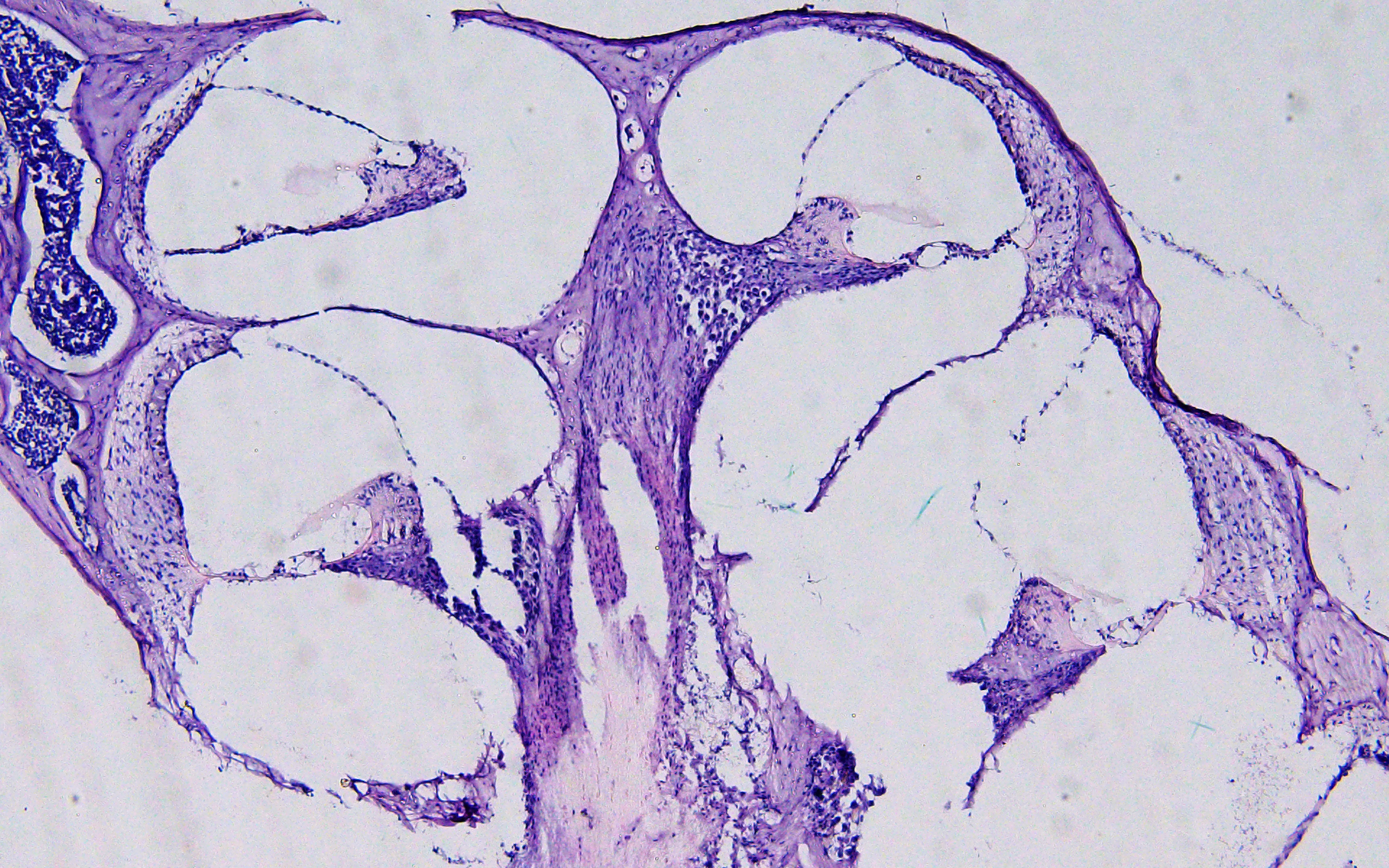

Supplement: Supplementary file 7 — Supporting File 7: advs75263‐sup‐0007‐Data5.zip. [file ADVS-13-e12538-s004.zip › Raw data of microscope images/Figure S12B-LPS.tif]

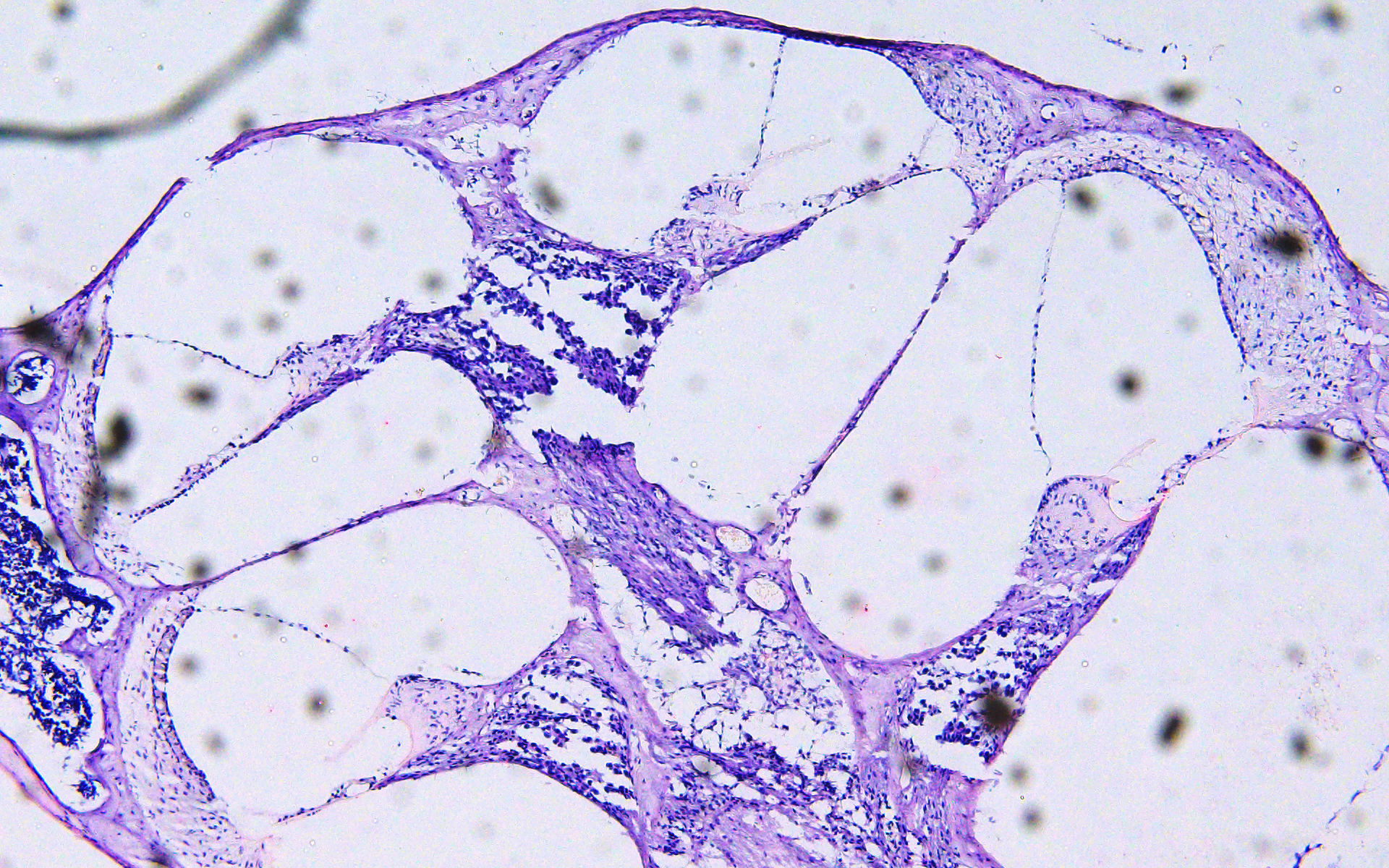

Supplement: Supplementary file 7 — Supporting File 7: advs75263‐sup‐0007‐Data5.zip. [file ADVS-13-e12538-s004.zip › Raw data of microscope images/Figure S12B-NS.tif]

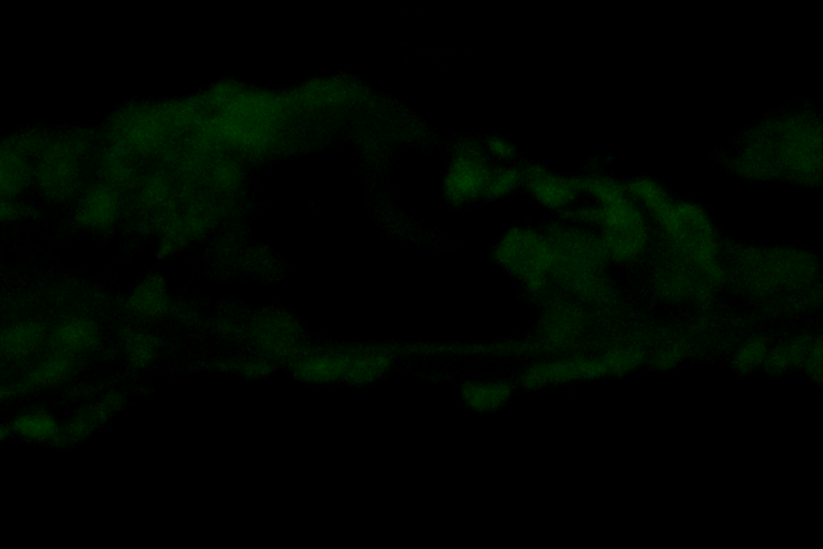

Supplement: Supplementary file 7 — Supporting File 7: advs75263‐sup‐0007‐Data5.zip. [file ADVS-13-e12538-s004.zip › Raw data of microscope images/Figure S13C-ACY.tif]

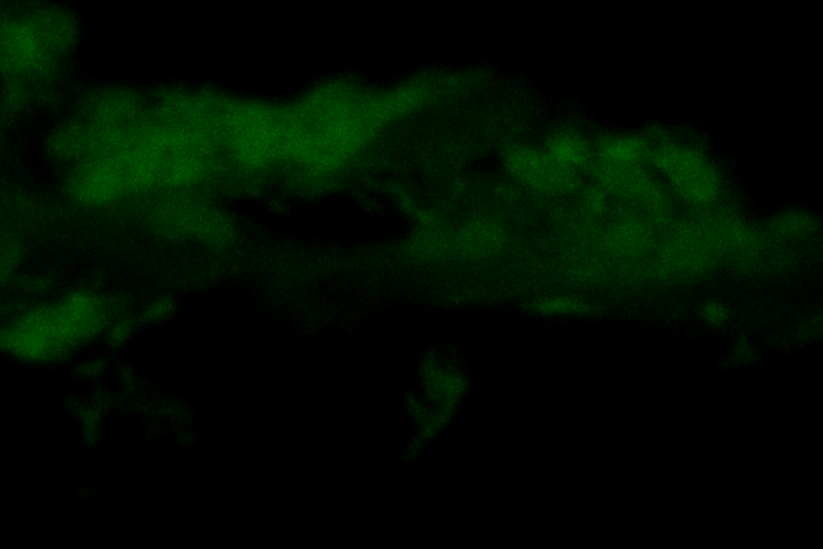

Supplement: Supplementary file 7 — Supporting File 7: advs75263‐sup‐0007‐Data5.zip. [file ADVS-13-e12538-s004.zip › Raw data of microscope images/Figure S13C-LPS+ACY.tif]

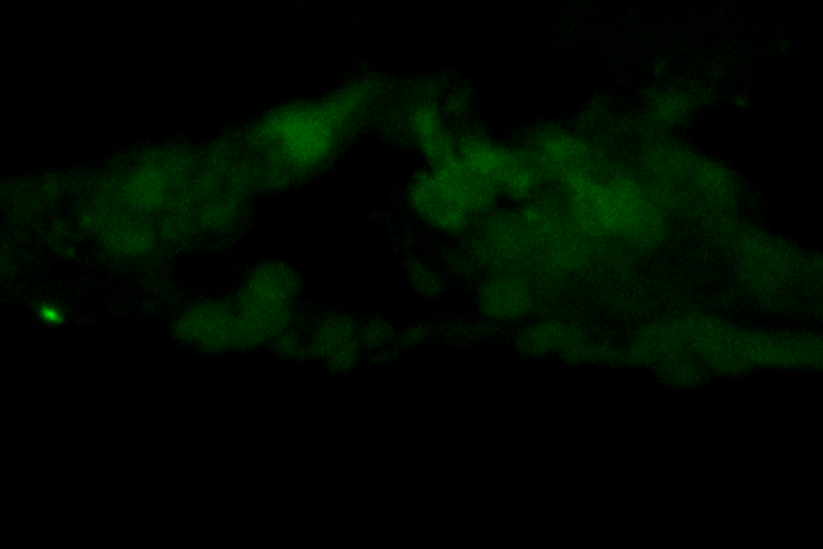

Supplement: Supplementary file 7 — Supporting File 7: advs75263‐sup‐0007‐Data5.zip. [file ADVS-13-e12538-s004.zip › Raw data of microscope images/Figure S13C-LPS.tif]

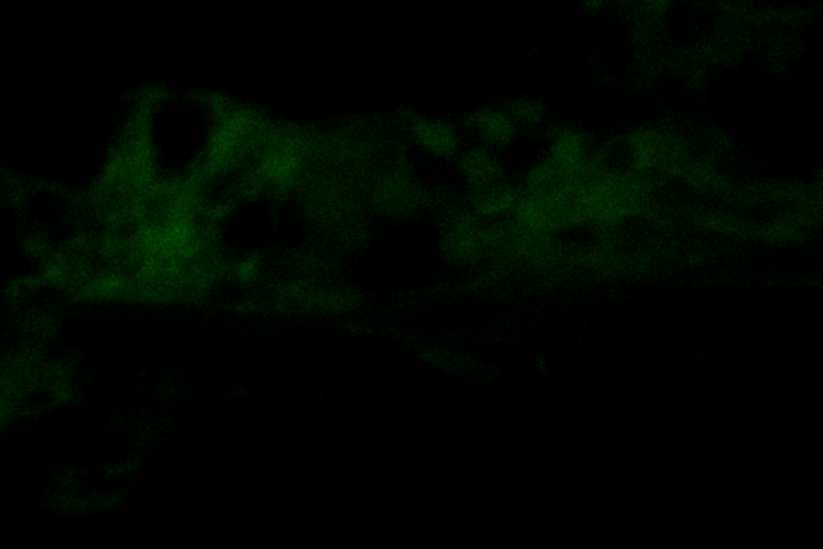

Supplement: Supplementary file 7 — Supporting File 7: advs75263‐sup‐0007‐Data5.zip. [file ADVS-13-e12538-s004.zip › Raw data of microscope images/Figure S13C-NS.tif]

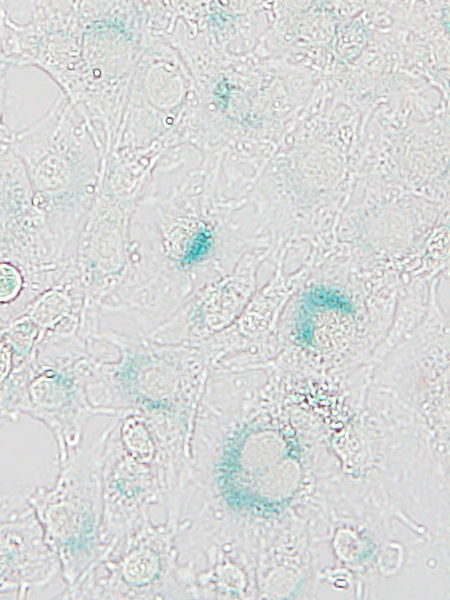

Supplement: Supplementary file 7 — Supporting File 7: advs75263‐sup‐0007‐Data5.zip. [file ADVS-13-e12538-s004.zip › Raw data of microscope images/Figure S13E-siHtrA1.tif]

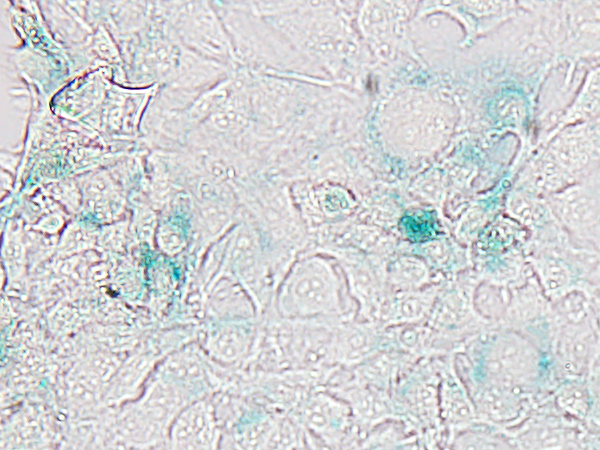

Supplement: Supplementary file 7 — Supporting File 7: advs75263‐sup‐0007‐Data5.zip. [file ADVS-13-e12538-s004.zip › Raw data of microscope images/Figure S13E-siNC.tif]

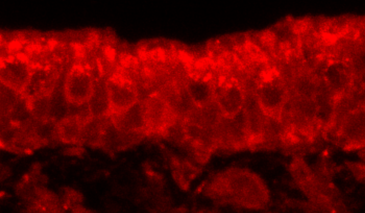

Supplement: Supplementary file 7 — Supporting File 7: advs75263‐sup‐0007‐Data5.zip. [file ADVS-13-e12538-s004.zip › Raw data of microscope images/Figure S1A-MD (1) DHE.tif]

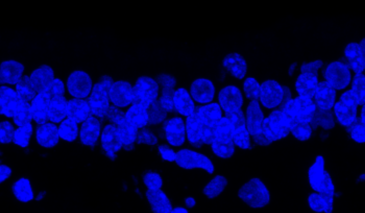

Supplement: Supplementary file 7 — Supporting File 7: advs75263‐sup‐0007‐Data5.zip. [file ADVS-13-e12538-s004.zip › Raw data of microscope images/Figure S1A-MD (2) DAPI.tif]

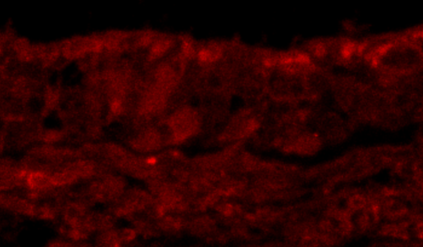

Supplement: Supplementary file 7 — Supporting File 7: advs75263‐sup‐0007‐Data5.zip. [file ADVS-13-e12538-s004.zip › Raw data of microscope images/Figure S1A-VS (1) DHE.tif]

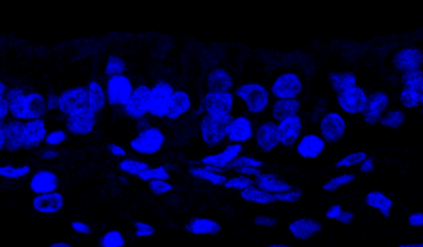

Supplement: Supplementary file 7 — Supporting File 7: advs75263‐sup‐0007‐Data5.zip. [file ADVS-13-e12538-s004.zip › Raw data of microscope images/Figure S1A-VS (2) DAPI.tif]

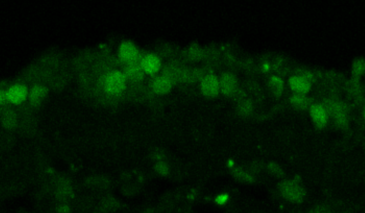

Supplement: Supplementary file 7 — Supporting File 7: advs75263‐sup‐0007‐Data5.zip. [file ADVS-13-e12538-s004.zip › Raw data of microscope images/Figure S1B-MD (1) γH2AX.tif]

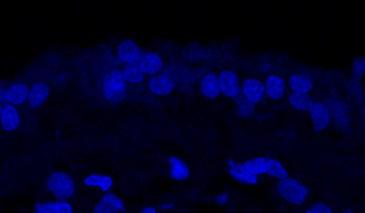

Supplement: Supplementary file 7 — Supporting File 7: advs75263‐sup‐0007‐Data5.zip. [file ADVS-13-e12538-s004.zip › Raw data of microscope images/Figure S1B-MD (2) DAPI.tif]

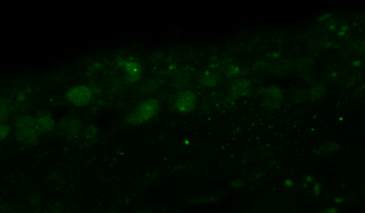

Supplement: Supplementary file 7 — Supporting File 7: advs75263‐sup‐0007‐Data5.zip. [file ADVS-13-e12538-s004.zip › Raw data of microscope images/Figure S1B-VS (1) γH2AX.tif]

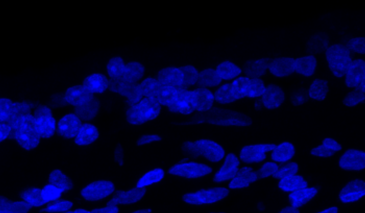

Supplement: Supplementary file 7 — Supporting File 7: advs75263‐sup‐0007‐Data5.zip. [file ADVS-13-e12538-s004.zip › Raw data of microscope images/Figure S1B-VS (2) DAPI.tif]

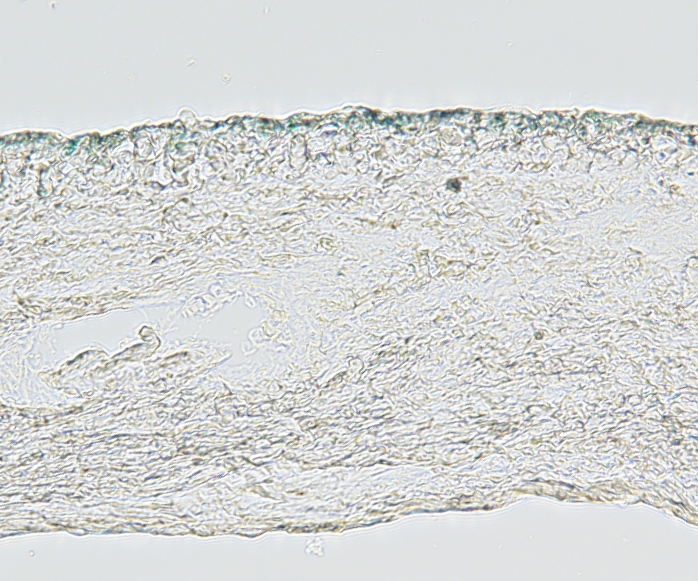

Supplement: Supplementary file 7 — Supporting File 7: advs75263‐sup‐0007‐Data5.zip. [file ADVS-13-e12538-s004.zip › Raw data of microscope images/Figure S1C-MD Gal.tif]

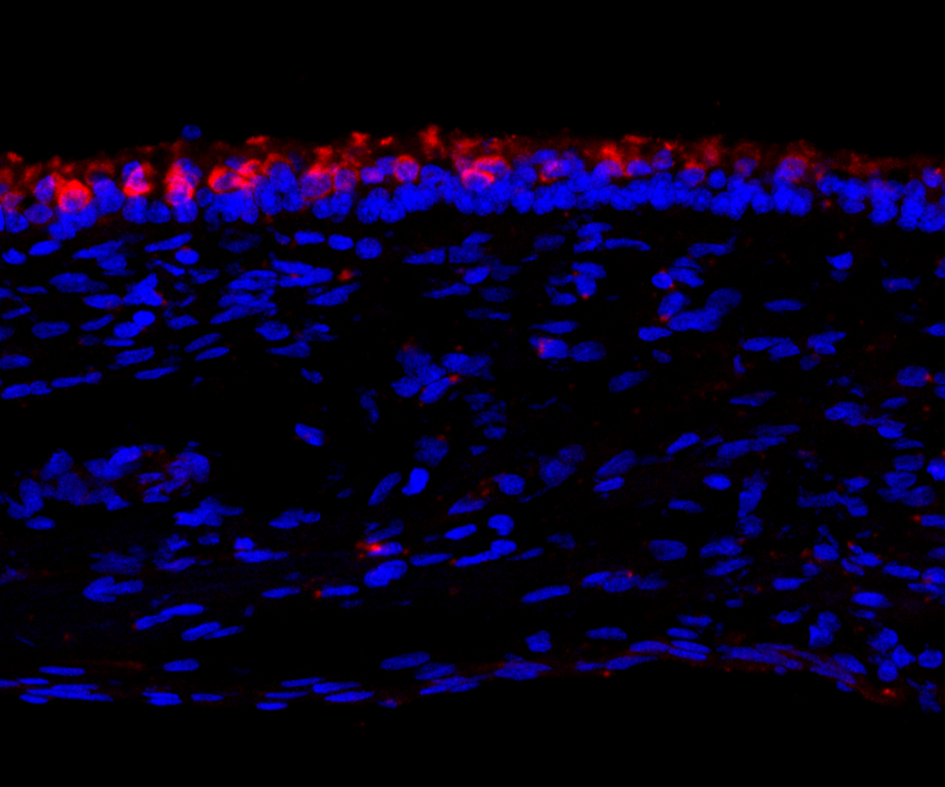

Supplement: Supplementary file 7 — Supporting File 7: advs75263‐sup‐0007‐Data5.zip. [file ADVS-13-e12538-s004.zip › Raw data of microscope images/Figure S1C-MD.tif]

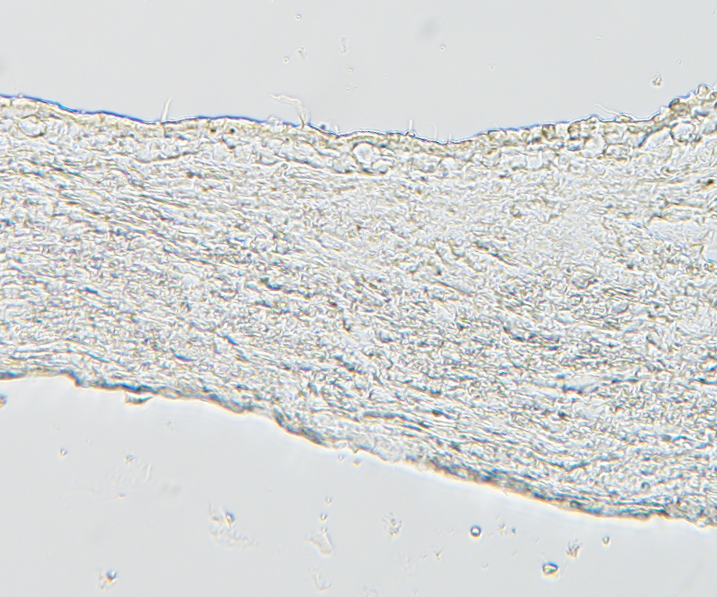

Supplement: Supplementary file 7 — Supporting File 7: advs75263‐sup‐0007‐Data5.zip. [file ADVS-13-e12538-s004.zip › Raw data of microscope images/Figure S1C-VS Gal.tif]

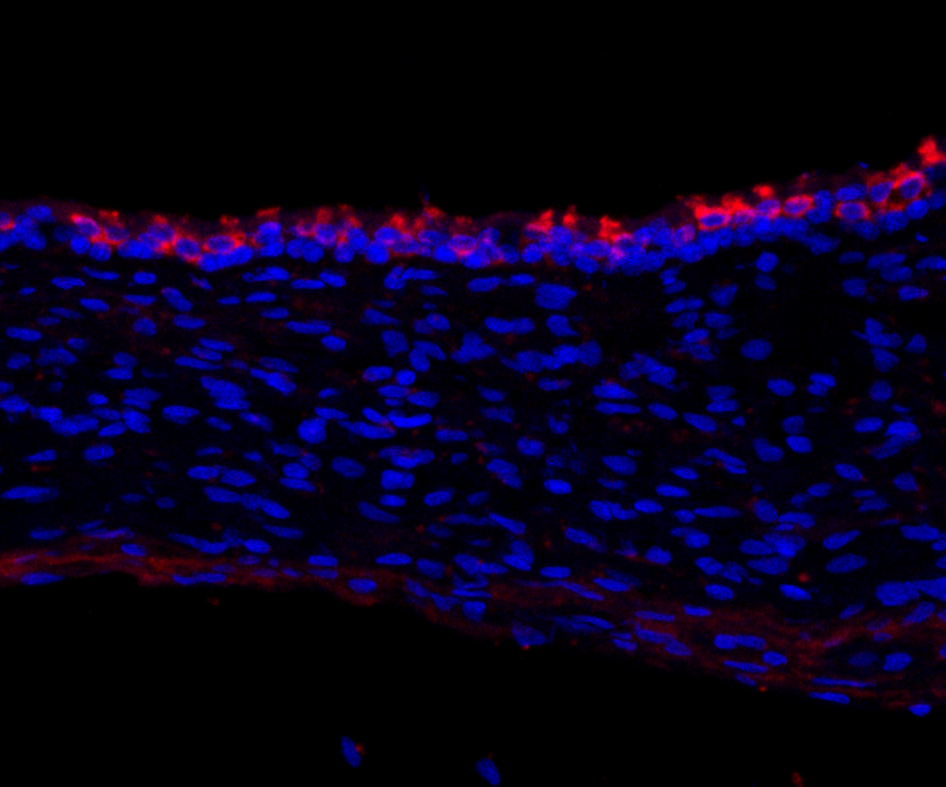

Supplement: Supplementary file 7 — Supporting File 7: advs75263‐sup‐0007‐Data5.zip. [file ADVS-13-e12538-s004.zip › Raw data of microscope images/Figure S1C-VS.tif]

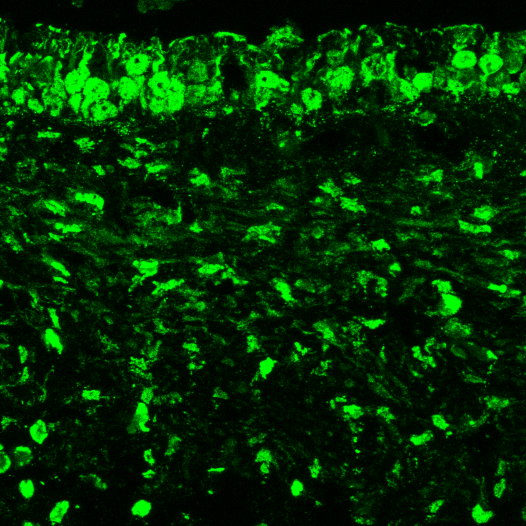

Supplement: Supplementary file 7 — Supporting File 7: advs75263‐sup‐0007‐Data5.zip. [file ADVS-13-e12538-s004.zip › Raw data of microscope images/Figure S1D-MD (1) P16.tif]

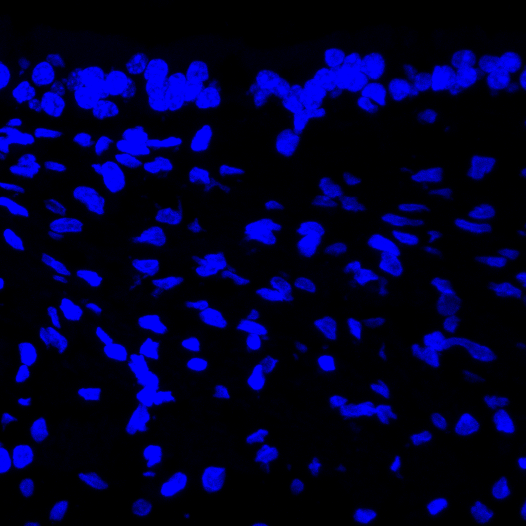

Supplement: Supplementary file 7 — Supporting File 7: advs75263‐sup‐0007‐Data5.zip. [file ADVS-13-e12538-s004.zip › Raw data of microscope images/Figure S1D-MD (2) DAPI.tif]

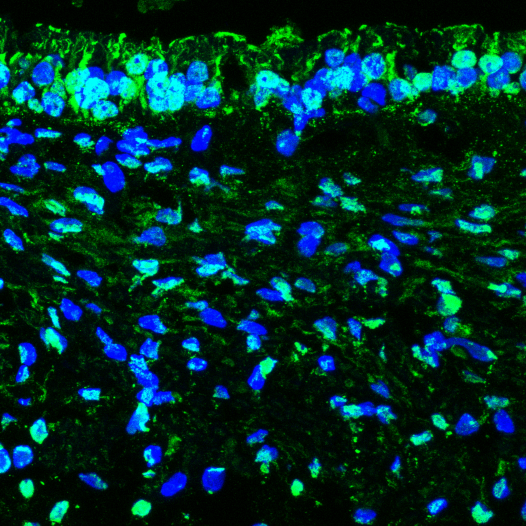

Supplement: Supplementary file 7 — Supporting File 7: advs75263‐sup‐0007‐Data5.zip. [file ADVS-13-e12538-s004.zip › Raw data of microscope images/Figure S1D-MD (3) Merge.tif]

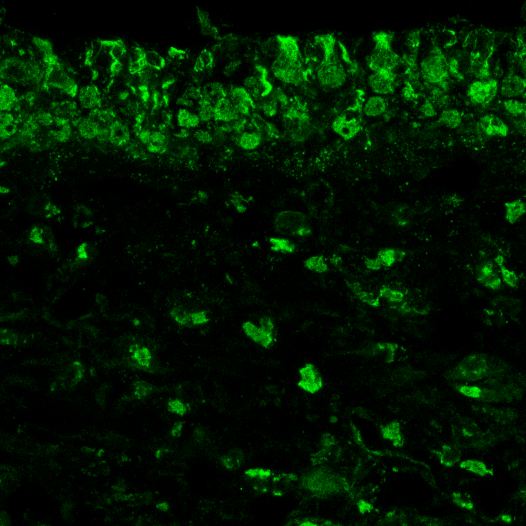

Supplement: Supplementary file 7 — Supporting File 7: advs75263‐sup‐0007‐Data5.zip. [file ADVS-13-e12538-s004.zip › Raw data of microscope images/Figure S1D-VS (1) P16.tif]

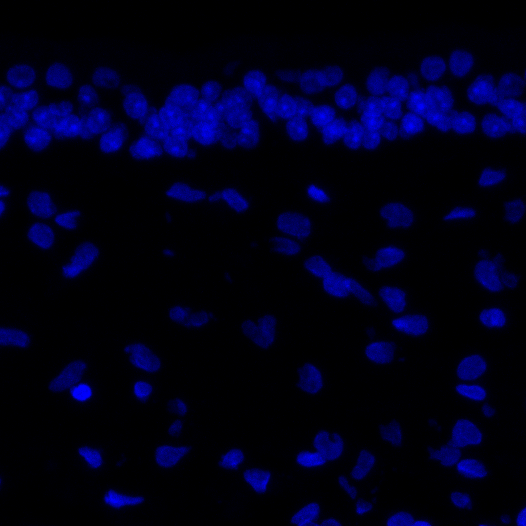

Supplement: Supplementary file 7 — Supporting File 7: advs75263‐sup‐0007‐Data5.zip. [file ADVS-13-e12538-s004.zip › Raw data of microscope images/Figure S1D-VS (2) DAPI.tif]

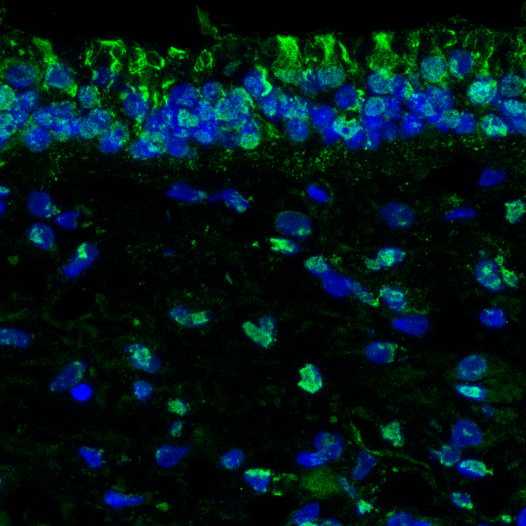

Supplement: Supplementary file 7 — Supporting File 7: advs75263‐sup‐0007‐Data5.zip. [file ADVS-13-e12538-s004.zip › Raw data of microscope images/Figure S1D-VS (3) Merge.tif]

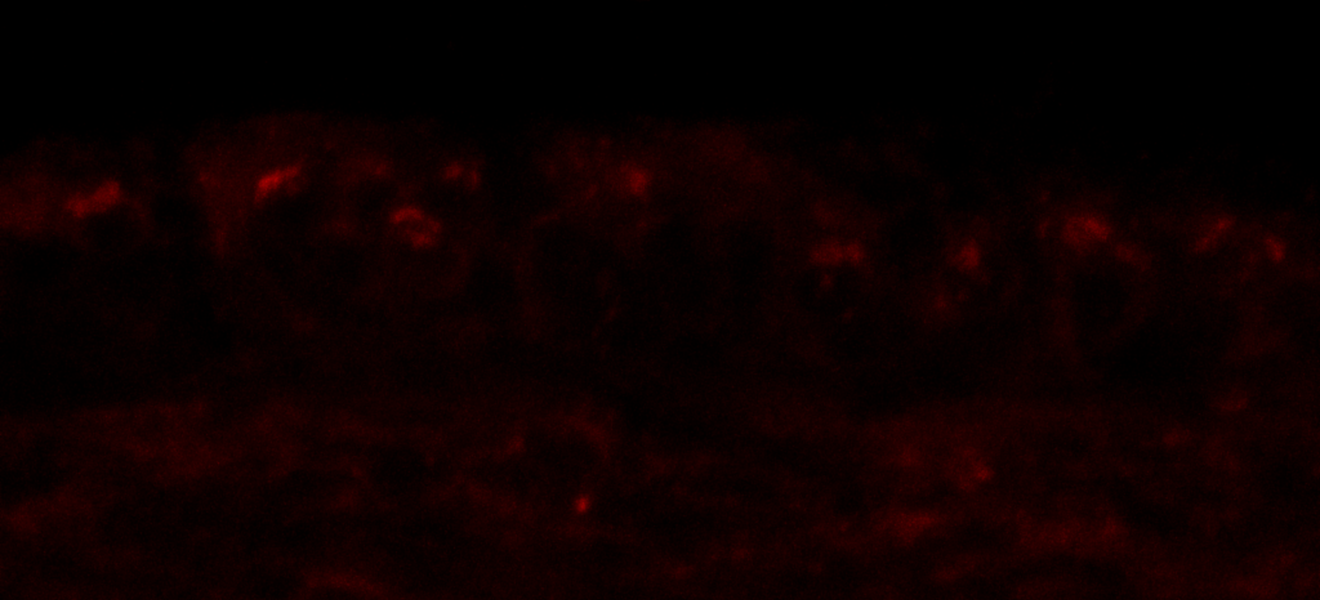

Supplement: Supplementary file 7 — Supporting File 7: advs75263‐sup‐0007‐Data5.zip. [file ADVS-13-e12538-s004.zip › Raw data of microscope images/Figure S1F-MD (1) pNFκB.tif]

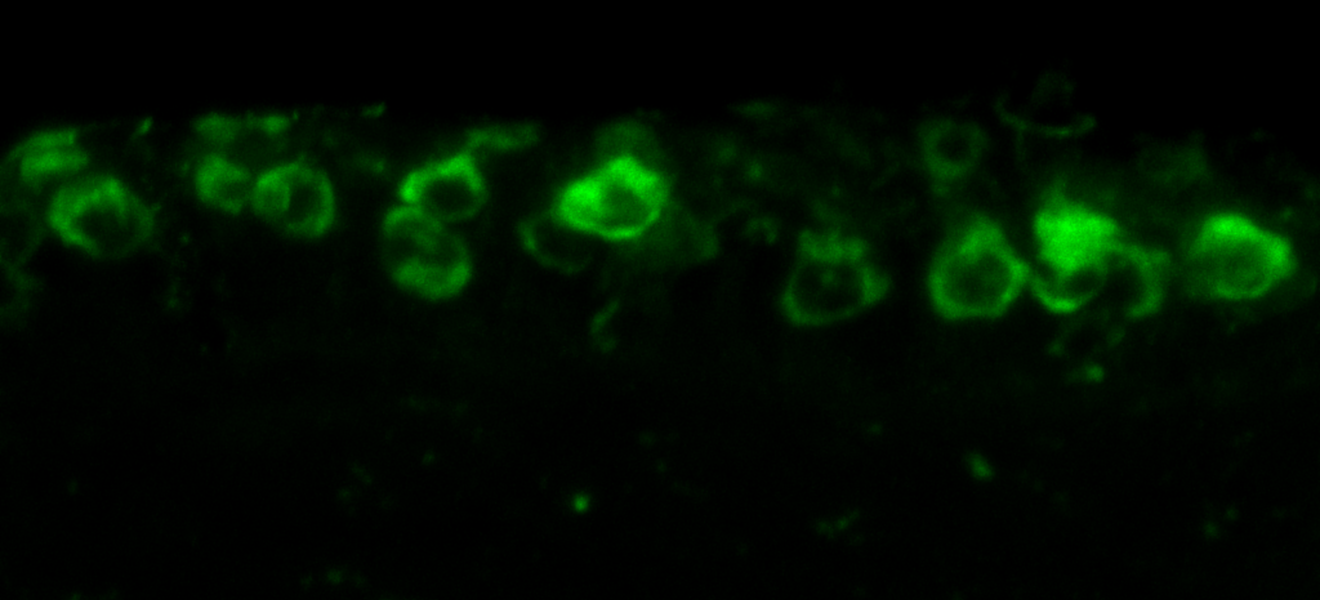

Supplement: Supplementary file 7 — Supporting File 7: advs75263‐sup‐0007‐Data5.zip. [file ADVS-13-e12538-s004.zip › Raw data of microscope images/Figure S1F-MD (2) Myo7a.tif]
