# Supplementary figures and images for: GATA4‐Driven Transcription of HtrA1 Promotes Cellular Senescence in Ménière's Disease and Age‐Related Audio‐Vestibular Dysfunction (part 2 of 2)
Source: Adv Sci (Weinh). 2026 Apr 14;13(39):e12538. doi: 10.1002/advs.202512538 (PMC13334991; doi:10.1002/advs.202512538)

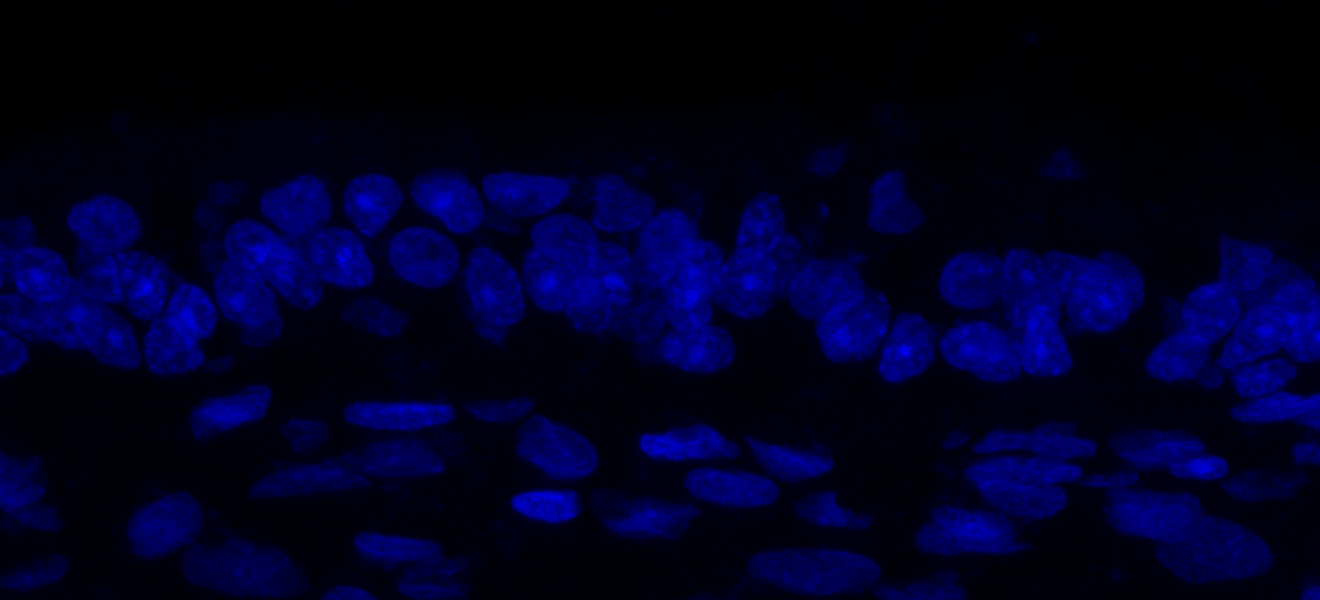

Supplement: Supplementary file 7 — Supporting File 7: advs75263‐sup‐0007‐Data5.zip. [file ADVS-13-e12538-s004.zip › Raw data of microscope images/Figure S1F-MD (3).tif]

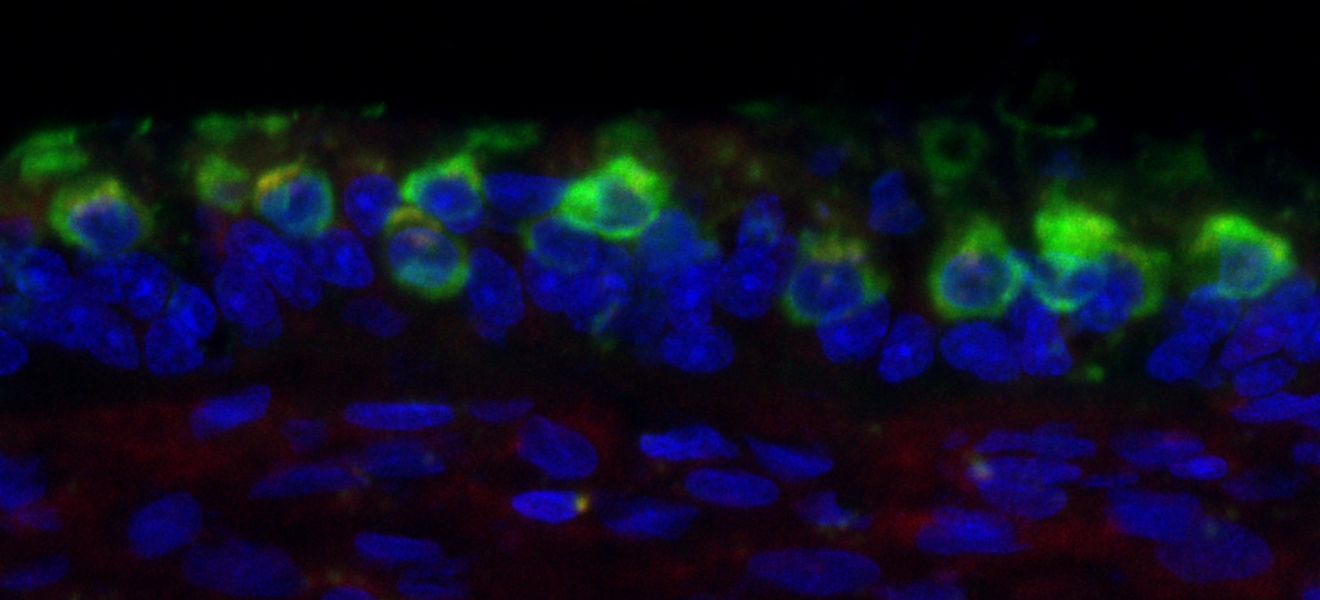

Supplement: Supplementary file 7 — Supporting File 7: advs75263‐sup‐0007‐Data5.zip. [file ADVS-13-e12538-s004.zip › Raw data of microscope images/Figure S1F-MD (4) Merge.tif]

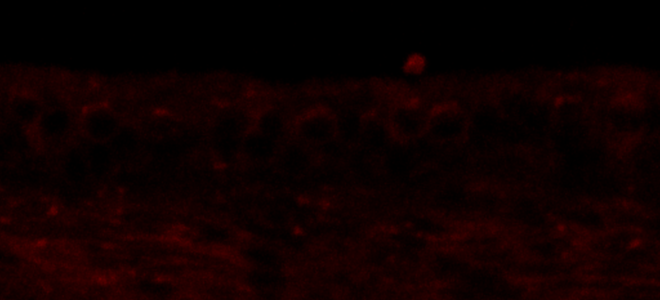

Supplement: Supplementary file 7 — Supporting File 7: advs75263‐sup‐0007‐Data5.zip. [file ADVS-13-e12538-s004.zip › Raw data of microscope images/Figure S1F-VS (1) pNFκB.tif]

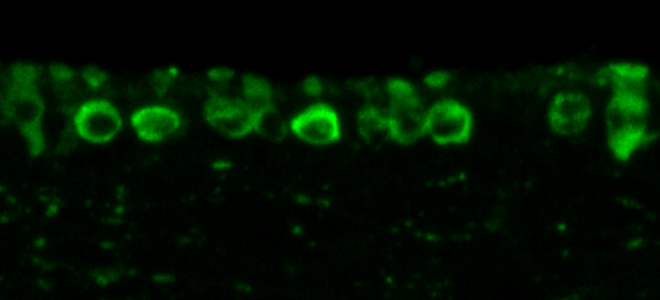

Supplement: Supplementary file 7 — Supporting File 7: advs75263‐sup‐0007‐Data5.zip. [file ADVS-13-e12538-s004.zip › Raw data of microscope images/Figure S1F-VS (2) Myo7a.tif]

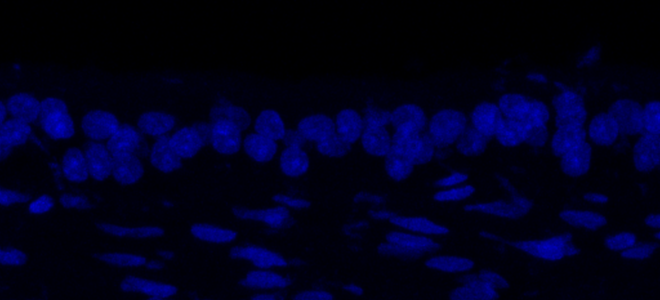

Supplement: Supplementary file 7 — Supporting File 7: advs75263‐sup‐0007‐Data5.zip. [file ADVS-13-e12538-s004.zip › Raw data of microscope images/Figure S1F-VS (3).tif]

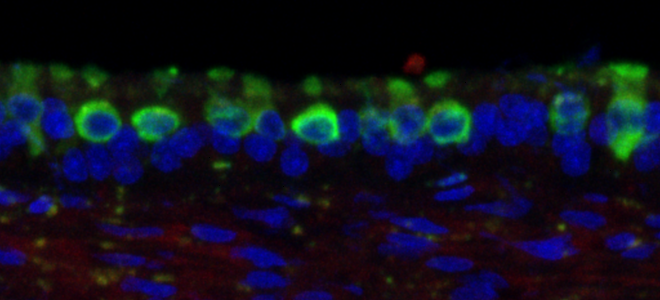

Supplement: Supplementary file 7 — Supporting File 7: advs75263‐sup‐0007‐Data5.zip. [file ADVS-13-e12538-s004.zip › Raw data of microscope images/Figure S1F-VS (4) Merge.tif]

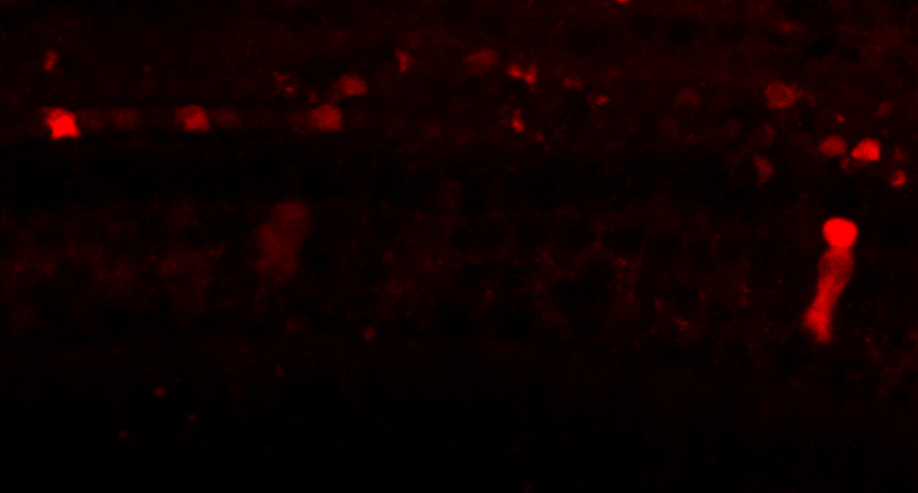

Supplement: Supplementary file 7 — Supporting File 7: advs75263‐sup‐0007‐Data5.zip. [file ADVS-13-e12538-s004.zip › Raw data of microscope images/Figure S1I-MD (1) pNFκB.tif]

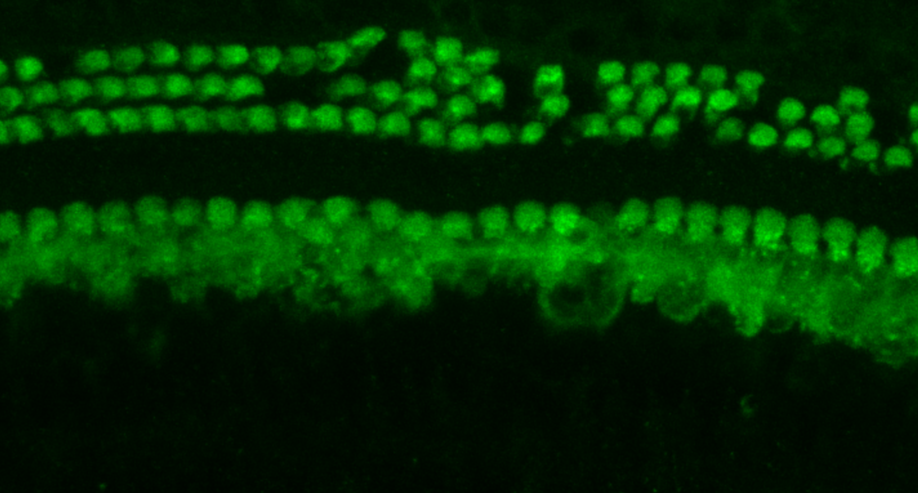

Supplement: Supplementary file 7 — Supporting File 7: advs75263‐sup‐0007‐Data5.zip. [file ADVS-13-e12538-s004.zip › Raw data of microscope images/Figure S1I-MD (2) Myo7a.tif]

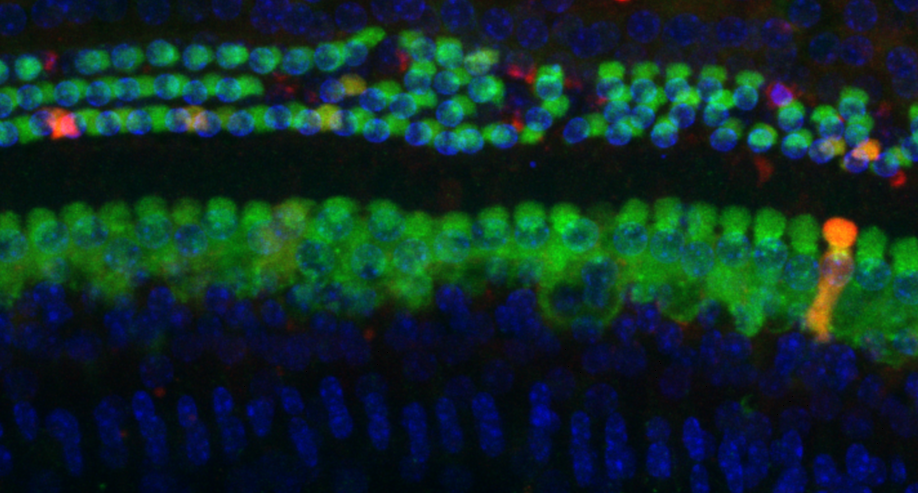

Supplement: Supplementary file 7 — Supporting File 7: advs75263‐sup‐0007‐Data5.zip. [file ADVS-13-e12538-s004.zip › Raw data of microscope images/Figure S1I-MD (3) Merge.tif]

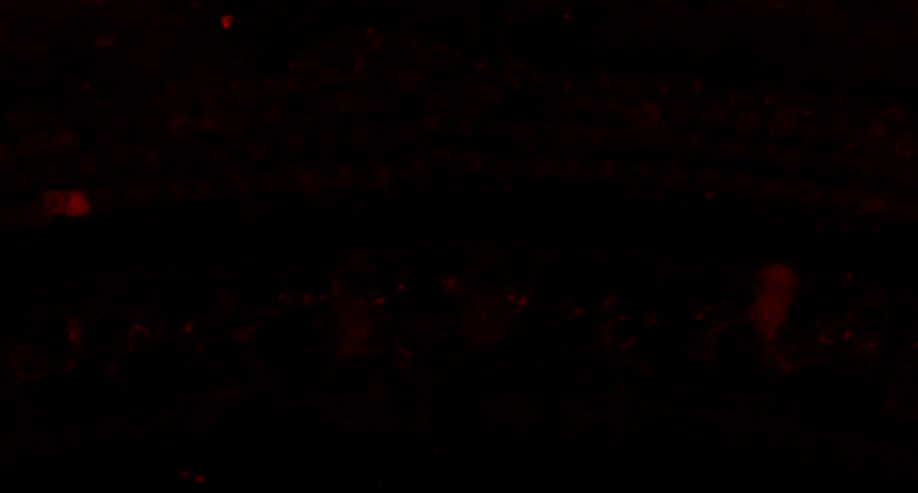

Supplement: Supplementary file 7 — Supporting File 7: advs75263‐sup‐0007‐Data5.zip. [file ADVS-13-e12538-s004.zip › Raw data of microscope images/Figure S1I-VS (1) pNFκB.tif]

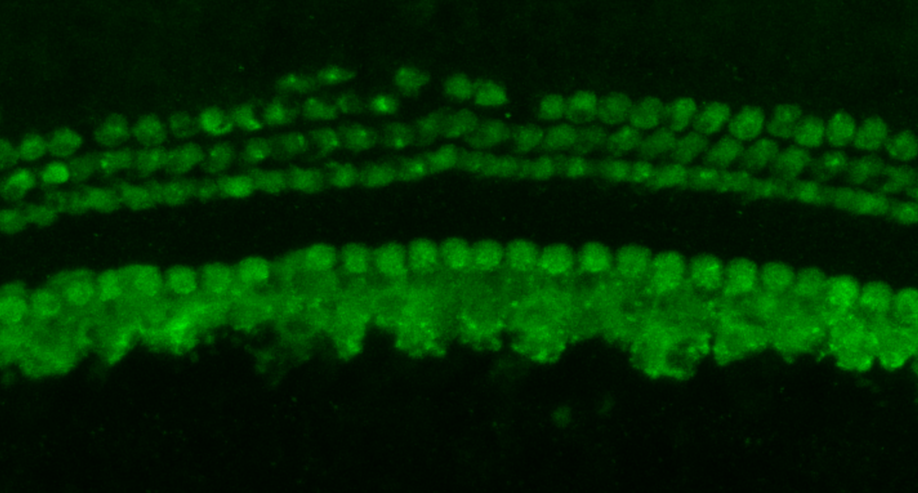

Supplement: Supplementary file 7 — Supporting File 7: advs75263‐sup‐0007‐Data5.zip. [file ADVS-13-e12538-s004.zip › Raw data of microscope images/Figure S1I-VS (2) Myo7a.tif]

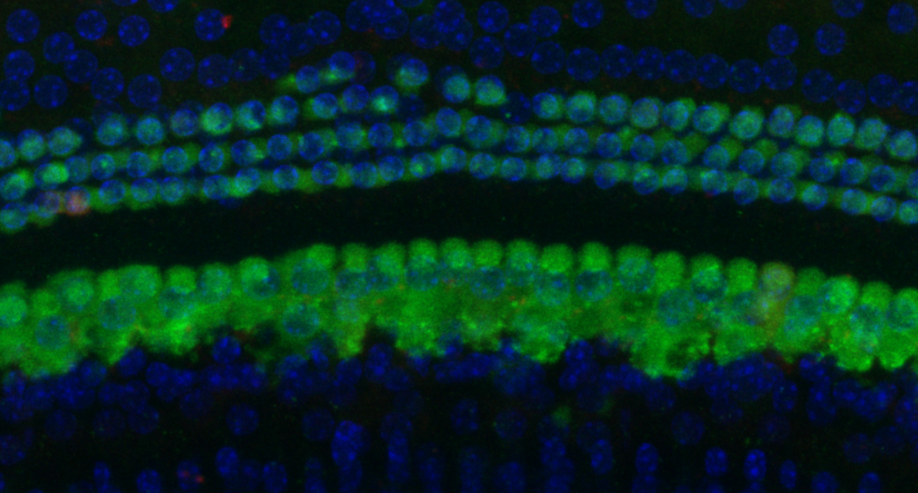

Supplement: Supplementary file 7 — Supporting File 7: advs75263‐sup‐0007‐Data5.zip. [file ADVS-13-e12538-s004.zip › Raw data of microscope images/Figure S1I-VS (3) Merge.tif]

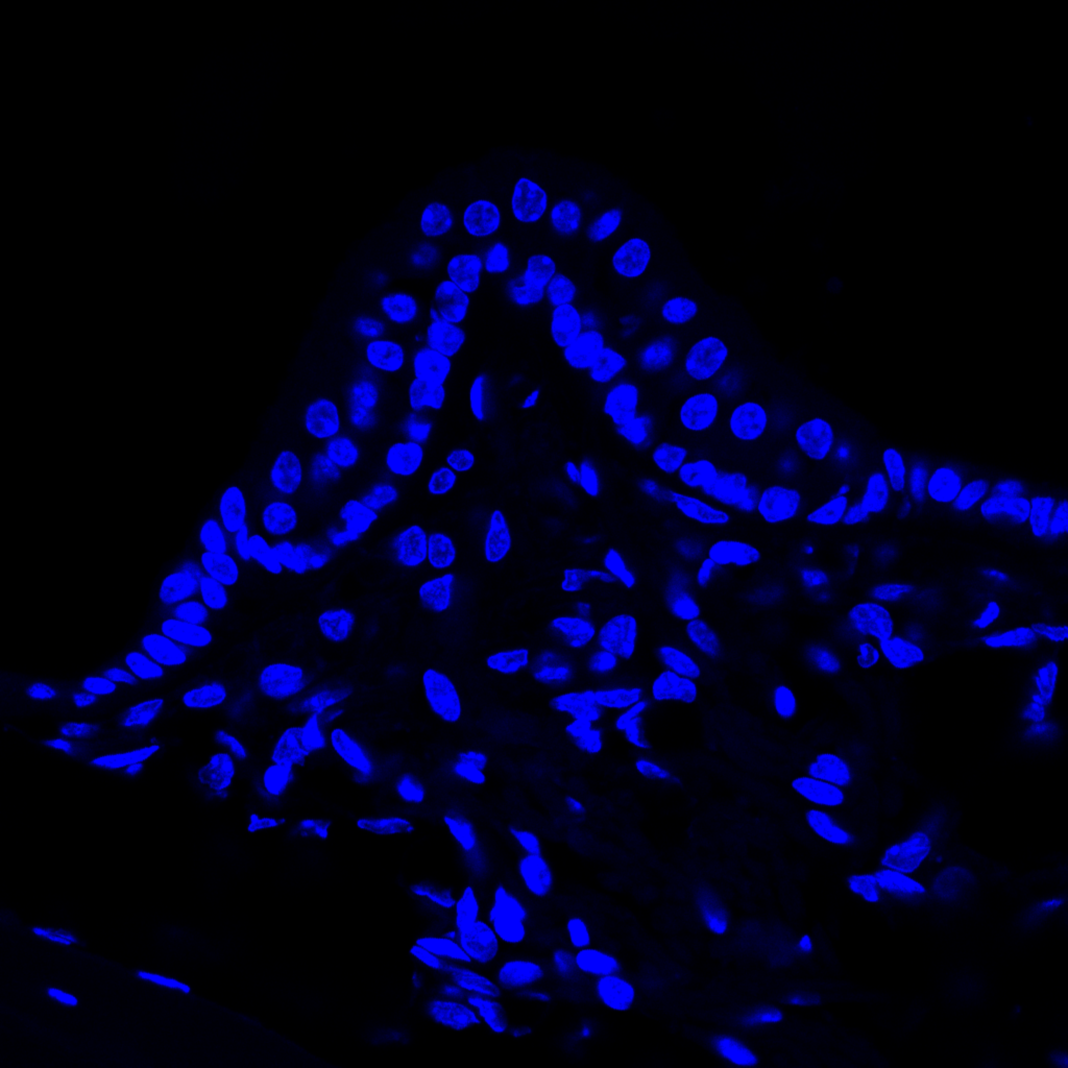

Supplement: Supplementary file 7 — Supporting File 7: advs75263‐sup‐0007‐Data5.zip. [file ADVS-13-e12538-s004.zip › Raw data of microscope images/Figure S2A-KO (1) DAPI.tif]

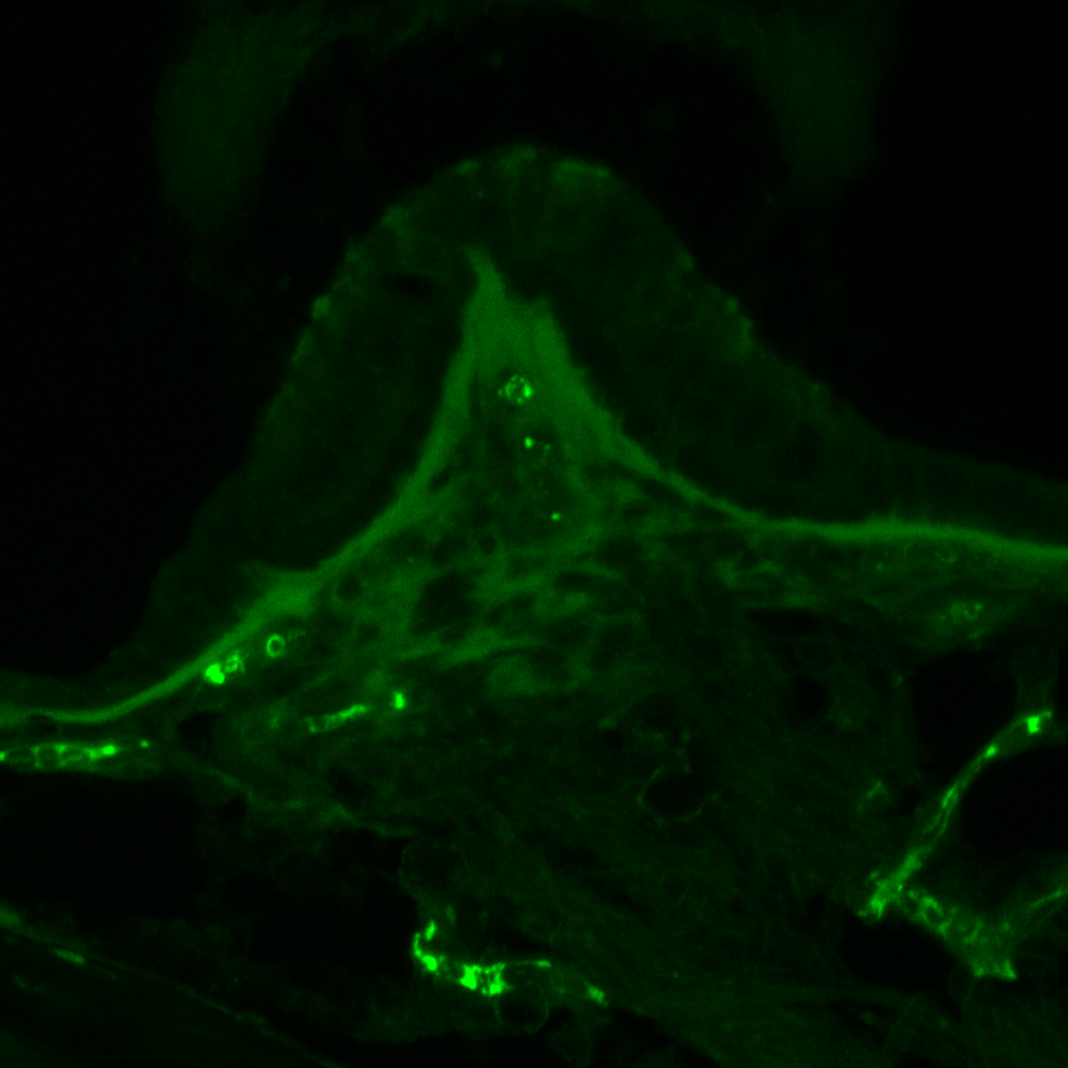

Supplement: Supplementary file 7 — Supporting File 7: advs75263‐sup‐0007‐Data5.zip. [file ADVS-13-e12538-s004.zip › Raw data of microscope images/Figure S2A-KO (2) GATA4.tif]

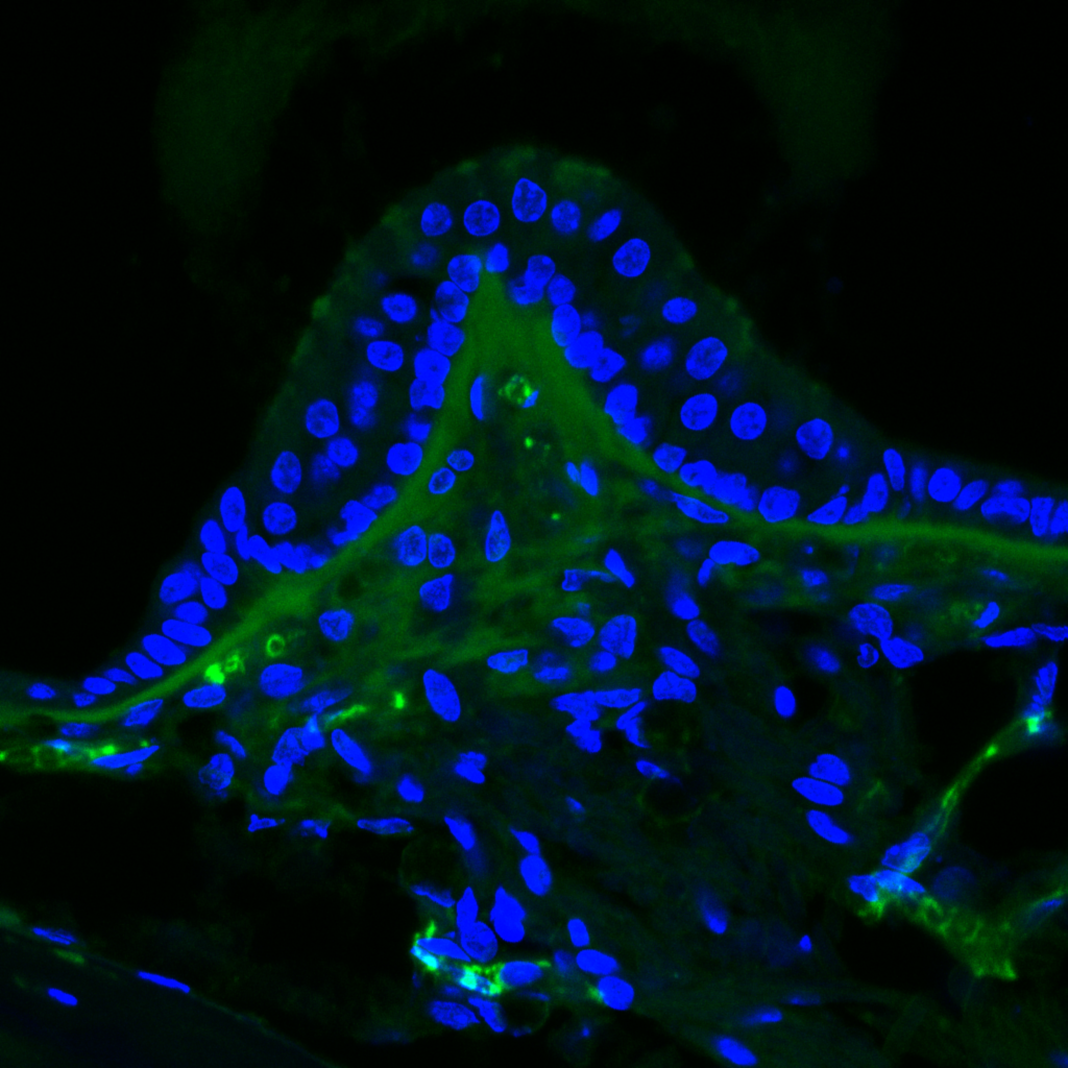

Supplement: Supplementary file 7 — Supporting File 7: advs75263‐sup‐0007‐Data5.zip. [file ADVS-13-e12538-s004.zip › Raw data of microscope images/Figure S2A-KO (3) Merge.tif]

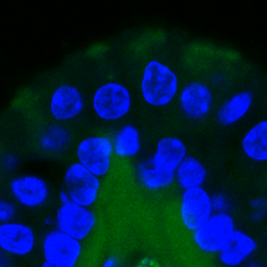

Supplement: Supplementary file 7 — Supporting File 7: advs75263‐sup‐0007‐Data5.zip. [file ADVS-13-e12538-s004.zip › Raw data of microscope images/Figure S2A-KO (4).tif]

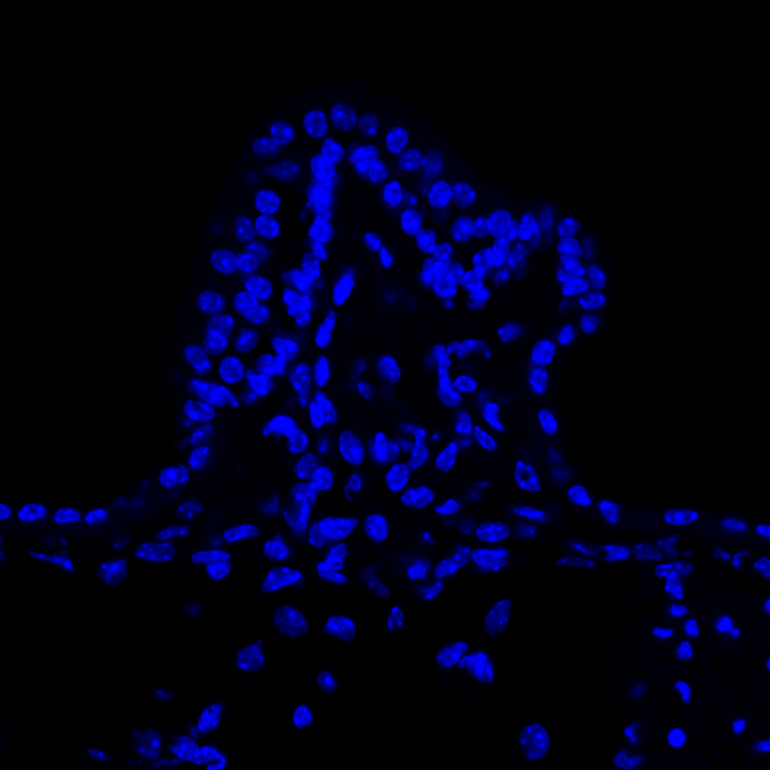

Supplement: Supplementary file 7 — Supporting File 7: advs75263‐sup‐0007‐Data5.zip. [file ADVS-13-e12538-s004.zip › Raw data of microscope images/Figure S2A-wt (1) DAPI.tif]

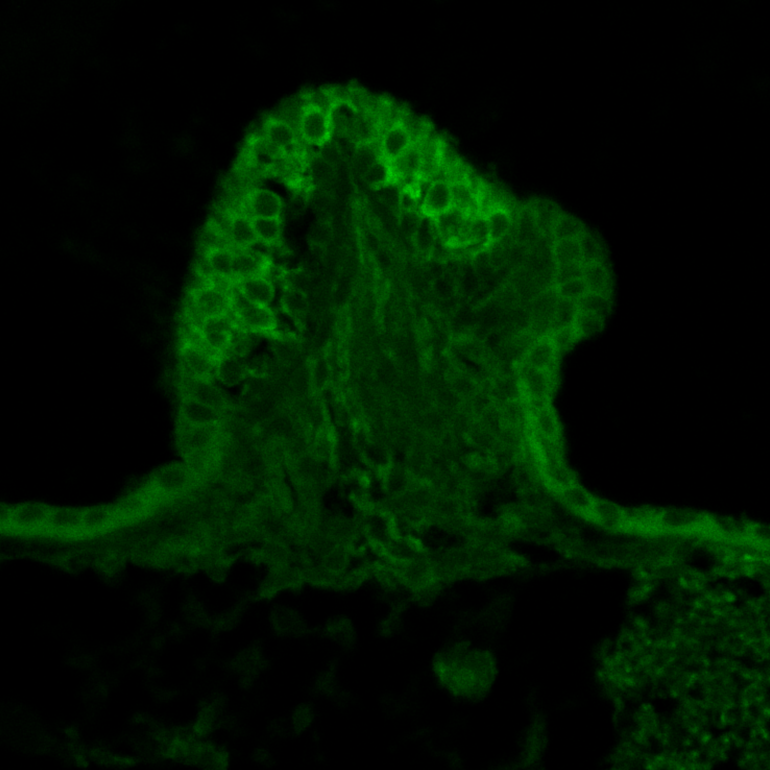

Supplement: Supplementary file 7 — Supporting File 7: advs75263‐sup‐0007‐Data5.zip. [file ADVS-13-e12538-s004.zip › Raw data of microscope images/Figure S2A-wt (2) GATA4.tif]

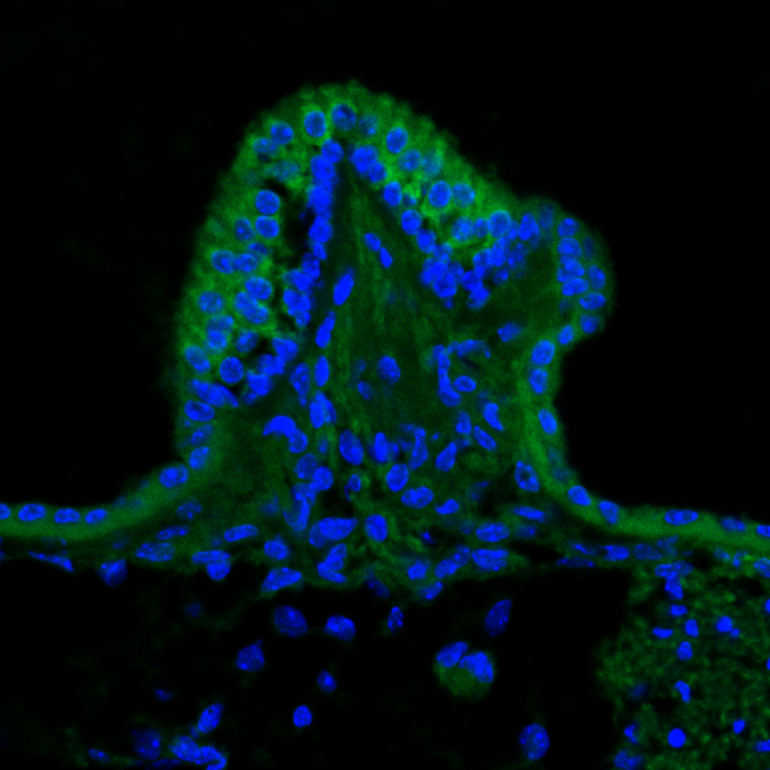

Supplement: Supplementary file 7 — Supporting File 7: advs75263‐sup‐0007‐Data5.zip. [file ADVS-13-e12538-s004.zip › Raw data of microscope images/Figure S2A-wt (3) Merge.tif]

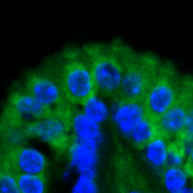

Supplement: Supplementary file 7 — Supporting File 7: advs75263‐sup‐0007‐Data5.zip. [file ADVS-13-e12538-s004.zip › Raw data of microscope images/Figure S2A-wt (4).tif]

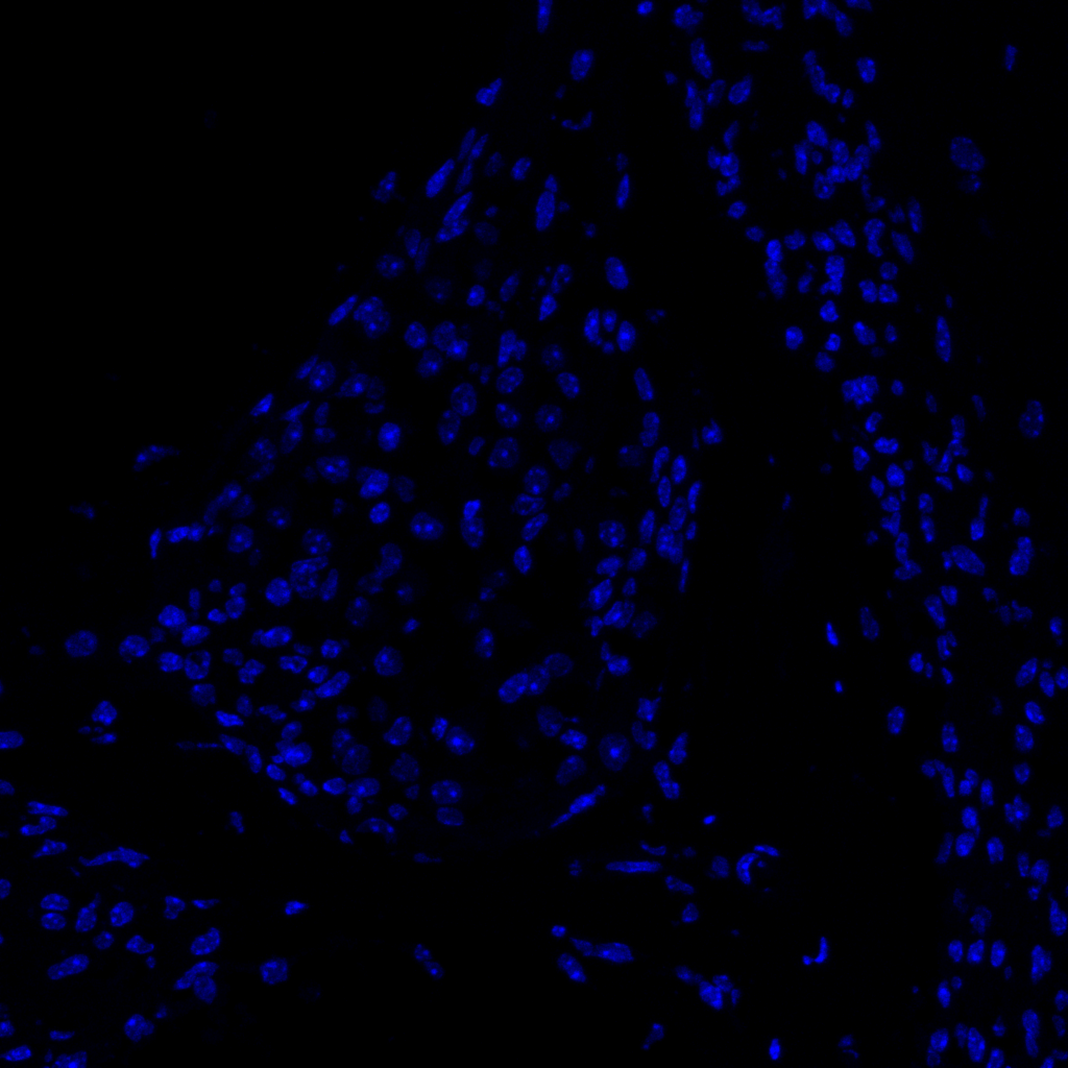

Supplement: Supplementary file 7 — Supporting File 7: advs75263‐sup‐0007‐Data5.zip. [file ADVS-13-e12538-s004.zip › Raw data of microscope images/Figure S2B-KO (1) DAPI.tif]

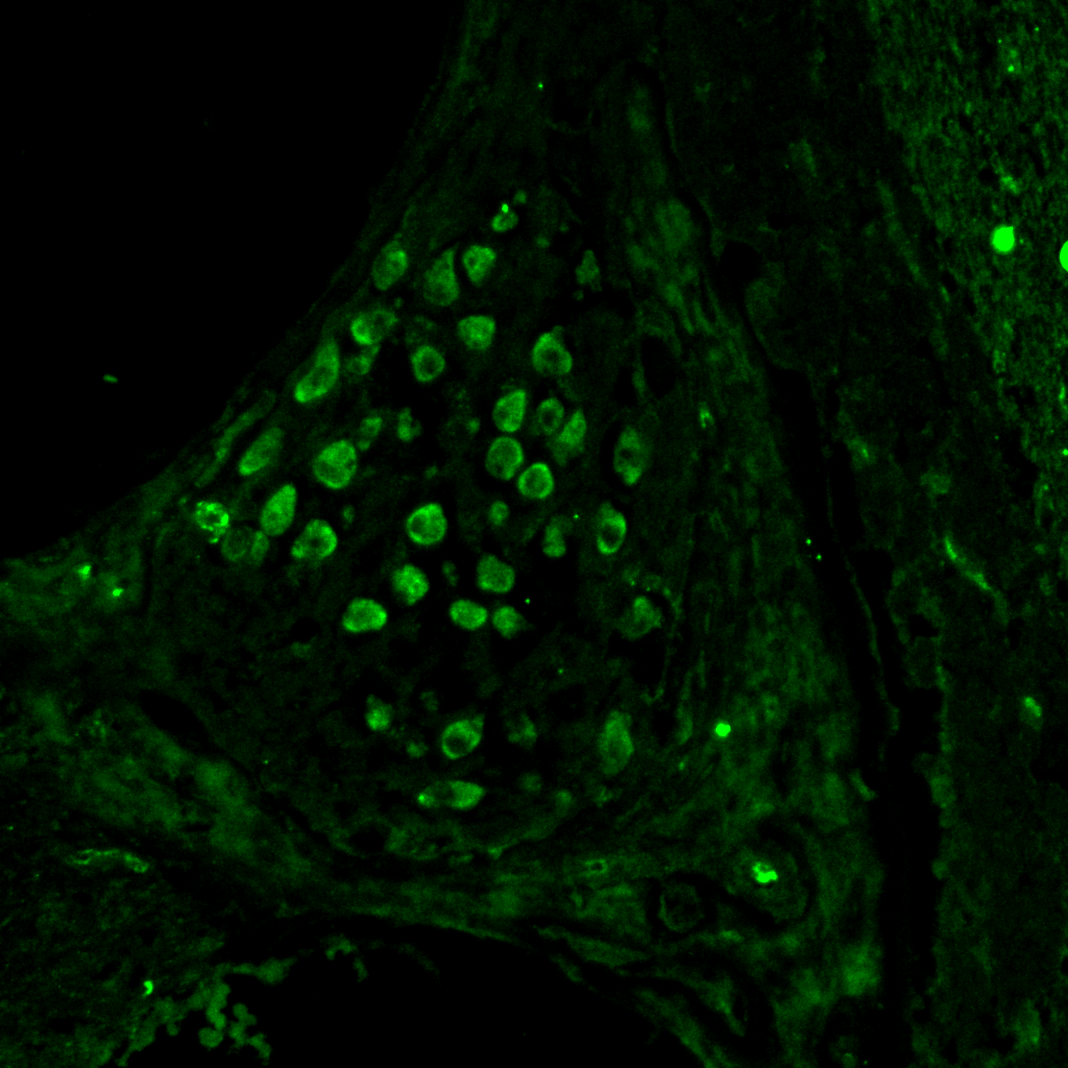

Supplement: Supplementary file 7 — Supporting File 7: advs75263‐sup‐0007‐Data5.zip. [file ADVS-13-e12538-s004.zip › Raw data of microscope images/Figure S2B-KO (2) GATA4.tif]

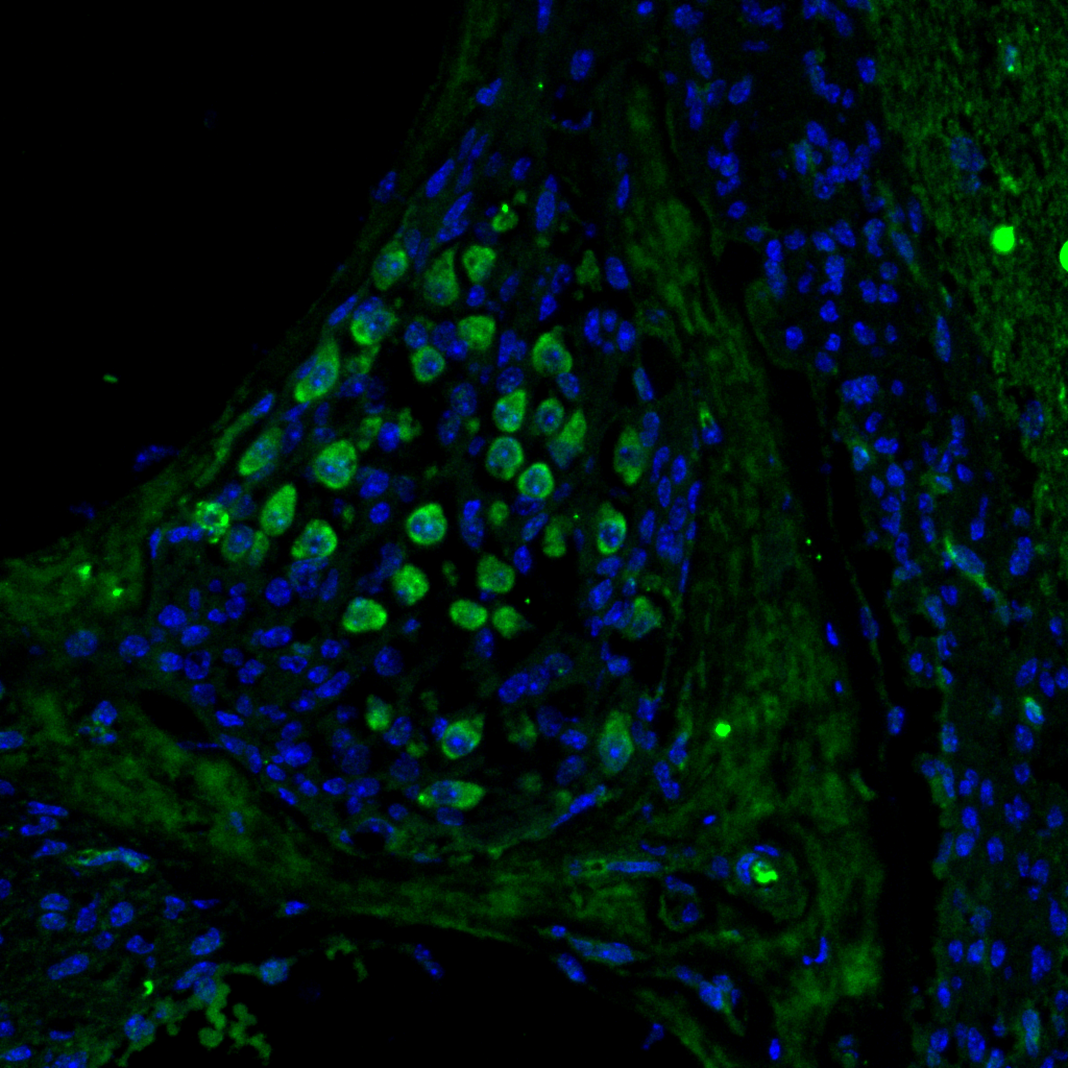

Supplement: Supplementary file 7 — Supporting File 7: advs75263‐sup‐0007‐Data5.zip. [file ADVS-13-e12538-s004.zip › Raw data of microscope images/Figure S2B-KO (3) Merge.tif]

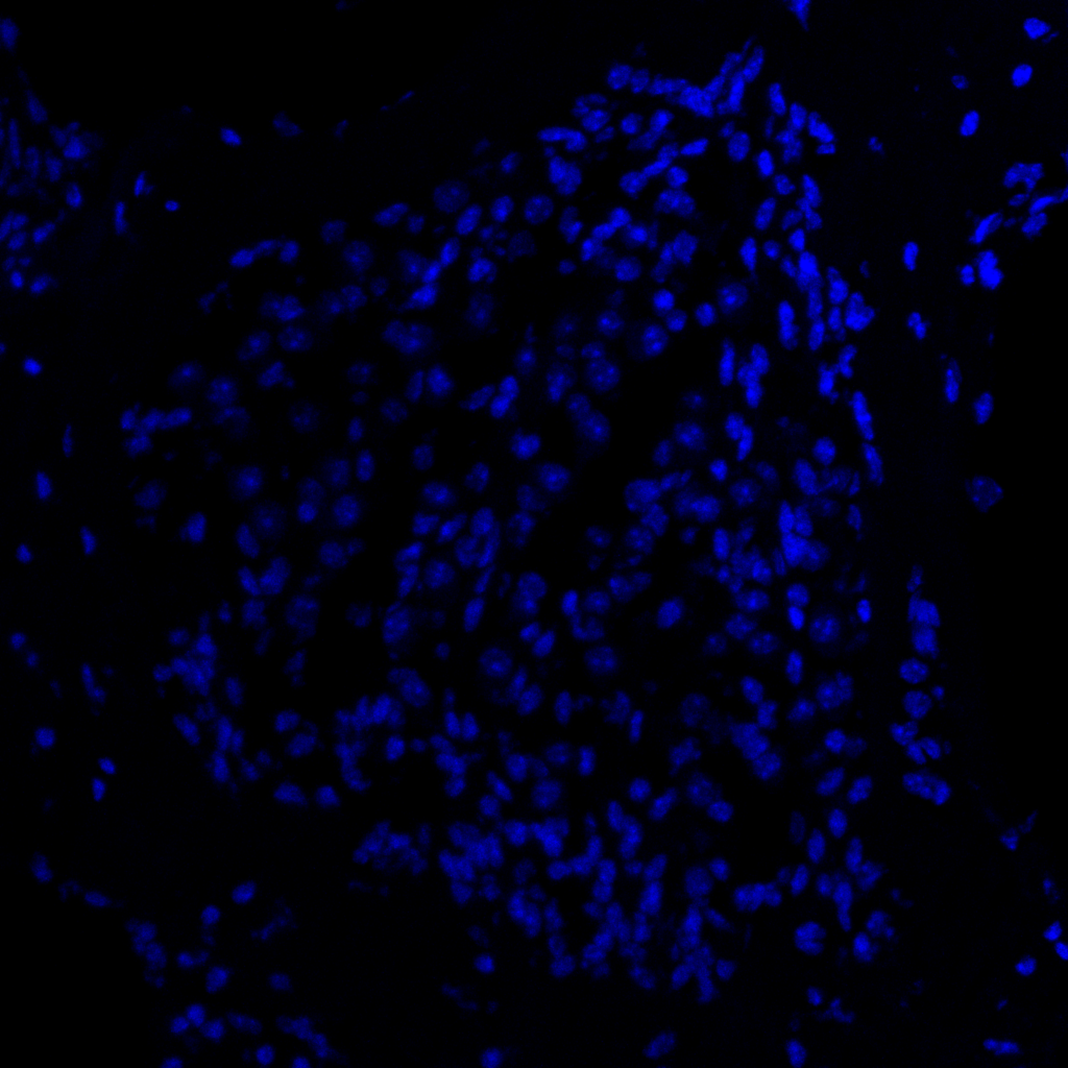

Supplement: Supplementary file 7 — Supporting File 7: advs75263‐sup‐0007‐Data5.zip. [file ADVS-13-e12538-s004.zip › Raw data of microscope images/Figure S2B-wt (1) DAPI.tif]

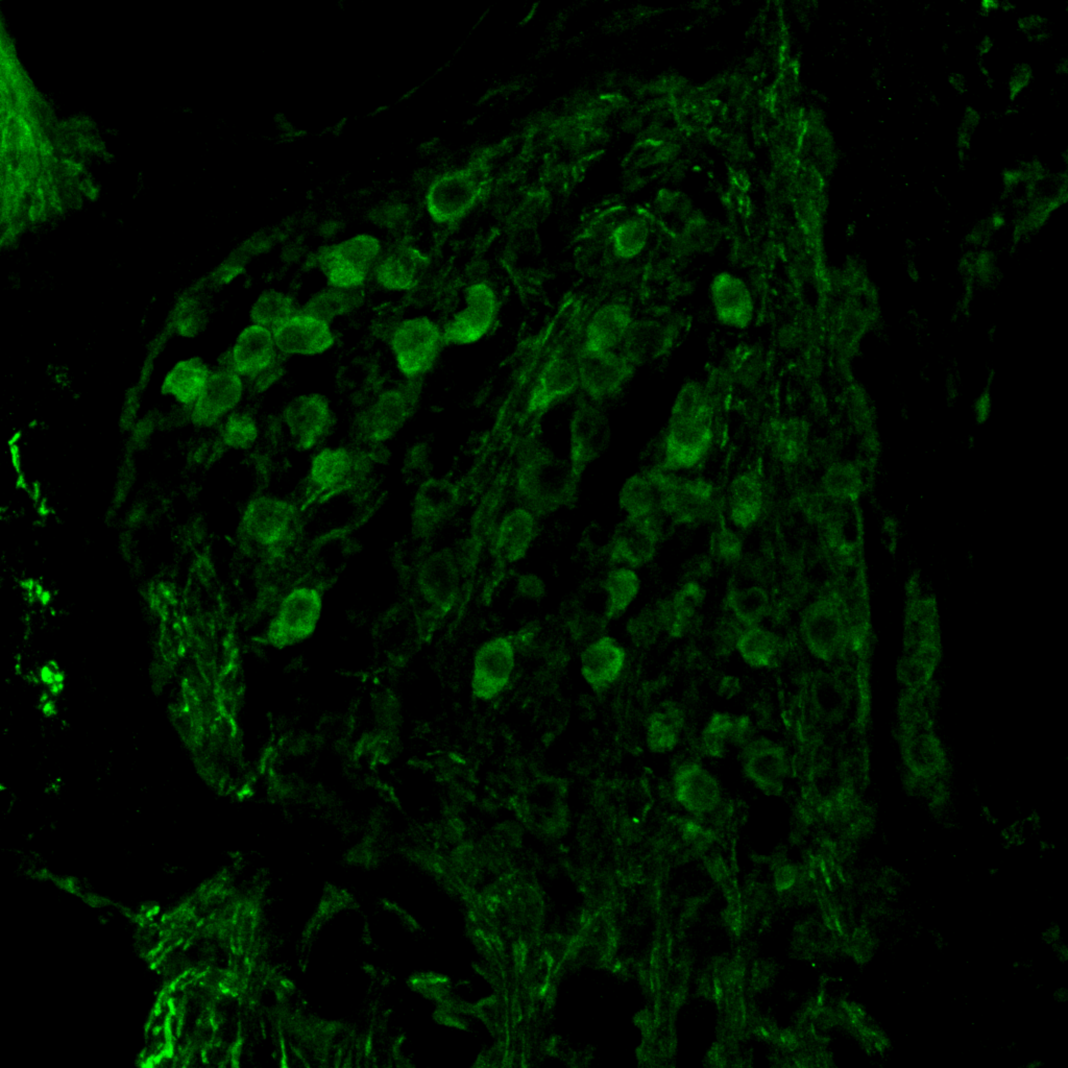

Supplement: Supplementary file 7 — Supporting File 7: advs75263‐sup‐0007‐Data5.zip. [file ADVS-13-e12538-s004.zip › Raw data of microscope images/Figure S2B-wt (2) GATA4.tif]

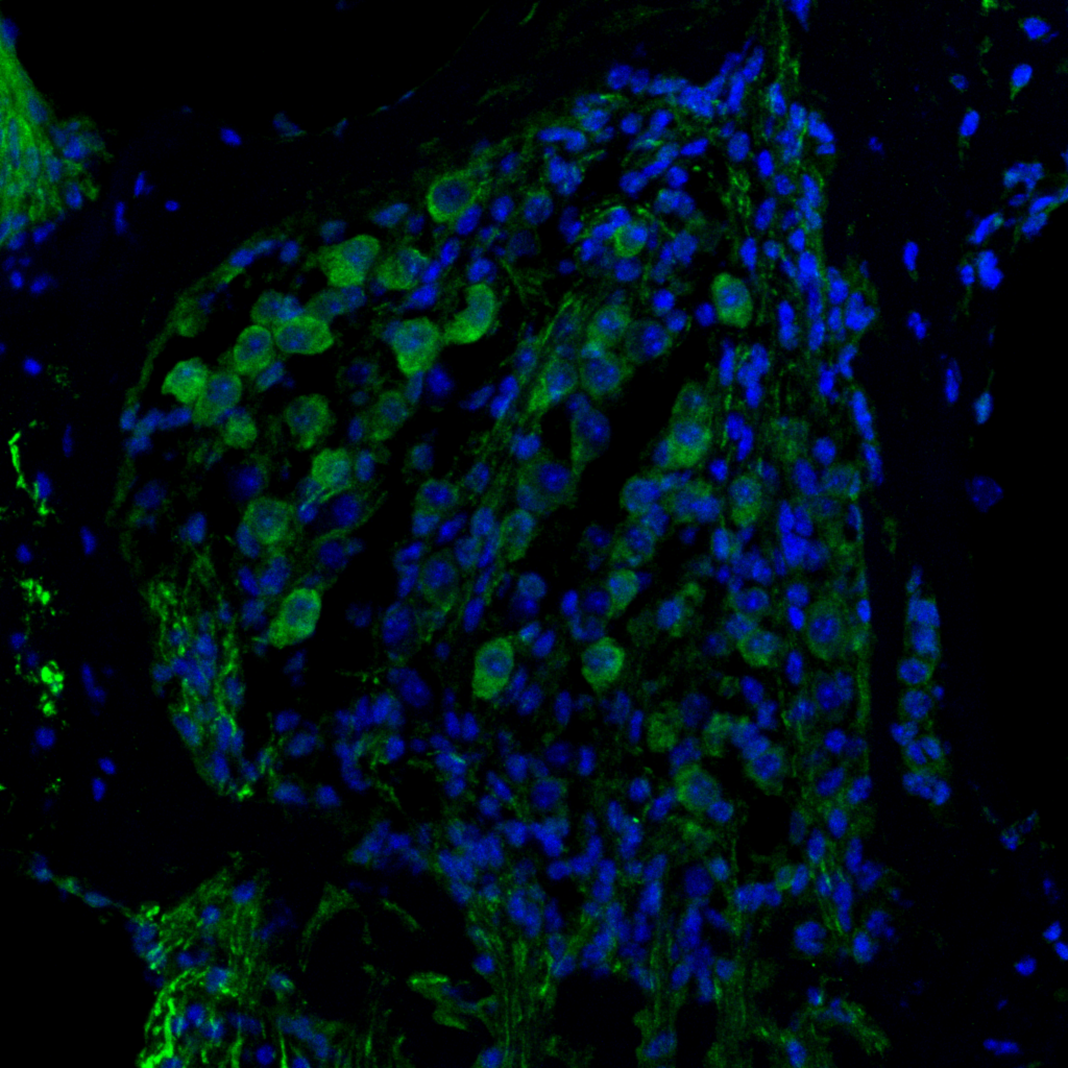

Supplement: Supplementary file 7 — Supporting File 7: advs75263‐sup‐0007‐Data5.zip. [file ADVS-13-e12538-s004.zip › Raw data of microscope images/Figure S2B-wt (3) Merge.tif]

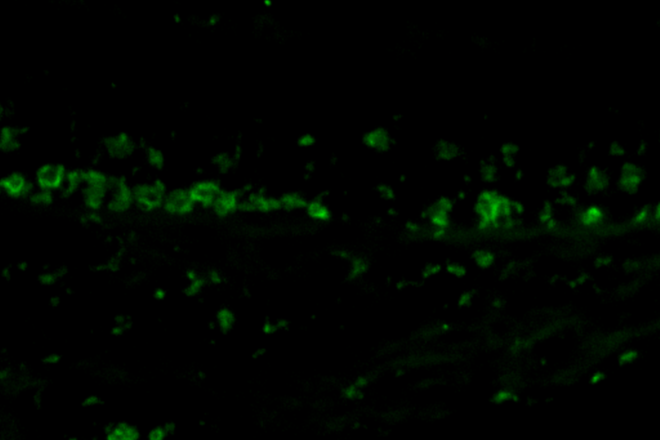

Supplement: Supplementary file 7 — Supporting File 7: advs75263‐sup‐0007‐Data5.zip. [file ADVS-13-e12538-s004.zip › Raw data of microscope images/Figure S2D-KO LPS (1) P16.tif]

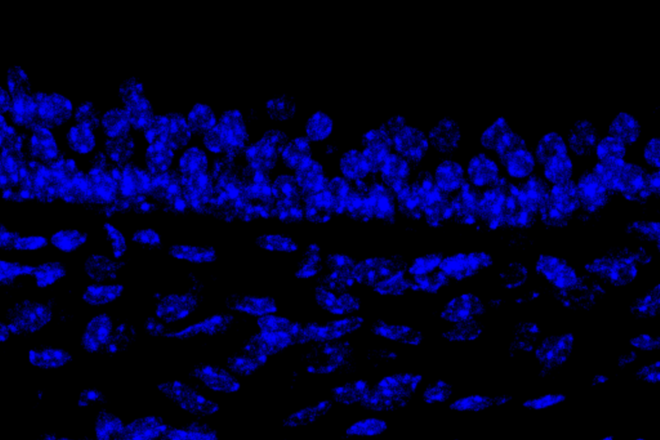

Supplement: Supplementary file 7 — Supporting File 7: advs75263‐sup‐0007‐Data5.zip. [file ADVS-13-e12538-s004.zip › Raw data of microscope images/Figure S2D-KO LPS (2) DAPI.tif]

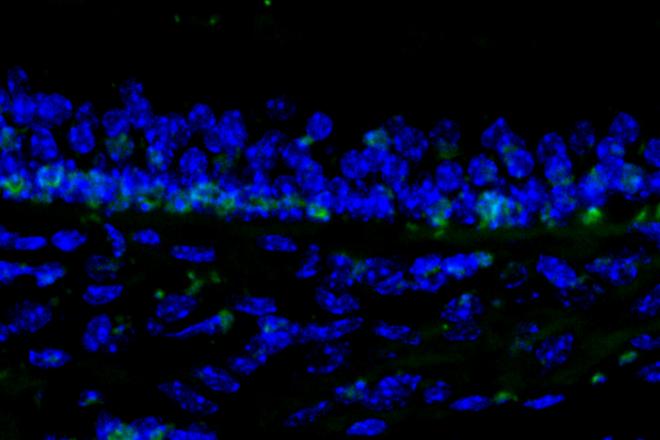

Supplement: Supplementary file 7 — Supporting File 7: advs75263‐sup‐0007‐Data5.zip. [file ADVS-13-e12538-s004.zip › Raw data of microscope images/Figure S2D-KO LPS (3) Merge.tif]

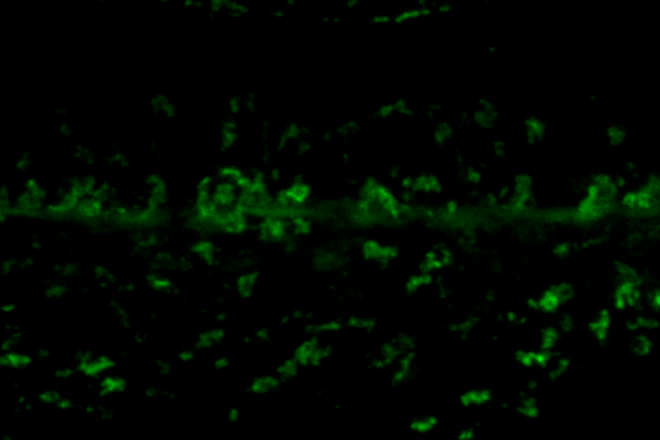

Supplement: Supplementary file 7 — Supporting File 7: advs75263‐sup‐0007‐Data5.zip. [file ADVS-13-e12538-s004.zip › Raw data of microscope images/Figure S2D-KO NS (1) P16.tif]

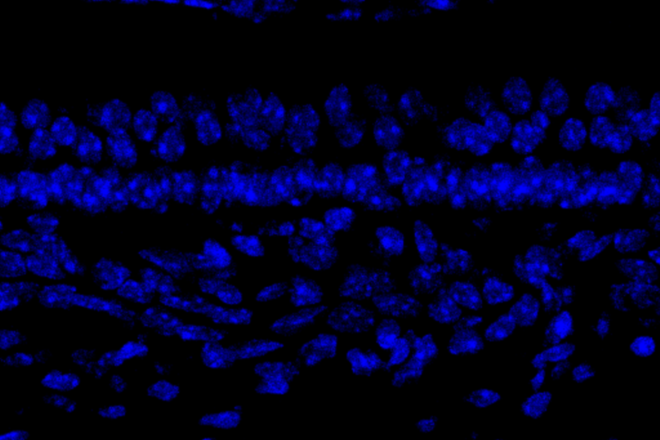

Supplement: Supplementary file 7 — Supporting File 7: advs75263‐sup‐0007‐Data5.zip. [file ADVS-13-e12538-s004.zip › Raw data of microscope images/Figure S2D-KO NS (2) DAPI.tif]

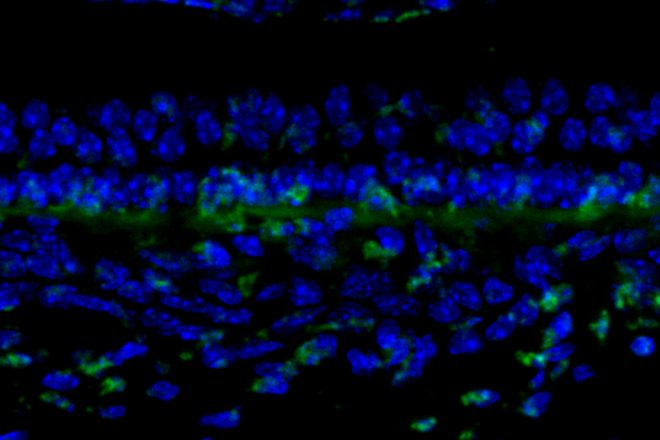

Supplement: Supplementary file 7 — Supporting File 7: advs75263‐sup‐0007‐Data5.zip. [file ADVS-13-e12538-s004.zip › Raw data of microscope images/Figure S2D-KO NS (3) Merge.tif]

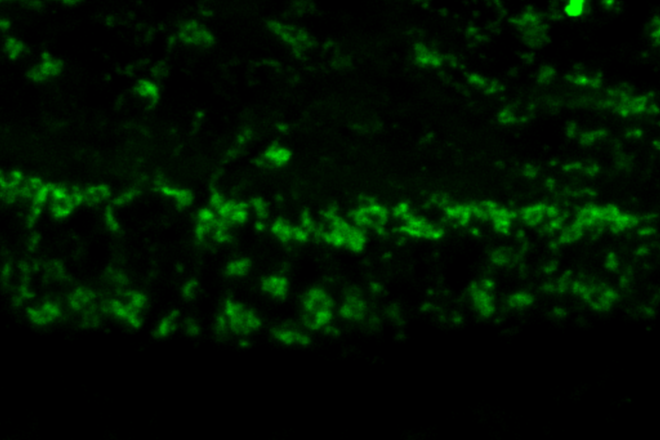

Supplement: Supplementary file 7 — Supporting File 7: advs75263‐sup‐0007‐Data5.zip. [file ADVS-13-e12538-s004.zip › Raw data of microscope images/Figure S2D-wt LPS (1) P16.tif]

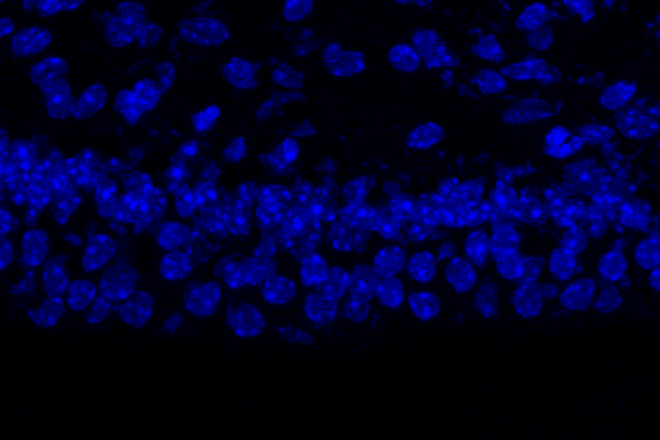

Supplement: Supplementary file 7 — Supporting File 7: advs75263‐sup‐0007‐Data5.zip. [file ADVS-13-e12538-s004.zip › Raw data of microscope images/Figure S2D-wt LPS (2) DAPI.tif]

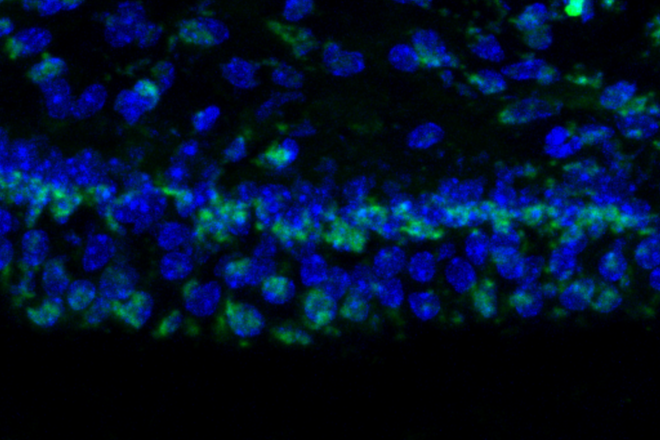

Supplement: Supplementary file 7 — Supporting File 7: advs75263‐sup‐0007‐Data5.zip. [file ADVS-13-e12538-s004.zip › Raw data of microscope images/Figure S2D-wt LPS (3) Merge.tif]

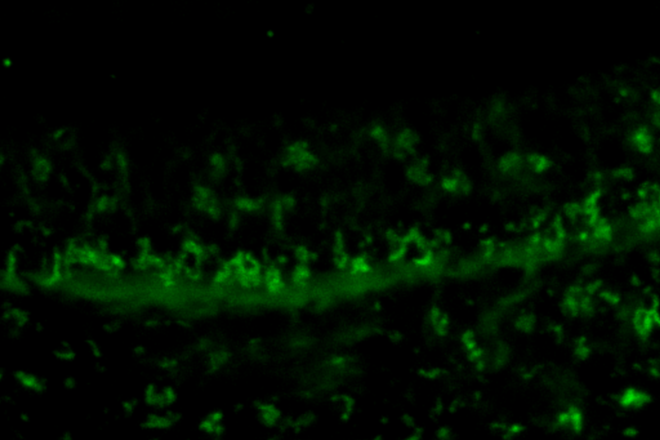

Supplement: Supplementary file 7 — Supporting File 7: advs75263‐sup‐0007‐Data5.zip. [file ADVS-13-e12538-s004.zip › Raw data of microscope images/Figure S2D-wt NS (1) P16.tif]

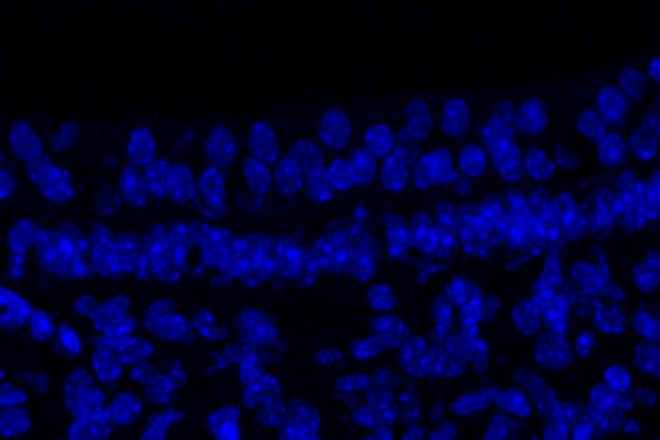

Supplement: Supplementary file 7 — Supporting File 7: advs75263‐sup‐0007‐Data5.zip. [file ADVS-13-e12538-s004.zip › Raw data of microscope images/Figure S2D-wt NS (2) DAPI.tif]

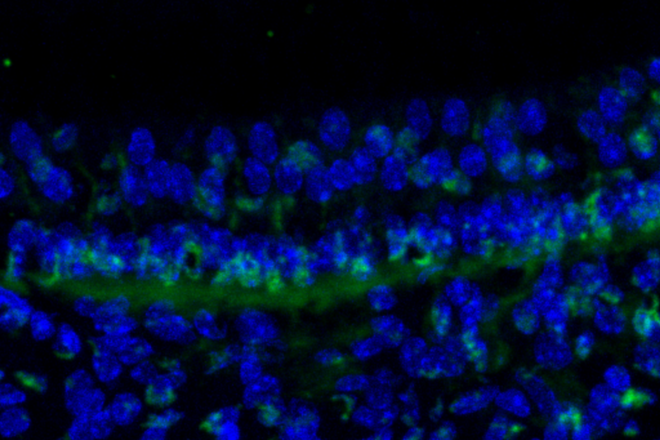

Supplement: Supplementary file 7 — Supporting File 7: advs75263‐sup‐0007‐Data5.zip. [file ADVS-13-e12538-s004.zip › Raw data of microscope images/Figure S2D-wt NS (3) Merge.tif]

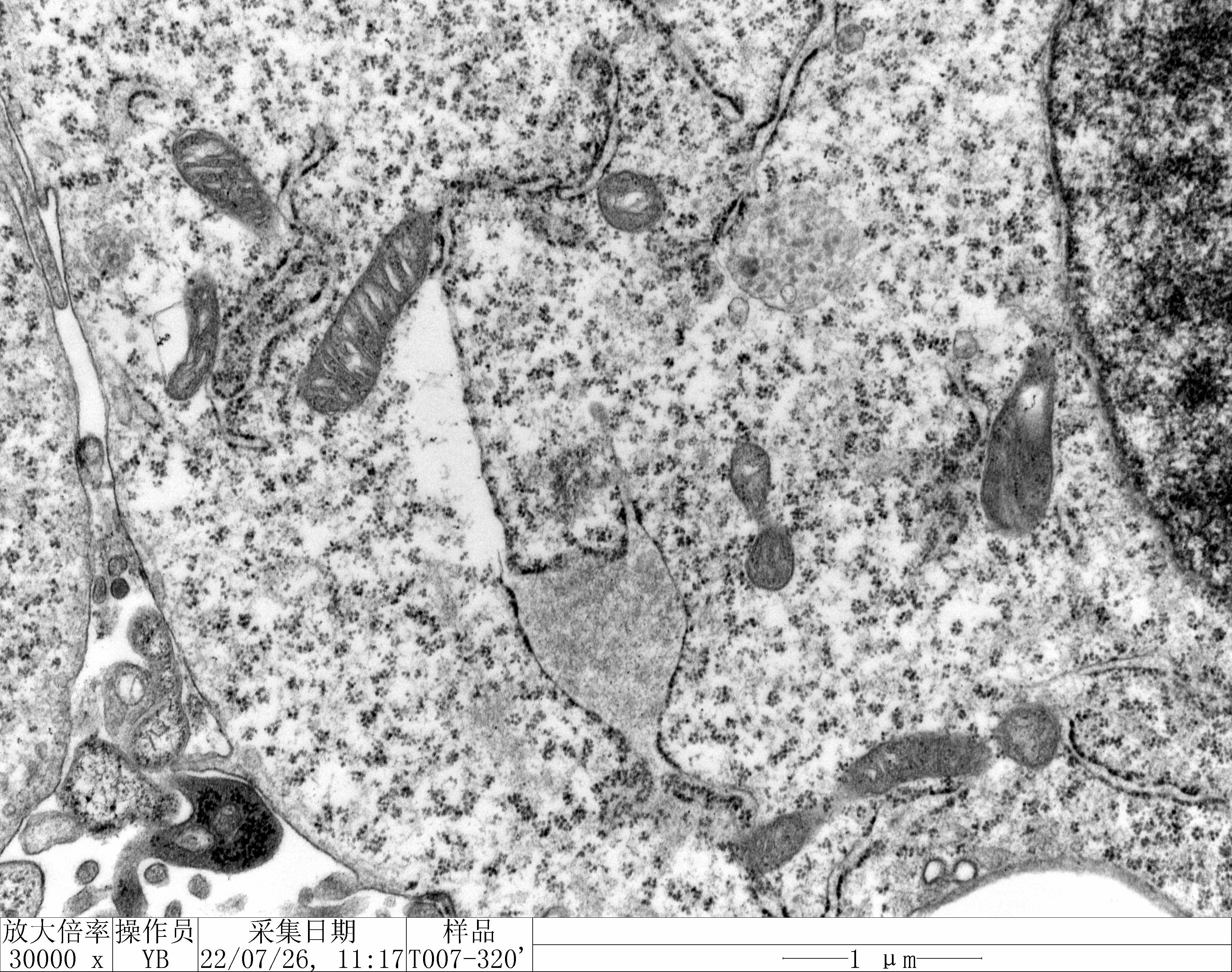

Supplement: Supplementary file 7 — Supporting File 7: advs75263‐sup‐0007‐Data5.zip. [file ADVS-13-e12538-s004.zip › Raw data of microscope images/Figure S3E-Ctrl 2.jpg]

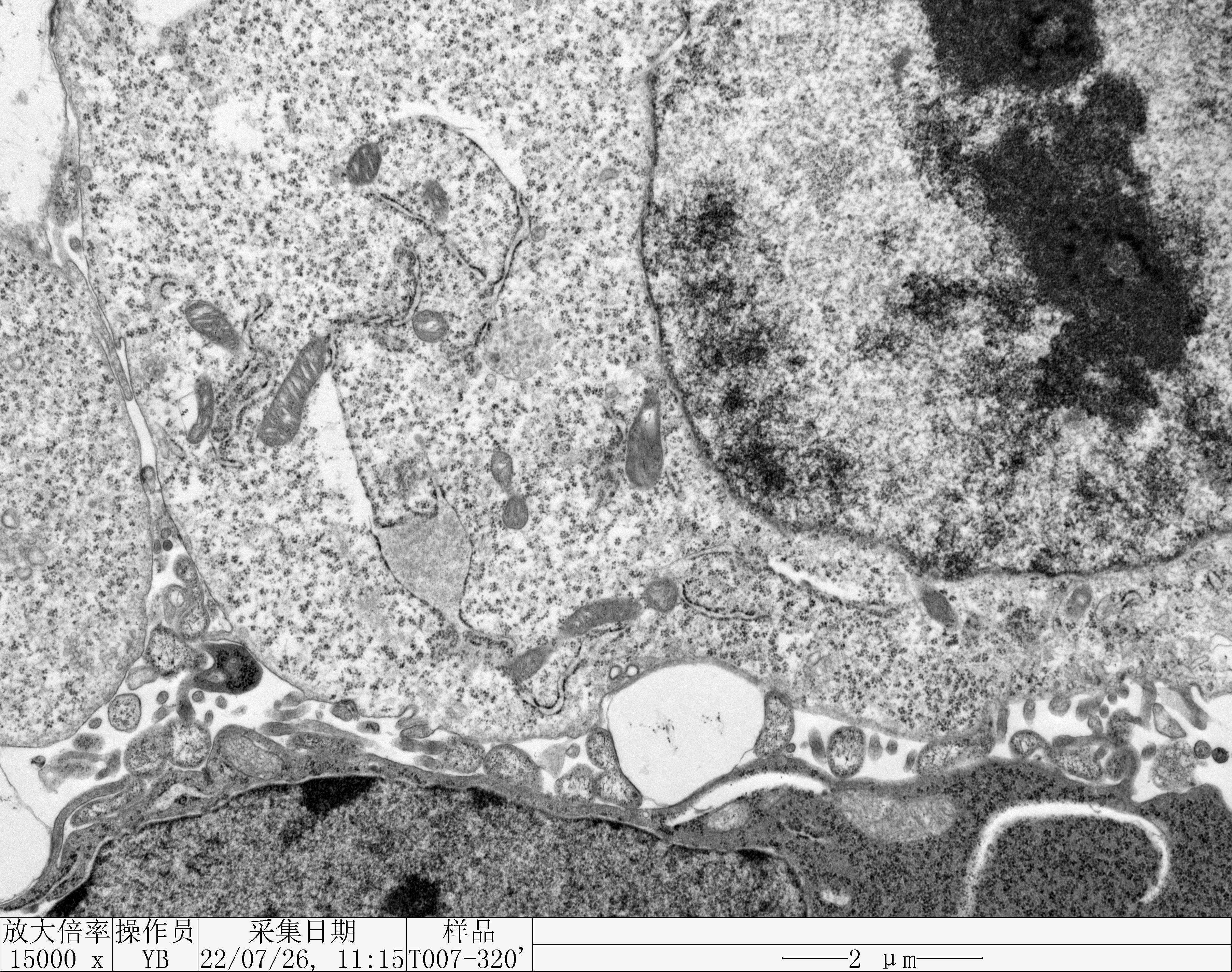

Supplement: Supplementary file 7 — Supporting File 7: advs75263‐sup‐0007‐Data5.zip. [file ADVS-13-e12538-s004.zip › Raw data of microscope images/Figure S3E-Ctrl.jpg]

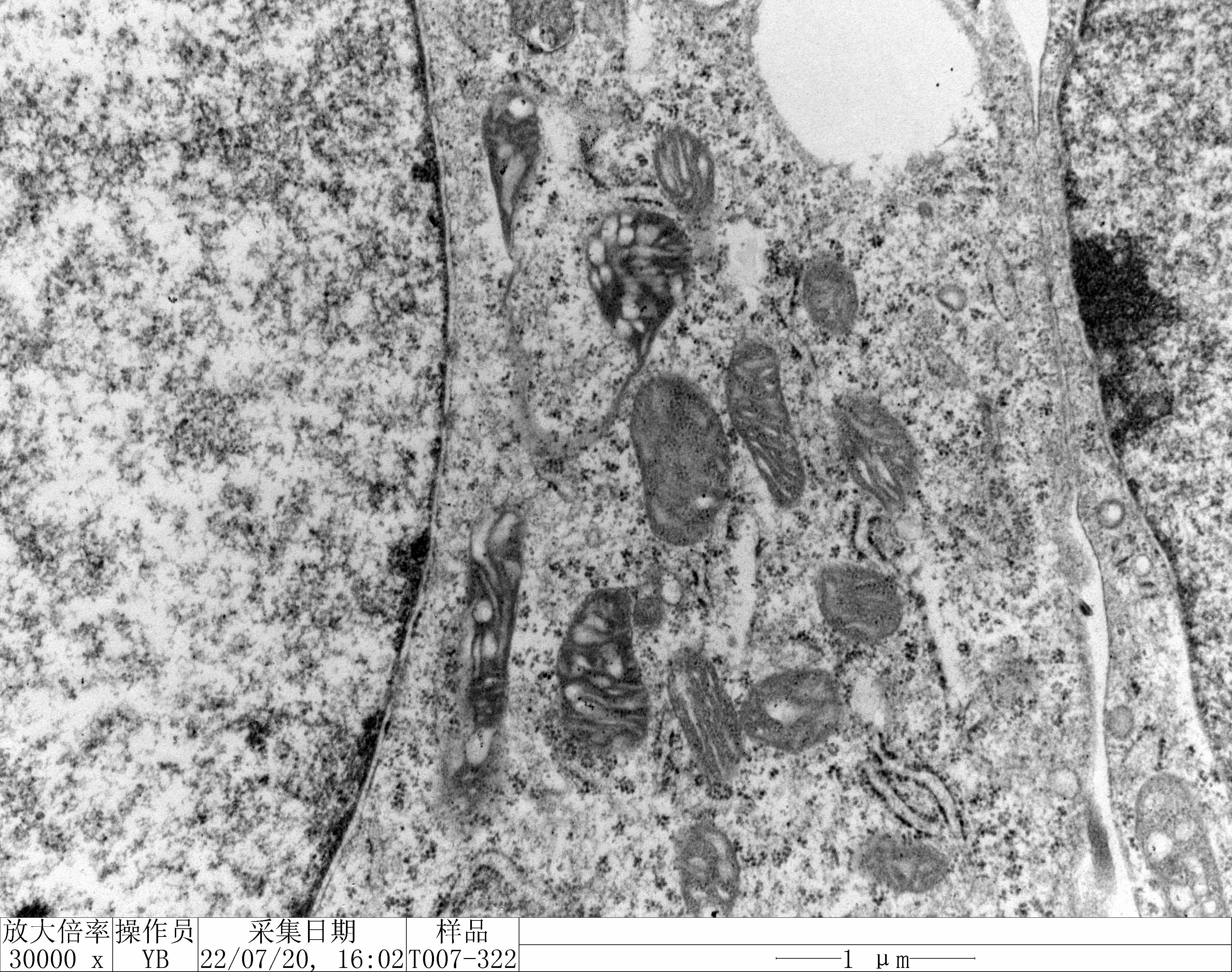

Supplement: Supplementary file 7 — Supporting File 7: advs75263‐sup‐0007‐Data5.zip. [file ADVS-13-e12538-s004.zip › Raw data of microscope images/Figure S3E-Dox 2.jpg]

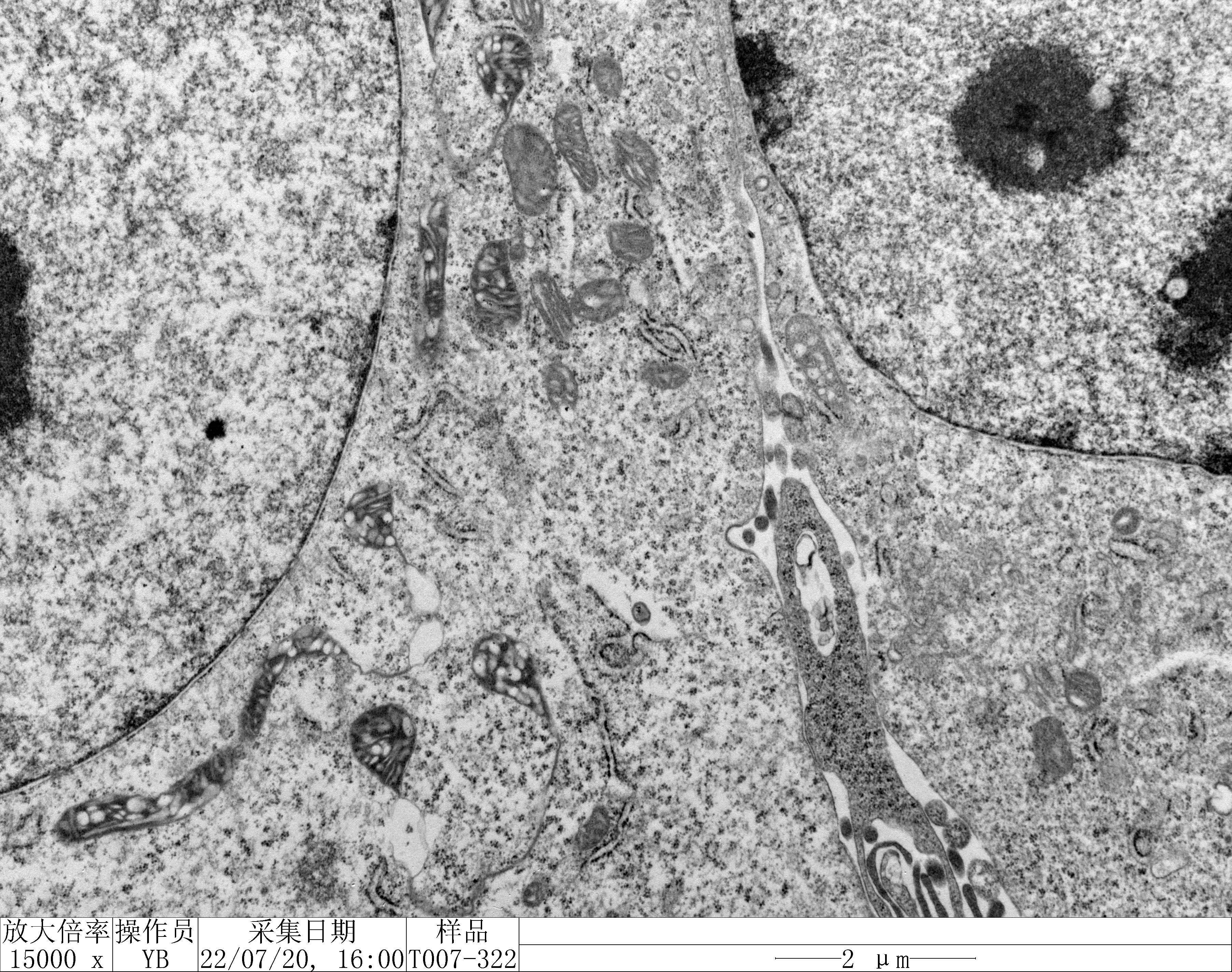

Supplement: Supplementary file 7 — Supporting File 7: advs75263‐sup‐0007‐Data5.zip. [file ADVS-13-e12538-s004.zip › Raw data of microscope images/Figure S3E-Dox.jpg]

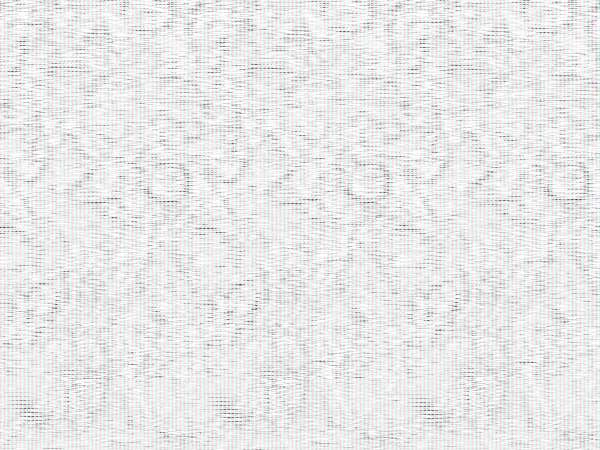

Supplement: Supplementary file 7 — Supporting File 7: advs75263‐sup‐0007‐Data5.zip. [file ADVS-13-e12538-s004.zip › Raw data of microscope images/Figure S4A-Dox+siGATA4.tif]

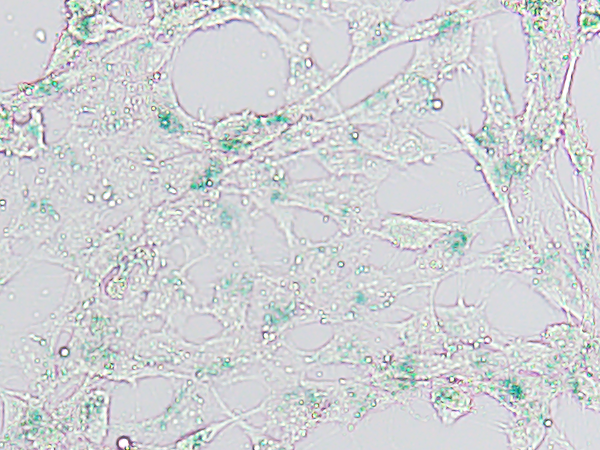

Supplement: Supplementary file 7 — Supporting File 7: advs75263‐sup‐0007‐Data5.zip. [file ADVS-13-e12538-s004.zip › Raw data of microscope images/Figure S4A-Dox.tif]

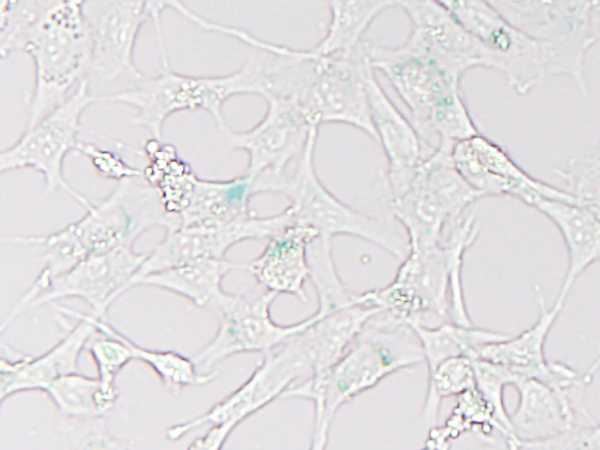

Supplement: Supplementary file 7 — Supporting File 7: advs75263‐sup‐0007‐Data5.zip. [file ADVS-13-e12538-s004.zip › Raw data of microscope images/Figure S4A-siGATA4.tif]

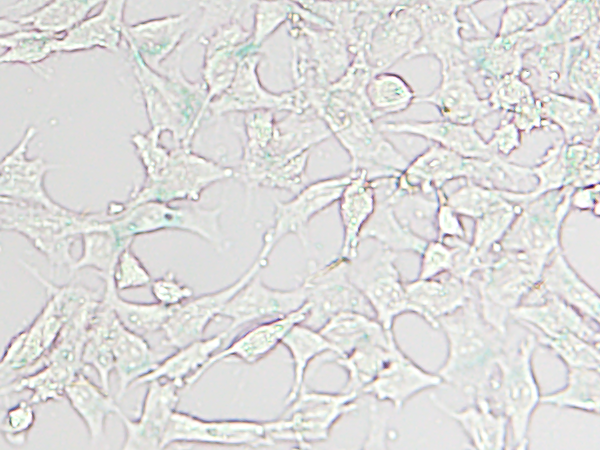

Supplement: Supplementary file 7 — Supporting File 7: advs75263‐sup‐0007‐Data5.zip. [file ADVS-13-e12538-s004.zip › Raw data of microscope images/Figure S4A-siNC.tif]

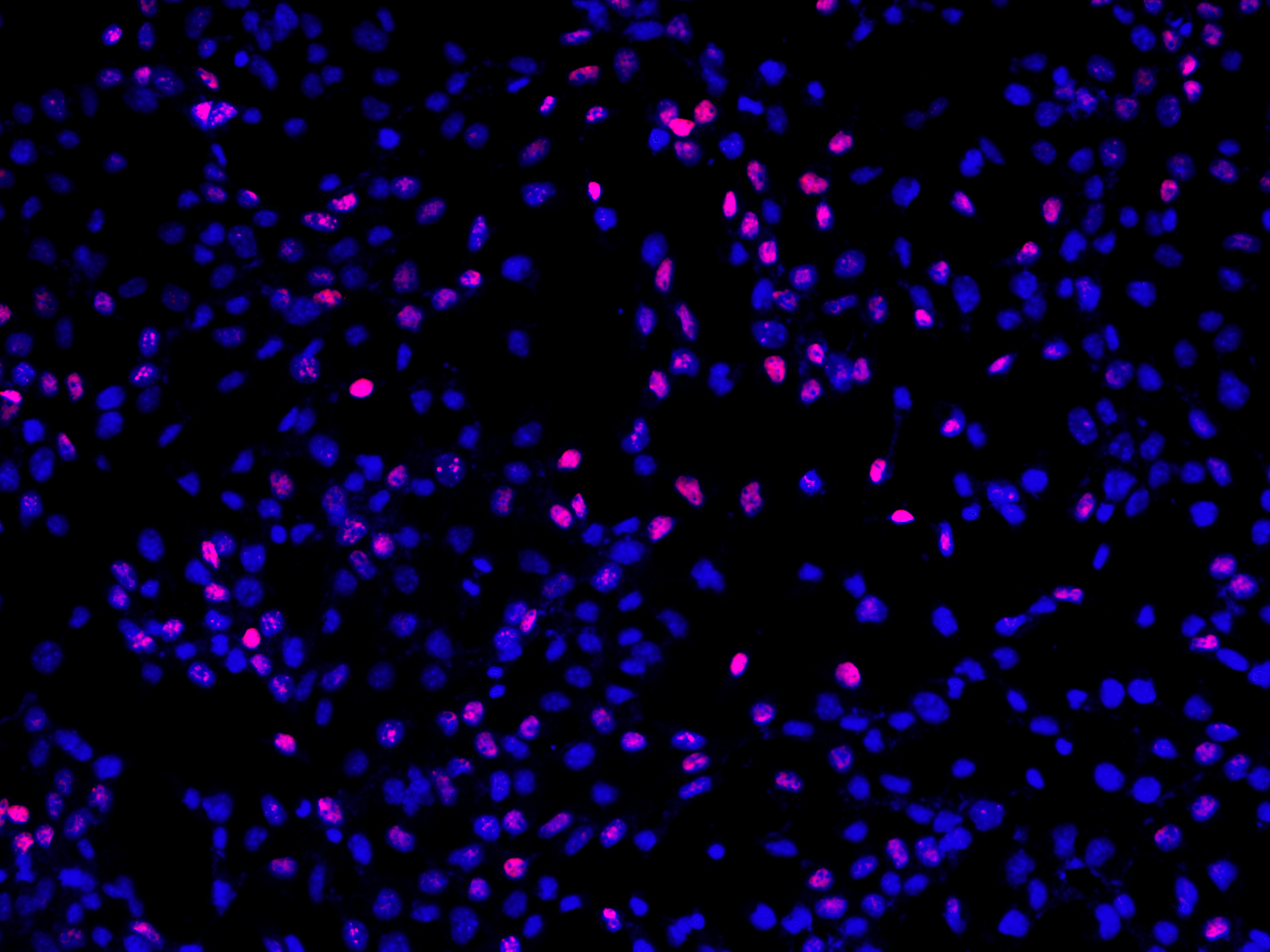

Supplement: Supplementary file 7 — Supporting File 7: advs75263‐sup‐0007‐Data5.zip. [file ADVS-13-e12538-s004.zip › Raw data of microscope images/Figure S4C-Dox+siGATA4.tif]

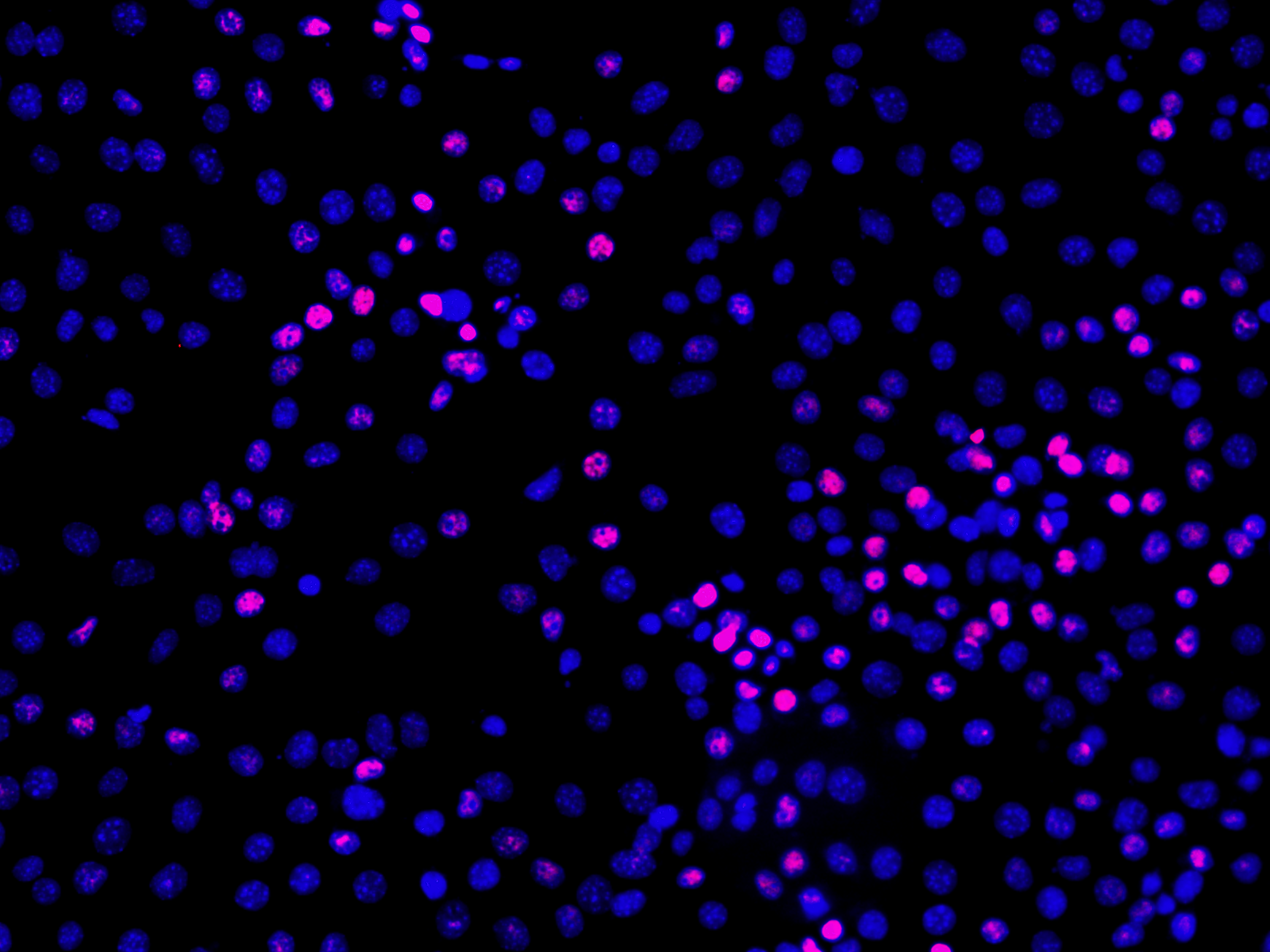

Supplement: Supplementary file 7 — Supporting File 7: advs75263‐sup‐0007‐Data5.zip. [file ADVS-13-e12538-s004.zip › Raw data of microscope images/Figure S4C-Dox.tif]

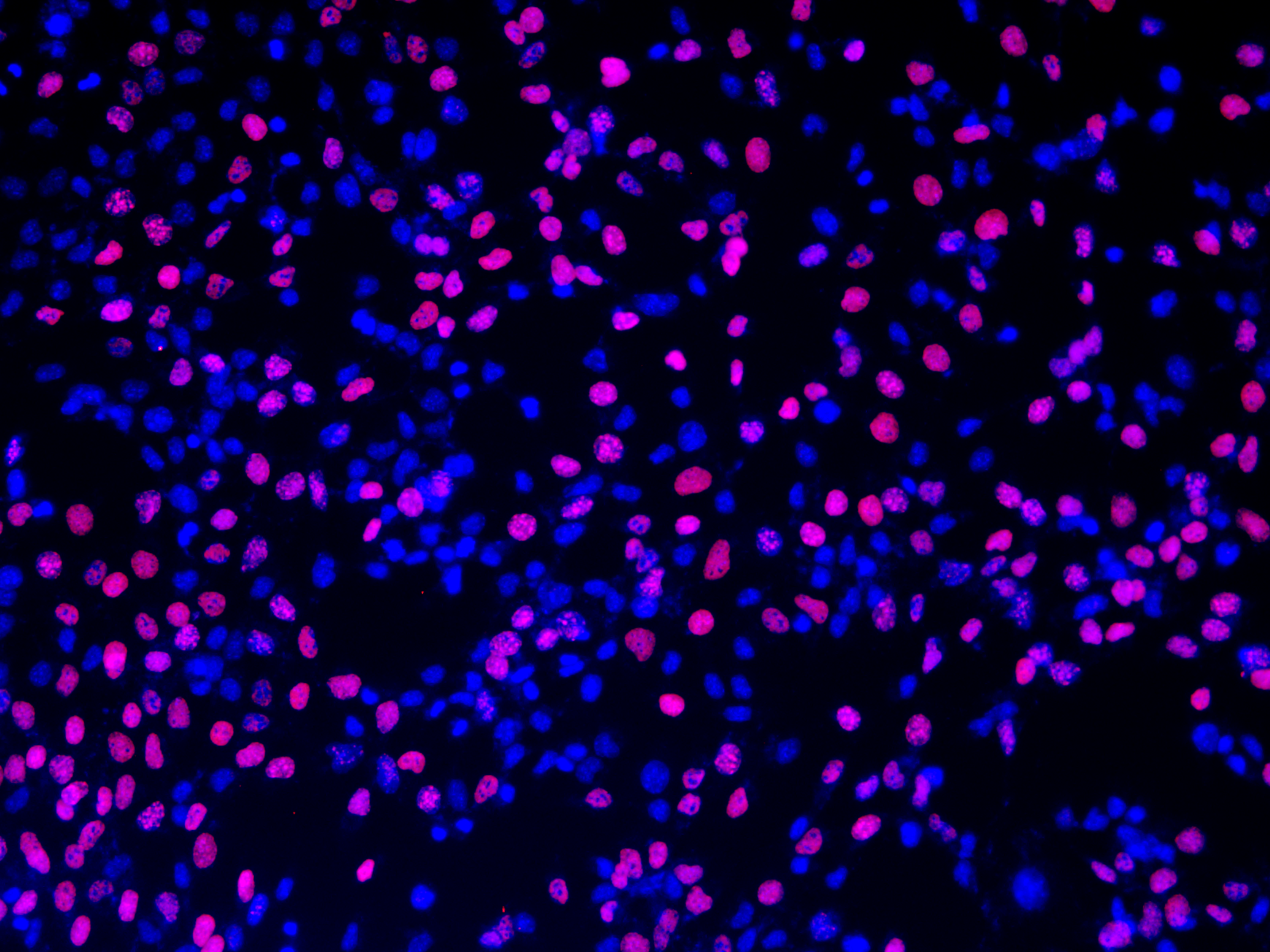

Supplement: Supplementary file 7 — Supporting File 7: advs75263‐sup‐0007‐Data5.zip. [file ADVS-13-e12538-s004.zip › Raw data of microscope images/Figure S4C-siGATA4.tif]

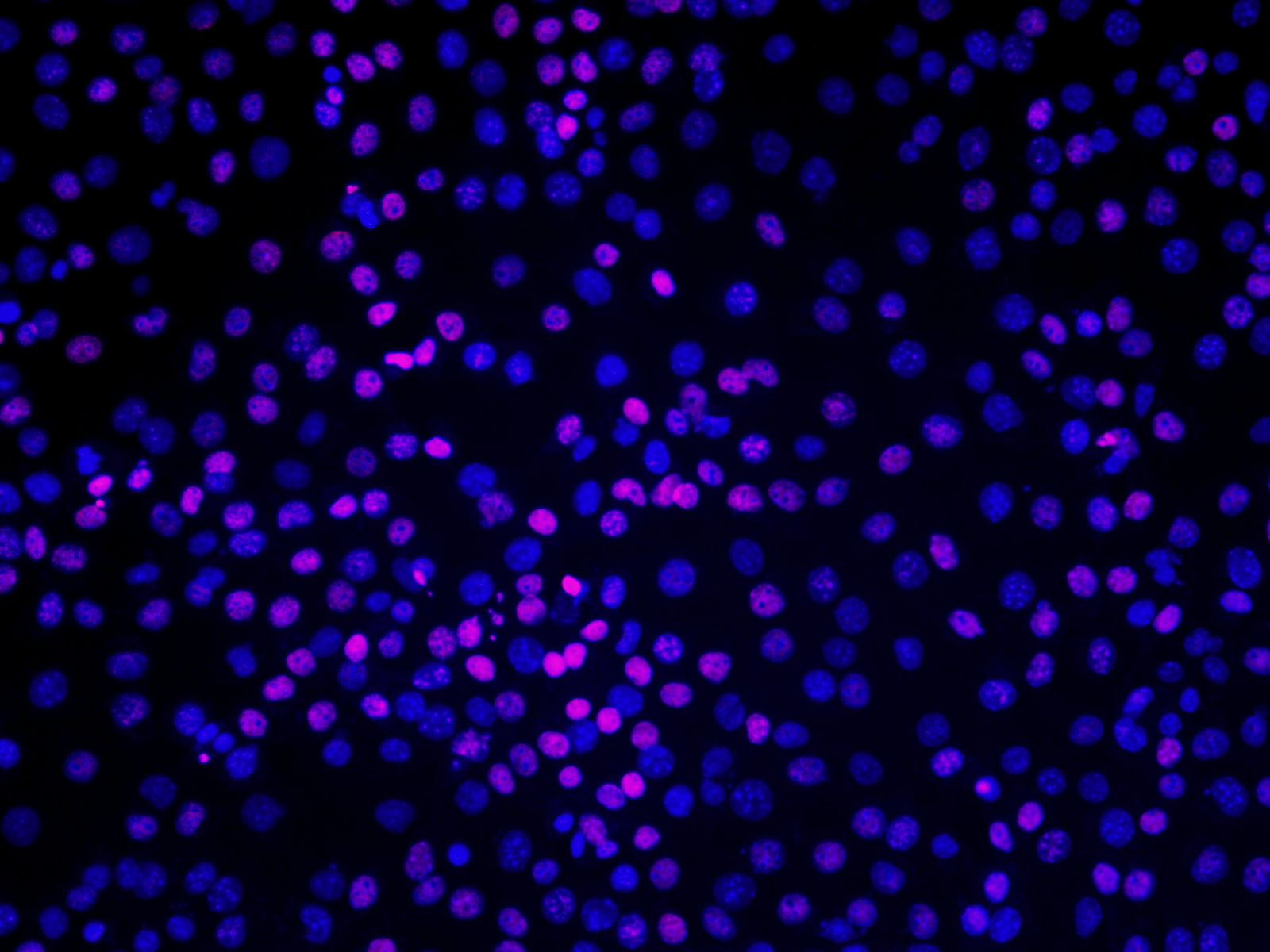

Supplement: Supplementary file 7 — Supporting File 7: advs75263‐sup‐0007‐Data5.zip. [file ADVS-13-e12538-s004.zip › Raw data of microscope images/Figure S4C-siNC.tif]

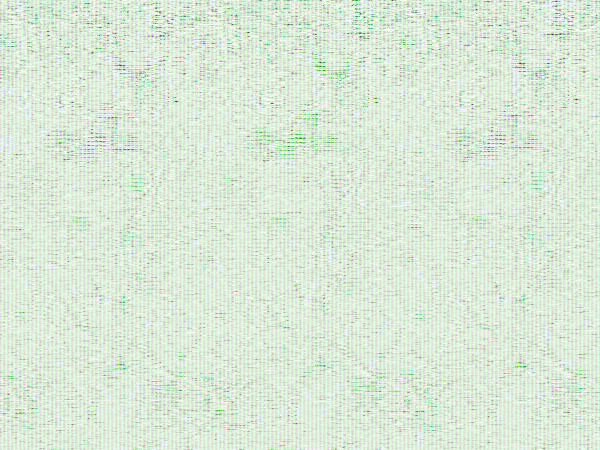

Supplement: Supplementary file 7 — Supporting File 7: advs75263‐sup‐0007‐Data5.zip. [file ADVS-13-e12538-s004.zip › Raw data of microscope images/Figure S5A-GATA4.tif]

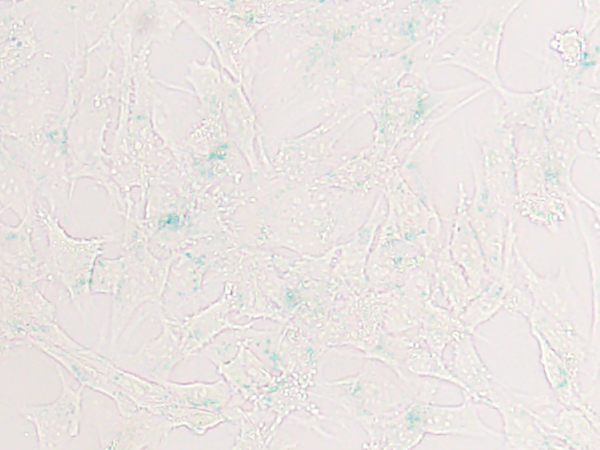

Supplement: Supplementary file 7 — Supporting File 7: advs75263‐sup‐0007‐Data5.zip. [file ADVS-13-e12538-s004.zip › Raw data of microscope images/Figure S5A-Vector.tif]

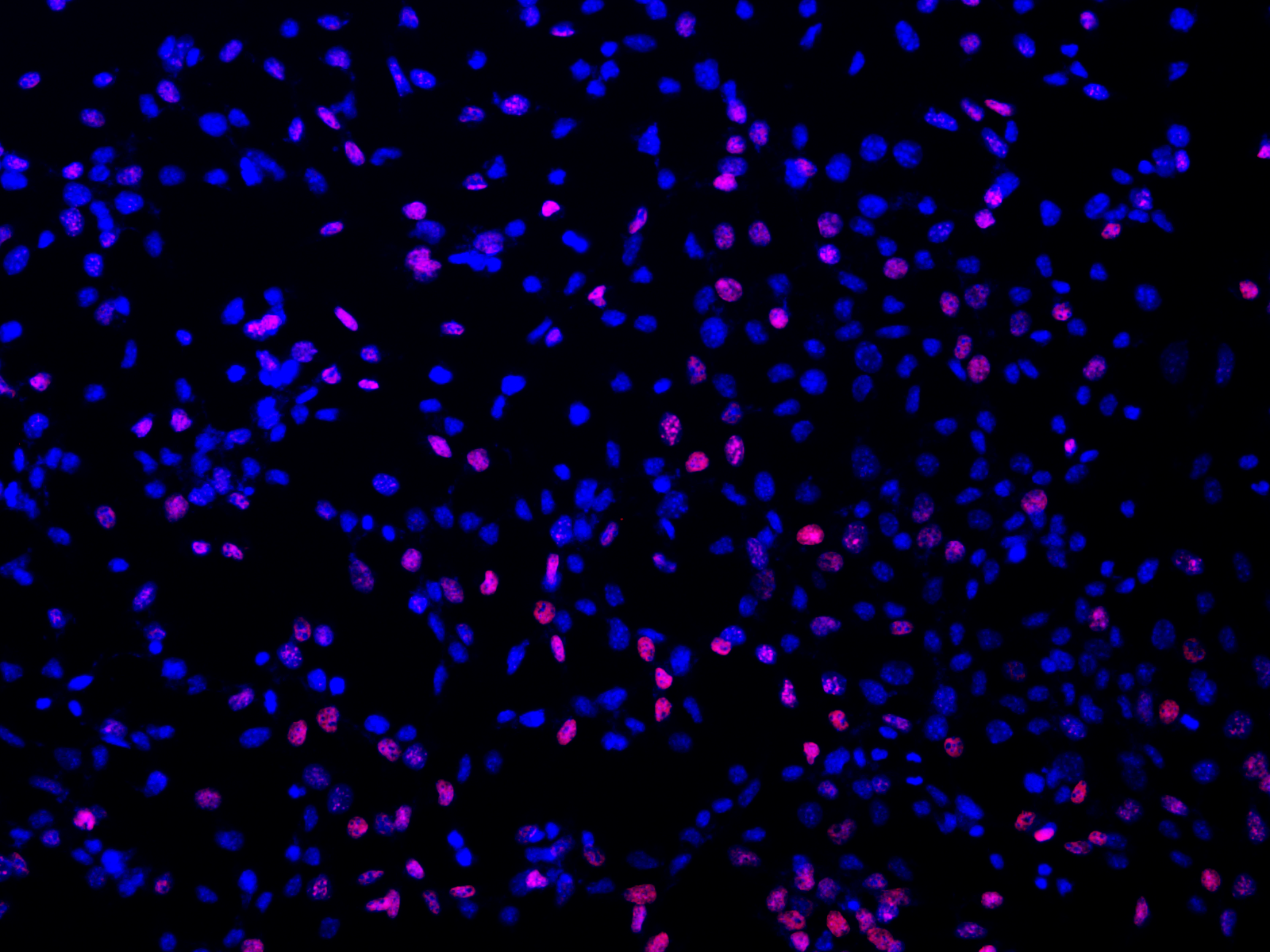

Supplement: Supplementary file 7 — Supporting File 7: advs75263‐sup‐0007‐Data5.zip. [file ADVS-13-e12538-s004.zip › Raw data of microscope images/Figure S5D-GATA4.tif]

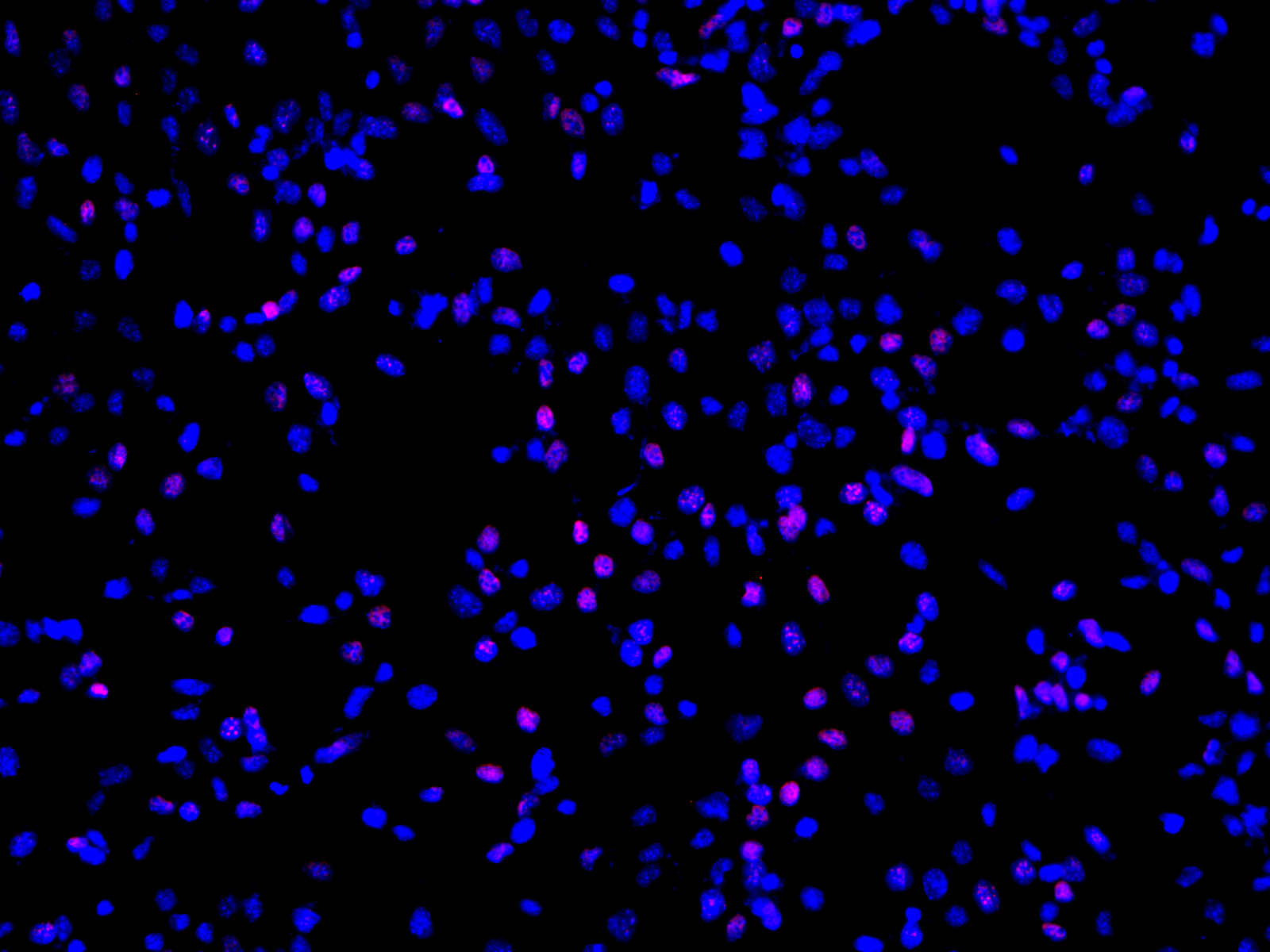

Supplement: Supplementary file 7 — Supporting File 7: advs75263‐sup‐0007‐Data5.zip. [file ADVS-13-e12538-s004.zip › Raw data of microscope images/Figure S5D-Vector.tif]

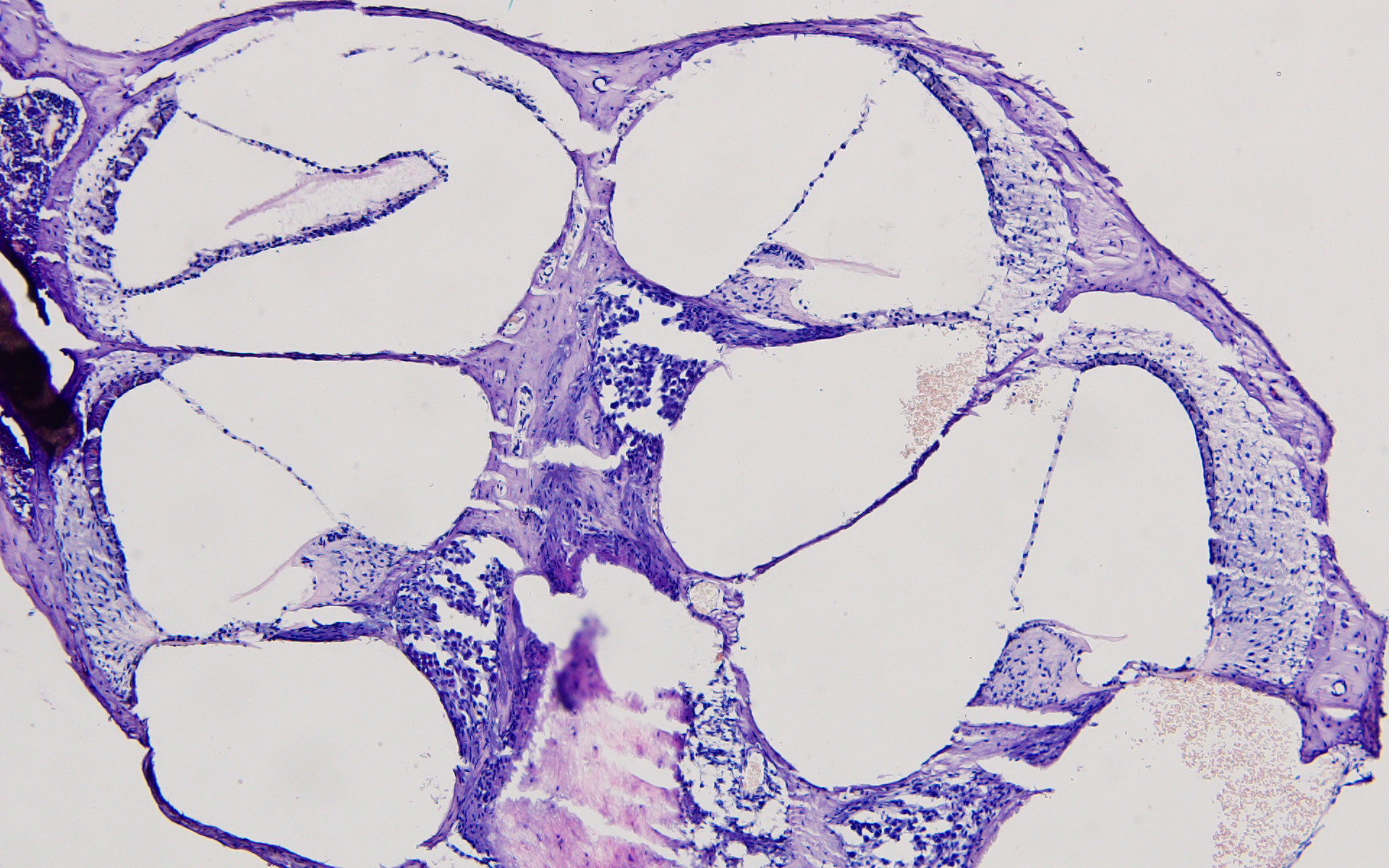

Supplement: Supplementary file 7 — Supporting File 7: advs75263‐sup‐0007‐Data5.zip. [file ADVS-13-e12538-s004.zip › Raw data of microscope images/Figure S7B-ACY.tif]

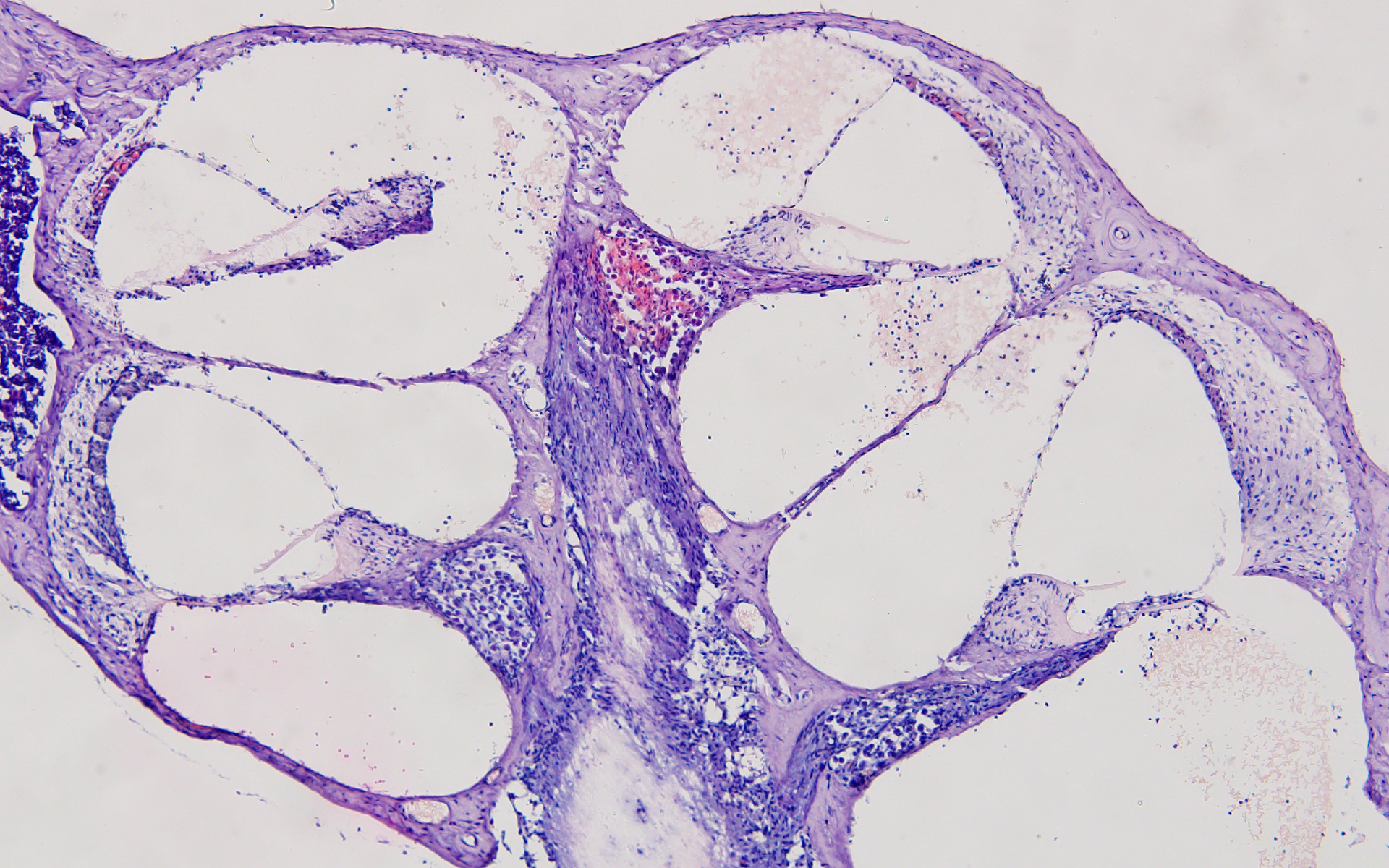

Supplement: Supplementary file 7 — Supporting File 7: advs75263‐sup‐0007‐Data5.zip. [file ADVS-13-e12538-s004.zip › Raw data of microscope images/Figure S7B-LPS+ACY.tif]

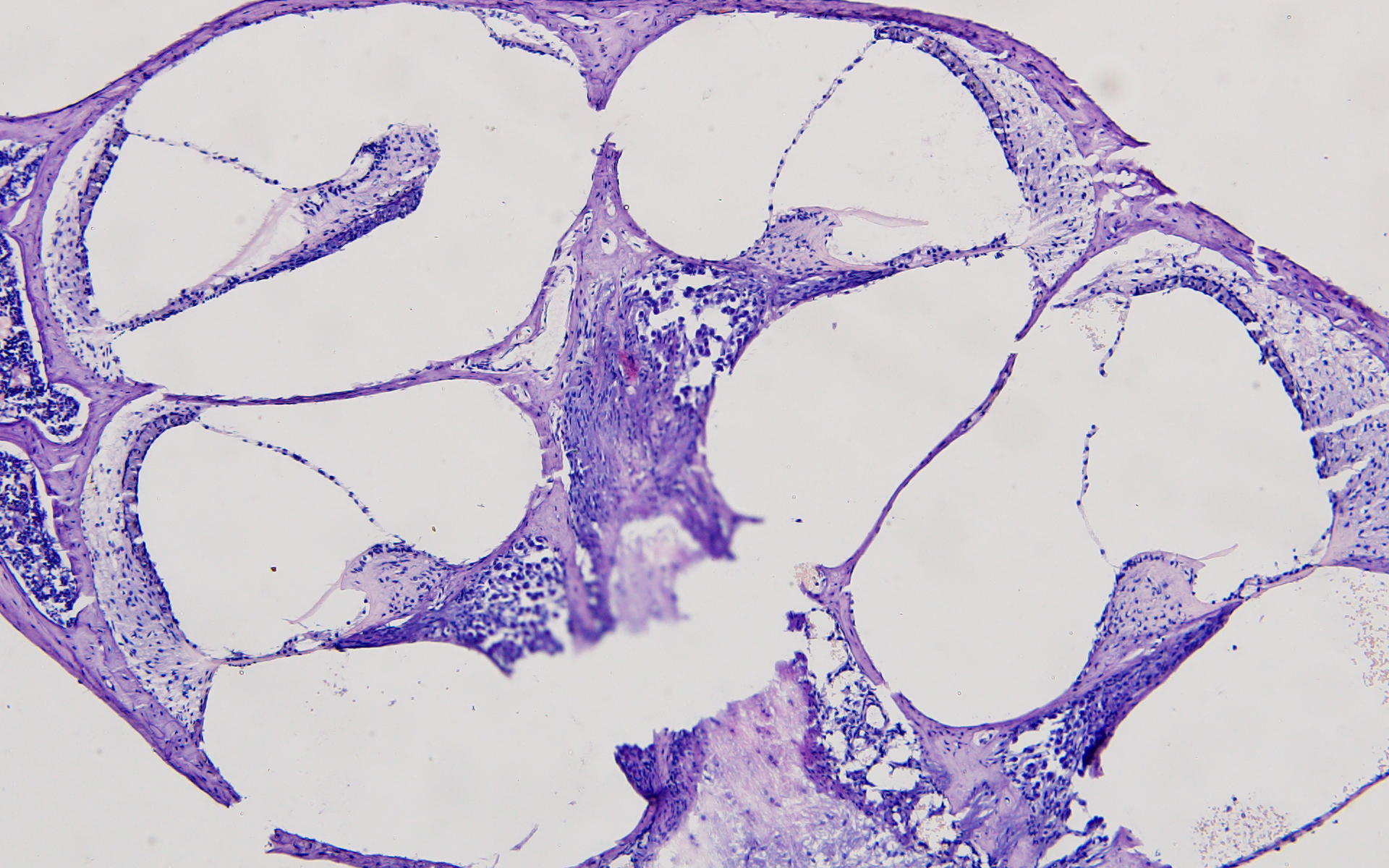

Supplement: Supplementary file 7 — Supporting File 7: advs75263‐sup‐0007‐Data5.zip. [file ADVS-13-e12538-s004.zip › Raw data of microscope images/Figure S7B-LPS.tif]

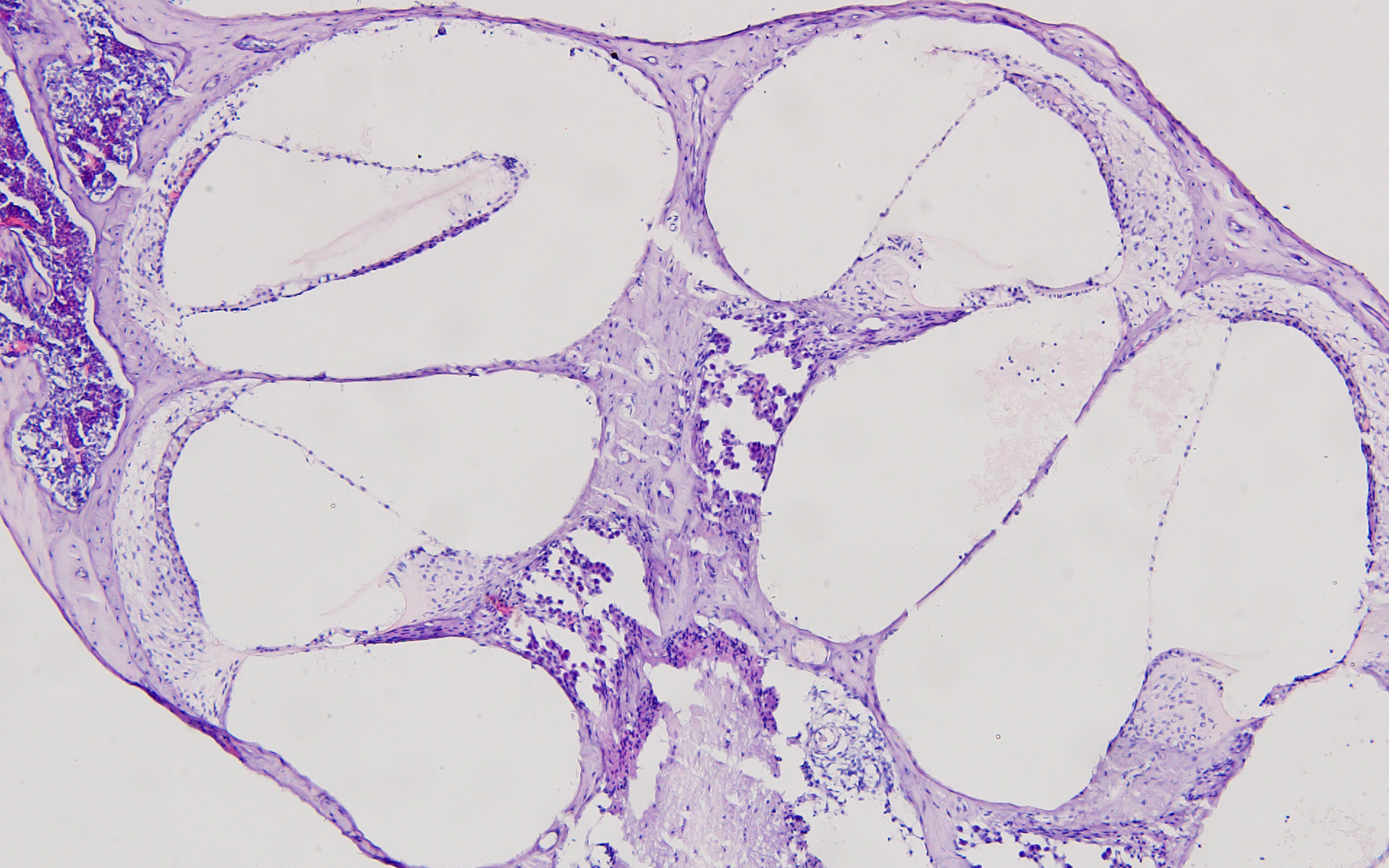

Supplement: Supplementary file 7 — Supporting File 7: advs75263‐sup‐0007‐Data5.zip. [file ADVS-13-e12538-s004.zip › Raw data of microscope images/Figure S7B-NS.tif]

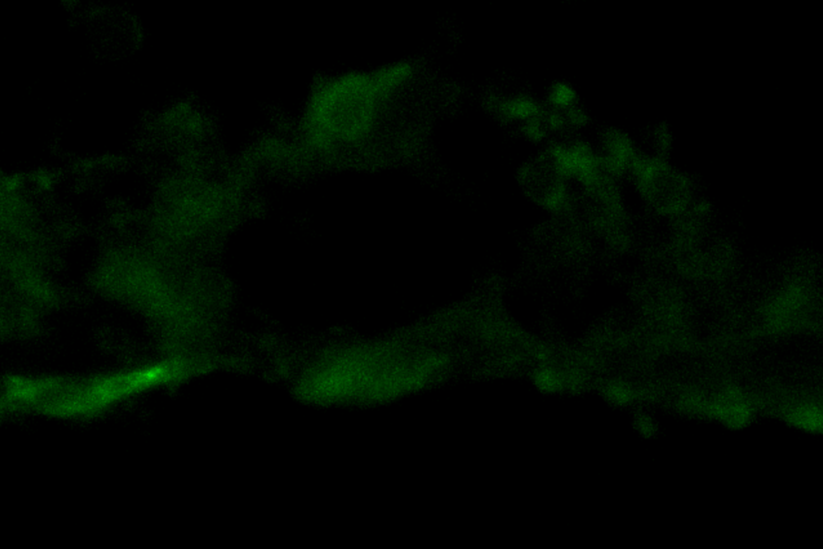

Supplement: Supplementary file 7 — Supporting File 7: advs75263‐sup‐0007‐Data5.zip. [file ADVS-13-e12538-s004.zip › Raw data of microscope images/Figure S7H-ACY.tif]

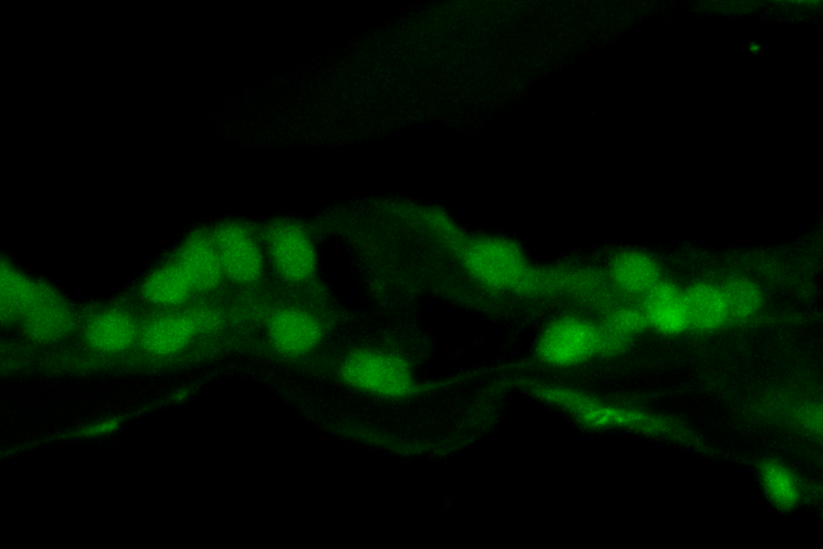

Supplement: Supplementary file 7 — Supporting File 7: advs75263‐sup‐0007‐Data5.zip. [file ADVS-13-e12538-s004.zip › Raw data of microscope images/Figure S7H-LPS+ACY.tif]

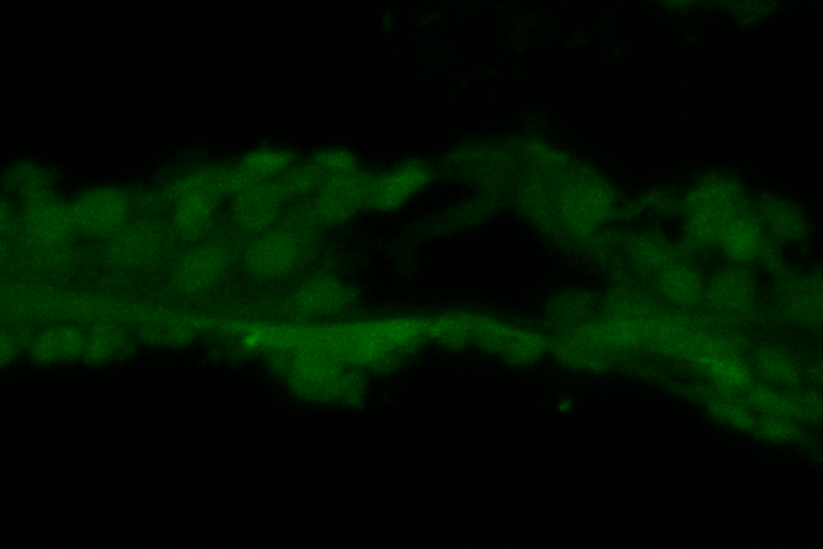

Supplement: Supplementary file 7 — Supporting File 7: advs75263‐sup‐0007‐Data5.zip. [file ADVS-13-e12538-s004.zip › Raw data of microscope images/Figure S7H-LPS.tif]

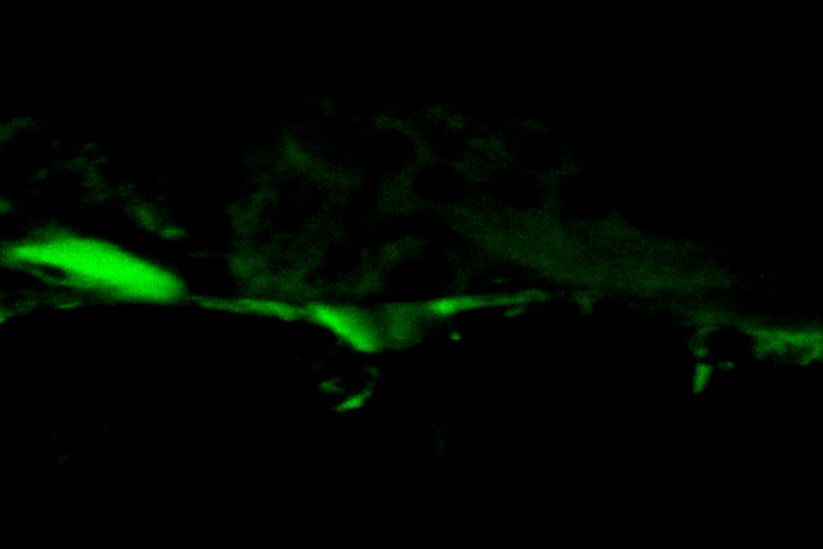

Supplement: Supplementary file 7 — Supporting File 7: advs75263‐sup‐0007‐Data5.zip. [file ADVS-13-e12538-s004.zip › Raw data of microscope images/Figure S7H-NS.tif]

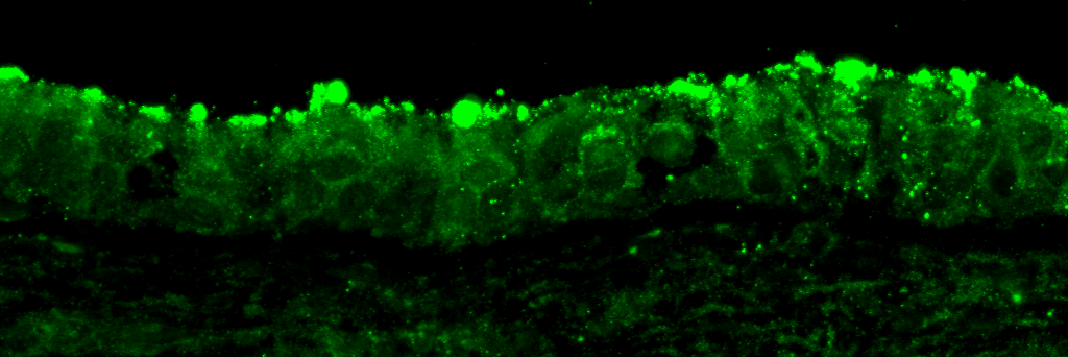

Supplement: Supplementary file 7 — Supporting File 7: advs75263‐sup‐0007‐Data5.zip. [file ADVS-13-e12538-s004.zip › Raw data of microscope images/Figure S9-MD (1) HtrA1.tif]

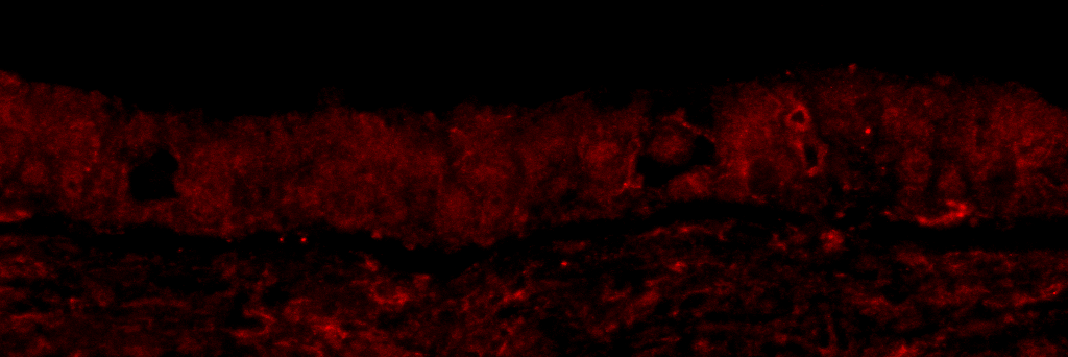

Supplement: Supplementary file 7 — Supporting File 7: advs75263‐sup‐0007‐Data5.zip. [file ADVS-13-e12538-s004.zip › Raw data of microscope images/Figure S9-MD (2) Myo7a.tif]

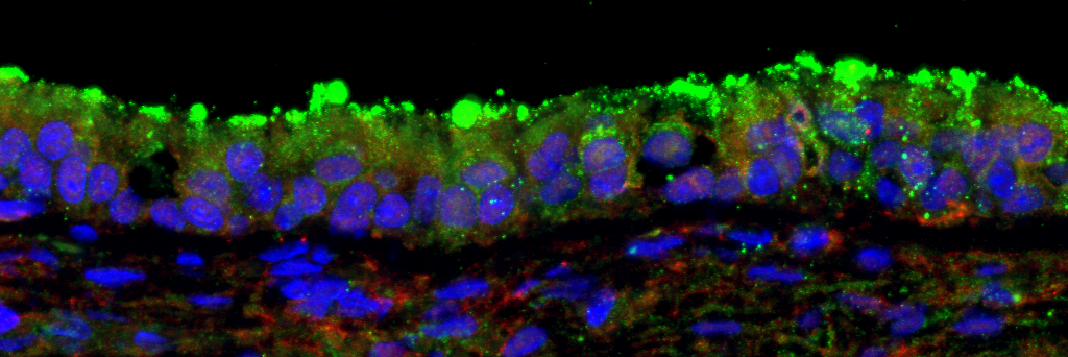

Supplement: Supplementary file 7 — Supporting File 7: advs75263‐sup‐0007‐Data5.zip. [file ADVS-13-e12538-s004.zip › Raw data of microscope images/Figure S9-MD (3) Merge.tif]

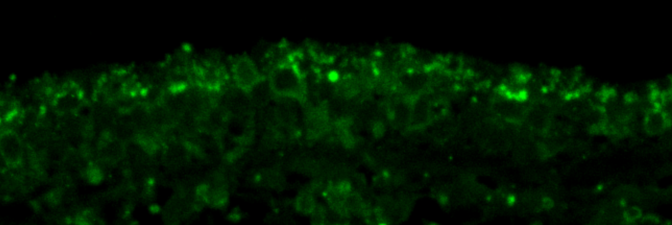

Supplement: Supplementary file 7 — Supporting File 7: advs75263‐sup‐0007‐Data5.zip. [file ADVS-13-e12538-s004.zip › Raw data of microscope images/Figure S9-VS (1) HtrA1.tif]

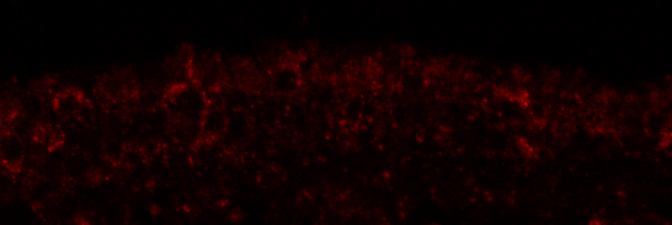

Supplement: Supplementary file 7 — Supporting File 7: advs75263‐sup‐0007‐Data5.zip. [file ADVS-13-e12538-s004.zip › Raw data of microscope images/Figure S9-VS (2) Myo7a.tif]

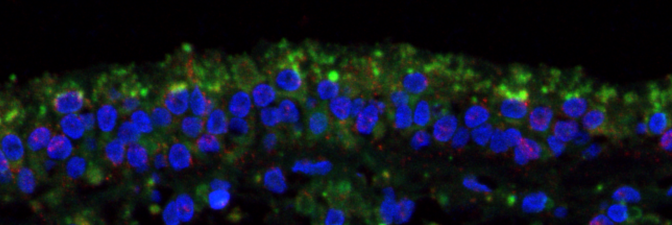

Supplement: Supplementary file 7 — Supporting File 7: advs75263‐sup‐0007‐Data5.zip. [file ADVS-13-e12538-s004.zip › Raw data of microscope images/Figure S9-VS (3) Merge.tif]
